# Supplementary figures and images for: Cardiotoxicity detection tool for breast cancer chemotherapy: a retrospective study
Source: PeerJ Comput Sci. 2024 Aug 2;12:e2230. doi: 10.7717/peerj-cs.2230 (PMC11323080; doi:10.7717/peerj-cs.2230)

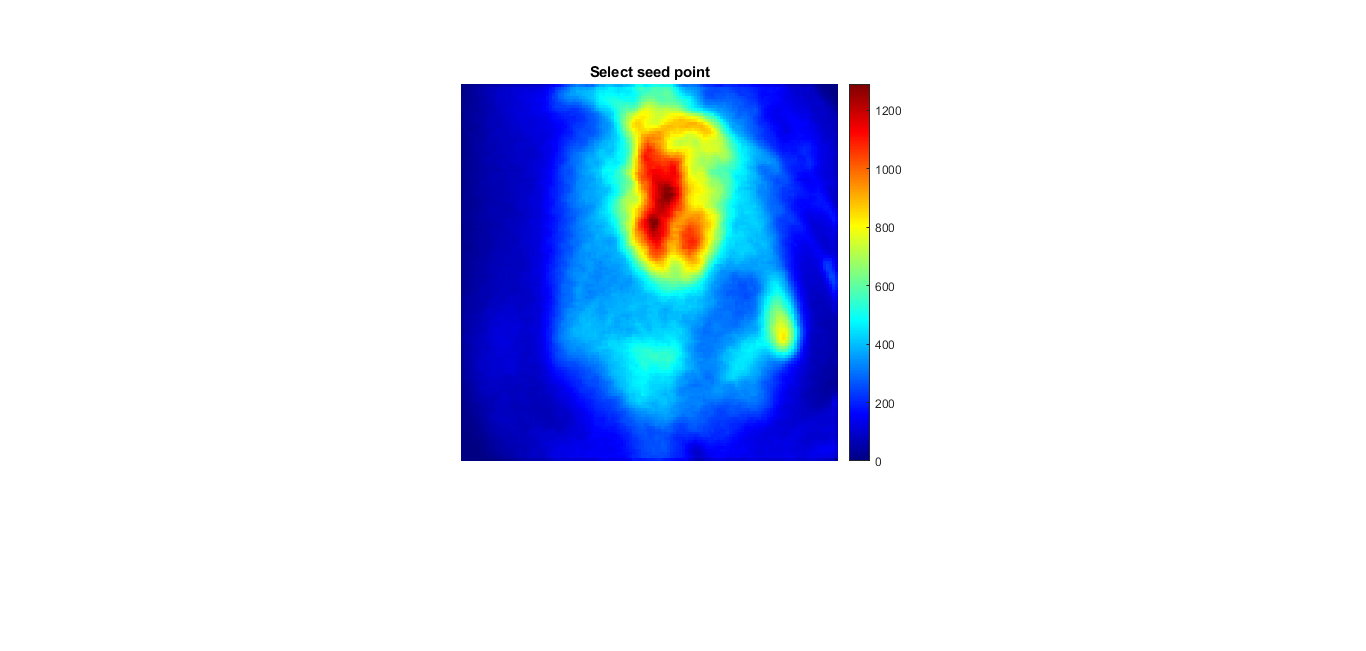

Supplement: Supplemental Information 13 — This offers a comprehensive framework for researchers to analyze parameters such as the left ventricle ejection fraction, dyssynchrony metrics, and numerous additional factors. [file peerj-cs-10-2230-s013.zip › Orig.bmp]

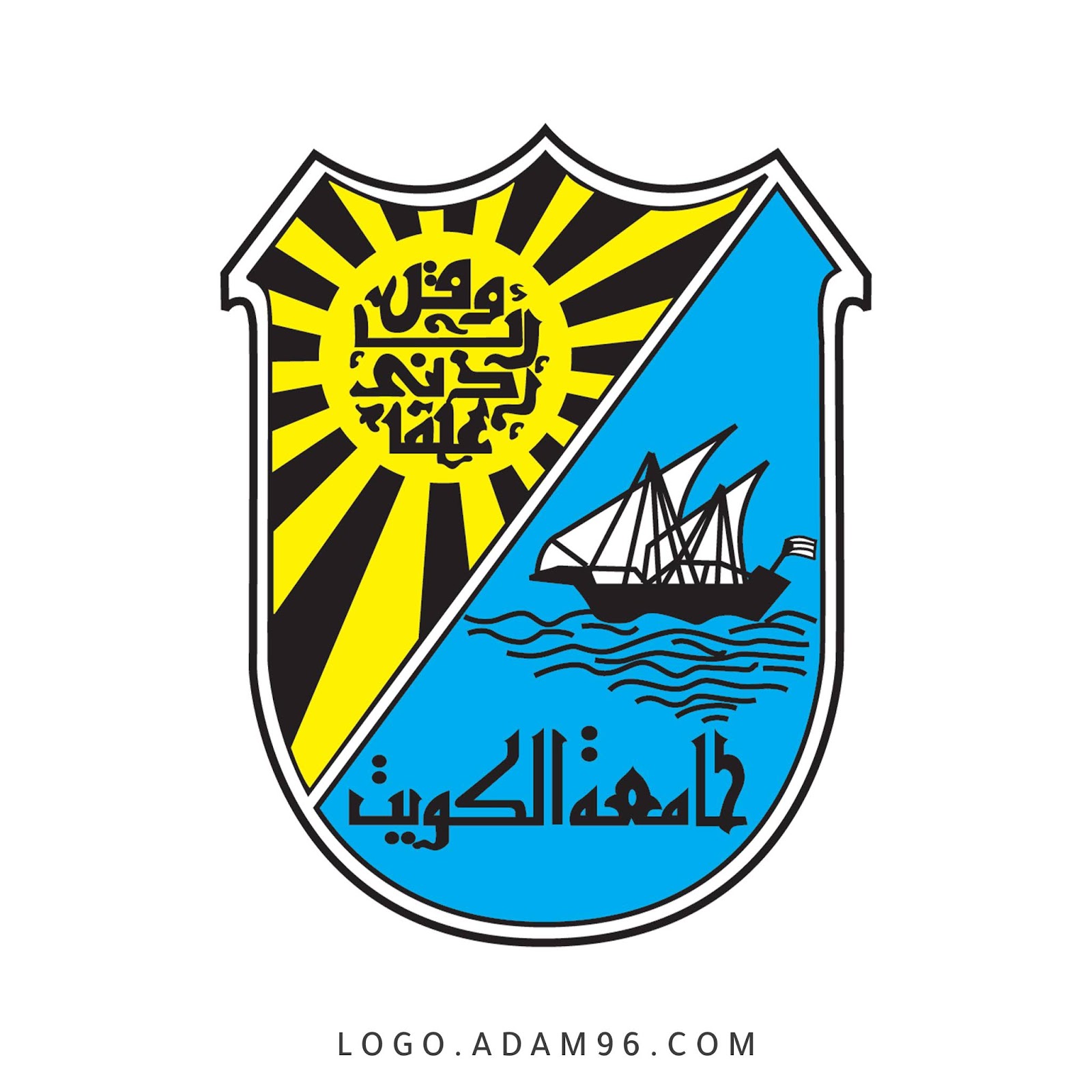

Supplement: Supplemental Information 13 — This offers a comprehensive framework for researchers to analyze parameters such as the left ventricle ejection fraction, dyssynchrony metrics, and numerous additional factors. [file peerj-cs-10-2230-s013.zip › logo.jpg]

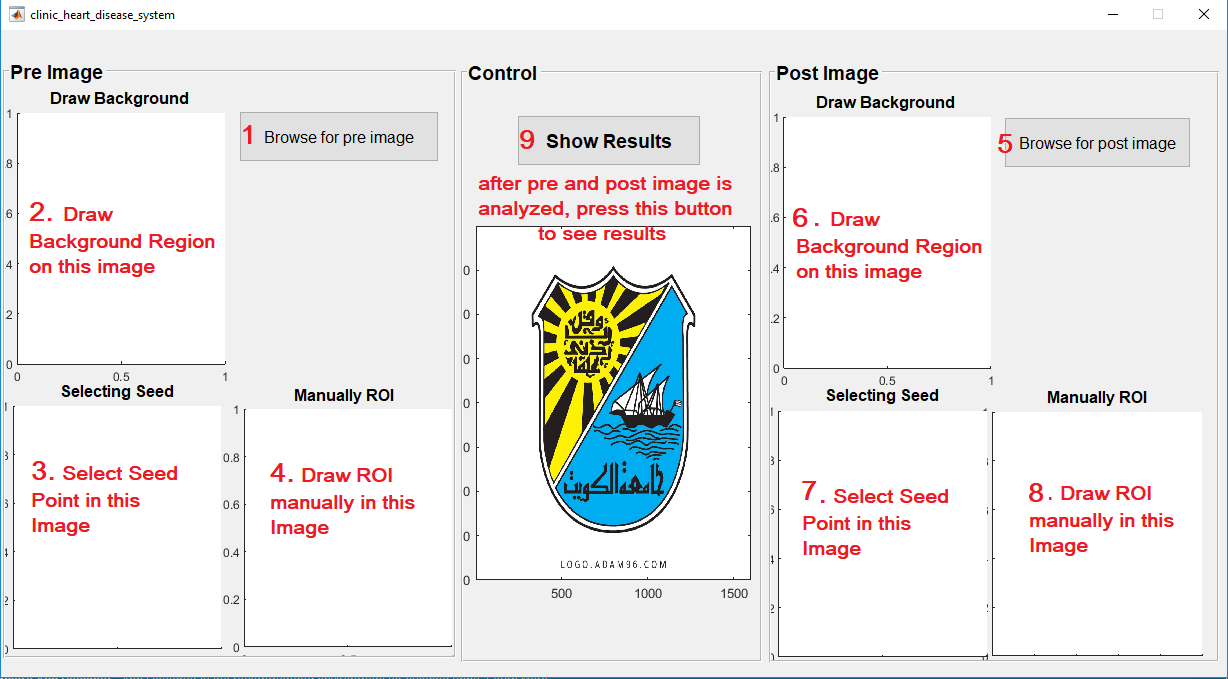

Supplement: Supplemental Information 13 — This offers a comprehensive framework for researchers to analyze parameters such as the left ventricle ejection fraction, dyssynchrony metrics, and numerous additional factors. [file peerj-cs-10-2230-s013.zip › help.png]
