# Supplementary material for: Cardiotoxicity detection tool for breast cancer chemotherapy: a retrospective study
Source: PeerJ Comput Sci. 2024 Aug 2;12:e2230. doi: 10.7717/peerj-cs.2230 (PMC11323080; doi:10.7717/peerj-cs.2230)
Supplement: Supplemental Information 14 — This offers a comprehensive framework for researchers to analyze parameters such as the left ventricle ejection fraction, dyssynchrony metrics, and numerous additional factors. [file peerj-cs-10-2230-s014.docx]

Appendix B: Table of code appendices: Corresponding codes are available in separate pages. Codes mentioned in this appendix are either modified or novel work. Function codes are denoted as Fun.

| S | M-file name | Type |
| --- | --- | --- |
|  | [ApEn_Slow](#ApEn_Slow) | Fun |
|  | [ApEnBoundEnForUnifron_ noisy_signal](#ApEnBoundEnForUnifron_noisy_signal) | Script |
|  | [BoundedProcess](#BoundedProcess) | Fun |
|  | [Chenvese](#Chenvese) | Fun |
|  | [clinic_heart_disease_system](#clinic_heart_disease_system) | GUI (m file) |
|  | [clinic_heart_disease_system.fig](#clinic_heart_disease_systemFig) | Figure file |
|  | [computeEntropySynchrony](#computeEntropySynchrony) | Fun |
|  | [computeEntropySynchronyNo](#computeEntropySynchronyNo) | Fun |
|  | [Dialogue_box_pre_post.m](#Dialogue_box_pre_post) | M file of GUI |
|  | [Dialogue_box_pre_post.fig](#Dialogue_box_pre_post) | Figure file (design) |
|  | [energy_Pcount _Gcount_iteration_To_excel](#energy_Pcount_Gcount_iteration_To_excel) | Script |
|  | [EntropySig](#EntropySig) | Fun |
|  | [exportInfo2Excel](#exportInfo2Excel) | Script |
|  | [fit_ellipse](#fit_ellipse) | Fun |
|  | [Image_ Entropy_ Experiment.m](#Image_Entropy_Experiment) | Script |
|  | [Image_ Entropy_ ExperimentPoisson.m](#Image_Entropy_ExperimentPoisson) | Script |
|  | [LVtimeActivityCurve.m](#LVtimeActivityCurve) | Fun |
|  | [mask_all.m](#mask_all) | Fun |
|  | [maskall3.m](#maskall3) | Fun |
|  | [max_min_MUGA.m](#max_min_MUGA) | Fun |
|  | [max_min_MUGA3.m](#max_min_MUGA3) | Fun |
|  | [MyHistogram](#MyHistogram) | Fun |
|  | [PhaseBkg16Images](#MyHistogram) | Script |
|  | [PhaseBkgFn](#PhaseBkgFn) | Fun |
|  | [phaseImageAll_ROIs](#phaseImageAll) | Script |
|  | [PlotEpsilonVsApEn](#PlotEpsilonVsApEn) | Script |
|  | [regionGrowing2](#regionGrowing2) | Fun |
|  | [regionGrowing3](#regionGrowing3) | Fun |
|  | [regionGrowingX](#regiongrowingX) | Fun |
|  | [ROI_max_min](#ROI_max_min) | Fun |
|  | [ROI_max_min3](#ROI_max_min3) | Fun |
|  | [Filtering_ Experiment_MSI_PSNR](#Filtering_Experiment_MSI_PSNR) | Script |
|  | [MMWF_2D.m](#MMWF_2D) | Fun |
|  | [elongation](#elongation).m | Script |
|  | [Image_Entropy_ Experiment_ New_Version_ Possion_ Gaussian](#Image_Entropy_Experiment_New_Version_Pos) | Script |
|  | [filtering_ experiment_ new_version_ fixed_noise_level](#filtering_experiment_new_version_fix) | Script |
|  | [Image_ Entropy_ Experiment_ modified_ Whole_Image](#Image_Entropy_Experiment_modified_Whole) | Script |
|  | [Image_ Entropy_ Experiment_ Possion_Gaussian_ ColumnScanning](#Image_Entropy_ColumnScanning) | Script |
|  | [Filtering_ Experiment _CNR _SR_MSI_PSNR](#Filtering_Experiment_CNR_SR_MSI_PSNR) | Script |
|  | [cnr](#cnr) | Fun |
|  | [Dos](#dos) | Fun |

| **M-file name** | ApEn_Slow |
| --- | --- |
| **Type** | Fun (Function) |
| **Description** | Slow implementation of approximate entropy.  **Inputs**: x : (a 1-d vector) input signal, m : (positive integer value) Embedding dimension  r : (non-negative real value) Tolerance parameter  **Output**: y : ApEn (y is always defined) |
| **comments** | Open source  Reference of ApEn: (209) |

**Code:**

function y = ApEn_slow(x, m, r)

% Slow implementation of approximate entropy (ApEn).

% Written by: Amir Omidvarnia, PhD

% Email: a.omidvarnia@brain.org.au

%

% Reference: S. M. Pincus, “Approximate entropy as a measure of system complexity.,”

% Proc. Natl. Acad. Sci., vol. 88, no. 6, pp. 2297–2301, Mar. 1991.

%

% Inputs:

% x : (a 1-d vector) input signal

% m : (positive integer value) Embedding dimension

% r : (non-negative real value) Tolerance parameter

% Output:

% y : ApEn (y is always defined)

%

% Example:

% x = rand(1,1000);

% y = ApEn_slow(x, 5, 0.2);

N = length(x);

phi_m_r = zeros(1,2);

m2 = m;

for ss = 1 : 2

m2 = m2 + (ss-1);

C = zeros(1,(N-m2+1));

for i = 1 : (N-m2+1)

x_i = x(i:(i+m2-1)); % Template

d = zeros(1,(N-m2+1));

for j = 1 : (N-m2+1)

x_j = x(j:(j+m2-1)); % Template

d(j) = max(abs(x_i - x_j)); % d_chebyshev

end

C(i) = sum(d<=r)/(N-m2+1);

end

phi_m_r(ss) = sum(log(C))/(N-m2+1);

end

y = phi_m_r(1) - phi_m_r(2);

%%%%%%%%%%%%%%%%%%%%%%%%%%%%%%%%%%%%%%%%%%%%%%%%

| **M-file name** | ApEnBoundEnForUnifron_ noisy_signal |
| --- | --- |
| **Type** | Script |
| **Description** | **Experiment**:  Generating uniform signal then applying two types of noise (pulse and multiplicative)  After that compute the ApEn and Bounded ApEn for each one |
| **comments** | Novel experiment written by us in 31-5-2021. |

**Code**:

%% Generating uniform signal then appying two types of noise (pulse and multiplicative)

%% After that compute the ApEn and Bounded ApEn for each one

% tolerance r and m parameters\

r_val=1;

m_val=2;

% Sine Signal

Fs = 8000; % samples per second

dt = 1/Fs; % seconds per sample

StopTime = 0.125; % seconds

t = (0:dt:StopTime-dt)'; % seconds

%%Sine wave:

Fc = 60; % hertz

s1 = sin(2*pi*Fc*t);

% Plot the signal versus time:

figure;

plot(t,s1);

xlabel('time (in seconds)');

title('Signal versus Time');

% define noise initiation and increament

init=0;

inc=0.03;

% noise

s2=imnoise(s1,'salt & pepper',init+inc);

s3=imnoise(s1,'salt & pepper',init+2*inc);

s4=imnoise(s1,'salt & pepper',init+3*inc);

s5=imnoise(s1,'salt & pepper',init+4*inc);

s6=imnoise(s1,'salt & pepper',init+5*inc);

s7=imnoise(s1,'salt & pepper',init+6*inc);

s8=imnoise(s1,'salt & pepper',init+7*inc);

s9=imnoise(s1,'salt & pepper',init+8*inc);

s10=imnoise(s1,'salt & pepper',init+9*inc);

%plot(s1(1:100));title('Original uniform signal');

figure

subplot(3,3,1),plot(s2);title('pulse nosiy 0.1 signal');

subplot(3,3,2),plot(s3);title('pulse nosiy 0.2 signal');

subplot(3,3,3),plot(s4);title('pulse nosiy 0.3 signal');

subplot(3,3,4),plot(s5);title('pulse nosiy 0.4 signal');

subplot(3,3,5),plot(s6);title('pulse nosiy 0.5 signal');

subplot(3,3,6),plot(s7);title('pulse nosiy 0.6 signal');

subplot(3,3,7),plot(s8);title('pulse nosiy 0.7 signal');

subplot(3,3,8),plot(s9);title('pulse nosiy 0.8 signal');

subplot(3,3,9),plot(s10);title('pulse nosiy 0.9 signal');

[en1]=EntropySig(s1)

[en2]=EntropySig(s2)

[en3]=EntropySig(s3)

[en4]=EntropySig(s4)

[en5]=EntropySig(s5)

[en6]=EntropySig(s6)

[en7]=EntropySig(s7)

[en8]=EntropySig(s8)

[en9]=EntropySig(s9)

[en10]=EntropySig(s10)

ApEn1= ApEn_slow(s1, m_val,r_val*std(s1))

ApEn2= ApEn_slow(s2, m_val,r_val*std(s2))

ApEn3= ApEn_slow(s3, m_val,r_val*std(s3))

ApEn4= ApEn_slow(s4, m_val,r_val*std(s4))

ApEn5= ApEn_slow(s5, m_val,r_val*std(s5))

ApEn6= ApEn_slow(s6, m_val,r_val*std(s6))

ApEn7= ApEn_slow(s7, m_val,r_val*std(s7))

ApEn8= ApEn_slow(s8, m_val,r_val*std(s8))

ApEn9= ApEn_slow(s9, m_val,r_val*std(s9))

ApEn10= ApEn_slow(s10, m_val,r_val*std(s10))

[BoundApEn1]=BoundedProcess(s1);

[BoundApEn2]=BoundedProcess(s2);

[BoundApEn3]=BoundedProcess(s3);

[BoundApEn4]=BoundedProcess(s4);

[BoundApEn5]=BoundedProcess(s5);

[BoundApEn6]=BoundedProcess(s6);

[BoundApEn7]=BoundedProcess(s7);

[BoundApEn8]=BoundedProcess(s8);

[BoundApEn9]=BoundedProcess(s9);

[BoundApEn10]=BoundedProcess(s10);

filename = 'Entropy.xlsx';

T1= table({en1},{en2},{en3},{en4},{en5},{en6},{en7},{en8},{en9},{en10});

T2 = table({ApEn1},{ApEn2},{ApEn3},{ApEn4},{ApEn5},{ApEn6},{ApEn7},{ApEn8},{ApEn9},{ApEn10});

T3 = table({BoundApEn1},{BoundApEn2},{BoundApEn3},{BoundApEn4},{BoundApEn5},{BoundApEn6},{BoundApEn7},{BoundApEn8},{BoundApEn9},{BoundApEn10});

writetable(T1,filename,'Sheet','Sheet1','WriteVariableNames',false,'Range',strcat('B3:k3'));

writetable(T2,filename,'Sheet','Sheet1','WriteVariableNames',false,'Range',strcat('B4:k4'));

writetable(T3,filename,'Sheet','Sheet1','WriteVariableNames',false,'Range',strcat('B5:k5'));

%%

s2=imnoise(s1,'speckle',init+inc);

s3=imnoise(s1,'speckle',init+2*inc);

s4=imnoise(s1,'speckle',init+3*inc);

s5=imnoise(s1,'speckle',init+4*inc);

s6=imnoise(s1,'speckle',init+5*inc);

s7=imnoise(s1,'speckle',init+6*inc);

s8=imnoise(s1,'speckle',init+7*inc);

s9=imnoise(s1,'speckle',init+8*inc);

s10=imnoise(s1,'speckle',init+9*inc);

figure

subplot(3,3,1),plot(s2);title('speckle nosiy 0.1 signal');

subplot(3,3,2),plot(s3);title('speckle nosiy 0.2 signal');

subplot(3,3,3),plot(s4);title('speckle nosiy 0.3 signal');

subplot(3,3,4),plot(s5);title('speckle nosiy 0.4 signal');

subplot(3,3,5),plot(s6);title('speckle nosiy 0.5 signal');

subplot(3,3,6),plot(s7);title('speckle nosiy 0.6 signal');

subplot(3,3,7),plot(s8);title('speckle nosiy 0.7 signal');

subplot(3,3,8),plot(s9);title('speckle nosiy 0.8 signal');

subplot(3,3,9),plot(s10);title('speckle nosiy 0.9 signal');

[en1]=EntropySig(s1)

[en2]=EntropySig(s2)

[en3]=EntropySig(s3)

[en4]=EntropySig(s4)

[en5]=EntropySig(s5)

[en6]=EntropySig(s6)

[en7]=EntropySig(s7)

[en8]=EntropySig(s8)

[en9]=EntropySig(s9)

[en10]=EntropySig(s10)

ApEn1= ApEn_slow(s1, m_val,r_val*std(s1))

ApEn2= ApEn_slow(s2, m_val,r_val*std(s2))

ApEn3= ApEn_slow(s3, m_val,r_val*std(s3))

ApEn4= ApEn_slow(s4, m_val,r_val*std(s4))

ApEn5= ApEn_slow(s5, m_val,r_val*std(s5))

ApEn6= ApEn_slow(s6, m_val,r_val*std(s6))

ApEn7= ApEn_slow(s7, m_val,r_val*std(s7))

ApEn8= ApEn_slow(s8, m_val,r_val*std(s8))

ApEn9= ApEn_slow(s9, m_val,r_val*std(s9))

ApEn10= ApEn_slow(s10, m_val,r_val*std(s10))

[BoundApEn1]=BoundedProcess(s1);

[BoundApEn2]=BoundedProcess(s2);

[BoundApEn3]=BoundedProcess(s3);

[BoundApEn4]=BoundedProcess(s4);

[BoundApEn5]=BoundedProcess(s5);

[BoundApEn6]=BoundedProcess(s6);

[BoundApEn7]=BoundedProcess(s7);

[BoundApEn8]=BoundedProcess(s8);

[BoundApEn9]=BoundedProcess(s9);

[BoundApEn10]=BoundedProcess(s10);

filename = 'Entropy.xlsx';

[ApEn1 ApEn2 ApEn3 ApEn4 ApEn5 ApEn6 ApEn7 ApEn8 ApEn9 ApEn10...

BoundApEn1 BoundApEn2 BoundApEn3 BoundApEn4 BoundApEn5...

BoundApEn6 BoundApEn7 BoundApEn8 BoundApEn9 BoundApEn10]

T1= table({en1},{en2},{en3},{en4},{en5},{en6},{en7},{en8},{en9},{en10});

T2 = table({ApEn1},{ApEn2},{ApEn3},{ApEn4},{ApEn5},{ApEn6},{ApEn7},{ApEn8},{ApEn9},{ApEn10});

T3 = table({BoundApEn1},{BoundApEn2},{BoundApEn3},{BoundApEn4},{BoundApEn5},{BoundApEn6},{BoundApEn7},{BoundApEn8},{BoundApEn9},{BoundApEn10});

writetable(T1,filename,'Sheet','Sheet1','WriteVariableNames',false,'Range',strcat('B8:k8'));

writetable(T2,filename,'Sheet','Sheet1','WriteVariableNames',false,'Range',strcat('B9:k9'));

writetable(T3,filename,'Sheet','Sheet1','WriteVariableNames',false,'Range',strcat('B10:k10'));

%%

s2=imnoise(s2,'salt & pepper',init+inc);

s3=imnoise(s3,'salt & pepper',init+2*inc);

s4=imnoise(s4,'salt & pepper',init+3*inc);

s5=imnoise(s5,'salt & pepper',init+4*inc);

s6=imnoise(s6,'salt & pepper',init+5*inc);

s7=imnoise(s7,'salt & pepper',init+6*inc);

s8=imnoise(s8,'salt & pepper',init+7*inc);

s9=imnoise(s9,'salt & pepper',init+8*inc);

s10=imnoise(s10,'salt & pepper',init+9*inc);

%plot(s1(1:100));title('Original uniform signal');

figure

subplot(3,3,1),plot(s2);title('mixed nosiy 0.1 signal');

subplot(3,3,2),plot(s3);title('mixed nosiy 0.2 signal');

subplot(3,3,3),plot(s4);title('mixed nosiy 0.3 signal');

subplot(3,3,4),plot(s5);title('mixed nosiy 0.4 signal');

subplot(3,3,5),plot(s6);title('mixed nosiy 0.5 signal');

subplot(3,3,6),plot(s7);title('mixed nosiy 0.6 signal');

subplot(3,3,7),plot(s8);title('mixed nosiy 0.7 signal');

subplot(3,3,8),plot(s9);title('mixed nosiy 0.8 signal');

subplot(3,3,9),plot(s10);title('mixed nosiy 0.9 signal');

[en1]=EntropySig(s1)

[en2]=EntropySig(s2)

[en3]=EntropySig(s3)

[en4]=EntropySig(s4)

[en5]=EntropySig(s5)

[en6]=EntropySig(s6)

[en7]=EntropySig(s7)

[en8]=EntropySig(s8)

[en9]=EntropySig(s9)

[en10]=EntropySig(s10)

ApEn1= ApEn_slow(s1, m_val,r_val*std(s1))

ApEn2= ApEn_slow(s2, m_val,r_val*std(s2))

ApEn3= ApEn_slow(s3, m_val,r_val*std(s3))

ApEn4= ApEn_slow(s4, m_val,r_val*std(s4))

ApEn5= ApEn_slow(s5, m_val,r_val*std(s5))

ApEn6= ApEn_slow(s6, m_val,r_val*std(s6))

ApEn7= ApEn_slow(s7, m_val,r_val*std(s7))

ApEn8= ApEn_slow(s8, m_val,r_val*std(s8))

ApEn9= ApEn_slow(s9, m_val,r_val*std(s9))

ApEn10= ApEn_slow(s10, m_val,r_val*std(s10))

[BoundApEn1]=BoundedProcess(s1);

[BoundApEn2]=BoundedProcess(s2);

[BoundApEn3]=BoundedProcess(s3);

[BoundApEn4]=BoundedProcess(s4);

[BoundApEn5]=BoundedProcess(s5);

[BoundApEn6]=BoundedProcess(s6);

[BoundApEn7]=BoundedProcess(s7);

[BoundApEn8]=BoundedProcess(s8);

[BoundApEn9]=BoundedProcess(s9);

[BoundApEn10]=BoundedProcess(s10);

filename = 'Entropy.xlsx';

T1= table({en1},{en2},{en3},{en4},{en5},{en6},{en7},{en8},{en9},{en10});

T2 = table({ApEn1},{ApEn2},{ApEn3},{ApEn4},{ApEn5},{ApEn6},{ApEn7},{ApEn8},{ApEn9},{ApEn10});

T3 = table({BoundApEn1},{BoundApEn2},{BoundApEn3},{BoundApEn4},{BoundApEn5},{BoundApEn6},{BoundApEn7},{BoundApEn8},{BoundApEn9},{BoundApEn10});

writetable(T1,filename,'Sheet','Sheet1','WriteVariableNames',false,'Range',strcat('B13:k13'));

writetable(T2,filename,'Sheet','Sheet1','WriteVariableNames',false,'Range',strcat('B14:k14'));

writetable(T3,filename,'Sheet','Sheet1','WriteVariableNames',false,'Range',strcat('B15:k15'));

%%

disp('Data has been written to excel sheet, check Entropy.xlsx in the current folder!');

%%%%%%%%%%%%%%%%%%%%%%%%%%%%%%%%%%%%%%%%%%%%%%%%

| **M-file name** | BoundedProcess |
| --- | --- |
| **Type** | Fun |
| **Description** | Compute the bounded approximate entropy, epsilon and log(epsilon) |
| **comments** | Novel experiment written by us in 21-5-2021 depending on (190) |

**Code:**

function [BoundApEn,epsilon,Lespsilon] = BoundedProcess(A)

% Compute the bounded approximate entropy, epsilon and log(epsilon)

% Input:

% A: The vector that Bounded process will be computed for

% Outputs:

% BoundedApEn: the bounded apen, epsilon and log(epsilon)

A=A./max(A);

BoundP=max(A);

BoundM=min(A);

k=0;

for i=2:1:size(A,1)

e(i)=A(i)-A(i-1);

if(e(i)>BoundP || e(i)<BoundM)

e(i)=e(i)+A(i-1);

k=k+1;

end

end

alpha=std(e);

betta=BoundP-BoundM;

epsilon=alpha/betta;

Lespsilon=log(epsilon) ;% log(epsilon)

r=1*std(e);% tolerance parameter

%BoundApEn=-log((2*sqrt(12)*alpha/betta+r.*2)/(12*((alpha/betta).^2))) %bounded aproaximate entropy

%BoundApEn=log(sqrt(3))+log(epsilon)-log(r) %bounded aproaximate entropy

BoundApEn=ApEn_slow(e,2,r);

end

%%%%%%%%%%%%%%%%%%%%%%%%%%%%%%%%%%%%%%%%%%%%%%%%

| **M-file name** | Chenvese |
| --- | --- |
| **Type** | Fun |
| **Description** | Active contour (edge-based and region based) |
| **comments** | Modified from Open source  Implemented by Yue Wu, Tufts University, 2009.(244) |

**Code:**

%==========================================================================

%

% Active contour with Chen-Vese Method

% for image segementation

%

% Implemented by Yue Wu (yue.wu@tufts.edu)

% Tufts University

% Feb 2009

% http://sites.google.com/site/rexstribeofimageprocessing/

%

% all rights reserved

% Last update 02/26/2009

%--------------------------------------------------------------------------

% Usage of varibles:

% input:

% I = any gray/double/RGB input image

% mask = initial mask, either customerlized or built-in

% num_iter = total number of iterations

% mu = weight of length term

% method = submethods pick from ('chen','vector','multiphase')

%

% Types of built-in mask functions

% 'small' = create a small circular mask

% 'medium' = create a medium circular mask

% 'large' = create a large circular mask

% 'whole' = create a mask with holes around

% 'whole+small' = create a two layer mask with one layer small

% circular mask and the other layer with holes around

% (only work for method 'multiphase')

% Types of methods

% 'chen' = general CV method

% 'vector' = CV method for vector image

% 'multiphase'= CV method for multiphase (2 phases applied here)

%

% output:

% phi0 = updated level set function

%

%--------------------------------------------------------------------------

%

% Description: This code implements the paper: "Active Contours Without

% Edges" by Chan and Vese for method 'chen', the paper:"Active Contours Without

% Edges for vector image" by Chan and Vese for method 'vector', and the paper

% "A Multiphase Level Set Framework for Image Segmentation Using the

% Mumford and Shah Model" by Chan and Vese.

%

%--------------------------------------------------------------------------

% Deomo: Please see HELP file for details

%==========================================================================

function [seg force] = chenvese(I,mask,num_iter,mu,method)

%%

%-- Default settings

% length term mu = 0.2 and default method = 'chan'

if(~exist('mu','var'))

mu=0.2;

end

if(~exist('method','var'))

method = 'chan';

end

%-- End default settings

%%

%-- Initializations on input image I and mask

% resize original image

s = 200./min(size(I,1),size(I,2)); % resize scale

if s<1

I = imresize(I,s);

end

% auto mask settings

if ischar(mask)

switch lower (mask)

case 'small'

mask = maskcircle2(I,'small');

case 'medium'

mask = maskcircle2(I,'medium');

case 'large'

mask = maskcircle2(I,'large');

case 'whole'

mask = maskcircle2(I,'whole');

%mask = init_mask(I,30);

case 'whole+small'

m1 = maskcircle2(I,'whole');

m2 = maskcircle2(I,'small');

mask = zeros(size(I,1),size(I,2),2);

mask(:,:,1) = m1(:,:,1);

mask(:,:,2) = m2(:,:,2);

otherwise

error('unrecognized mask shape name (MASK).');

end

else

if s<1

mask = imresize(mask,s);

end

if size(mask,1)>size(I,1) || size(mask,2)>size(I,2)

error('dimensions of mask unmathch those of the image.')

end

switch lower(method)

case 'multiphase'

if (size(mask,3) == 1)

error('multiphase requires two masks but only gets one.')

end

end

end

switch lower(method)

case 'chan'

if size(I,3)== 3

P = rgb2gray(uint8(I));

P = double(P);

elseif size(I,3) == 2

P = 0.5.*(double(I(:,:,1))+double(I(:,:,2)));

else

P = double(I);

end

layer = 1;

case 'vector'

s = 200./min(size(I,1),size(I,2)); % resize scale

I = imresize(I,s);

mask = imresize(mask,s);

layer = size(I,3);

if layer == 1

display('only one image component for vector image')

end

P = double(I);

case 'multiphase'

layer = size(I,3);

if size(I,1)*size(I,2)>200^2

s = 200./min(size(I,1),size(I,2)); % resize scale

I = imresize(I,s);

mask = imresize(mask,s);

end

P = double(I); %P store the original image

otherwise

error('!invalid method')

end

%-- End Initializations on input image I and mask

%%

%-- Core function

switch lower(method)

case {'chan','vector'}

%-- SDF

% Get the distance map of the initial mask

mask = mask(:,:,1);

phi0 = bwdist(mask)-bwdist(1-mask)+im2double(mask)-.5;

% initial force, set to eps to avoid division by zeros

force = eps;

%-- End Initialization

%-- Display settings

%figure();

%subplot(2,2,1); imshow(I); title('Input Image');

%subplot(2,2,2); contour(flipud(phi0), [0 0], 'r','LineWidth',1); title('initial contour');

%subplot(2,2,3); title('Segmentation');

%-- End Display original image and mask

%-- Main loop

for n=1:num_iter

inidx = find(phi0>=0); % frontground index

outidx = find(phi0<0); % background index

force_image = 0; % initial image force for each layer

for i=1:layer

L = im2double(P(:,:,i)); % get one image component

c1 = sum(sum(L.*Heaviside(phi0)))/(length(inidx)+eps); % average inside of Phi0

c2 = sum(sum(L.*(1-Heaviside(phi0))))/(length(outidx)+eps); % verage outside of Phi0

force_image=-(L-c1).^2+(L-c2).^2+force_image;

% sum Image Force on all components (used for vector image)

% if 'chan' is applied, this loop become one sigle code as a

% result of layer = 1

end

% calculate the external force of the image

force = mu*kappa(phi0)./max(max(abs(kappa(phi0))))+1/layer.*force_image;

% normalized the force

force = force./(max(max(abs(force))));

% get stepsize dt

dt=0.5;

% get parameters for checking whether to stop

old = phi0;

phi0 = phi0+dt.*force;

new = phi0;

indicator = checkstop(old,new,dt);

% intermediate output

if(mod(n,20) == 0)

showphi(I,phi0,n);

end;

if indicator % decide to stop or continue

showphi(I,phi0,n);

%make mask from SDF

seg = phi0<=0; %-- Get mask from levelset

%subplot(2,2,4); imshow(seg); title('Global Region-Based Segmentation');

return;

end

end;

showphi(I,phi0,n);

%make mask from SDF

seg = phi0<=0; %-- Get mask from levelset

%subplot(2,2,4); imshow(seg); title('Global Region-Based Segmentation');

case 'multiphase'

%-- Initializations

% Get the distance map of the initial masks

mask1 = mask(:,:,1);

mask2 = mask(:,:,2);

phi1=bwdist(mask1)-bwdist(1-mask1)+im2double(mask1)-.5;%Get phi1 from the initial mask 1

phi2=bwdist(mask2)-bwdist(1-mask2)+im2double(mask2)-.5;%Get phi1 from the initial mask 2

%-- Display settings

figure();

%subplot(2,2,1);

if layer ~= 1

%imshow(I); title('Input Image');

else

%imagesc(P); axis image; colormap(gray);title('Input Image');

end

%subplot(2,2,2);

%hold on

%contour(flipud(mask1),[0,0],'r','LineWidth',2.5);

%contour(flipud(mask1),[0,0],'x','LineWidth',1);

%contour(flipud(mask2),[0,0],'g','LineWidth',2.5);

%contour(flipud(mask2),[0,0],'x','LineWidth',1);

%title('initial contour');

%hold off

%subplot(2,2,3); title('Segmentation');

%-- End display settings

%Main loop

for n=1:num_iter

%-- Narrow band for each phase

nb1 = find(phi1<1.2 & phi1>=-1.2); %narrow band of phi1

inidx1 = find(phi1>=0); %phi1 frontground index

outidx1 = find(phi1<0); %phi1 background index

nb2 = find(phi2<1.2 & phi2>=-1.2); %narrow band of phi2

inidx2 = find(phi2>=0); %phi2 frontground index

outidx2 = find(phi2<0); %phi2 background index

%-- End initiliazaions on narrow band

%-- Mean calculations for different partitions

%c11 = mean (phi1>0 & phi2>0)

%c12 = mean (phi1>0 & phi2<0)

%c21 = mean (phi1<0 & phi2>0)

%c22 = mean (phi1<0 & phi2<0)

cc11 = intersect(inidx1,inidx2); %index belong to (phi1>0 & phi2>0)

cc12 = intersect(inidx1,outidx2); %index belong to (phi1>0 & phi2<0)

cc21 = intersect(outidx1,inidx2); %index belong to (phi1<0 & phi2>0)

cc22 = intersect(outidx1,outidx2); %index belong to (phi1<0 & phi2<0)

f_image11 = 0;

f_image12 = 0;

f_image21 = 0;

f_image22 = 0; % initial image force for each layer

for i=1:layer

L = im2double(P(:,:,i)); % get one image component

if isempty(cc11)

c11 = eps;

else

c11 = mean(L(cc11));

end

if isempty(cc12)

c12 = eps;

else

c12 = mean(L(cc12));

end

if isempty(cc21)

c21 = eps;

else

c21 = mean(L(cc21));

end

if isempty(cc22)

c22 = eps;

else

c22 = mean(L(cc22));

end

%-- End mean calculation

%-- Force calculation and normalization

% force on each partition

f_image11=(L-c11).^2.*Heaviside(phi1).*Heaviside(phi2)+f_image11;

f_image12=(L-c12).^2.*Heaviside(phi1).*(1-Heaviside(phi2))+f_image12;

f_image21=(L-c21).^2.*(1-Heaviside(phi1)).*Heaviside(phi2)+f_image21;

f_image22=(L-c22).^2.*(1-Heaviside(phi1)).*(1-Heaviside(phi2))+f_image22;

end

% sum Image Force on all components (used for vector image)

% if 'chan' is applied, this loop become one sigle code as a

% result of layer = 1

% calculate the external force of the image

% curvature on phi1

curvature1 = mu*kappa(phi1);

curvature1 = curvature1(nb1);

% image force on phi1

fim1 = 1/layer.*(-f_image11(nb1)+f_image21(nb1)-f_image12(nb1)+f_image22(nb1));

fim1 = fim1./max(abs(fim1)+eps);

% curvature on phi2

curvature2 = mu*kappa(phi2);

curvature2 = curvature2(nb2);

% image force on phi2

fim2 = 1/layer.*(-f_image11(nb2)+f_image12(nb2)-f_image21(nb2)+f_image22(nb2));

fim2 = fim2./max(abs(fim2)+eps);

% force on phi1 and phi2

force1 = curvature1+fim1;

force2 = curvature2+fim2;

%-- End force calculation

% detal t

dt = 1.5;

old(:,:,1) = phi1;

old(:,:,2) = phi2;

%update of phi1 and phi2

phi1(nb1) = phi1(nb1)+dt.*force1;

phi2(nb2) = phi2(nb2)+dt.*force2;

new(:,:,1) = phi1;

new(:,:,2) = phi2;

indicator = checkstop(old,new,dt);

if indicator

showphi(I, new, n);

%make mask from SDF

seg11 = (phi1>0 & phi2>0); %-- Get mask from levelset

seg12 = (phi1>0 & phi2<0);

seg21 = (phi1<0 & phi2>0);

seg22 = (phi1<0 & phi2<0);

se = strel('disk',1);

aa1 = imerode(seg11,se);

aa2 = imerode(seg12,se);

aa3 = imerode(seg21,se);

aa4 = imerode(seg22,se);

seg = aa1+2*aa2+3*aa3+4*aa4;

%subplot(2,2,4); imagesc(seg);axis image;title('Global Region-Based Segmentation');

return

end

% re-initializations

phi1 = reinitialization(phi1, 0.6);%sussman(phi1, 0.6);%

phi2 = reinitialization(phi2, 0.6);%sussman(phi2,0.6);

%intermediate output

if(mod(n,20) == 0)

phi(:,:,1) = phi1;

phi(:,:,2) = phi2;

showphi(I, phi, n);

end;

end;

phi(:,:,1) = phi1;

phi(:,:,2) = phi2;

showphi(I, phi, n);

%make mask from SDF

seg11 = (phi1>0 & phi2>0); %-- Get mask from levelset

seg12 = (phi1>0 & phi2<0);

seg21 = (phi1<0 & phi2>0);

seg22 = (phi1<0 & phi2<0);

se = strel('disk',1);

aa1 = imerode(seg11,se);

aa2 = imerode(seg12,se);

aa3 = imerode(seg21,se);

aa4 = imerode(seg22,se);

seg = aa1+2*aa2+3*aa3+4*aa4;

%seg = bwlabel(seg);

%subplot(2,2,4); imagesc(seg);axis image;title('Global Region-Based Segmentation');

end

end

function H=Heaviside(z)

% Heaviside step function (smoothed version)

% Copyright (c) 2009,

% Yue Wu @ ECE Department, Tufts University

% All Rights Reserved

Epsilon=10^(-5);

H=zeros(size(z,1),size(z,2));

idx1=find(z>Epsilon);

idx2=find(z<Epsilon & z>-Epsilon);

H(idx1)=1;

for i=1:length(idx2)

H(idx2(i))=1/2*(1+z(idx2(i))/Epsilon+1/pi*sin(pi*z(idx2(i))/Epsilon));

end;

end

%%

function KG = kappa(I)

% get curvature information of input image

% input: 2D image I

% output: curvature matrix KG

% Copyright (c) 2009,

% Yue Wu @ ECE Department, Tufts University

% All Rights Reserved

I = double(I);

[m,n] = size(I);

P = padarray(I,[1,1],1,'pre');

P = padarray(P,[1,1],1,'post');

% central difference

fy = P(3:end,2:n+1)-P(1:m,2:n+1);

fx = P(2:m+1,3:end)-P(2:m+1,1:n);

fyy = P(3:end,2:n+1)+P(1:m,2:n+1)-2*I;

fxx = P(2:m+1,3:end)+P(2:m+1,1:n)-2*I;

fxy = 0.25.*(P(3:end,3:end)-P(1:m,3:end)+P(3:end,1:n)-P(1:m,1:n));

G = (fx.^2+fy.^2).^(0.5);

K = (fxx.*fy.^2-2*fxy.*fx.*fy+fyy.*fx.^2)./((fx.^2+fy.^2+eps).^(1.5));

KG = K.*G;

KG(1,:) = eps;

KG(end,:) = eps;

KG(:,1) = eps;

KG(:,end) = eps;

KG = KG./max(max(abs(KG)));

end

%%

function m = maskcircle2(I,type)

% auto pick a circular mask for image I

% built-in mask creation function

% Input: I : input image

% type: mask shape keywords

% Output: m : mask image

% Copyright (c) 2009,

% Yue Wu @ ECE Department, Tufts University

% All Rights Reserved

if size(I,3)~=3

temp = double(I(:,:,1));

else

temp = double(rgb2gray(I));

end

h = [0 1 0; 1 -4 1; 0 1 0];

T = conv2(temp,h);

T(1,:) = 0;

T(end,:) = 0;

T(:,1) = 0;

T(:,end) = 0;

thre = max(max(abs(T)))*.5;

idx = find(abs(T) > thre);

[cx,cy] = ind2sub(size(T),idx);

cx = round(mean(cx));

cy = round(mean(cy));

[x,y] = meshgrid(1:min(size(temp,1),size(temp,2)));

m = zeros(size(temp));

[p,q] = size(temp);

switch lower (type)

case 'small'

r = 10;

n = zeros(size(x));

n((x-cx).^2+(y-cy).^2<r.^2) = 1;

m(1:size(n,1),1:size(n,2)) = n;

%m((x-cx).^2+(y-cy).^2<r.^2) = 1;

case 'medium'

r = min(min(cx,p-cx),min(cy,q-cy));

r = max(2/3*r,25);

n = zeros(size(x));

n((x-cx).^2+(y-cy).^2<r.^2) = 1;

m(1:size(n,1),1:size(n,2)) = n;

%m((x-cx).^2+(y-cy).^2<r.^2) = 1;

case 'large'

r = min(min(cx,p-cx),min(cy,q-cy));

r = max(2/3*r,60);

n = zeros(size(x));

n((x-cx).^2+(y-cy).^2<r.^2) = 1;

m(1:size(n,1),1:size(n,2)) = n;

%m((x-cx).^2+(y-cy).^2<r.^2) = 1;

case 'whole'

r = 9;

m = zeros(round(ceil(max(p,q)/2/(r+1))*3*(r+1)));

siz = size(m,1);

sx = round(siz/2);

i = 1:round(siz/2/(r+1));

j = 1:round(0.9*siz/2/(r+1));

j = j-round(median(j));

m(sx+2*j*(r+1),(2*i-1)*(r+1)) = 1;

se = strel('disk',r);

m = imdilate(m,se);

m = m(round(siz/2-p/2-6):round(siz/2-p/2-6)+p-1,round(siz/2-q/2-6):round(siz/2-q/2-6)+q-1);

end

tem(:,:,1) = m;

M = padarray(m,[floor(2/3*r),floor(2/3*r)],0,'post');

tem(:,:,2) = M(floor(2/3*r)+1:end,floor(2/3*r)+1:end);

m = tem;

return

end

%%

function D = reinitialization(D,dt)

% reinitialize the distance map for active contour

% Copyright (c) 2009,

% Yue Wu @ ECE Department, Tufts University

% All Rights Reserved

T = padarray(D,[1,1],0,'post');

T = padarray(T,[1,1],0,'pre');

% differences on all directions

a = D-T(1:end-2,2:end-1);

b = T(3:end,2:end-1)-D;

c = D-T(2:end-1,1:end-2);

d = T(2:end-1,3:end)-D;

a_p = max(a,0);

a_m = min(a,0);

b_p = max(b,0);

b_m = min(b,0);

c_p = max(c,0);

c_m = min(c,0);

d_p = max(d,0);

d_m = min(d,0);

G = zeros(size(D));

ind_plus = find(D>0);

ind_minus = find(D<0);

G(ind_plus) = sqrt(max(a_p(ind_plus).^2,b_m(ind_plus).^2)+max(c_p(ind_plus).^2,d_m(ind_plus).^2))-1;

G(ind_minus) = sqrt(max(a_m(ind_minus).^2,b_p(ind_minus).^2)+max(c_m(ind_minus).^2,d_p(ind_minus).^2))-1;

sign_D = D./sqrt(D.^2+1);

D = D-dt.*sign_D.*G;

end

%%

function showphi(I, phi, i)

% show curve evolution of phi

% Copyright (c) 2009,

% Yue Wu @ ECE Department, Tufts University

% All Rights Reserved

for j = 1:size(phi,3)

phi_{j} = phi(:,:,j);

end

%imshow(I,'initialmagnification','fit','displayrange',[0 255]);

%hold on;

if size(phi,3) == 1

%contour(phi_{1}, [0 0], 'r','LineWidth',4);

%contour(phi_{1}, [0 0], 'g','LineWidth',1.3);

else

%contour(phi_{1}, [0 0], 'r','LineWidth',4);

%contour(phi_{1}, [0 0], 'x','LineWidth',1.3);

%contour(phi_{2}, [0 0], 'g','LineWidth',4);

%contour(phi_{2}, [0 0], 'x','LineWidth',1.3);

end

%hold off;

%title([num2str(i) ' Iterations']);

%drawnow;

end

%%

function indicator = checkstop(old,new,dt)

% indicate whether we should performance further iteraions or stop

% Copyright (c) 2009,

% Yue Wu @ ECE Department, Tufts University

% All Rights Reserved

layer = size(new,3);

for i = 1:layer

old_{i} = old(:,:,i);

new_{i} = new(:,:,i);

end

if layer

ind = find(abs(new)<=.5);

M = length(ind);

Q = sum(abs(new(ind)-old(ind)))./M;

if Q<=dt*.18^2

indicator = 1;

else

indicator = 0;

end

else

ind1 = find(abs(old_{1})<1);

ind2 = find(abs(old_{2})<1);

M1 = length(ind1);

M2 = length(ind2);

Q1 = sum(abs(new_{1}(ind1)-old_{1}(ind1)))./M1;

Q2 = sum(abs(new_{2}(ind2)-old_{2}(ind2)))./M2;

if Q1<=dt*.18^2 && Q2<=dt*.18^2

indicator = 1;

else

indicator = 0;

end

end

return

end

%%

%%%%%%%%%%%%%%%%%%%%%%%%%%%%%%%%%%%%%%%%%%%%%%%%

| **M-file name** | clinic_heart_disease_system |
| --- | --- |
| **Type** | GUI (m file) |
| **Description** | The main GUI of our project. It represents the platform of experimenter to evaluate the coded algorithms |
| **comments** | Novel. Written by us. |

**Code:**

function varargout = clinic_heart_disease_system(varargin)

% CLINIC_HEART_DISEASE_SYSTEM MATLAB code for clinic_heart_disease_system.fig

% CLINIC_HEART_DISEASE_SYSTEM, by itself, creates a new CLINIC_HEART_DISEASE_SYSTEM or raises the existing

% singleton*.

%

% H = CLINIC_HEART_DISEASE_SYSTEM returns the handle to a new CLINIC_HEART_DISEASE_SYSTEM or the handle to

% the existing singleton*.

%

% CLINIC_HEART_DISEASE_SYSTEM('CALLBACK',hObject,eventData,handles,...) calls the local

% function named CALLBACK in CLINIC_HEART_DISEASE_SYSTEM.M with the given input arguments.

%

% CLINIC_HEART_DISEASE_SYSTEM('Property','Value',...) creates a new CLINIC_HEART_DISEASE_SYSTEM or raises the

% existing singleton*. Starting from the left, property value pairs are

% applied to the GUI before clinic_heart_disease_system_OpeningFcn gets called. An

% unrecognized property name or invalid value makes property application

% stop. All inputs are passed to clinic_heart_disease_system_OpeningFcn via varargin.

%

% *See GUI Options on GUIDE's Tools menu. Choose "GUI allows only one

% instance to run (singleton)".

%

% See also: GUIDE, GUIDATA, GUIHANDLES

% Edit the above text to modify the response to help clinic_heart_d isease_system

% Last Modified by GUIDE v2.5 13-Feb-2021 22:25:17

% Begin initialization code - DO NOT EDIT

gui_Singleton = 1;

gui_State = struct('gui_Name', mfilename, ...

'gui_Singleton', gui_Singleton, ...

'gui_OpeningFcn', @clinic_heart_disease_system_OpeningFcn, ...

'gui_OutputFcn', @clinic_heart_disease_system_OutputFcn, ...

'gui_LayoutFcn', [] , ...

'gui_Callback', []);

if nargin && ischar(varargin{1})

gui_State.gui_Callback = str2func(varargin{1});

end

if nargout

[varargout{1:nargout}] = gui_mainfcn(gui_State, varargin{:});

else

gui_mainfcn(gui_State, varargin{:});

end

% End initialization code - DO NOT EDIT

% --- Executes just before clinic_heart_disease_system is made visible.

function clinic_heart_disease_system_OpeningFcn(hObject, eventdata, handles, varargin)

% This function has no output args, see OutputFcn.

% hObject handle to figure

% eventdata reserved - to be defined in a future version of MATLAB

% handles structure with handles and user data (see GUIDATA)

% varargin command line arguments to clinic_heart_disease_system (see VARARGIN)

% Choose default command line output for clinic_heart_disease_system

handles.output = hObject;

I=imread('logo.jpg');

axes(handles.axes7);

imagesc(I);

%numofIter=[13 12 11 10 9 9 9 9 10 10 10 11 11 12 13 13]; % you can change num of iteration of each ROI of the 16 frames

numofIter=[13 10]; % you can change num of iteration of each ROI of the 16 frames

numofIterRG=[550 350]; % for region growing X algorithm

XD=0; YD=0; % change XD for the horizontal displacement and YD for the vertical displacement

SmoothFactor=0.2; ContractionBias=0;

method='Chan-Vese';

%method='edge';

save AC SmoothFactor ContractionBias method

save maskallparam numofIter numofIterRG XD YD

% Update handles structure

guidata(hObject, handles);

% UIWAIT makes clinic_heart_disease_system wait for user response (see UIRESUME)

% uiwait(handles.figure1);

% --- Outputs from this function are returned to the command line.

function varargout = clinic_heart_disease_system_OutputFcn(hObject, eventdata, handles)

% varargout cell array for returning output args (see VARARGOUT);

% hObject handle to figure

% eventdata reserved - to be defined in a future version of MATLAB

% handles structure with handles and user data (see GUIDATA)

% Get default command line output from handles structure

varargout{1} = handles.output;

% --- Executes on button press in pushbutton1.

function pushbutton1_Callback(hObject, eventdata, handles)

%% Post Part %%

load info info;

tag11=1;

save prepost1 tag11

%get mask for the roi

%% Background Subtraction Post

for i=1:info.NumberOfFrames

mean_val(i)=mean(mean(handles.imvol(:,:,i)));

end

% mx_val=max(max(mean_val));

% ind_max=find(mx_val==mean_val);

% maxImg=handles.imvol(:,:,ind_max);

% handles.imvolmx=mean(handles.imvol,3);

%

%axes(handles.axes1)

%% max - min of ROI Post

[handles.All_masks1,handles.smoothedPoly11, handles.smoothedPoly12,handles.max_img1,handles.min_img1,handles.imvolmx1,handles.imvolmn1,handles.bw, handles.bwMin1, handles.area1Post, handles.area2Post, handles.ratePost,handles.minLongDIVmaxLPost, handles.minShortDIVmaxLPost,handles.AverageDivPost, handles.circularityMaxPost, handles.circularityMinPost,handles.ElongationMaxPost,handles.ElongationMinPost,sp_post]=max_min_MUGA(handles.imvol,0);

save sp_post sp_post;

% background

%mb=msgbox("Please draw the background region","Draw BKG")

%waitfor(mb);

axes(handles.axes2);axis tight;

imagesc(sum(handles.imvol,3)); colormap(jet),colorbar ;

NewImg1=imdilate(handles.bw,strel('disk',8))-imdilate(handles.bw,strel('disk',6));

NewImg2=imdilate(handles.bw,strel('disk',7))-imdilate(handles.bw,strel('disk',5));

NewImg3=imdilate(handles.bw,strel('disk',6))-imdilate(handles.bw,strel('disk',4));

NewImg4=imdilate(handles.bw,strel('disk',5))-imdilate(handles.bw,strel('disk',3));

NewImg5=imdilate(handles.bw,strel('disk',4))-imdilate(handles.bw,strel('disk',2));

S1=regionprops(bwlabel(NewImg1),'Centroid');

S2=regionprops(bwlabel(NewImg2),'Centroid');

S3=regionprops(bwlabel(NewImg3),'Centroid');

S4=regionprops(bwlabel(NewImg4),'Centroid');

S5=regionprops(bwlabel(NewImg5),'Centroid');

cc1=round([S1.Centroid]);

cc2=round([S2.Centroid]);

cc3=round([S3.Centroid]);

cc4=round([S4.Centroid]);

cc5=round([S5.Centroid]);

D1=sum(sum(handles.imvolmn1(cc1(2):end,cc1(1):end)));

D2=sum(sum(handles.imvolmn1(cc2(2):end,cc2(1):end)));

D3=sum(sum(handles.imvolmn1(cc3(2):end,cc3(1):end)));

D4=sum(sum(handles.imvolmn1(cc4(2):end,cc4(1):end)));

D5=sum(sum(handles.imvolmn1(cc5(2):end,cc5(1):end)));

NewImg1(1:cc1(2),1:cc1(1))=0;

NewImg1(1:cc1(2),cc1(1):end)=0;

NewImg1(cc1(2):end,1:cc1(1))=0;

NewImg2(1:cc2(2),1:cc2(1))=0;

NewImg2(1:cc2(2),cc2(1):end)=0;

NewImg2(cc2(2):end,1:cc2(1))=0;

NewImg3(1:cc3(2),1:cc3(1))=0;

NewImg3(1:cc3(2),cc3(1):end)=0;

NewImg3(cc3(2):end,1:cc3(1))=0;

NewImg4(1:cc4(2),1:cc4(1))=0;

NewImg4(1:cc4(2),cc4(1):end)=0;

NewImg4(cc4(2):end,1:cc4(1))=0;

NewImg5(1:cc5(2),1:cc5(1))=0;

NewImg5(1:cc5(2),cc5(1):end)=0;

NewImg5(cc5(2):end,1:cc5(1))=0;

% NewImg1(cc1(2)+15:end,:)=0;

% NewImg1(:,1:cc1(1)+5)=0;

% NewImg2(cc2(2)+15:end,:)=0;

% NewImg2(:,1:cc2(1)+5)=0;

% NewImg3(cc3(2)+15:end,:)=0;

% NewImg3(:,1:cc3(1)+5)=0;

% NewImg4(cc4(2)+15:end,:)=0;

% NewImg4(:,1:cc4(1)+5)=0;

% NewImg5(cc5(2)+15:end,:)=0;

% NewImg5(:,1:cc5(1)+5)=0;

NewImg6=NewImg1|NewImg2|NewImg3|NewImg4|NewImg5;

handles.imvolmn11=handles.min_img1;

handles.imvolmn11(NewImg6==0)=0;

handles.meanBG1=mean(mean(handles.min_img1(NewImg1==1)));

handles.meanBG2=mean(mean(handles.min_img1(NewImg2==1)));

handles.meanBG3=mean(mean(handles.min_img1(NewImg3==1)));

handles.meanBG4=mean(mean(handles.min_img1(NewImg4==1)));

handles.meanBG5=mean(mean(handles.min_img1(NewImg5==1)));

%handles.meanBGMn2=(handles.meanBG1+handles.meanBG2+handles.meanBG3+handles.meanBG4+handles.meanBG5)/5

handles.meanBGMn2=min([handles.meanBG1,handles.meanBG2,handles.meanBG3,handles.meanBG4,handles.meanBG5]);

mtx=[handles.meanBG1,handles.meanBG2,handles.meanBG3,handles.meanBG4,handles.meanBG5];

indN=find(mtx==min(mtx));

indX=indN(1);

%meanBkgCount=mtx(indX)

if(indX==1)PixelsBkgMin=sum(sum(NewImg1));

elseif(indX==2)PixelsBkgMin=sum(sum(NewImg2));NewImg1=NewImg2;

elseif(indX==3)PixelsBkgMin=sum(sum(NewImg3));NewImg1=NewImg3;

elseif(indX==4)PixelsBkgMin=sum(sum(NewImg4));NewImg1=NewImg4;

else PixelsBkgMin=sum(sum(NewImg5));NewImg1=NewImg5;

end

meanBGMn2=handles.meanBGMn2;

save meanBGMn2 meanBGMn2

handles.PixelsBkgMinPost=PixelsBkgMin;

%h1 = imfreehand;

BG=NewImg1;

%handles.meanBG=mean(mean(mean(handles.imvol(NewImg1==1))));

ee=edge(NewImg1,'canny');

ee=NewImg1;

[xE yE]=find(ee==1);

waitfor(xE);

hold on

aaa=plot(yE,xE,'r');

hold off;

%%

NewImg1=imdilate(handles.bwMin1,strel('disk',8))-imdilate(handles.bwMin1,strel('disk',6));

NewImg2=imdilate(handles.bwMin1,strel('disk',7))-imdilate(handles.bwMin1,strel('disk',5));

NewImg3=imdilate(handles.bwMin1,strel('disk',6))-imdilate(handles.bwMin1,strel('disk',4));

NewImg4=imdilate(handles.bwMin1,strel('disk',5))-imdilate(handles.bwMin1,strel('disk',3));

NewImg5=imdilate(handles.bwMin1,strel('disk',4))-imdilate(handles.bwMin1,strel('disk',2));

S1=regionprops(bwlabel(NewImg1),'Centroid');

S2=regionprops(bwlabel(NewImg2),'Centroid');

S3=regionprops(bwlabel(NewImg3),'Centroid');

S4=regionprops(bwlabel(NewImg4),'Centroid');

S5=regionprops(bwlabel(NewImg5),'Centroid');

cc1=round([S1.Centroid]);

cc2=round([S2.Centroid]);

cc3=round([S3.Centroid]);

cc4=round([S4.Centroid]);

cc5=round([S5.Centroid]);

% A=sum(sum(handles.imvolmn1(1:cc(2),1:cc(1))));

% B=sum(sum(handles.imvolmn1(1:cc(2),cc(1):end)));

% C=sum(sum(handles.imvolmn1(cc(2):end,1:cc(1))));

D1=sum(sum(handles.imvolmn1(cc1(2):end,cc1(1):end)));

D2=sum(sum(handles.imvolmn1(cc2(2):end,cc2(1):end)));

D3=sum(sum(handles.imvolmn1(cc3(2):end,cc3(1):end)));

D4=sum(sum(handles.imvolmn1(cc4(2):end,cc4(1):end)));

D5=sum(sum(handles.imvolmn1(cc5(2):end,cc5(1):end)));

NewImg1(1:cc1(2),1:cc1(1))=0;

NewImg1(1:cc1(2),cc1(1):end)=0;

NewImg1(cc1(2):end,1:cc1(1))=0;

NewImg2(1:cc2(2),1:cc2(1))=0;

NewImg2(1:cc2(2),cc2(1):end)=0;

NewImg2(cc2(2):end,1:cc2(1))=0;

NewImg3(1:cc3(2),1:cc3(1))=0;

NewImg3(1:cc3(2),cc3(1):end)=0;

NewImg3(cc3(2):end,1:cc3(1))=0;

NewImg4(1:cc4(2),1:cc4(1))=0;

NewImg4(1:cc4(2),cc4(1):end)=0;

NewImg4(cc4(2):end,1:cc4(1))=0;

NewImg5(1:cc5(2),1:cc5(1))=0;

NewImg5(1:cc5(2),cc5(1):end)=0;

NewImg5(cc5(2):end,1:cc5(1))=0;

% NewImg1(cc1(2)+15:end,:)=0;

% NewImg1(:,1:cc1(1)+5)=0;

% NewImg2(cc2(2)+15:end,:)=0;

% NewImg2(:,1:cc2(1)+5)=0;

% NewImg3(cc3(2)+15:end,:)=0;

% NewImg3(:,1:cc3(1)+5)=0;

% NewImg4(cc4(2)+15:end,:)=0;

% NewImg4(:,1:cc4(1)+5)=0;

% NewImg5(cc5(2)+15:end,:)=0;

% NewImg5(:,1:cc5(1)+5)=0;

NewImg6=NewImg1|NewImg2|NewImg3|NewImg4|NewImg5;

handles.imvolmn11=handles.min_img1;

handles.imvolmn11(NewImg6==0)=0;

handles.meanBG1=mean(mean(handles.max_img1(NewImg1==1)));

handles.meanBG2=mean(mean(handles.max_img1(NewImg2==1)));

handles.meanBG3=mean(mean(handles.max_img1(NewImg3==1)));

handles.meanBG4=mean(mean(handles.max_img1(NewImg4==1)));

handles.meanBG5=mean(mean(handles.max_img1(NewImg5==1)));

%handles.meanBGMx2=(handles.meanBG1+handles.meanBG2+handles.meanBG3+handles.meanBG4+handles.meanBG5)/5

handles.meanBGMx2=min([handles.meanBG1,handles.meanBG2,handles.meanBG3,handles.meanBG4,handles.meanBG5]);

mtx=[handles.meanBG1,handles.meanBG2,handles.meanBG3,handles.meanBG4,handles.meanBG5];

indN=find(mtx==min(mtx));

indX=indN(1);

%meanBkgMaxCount=mtx(indX)

if(indX==1)PixelsBkgMax=sum(sum(NewImg1));

elseif(indX==2)PixelsBkgMax=sum(sum(NewImg2));

elseif(indX==3)PixelsBkgMax=sum(sum(NewImg3));

elseif(indX==4)PixelsBkgMax=sum(sum(NewImg4));

else PixelsBkgMax=sum(sum(NewImg5));

end

handles.PixelsBkgMaxPost=PixelsBkgMax;

%%

%handles.meanBG=max([handles.meanBG1+handles.meanBG2+handles.meanBG3+handles.meanBG4+handles.meanBG5]);

handles.imvolmn11=handles.min_img1;

handles.imvolmn11(NewImg1==0)=0;

%waitfor(aaa);

%

%handles.max_img1=handles.max_img1-handles.meanBGMx;

PixelNumMaxPost=sum(sum(handles.bw));

%handles.min_img1=handles.min_img1-handles.meanBGMn;

PixelNumMinPost=sum(sum(handles.bwMin1));

TotalBGmaxPost=sum(sum(sum(handles.max_img1(handles.bw==1))));

TotalBGminPost=sum(sum(sum(handles.min_img1(handles.bwMin1==1))));

waitfor(handles.bw);

handles.bw =uint16(handles.bw);

% creat mask

handles.max_mask=sum(handles.All_masks1,3);

handles.max_mask=uint16(im2bw(handles.max_mask,0.1));

for i=1:16

handles.imvol1(:,:,i) = (handles.max_mask.*handles.imvol(:,:,i));

end

% calculate mean count/pixel

handles.mnzs = mean(nonzeros(handles.imvol1));

% calculate lung uptake

mb=msgbox('Select Manually Lung ROI (Post)','select ROI') ;

waitfor(mb);

axes(handles.axes3);

imagesc(sum(handles.imvol,3));colormap(jet),colorbar;

% draw ROI

hh = imrect;

handles.bw11=createMask(hh);

handles.bw11 =uint16(handles.bw11);

%close Figure 4

%close Figure 3

%close Figure 31

% creat mask

for i=1:16

handles.imvol11(:,:,i) = (handles.bw11.*handles.imvol(:,:,i));

end

handles.mnzs11 = mean(nonzeros(handles.imvol11));

handles.heart2lungpost = handles.mnzs11/handles.mnzs;

%estimates the area of the objects in binary image handles.bw and handles.bw1.

handles.A1 =bwarea(handles.bw);

handles.A2 =bwarea(handles.bw1);

% Max area ratio

handles.TIDmax = handles.A1/handles.A2;

% Min area ratio

handles.A1 =bwarea(handles.bwMin1);

handles.A2 =bwarea(handles.bwMin2);

handles.TIDmin = handles.A1/handles.A2;

mb=msgbox('post part is done, show results now');

waitfor(mb);

guidata(hObject, handles);

% hObject handle to pushbutton1 (see GCBO)

% eventdata reserved - to be defined in a future version of MATLAB

% handles structure with handles and user data (see GUIDATA)

% --- Executes on button press in pushbutton2.

function pushbutton2_Callback(hObject, eventdata, handles)

%% Pre Part %%

%read images (pre-chemo)

load info2 info2;

tag11=0;

save prepost1 tag11

%butterworth smoothing

% view

%figure (2), imshow(sum(handles.imvol2,3),[]);

% draw ROI

%% Background Subtraction Pre

for i=1:info2.NumberOfFrames

mean_val(i)=mean(mean(handles.imvol2(:,:,i)));

end

%mx_val=max(max(mean_val));

%ind_max=find(mx_val==mean_val);

%maxImg=handles.imvol2(:,:,ind_max);

%handles.imvolmx=mean(handles.imvol2,3);

%mb=msgbox("Please draw the background region","draw bkg");

%waitfor(mb);

%axes(handles.axes4);

%imagesc(handles.imvolmx); colormap(jet),colorbar ;

% h1 = imfreehand;

% BG=createMask(h1);

% handles.meanBG=mean(mean(mean(handles.imvolmx(BG==1))));

% waitfor(BG);

%close;

clear BG h1 handles.imvolmx

%% max - min of ROI Pre

% axes(handles.axes4)

[handles.All_masks2,handles.smoothedPoly21, handles.smoothedPoly22, handles.max_img2,handles.min_img2,handles.imvolmx2,handles.imvolmn2,handles.bw1, handles.bwMin2, handles.area1Pre, handles.area2Pre, handles.ratePre,handles.minLongDIVmaxLPre, handles.minShortDIVmaxLPre,handles.AverageDivPre, handles.circularityMaxPre, handles.circularityMinPre,handles.ElongationMaxPre,handles.ElongationMinPre,sp_pre]=max_min_MUGA(handles.imvol2,0);

save sp_pre sp_pre;

% handles.meanBGmax=mean(mean(mean(handles.max_img2(handles.bw1==1))));

% handles.meanBGmin=mean(mean(mean(handles.min_img2(handles.bwMin2==1))));

axes(handles.axes5);axis tight;

imagesc(sum(handles.imvol2,3)); colormap(jet),colorbar ;

NewImg1=imdilate(handles.bw1,strel('disk',8))-imdilate(handles.bw1,strel('disk',6));

NewImg2=imdilate(handles.bw1,strel('disk',7))-imdilate(handles.bw1,strel('disk',5));

NewImg3=imdilate(handles.bw1,strel('disk',6))-imdilate(handles.bw1,strel('disk',4));

NewImg4=imdilate(handles.bw1,strel('disk',5))-imdilate(handles.bw1,strel('disk',3));

NewImg5=imdilate(handles.bw1,strel('disk',4))-imdilate(handles.bw1,strel('disk',2));

S1=regionprops(bwlabel(NewImg1),'Centroid');

S2=regionprops(bwlabel(NewImg2),'Centroid');

S3=regionprops(bwlabel(NewImg3),'Centroid');

S4=regionprops(bwlabel(NewImg4),'Centroid');

S5=regionprops(bwlabel(NewImg5),'Centroid');

cc1=round([S1.Centroid]);

cc2=round([S2.Centroid]);

cc3=round([S3.Centroid]);

cc4=round([S4.Centroid]);

cc5=round([S5.Centroid]);

D1=sum(sum(handles.imvolmn2(cc1(2):end,cc1(1):end)));

D2=sum(sum(handles.imvolmn2(cc2(2):end,cc2(1):end)));

D3=sum(sum(handles.imvolmn2(cc3(2):end,cc3(1):end)));

D4=sum(sum(handles.imvolmn2(cc4(2):end,cc4(1):end)));

D5=sum(sum(handles.imvolmn2(cc5(2):end,cc5(1):end)));

NewImg1(1:cc1(2),1:cc1(1))=0;

NewImg1(1:cc1(2),cc1(1):end)=0;

NewImg1(cc1(2):end,1:cc1(1))=0;

NewImg2(1:cc2(2),1:cc2(1))=0;

NewImg2(1:cc2(2),cc2(1):end)=0;

NewImg2(cc2(2):end,1:cc2(1))=0;

NewImg3(1:cc3(2),1:cc3(1))=0;

NewImg3(1:cc3(2),cc3(1):end)=0;

NewImg3(cc3(2):end,1:cc3(1))=0;

NewImg4(1:cc4(2),1:cc4(1))=0;

NewImg4(1:cc4(2),cc4(1):end)=0;

NewImg4(cc4(2):end,1:cc4(1))=0;

NewImg5(1:cc5(2),1:cc5(1))=0;

NewImg5(1:cc5(2),cc5(1):end)=0;

NewImg5(cc5(2):end,1:cc5(1))=0;

%NewImg1(cc1(2)+15:end,:)=0;

%NewImg1(:,1:cc1(1)+5)=0;

%NewImg2(cc2(2)+15:end,:)=0;

%NewImg2(:,1:cc2(1)+5)=0;

%NewImg3(cc3(2)+15:end,:)=0;

%NewImg3(:,1:cc3(1)+5)=0;

%NewImg4(cc4(2)+15:end,:)=0;

%NewImg4(:,1:cc4(1)+5)=0;

%NewImg5(cc5(2)+15:end,:)=0;

%NewImg5(:,1:cc5(1)+5)=0;

NewImg6=NewImg1|NewImg2|NewImg3|NewImg4|NewImg5;

% Vec=[D1 D2 D3 D4 D5];

% x=find(Vec==min(Vec));

% if(x(1)==1)

% NewImg1(1:cc1(2),1:cc1(1))=0;

% NewImg1(1:cc1(2),cc1(1):end)=0;

% NewImg1(cc1(2):end,1:cc1(1))=0;

% elseif(x(1)==2)

% NewImg1=NewImg2;

% NewImg1(1:cc2(2),1:cc2(1))=0;

% NewImg1(1:cc2(2),cc2(1):end)=0;

% NewImg1(cc2(2):end,1:cc2(1))=0;

% elseif (x(1)==3)

% NewImg1=NewImg3;

% NewImg1(1:cc3(2),1:cc3(1))=0;

% NewImg1(1:cc3(2),cc3(1):end)=0;

% NewImg1(cc3(2):end,1:cc3(1))=0;

% elseif (x(1)==4)

% NewImg1=NewImg4;

% NewImg1(1:cc4(2),1:cc4(1))=0;

% NewImg1(1:cc4(2),cc4(1):end)=0;

% NewImg1(cc4(2):end,1:cc4(1))=0;

% else

% NewImg1=NewImg5;

% NewImg1(1:cc5(2),1:cc5(1))=0;

% NewImg1(1:cc5(2),cc5(1):end)=0;

% NewImg1(cc5(2):end,1:cc5(1))=0;

% end

handles.imvolmn11=handles.min_img2;

handles.imvolmn11(NewImg6==0)=0;

handles.meanBG1=mean(mean(handles.min_img2(NewImg1==1)));

handles.meanBG2=mean(mean(handles.min_img2(NewImg2==1)));

handles.meanBG3=mean(mean(handles.min_img2(NewImg3==1)));

handles.meanBG4=mean(mean(handles.min_img2(NewImg4==1)));

handles.meanBG5=mean(mean(handles.min_img2(NewImg5==1)));

%handles.meanBGMn=(handles.meanBG1+handles.meanBG2+handles.meanBG3+handles.meanBG4+handles.meanBG5)/5

handles.meanBGMn=min([handles.meanBG1,handles.meanBG2,handles.meanBG3,handles.meanBG4,handles.meanBG5]);

meanBGMn=handles.meanBGMn;

save meanBGMn meanBGMn

handles.imvolmn11=handles.min_img2;

handles.imvolmn11(NewImg1==0)=0;

mtx=[handles.meanBG1,handles.meanBG2,handles.meanBG3,handles.meanBG4,handles.meanBG5];

indN=find(mtx==min(mtx));

indX=indN(1);

%meanBkgMaxCount=mtx(indX)

if(indX==1)PixelsBkgMin=sum(sum(NewImg1));

elseif(indX==2)PixelsBkgMin=sum(sum(NewImg2));NewImg1=NewImg2;

elseif(indX==3)PixelsBkgMin=sum(sum(NewImg3));NewImg1=NewImg3;

elseif(indX==4)PixelsBkgMin=sum(sum(NewImg4));NewImg1=NewImg4;

else PixelsBkgMin=sum(sum(NewImg5));NewImg1=NewImg5;

end

handles.PixelsBkgMinPre=PixelsBkgMin;

%h1 = imfreehand;

%handles.meanBG=mean(mean(mean(handles.imvol2(NewImg1==1))))

ee=edge(NewImg1,'canny');

ee=NewImg1;

[xE yE]=find(ee==1);

waitfor(xE);

hold on

aaa=plot(yE,xE,'r');

hold off;

%%

NewImg1=imdilate(handles.bwMin2,strel('disk',8))-imdilate(handles.bwMin2,strel('disk',6));

NewImg2=imdilate(handles.bwMin2,strel('disk',7))-imdilate(handles.bwMin2,strel('disk',5));

NewImg3=imdilate(handles.bwMin2,strel('disk',6))-imdilate(handles.bwMin2,strel('disk',4));

NewImg4=imdilate(handles.bwMin2,strel('disk',5))-imdilate(handles.bwMin2,strel('disk',3));

NewImg5=imdilate(handles.bwMin2,strel('disk',4))-imdilate(handles.bwMin2,strel('disk',2));

S1=regionprops(bwlabel(NewImg1),'Centroid');

S2=regionprops(bwlabel(NewImg2),'Centroid');

S3=regionprops(bwlabel(NewImg3),'Centroid');

S4=regionprops(bwlabel(NewImg4),'Centroid');

S5=regionprops(bwlabel(NewImg5),'Centroid');

cc1=round([S1.Centroid]);

cc2=round([S2.Centroid]);

cc3=round([S3.Centroid]);

cc4=round([S4.Centroid]);

cc5=round([S5.Centroid]);

D1=sum(sum(handles.imvolmn2(cc1(2):end,cc1(1):end)));

D2=sum(sum(handles.imvolmn2(cc2(2):end,cc2(1):end)));

D3=sum(sum(handles.imvolmn2(cc3(2):end,cc3(1):end)));

D4=sum(sum(handles.imvolmn2(cc4(2):end,cc4(1):end)));

D5=sum(sum(handles.imvolmn2(cc5(2):end,cc5(1):end)));

NewImg1(1:cc1(2),1:cc1(1))=0;

NewImg1(1:cc1(2),cc1(1):end)=0;

NewImg1(cc1(2):end,1:cc1(1))=0;

NewImg2(1:cc2(2),1:cc2(1))=0;

NewImg2(1:cc2(2),cc2(1):end)=0;

NewImg2(cc2(2):end,1:cc2(1))=0;

NewImg3(1:cc3(2),1:cc3(1))=0;

NewImg3(1:cc3(2),cc3(1):end)=0;

NewImg3(cc3(2):end,1:cc3(1))=0;

NewImg4(1:cc4(2),1:cc4(1))=0;

NewImg4(1:cc4(2),cc4(1):end)=0;

NewImg4(cc4(2):end,1:cc4(1))=0;

NewImg5(1:cc5(2),1:cc5(1))=0;

NewImg5(1:cc5(2),cc5(1):end)=0;

NewImg5(cc5(2):end,1:cc5(1))=0;

%NewImg1(cc1(2)+15:end,:)=0;

%NewImg1(:,1:cc1(1)+5)=0;

%NewImg2(cc2(2)+15:end,:)=0;

%NewImg2(:,1:cc2(1)+5)=0;

%NewImg3(cc3(2)+15:end,:)=0;

%NewImg3(:,1:cc3(1)+5)=0;

%NewImg4(cc4(2)+15:end,:)=0;

%NewImg4(:,1:cc4(1)+5)=0;

%NewImg5(cc5(2)+15:end,:)=0;

%NewImg5(:,1:cc5(1)+5)=0;

NewImg6=NewImg1|NewImg2|NewImg3|NewImg4|NewImg5;

handles.imvolmn11=handles.min_img2;

handles.imvolmn11(NewImg6==0)=0;

handles.meanBG1=mean(mean(handles.max_img2(NewImg1==1)));

handles.meanBG2=mean(mean(handles.max_img2(NewImg2==1)));

handles.meanBG3=mean(mean(handles.max_img2(NewImg3==1)));

handles.meanBG4=mean(mean(handles.max_img2(NewImg4==1)));

handles.meanBG5=mean(mean(handles.max_img2(NewImg5==1)));

%handles.meanBGMx=(handles.meanBG1+handles.meanBG2+handles.meanBG3+handles.meanBG4+handles.meanBG5)/5

handles.meanBGMx=min([handles.meanBG1,handles.meanBG2,handles.meanBG3,handles.meanBG4,handles.meanBG5]);

mtx=[handles.meanBG1,handles.meanBG2,handles.meanBG3,handles.meanBG4,handles.meanBG5];

indN=find(mtx==min(mtx));

indX=indN(1);

%meanBkgMaxCount=mtx(indX)

if(indX==1)PixelsBkgMax=sum(sum(NewImg1));

elseif(indX==2)PixelsBkgMax=sum(sum(NewImg2));

elseif(indX==3)PixelsBkgMax=sum(sum(NewImg3));

elseif(indX==4)PixelsBkgMax=sum(sum(NewImg4));

else PixelsBkgMax=sum(sum(NewImg5));

end

handles.PixelsBkgMaxPre=PixelsBkgMax;

%NewImg1(cc1(2)+15:end,:)=0;

%handles.max_img2=handles.max_img2-handles.meanBGMx;

PixelNumMaxPre=sum(sum(handles.bw1));

%handles.min_img2=handles.min_img2-handles.meanBGMn;

PixelNumMinPre=sum(sum(handles.bwMin2));

TotalBGmaxPre=sum(sum(sum(handles.max_img2(handles.bw1==1))));

TotalBGminPre=sum(sum(sum(handles.min_img2(handles.bwMin2==1))));

%handles.meanBGmax=mean(mean(mean(handles.max_img1(handles.bw==1))));

%handles.meanBGmin=mean(mean(mean(handles.min_img1(handles.bwMin1==1))));

%LVEFpre=100*((TotalBGmaxPre-TotalBGminPre)/TotalBGmaxPre)

%clear handles.meanBGmax handles.meanBGmin

%handles.bw1 = ROI_semi_aut(handles.imvol2,2);

waitfor(handles.bw1);

%s11=regionprops(handles.bw1,'PixelList');

%PixelList1=[s11.PixelList];

handles.bw1 =uint16(handles.bw1);

% creat mask

handles.max_mask2=sum(handles.All_masks2,3);

handles.max_mask2=uint16(im2bw(handles.max_mask2,0.1));

for i=1:16

handles.imvol3(:,:,i) = (handles.max_mask2.*handles.imvol2(:,:,i));

end

%% substraction of pre image

%handles.imvol3=handles.imvol3-handles.meanBG;

% calculate count/pixel

handles.mnzs1 = mean(nonzeros(handles.imvol3));

% calculate meanmean count/pixel ratio (post/pre), (to see wether increased TID is atributed to increased tc

%accomulqation.).

% lung uptake

mb=msgbox('Select Manually Lung ROI (Pre)','select roi');

waitfor(mb);

axes(handles.axes6);

imagesc(sum(handles.imvol2,3));colormap(jet),colorbar ;

% draw ROI

hh = imrect;

handles.bw22=createMask(hh);

handles.bw22 =uint16(handles.bw22);

% close Figure 4

% close Figure 3

% close Figure 31

% creat mask

for i=1:16

handles.imvol33(:,:,i) = (handles.bw22.*handles.imvol2(:,:,i));

end

%handles.imvol33=handles.imvol33-handles.meanBG;

% calculate count/pixel

handles.mnzs22 = mean(nonzeros(handles.imvol33));

handles.heart2lungpre = handles.mnzs22/handles.mnzs1; %LHR in excel sheet

msgbox('pre part is done, go to post part');

guidata(hObject, handles);

% hObject handle to pushbutton2 (see GCBO)

% eventdata reserved - to be defined in a future version of MATLAB

% handles structure with handles and user data (see GUIDATA)

function pushbutton3_Callback(hObject, eventdata, handles)

try

close 1

close 2

close 3

close 4

close Dialogue_box_pre_post

catch

end

% --- Executes on button press in pushbutton3.

%% Calculations

%%

%sync and entro &&&&&&&&&&&&&&&&&&&&&&&&&&&&&&&&&&&&&&&&&&

load sp_post sp_post;

load sp_pre sp_pre;

tag11=1;

save prepost1 tag11

[handles.All_masksx,AA1,bb1,cc1,dd1,ee1,ff1,gg1,hh1,aa,bb,cc,dd,ee,ff,gg,hh2,II,JJ,spp]=max_min_MUGA3(handles.imvol,handles.meanBGMx,sp_post,[],[]);

tag11=0;

save prepost1 tag11

[handles.All_masksx2,AA1,bb1,cc1,dd1,ee1,ff1,gg1,hh1,aa,bb,cc,dd,ee,ff,gg,hh2,II,JJ,spp]=max_min_MUGA3(handles.imvol2,handles.meanBGMx,sp_pre,gg1,hh1);

%

for i=1:16

handles.imvol1x(:,:,i) = (handles.All_masks1(:,:,i).*handles.imvol(:,:,i));

handles.imvol1xY(:,:,i) = (handles.All_masks2(:,:,i).*handles.imvol2(:,:,i));

end

[TT, TT1,TT2,TT3,Weight1]=LVtimeActivityCurve(handles.imvol1x,2,0,[],1);

[TT, TT1,TT2,TT3,Weight2]=LVtimeActivityCurve(handles.imvol1xY,2,0,[],1);

%

for i=1:16

if(Weight1>0)

handles.All_masks1(:,:,i)=imdilate(handles.All_masks1(:,:,i),strel('disk',Weight1(i)));

handles.All_masksx(:,:,i)=imdilate(handles.All_masksx(:,:,i),strel('disk',Weight1(i)))

else

handles.All_masks1(:,:,i)=imerode(handles.All_masks1(:,:,i),strel('disk',abs(Weight1(i))));

handles.All_masksx(:,:,i)=imerode(handles.All_masksx(:,:,i),strel('disk',abs(Weight1(i))));

end

if(Weight2>0)

handles.All_masks2(:,:,i)=imdilate(handles.All_masks2(:,:,i),strel('disk',Weight2(i)));

handles.All_masksx2(:,:,i)=imdilate(handles.All_masksx2(:,:,i),strel('disk',Weight2(i)));

else

handles.All_masks2(:,:,i)=imerode(handles.All_masks2(:,:,i),strel('disk',abs(Weight2(i))));

handles.All_masksx2(:,:,i)=imerode(handles.All_masksx2(:,:,i),strel('disk',abs(Weight2(i))));

end

end

handles.max_maskx=sum(handles.All_masksx,3);

handles.max_maskx=uint16(im2bw(handles.max_maskx,0.1));

%

handles.max_maskx2=sum(handles.All_masksx2,3);

handles.max_maskx2=uint16(im2bw(handles.max_maskx2,0.1));

%

for i=1:16

handles.imvol1E(:,:,i) = (handles.max_maskx.*medfilt2(handles.imvol(:,:,i),[5 5]));

handles.imvol1m(:,:,i) = (handles.max_maskx.*handles.imvol(:,:,i));

handles.imvol3E(:,:,i) = (handles.max_maskx2.*medfilt2(handles.imvol2(:,:,i),[5 5]));

handles.imvol31(:,:,i) = (handles.max_maskx2.*(handles.imvol2(:,:,i)));

end

%figure,imshow(sum(handles.imvol3E,3),[])

%max(handles.imvol3E(:))

hf=figure;

save handles handles

load energyPost energy numofIter

for i=1:16

ax=handles.imvol(:,:,i);

ax(edge(handles.All_masks1(:,:,i))==1)=0;

ha(i)=subplot(4,4,i),imshow(ax,[]);colormap('jet');

end

pos = get(ha, 'position');

dim = cellfun(@(x) x.*[1 1 0.5 0.5], pos, 'uni',0);

for i=1:16

imvol_for_count(:,:,i)=handles.imvol(:,:,i).*uint16(handles.All_masks1(:,:,i));

Gray_count(i)=sum(sum(imvol_for_count(:,:,i)));

pixel_count(i)=sum(sum(handles.All_masks1(:,:,i)));

txt=strcat('E= ',num2str(energy(i)),', Itr= ',num2str(numofIter(i)),', GC= ',num2str(Gray_count(i)),', PC= ',num2str(pixel_count(i)));

annotation(hf, 'textbox', dim{i}-0.02, 'String', num2str(txt), 'vert', 'bottom', 'EdgeColor','none','FitBoxToText','off');

end

sgtitle('Post');

save postInf energy numofIter Gray_count pixel_count

hf = figure;

load energyPre energy numofIter

for i=1:16

ax=handles.imvol2(:,:,i);

ax(edge(handles.All_masks2(:,:,i))==1)=0;

ha(i)=subplot(4,4,i),imshow(ax,[]);colormap('jet');

end

pos = get(ha, 'position');

dim = cellfun(@(x) x.*[1 1 0.5 0.5], pos, 'uni',0);

for i=1:16

imvol_for_count(:,:,i)=handles.imvol2(:,:,i).*uint16(handles.All_masks2(:,:,i));

Gray_count(i)=sum(sum(imvol_for_count(:,:,i)));

pixel_count(i)=sum(sum(handles.All_masks2(:,:,i)));

txt=strcat('E= ',num2str(energy(i)),', Itr= ',num2str(numofIter(i)),', GC= ',num2str(Gray_count(i)),', PC= ',num2str(pixel_count(i)));

annotation(hf, 'textbox', dim{i}-0.02, 'String', num2str(txt), 'vert', 'bottom', 'EdgeColor','none','FitBoxToText','off');

end

sgtitle('Pre');

save preInf energy numofIter Gray_count pixel_count

energy_Pcount_Gcount_iteration_To_excel; % call script to insert data into excel sheet energy

handles.post2pre = handles.mnzs/handles.mnzs1

meanBG=(handles.meanBGMx2+handles.meanBGMn2)/2;

%meanBG=min([handles.meanBGMx2,handles.meanBGMn2]);

handles.imvol1m=uint16(double(handles.imvol1m)-double(meanBG));

handles.imvol1E=uint16(double(handles.imvol1E)-double(meanBG));

tag=1;

save prepost tag

[,Sync_post]=computeEntropySynchronyNo(handles.imvol1m,-1,-1,0);

[Entro_post,]=computeEntropySynchronyNo(handles.imvol1E,-1,-1,10);

xEn=sum(handles.imvol1E,3);

A=xEn(xEn~=0);

ApEnPost= ApEn_slow(A, 2,1*std(A));

save A A

[BoundApEnPost,epsilonPost,LspsilonPost]=BoundedProcess(A);

A1=rad2deg(angle(fft(A)));

A2=0;

for i=1:size(A1,1)

if (A1(i)<0)

A2=A2+(180-A1(i));

end

if (A1(i)>0)

A2=A2+(A1(i));

end

end

phasePost=sum(A2)/size(A1,1);

tag=0;

save prepost tag

meanBG=(handles.meanBGMx2+handles.meanBGMn2)/2;

%meanBG=min([handles.meanBGMx,handles.meanBGMn]);

handles.imvol31=uint16(double(handles.imvol31)-double(meanBG));

handles.imvol3E=uint16(double(handles.imvol3E)-double(meanBG));

[,Sync_pre]=computeEntropySynchronyNo(handles.imvol31,-1,-1,0);

[Entro_pre,]=computeEntropySynchronyNo(handles.imvol3E,-1,-1,10);

xEn=sum(handles.imvol3E,3);

A=xEn(xEn~=0);

save xEn xEn

ApEnPre= ApEn_slow(A, 2, 1*std(A));

[BoundApEnPre,epsilonPre,LspsilonPre]=BoundedProcess(A);

A1=rad2deg(angle(fft(A)));

A2=0;

for i=1:size(A1,1)

if (A1(i)<0)

A2=A2+(180-A1(i));

end

if (A1(i)>0)

A2=A2+(A1(i));

end

end

phasePre=sum(A2)/size(A1,1);

% close all previous figures

%close all

%close Figure 4

% close Figure 3

% close Figure 31

%% LVEF Phase

%%

[phase] = PhaseBkgFn(handles.imvol,handles.bw,sp_post,1);

[TotalBGmaxPostMXMN, TotalBGminPostMXMN ,indMaxMXMN,indMinMXMN,nmn]=LVtimeActivityCurve(phase,0,2,[],1);

%figure

PixelNumMaxPostMXMN=sum(sum(handles.All_masks1(:,:,indMaxMXMN(1))));

PixelNumMinPostMXMN=sum(sum(handles.All_masks1(:,:,indMinMXMN(1))));

TotalBGmaxPostMXMN=TotalBGmaxPostMXMN-(PixelNumMaxPostMXMN*handles.meanBGMn2);

TotalBGminPostMXMN=TotalBGminPostMXMN-(PixelNumMinPostMXMN*handles.meanBGMn2);

LVEFpostPhase=100*(((TotalBGmaxPostMXMN)-(TotalBGminPostMXMN))/(TotalBGmaxPostMXMN));

[phase] = PhaseBkgFn(handles.imvol2,handles.bw1,sp_pre,0);

[TotalBGmaxPreMXMN, TotalBGminPreMXMN ,indMaxMXMN,indMinMXMN,nmn]=LVtimeActivityCurve(phase,0,2,[],1);

figure

PixelNumMaxPreMXMN=sum(sum(handles.All_masks1(:,:,indMaxMXMN(1))));

PixelNumMinPreMXMN=sum(sum(handles.All_masks1(:,:,indMinMXMN(1))));

TotalBGmaxPreMXMN=TotalBGmaxPreMXMN-(PixelNumMaxPreMXMN*handles.meanBGMn2);

TotalBGminPreMXMN=TotalBGminPreMXMN-(PixelNumMinPreMXMN*handles.meanBGMn2);

LVEFprePhase=100*(((TotalBGmaxPreMXMN)-(TotalBGminPreMXMN))/(TotalBGmaxPreMXMN));

%%

%% show results

%% draw LV time activity curve for post image

%figure

%subplot(1,2,1)

handles.max_mask=sum(handles.All_masks1,3);

handles.max_mask=uint16(im2bw(handles.max_mask,0.1));

handles.max_mask2=sum(handles.All_masks2,3);

handles.max_mask2=uint16(im2bw(handles.max_mask2,0.1));

% handles.imvolx=handles.imvol;

% handles.imvolx2=handles.imvol2;

handles.imvolxY=uint16(double(handles.imvol)-double(handles.meanBGMn2));

handles.imvolxY2=uint16(double(handles.imvol2)-double(handles.meanBGMn));

handles.imvolx=handles.imvol;

handles.imvolx2=handles.imvol2;

for i=1:16

handles.imvol1x(:,:,i) = (handles.All_masks1(:,:,i).*handles.imvolx(:,:,i));

handles.imvol3x(:,:,i) = (handles.All_masks2(:,:,i).*handles.imvolx2(:,:,i));

handles.imvol1xY(:,:,i) = (handles.All_masks1(:,:,i).*handles.imvolxY(:,:,i));

handles.imvol3xY(:,:,i) = (handles.All_masks2(:,:,i).*handles.imvolxY2(:,:,i));

end

[TotalBGmaxPost1, TotalBGminPost1 ,indMax,indMin,nmn]=LVtimeActivityCurve(handles.imvol1x,1,0,[],1);

% compute entropy, apen and bounded entropy based on max and min images only

handles.imvol1E1(:,:,1)=handles.imvol1x(:,:,indMin);

handles.imvol1E1(:,:,2)=handles.imvol1x(:,:,indMax);

handles.imvol1m1(:,:,1)=handles.imvol1x(:,:,indMin);

handles.imvol1m1(:,:,2)=handles.imvol1x(:,:,indMax);

[Entro_PostMXMN,]=computeEntropySynchronyNo(handles.imvol1E1,indMax,indMin,0);

[,Synchrony_PostMXMN]=computeEntropySynchronyNo(handles.imvol1m1,indMax,indMin,0);

xEn=sum(handles.imvol1E1,3);

A=xEn(xEn~=0);

ApEnPostMXMN= ApEn_slow(A, 2,1*std(A));

[BoundApEnPostMXMN,epsilonPostMXMN,LspsilonPostMXMN]=BoundedProcess(A);

[TotalBGmaxPostY1, TotalBGminPostY1 ,indMaxY,indMinY,nmn]=LVtimeActivityCurve(handles.imvol1xY,1,handles.imvol1x,[],0);

%handles.All_masks1(:,:,indMax)=imdilate(handles.All_masks1(:,:,indMax),strel('disk',2));

%handles.All_masks1(:,:,indMin)=imdilate(handles.All_masks1(:,:,indMin),strel('disk',2));

PixelNumMaxPost=sum(sum(handles.All_masks1(:,:,indMax(1))))%max count in excel sheet

PixelNumMinPost=sum(sum(handles.All_masks1(:,:,indMin(1))))%min count in excel sheet

TotalBGmaxPost=TotalBGmaxPost1-(PixelNumMaxPost*handles.meanBGMn2); %max count in excel sheet

TotalBGminPost=TotalBGminPost1-(PixelNumMinPost*handles.meanBGMn2); %min count in excel sheet

%

PixelNumMaxPostY=sum(sum(handles.All_masks1(:,:,indMaxY(1))));

PixelNumMinPostY=sum(sum(handles.All_masks1(:,:,indMinY(1))));

TotalBGmaxPostY=TotalBGmaxPostY1-(PixelNumMaxPostY*handles.meanBGMn2);

TotalBGminPostY=TotalBGminPostY1-(PixelNumMinPostY*handles.meanBGMn2);

%compute LVEF based on max and min ROIs only

A1=uint16(zeros(size(handles.imvol1x)));A2=A1;

for i=1:16

A1(:,:,i)=rad2deg(angle(fftshift(fft2(handles.imvol1x(:,:,i)))));

if (A1(:,:,i)<0)

A2(:,:,i)=(180-A1(:,:,i));

end

if (A1(:,:,i)>0)

A2(:,:,i)=(A1(:,:,i));

end

end

%[TotalBGmaxPostMXMN, TotalBGminPostMXMN

%,indMaxMXMN,indMinMXMN,nmn]=LVtimeActivityCurve(A1,1,2,[],0);.

%%

%

%LVEFpost=100*(((TotalBGmaxPost-PixelNumMaxPost*handles.meanBG)-(TotalBGminPost-PixelNumMinPost*handles.meanBG))/(TotalBGmaxPost-PixelNumMaxPost*handles.meanBG))

LVEFpost=100*(((TotalBGmaxPost)-(TotalBGminPost))/(TotalBGmaxPost));

LVEFpost2=100*(((TotalBGmaxPostY)-(TotalBGminPostY))/(TotalBGmaxPostY));

%subplot(1,2,2)

[TotalBGmaxPre1, TotalBGminPre1,indMax,indMin,nmn]=LVtimeActivityCurve(handles.imvol3x,2,0,[],1);

% compute entropy, apen and bounded entropy based on max and min images only

handles.imvol3x1(:,:,1)=handles.imvol3x(:,:,indMin);

handles.imvol3x1(:,:,2)=handles.imvol3x(:,:,indMax);

handles.imvol311(:,:,1)=handles.imvol3x(:,:,indMin);

handles.imvol311(:,:,2)=handles.imvol3x(:,:,indMax);

[Entro_PreMXMN,]=computeEntropySynchronyNo(handles.imvol3x1,indMax,indMin,0);

[,Synchrony_PreMXMN]=computeEntropySynchronyNo(handles.imvol311,indMax,indMin,0);

xEn=sum(handles.imvol1E1,3);

A=xEn(xEn~=0);

ApEnPreMXMN= ApEn_slow(A, 2,1*std(A));

[BoundApEnPreMXMN,epsilonPreMXMN,LspsilonPreMXMN]=BoundedProcess(A);

[TotalBGmaxPreY1, TotalBGminPreY1,indMaxY,indMinY,nmn]=LVtimeActivityCurve(handles.imvol3xY,2,handles.imvol3x,[],0);

%handles.All_masks1(:,:,indMax)=imdilate(handles.All_masks1(:,:,indMax),strel('disk',2));

%handles.All_masks1(:,:,indMin)=imdilate(handles.All_masks1(:,:,indMin),strel('disk',2));

PixelNumMaxPre=sum(sum(handles.All_masks2(:,:,indMax(1))))% max pixel num in excel sheet

% indMax

% indMin

PixelNumMinPre=sum(sum(handles.All_masks2(:,:,indMin(1)))) % min pixel num in excel sheet

TotalBGmaxPre=TotalBGmaxPre1-(PixelNumMaxPre*handles.meanBGMn);%max count in excel sheet

TotalBGminPre=TotalBGminPre1-(PixelNumMinPre*handles.meanBGMn);%min count in excel sheet

PixelNumMaxPreY=sum(sum(handles.All_masks2(:,:,indMaxY(1))));

PixelNumMinPreY=sum(sum(handles.All_masks2(:,:,indMinY(1))));

TotalBGmaxPreY=TotalBGmaxPreY1-(PixelNumMaxPreY*handles.meanBGMn);

TotalBGminPreY=TotalBGminPreY1-(PixelNumMinPreY*handles.meanBGMn);

%LVEFpre=100*(((TotalBGmaxPre-PixelNumMaxPre*handles.meanBG)-(TotalBGminPre-PixelNumMinPre*handles.meanBG))/(TotalBGmaxPre-PixelNumMaxPre*handles.meanBG))

LVEFpre=100*(((TotalBGmaxPre-TotalBGminPre))/(TotalBGmaxPre));

LVEFpre2=100*(((TotalBGmaxPreY-TotalBGminPreY))/(TotalBGmaxPreY));

%compute LVEF based on phase image only

A1=uint16(zeros(size(handles.imvol3x)));A2=A1;

for i=1:16

A1(:,:,i)=rad2deg(angle(fftshift(fft2(handles.imvol3x(:,:,i)))));

if (A1(:,:,i)<0)

A2(:,:,i)=(180+A1(:,:,i));

end

if (A1(:,:,i)>0)

A2(:,:,i)=(A1(:,:,i));

end

end

%[TotalBGmaxPreMXMN, TotalBGminPreMXMN ,indMaxMXMN,indMinMXMN,nmn]=LVtimeActivityCurve(A1,2,2,[],0);

close;

%% save

%Extract some information from post image into workspace

load info info;

PatientID=info.PatientID;

PatientAge=info.PatientAge;

PatientWeight=info.PatientWeight;

AcquisitionDate=info.AcquisitionDate;

Manufacturer=info.Manufacturer;

HeartRate=info.HeartRate;

FrameTime=info.GatedInformationSequence.Item_1.DataInformationSequence.Item_1.FrameTime;

LowRRValue=info.GatedInformationSequence.Item_1.DataInformationSequence.Item_1.LowRRValue;

HighRRValue=info.GatedInformationSequence.Item_1.DataInformationSequence.Item_1.HighRRValue;%(mean RR duration) in excel sheet

IntervalsAcquired=info.GatedInformationSequence.Item_1.DataInformationSequence.Item_1.IntervalsAcquired;% Accepted beats in excel sheet

IntervalsRejected=info.GatedInformationSequence.Item_1.DataInformationSequence.Item_1.IntervalsRejected; % rejected beats in excel sheet

% info of pre image

load info2 info2;

PatientAge2=info2.PatientAge;

PatientWeight2=info2.PatientWeight;

AcquisitionDate2=info2.AcquisitionDate;

HeartRate2=info2.HeartRate;

FrameTime2=info2.GatedInformationSequence.Item_1.DataInformationSequence.Item_1.FrameTime;

LowRRValue2=info2.GatedInformationSequence.Item_1.DataInformationSequence.Item_1.LowRRValue;

HighRRValue2=info2.GatedInformationSequence.Item_1.DataInformationSequence.Item_1.HighRRValue; % (mean RR duration) in excel sheet

IntervalsAcquired2=info2.GatedInformationSequence.Item_1.DataInformationSequence.Item_1.IntervalsAcquired; % Accepted beats in excel sheet

IntervalsRejected2=info2.GatedInformationSequence.Item_1.DataInformationSequence.Item_1.IntervalsRejected; % rejected beats in excel sheet

area1Post=handles.area1Post;area2Post=handles.area2Post;

AverageDivPost=handles.AverageDivPost; circularityMaxPost=handles.circularityMaxPost;

circularityMinPost=handles.circularityMinPost; ElongationMaxPost=handles.ElongationMaxPost;

ElongationMinPost=handles.ElongationMinPost; heart2lungpost=handles.heart2lungpost;

minLongDIVmaxLPost=handles.minLongDIVmaxLPost; minShortDIVmaxLPost=handles.minShortDIVmaxLPost;

post2pre=handles.post2pre; ratePost=handles.ratePost;TIDmax=handles.TIDmax; TIDmin=handles.TIDmin;

PixelsBkgMaxPost=handles.PixelsBkgMaxPost;

PixelsBkgMinPost=handles.PixelsBkgMinPost;

meanBGMx2=handles.meanBGMx2; meanBGMn2=handles.meanBGMn2;

save post AcquisitionDate area1Post area2Post AverageDivPost circularityMaxPost circularityMinPost ElongationMaxPost ...

ElongationMinPost Entro_post ApEnPost FrameTime heart2lungpost HeartRate HighRRValue IntervalsAcquired IntervalsRejected LowRRValue...

LVEFpost Manufacturer minLongDIVmaxLPost minShortDIVmaxLPost PatientAge PatientID PatientWeight PixelNumMaxPost PixelNumMinPost...

post2pre ratePost Sync_post TIDmax TIDmin TotalBGmaxPost TotalBGmaxPost1 TotalBGminPost TotalBGminPost1 phasePost PixelsBkgMaxPost...

PixelsBkgMinPost meanBGMx2 meanBGMn2 BoundApEnPost epsilonPost LspsilonPost ApEnPostMXMN Entro_PostMXMN BoundApEnPostMXMN LVEFpostPhase Synchrony_PostMXMN

area1Pre=handles.area1Pre; area2Pre=handles.area2Pre; AverageDivPre=handles.AverageDivPre;

circularityMaxPre=handles.circularityMaxPre; circularityMinPre=handles.circularityMinPre;

ElongationMaxPre=handles.ElongationMaxPre; ElongationMinPre=handles.ElongationMinPre;

heart2lungpre=handles.heart2lungpre; minLongDIVmaxLPre=handles.minLongDIVmaxLPre; minShortDIVmaxLPre=handles.minShortDIVmaxLPre;

post2pre=handles.post2pre; ratePre=handles.ratePre; TIDmax=handles.TIDmax; TIDmin=handles.TIDmin;

PixelsBkgMaxPre=handles.PixelsBkgMaxPre;

PixelsBkgMinPre=handles.PixelsBkgMinPre;

meanBGMx=handles.meanBGMx; meanBGMn=handles.meanBGMn;

save pre AcquisitionDate2 area1Pre area2Pre AverageDivPre circularityMaxPre circularityMinPre ElongationMaxPre ...

ElongationMinPre Entro_pre ApEnPre FrameTime2 heart2lungpre HeartRate2 HighRRValue2 IntervalsAcquired2 IntervalsRejected2 LowRRValue2...

LVEFpre Manufacturer minLongDIVmaxLPre minShortDIVmaxLPre PatientAge2 PatientID PatientWeight2 PixelNumMaxPre PixelNumMinPre...

post2pre ratePre Sync_pre TIDmax TIDmin TotalBGmaxPre TotalBGmaxPre1 TotalBGminPre TotalBGminPre1 phasePre PixelsBkgMaxPre PixelsBkgMinPre...

meanBGMx meanBGMn BoundApEnPre epsilonPre LspsilonPre ApEnPreMXMN Entro_PreMXMN BoundApEnPreMXMN LVEFprePhase Synchrony_PreMXMN

max_img1=handles.max_img1;imvolmx1=handles.imvolmx1;min_img1=handles.min_img1;

imvolmn1=handles.imvolmn1;max_img2=handles.max_img2;imvolmx2=handles.imvolmx2;

min_img2=handles.min_img2;imvolmn2=handles.imvolmn2;smoothedPoly11=handles.smoothedPoly11;

smoothedPoly12=handles.smoothedPoly12; smoothedPoly21=handles.smoothedPoly21; smoothedPoly22=handles.smoothedPoly22;

save deletedInfo max_img1 imvolmx1 min_img1 imvolmn1 max_img2 imvolmx2 min_img2 imvolmn2 smoothedPoly11 smoothedPoly12 smoothedPoly21 smoothedPoly22;

Dialogue_box_pre_post

guidata(hObject, handles);

% hObject handle to pushbutton3 (see GCBO)

% eventdata reserved - to be defined in a future version of MATLAB

% handles structure with handles and user data (see GUIDATA)

% --- Executes on button press in pushbutton4.

function pushbutton4_Callback(hObject, eventdata, handles)

% hObject handle to pushbutton4 (see GCBO)

% eventdata reserved - to be defined in a future version of MATLAB

% handles structure with handles and user data (see GUIDATA)

I=imread('help.png');

figure

imagesc(I);

% --- Executes on slider movement.

function slider1_Callback(hObject, eventdata, handles)

% hObject handle to slider1 (see GCBO)

% eventdata reserved - to be defined in a future version of MATLAB

% handles structure with handles and user data (see GUIDATA)

axes(handles.axes1)

imvolx=sum(handles.imvol,3);

L1=get(handles.slider1,'value');

L2=get(handles.slider2,'value');

imshow(im2uint8(mat2gray(imvolx))) , hold all,colormap(jet),colorbar;

try

caxis([L1 L2]);

catch

b=msgbox('Wrong scale, right bar must be greater than left one');

set(handles.slider1,'value',0);

set(handles.slider2,'value',255);

end

% Hints: get(hObject,'Value') returns position of slider

% get(hObject,'Min') and get(hObject,'Max') to determine range of slider

% --- Executes during object creation, after setting all properties.

function slider1_CreateFcn(hObject, eventdata, handles)

% hObject handle to slider1 (see GCBO)

% eventdata reserved - to be defined in a future version of MATLAB

% handles empty - handles not created until after all CreateFcns called

% Hint: slider controls usually have a light gray background.

if isequal(get(hObject,'BackgroundColor'), get(0,'defaultUicontrolBackgroundColor'))

set(hObject,'BackgroundColor',[.9 .9 .9]);

end

% --- Executes on slider movement.

function slider2_Callback(hObject, eventdata, handles)

axes(handles.axes1)

imvolx=sum(handles.imvol,3);

L1=get(handles.slider1,'value');

L2=get(handles.slider2,'value');

imshow(im2uint8(mat2gray(imvolx))) , hold all,colormap(jet),colorbar;

try

caxis([L1 L2]);

catch

b=msgbox('Wrong scale, right bar must be greater than left one');

set(handles.slider1,'value',0);

set(handles.slider2,'value',255);

end

% hObject handle to slider2 (see GCBO)

% eventdata reserved - to be defined in a future version of MATLAB

% handles structure with handles and user data (see GUIDATA)

% Hints: get(hObject,'Value') returns position of slider

% get(hObject,'Min') and get(hObject,'Max') to determine range of slider

% --- Executes during object creation, after setting all properties.

function slider2_CreateFcn(hObject, eventdata, handles)

% hObject handle to slider2 (see GCBO)

% eventdata reserved - to be defined in a future version of MATLAB

% handles empty - handles not created until after all CreateFcns called

% Hint: slider controls usually have a light gray background.

if isequal(get(hObject,'BackgroundColor'), get(0,'defaultUicontrolBackgroundColor'))

set(hObject,'BackgroundColor',[.9 .9 .9]);

end

% --- Executes on slider movement.

function slider3_Callback(hObject, eventdata, handles)

axes(handles.axes4)

imvolx=sum(handles.imvol2,3);

L1=get(handles.slider3,'value');

L2=get(handles.slider4,'value');

imshow(im2uint8(mat2gray(imvolx))) , hold all,colormap(jet),colorbar;

try

caxis([L1 L2]);

catch

b=msgbox('Wrong scale, right bar must be greater than left one');

set(handles.slider3,'value',0);

set(handles.slider4,'value',255);

end

% hObject handle to slider3 (see GCBO)

% eventdata reserved - to be defined in a future version of MATLAB

% handles structure with handles and user data (see GUIDATA)

% Hints: get(hObject,'Value') returns position of slider

% get(hObject,'Min') and get(hObject,'Max') to determine range of slider

% --- Executes during object creation, after setting all properties.

function slider3_CreateFcn(hObject, eventdata, handles)

% hObject handle to slider3 (see GCBO)

% eventdata reserved - to be defined in a future version of MATLAB

% handles empty - handles not created until after all CreateFcns called

% Hint: slider controls usually have a light gray background.

if isequal(get(hObject,'BackgroundColor'), get(0,'defaultUicontrolBackgroundColor'))

set(hObject,'BackgroundColor',[.9 .9 .9]);

end

% --- Executes on slider movement.

function slider4_Callback(hObject, eventdata, handles)

axes(handles.axes4)

imvolx=sum(handles.imvol2,3);

L1=get(handles.slider3,'value');

L2=get(handles.slider4,'value');

imshow(im2uint8(mat2gray(imvolx))) , hold all,colormap(jet),colorbar;

try

caxis([L1 L2]);

catch

b=msgbox('Wrong scale, right bar must be greater than left one');

set(handles.slider3,'value',0);

set(handles.slider4,'value',255);

end

% hObject handle to slider4 (see GCBO)

% eventdata reserved - to be defined in a future version of MATLAB

% handles structure with handles and user data (see GUIDATA)

% Hints: get(hObject,'Value') returns position of slider

% get(hObject,'Min') and get(hObject,'Max') to determine range of slider

% --- Executes during object creation, after setting all properties.

function slider4_CreateFcn(hObject, eventdata, handles)

% hObject handle to slider4 (see GCBO)

% eventdata reserved - to be defined in a future version of MATLAB

% handles empty - handles not created until after all CreateFcns called

% Hint: slider controls usually have a light gray background.

if isequal(get(hObject,'BackgroundColor'), get(0,'defaultUicontrolBackgroundColor'))

set(hObject,'BackgroundColor',[.9 .9 .9]);

end

% --- Executes on button press in pushbutton6.

function pushbutton6_Callback(hObject, eventdata, handles)

try

close 1

close 2

close 3

close 4

close Dialogue_box_pre_post

catch

end

x2 = inputdlg('Pick Pre image:',...

'Define Image', [1]);

handles.name2= (x2{:}); % image number

handles.imvol2 = squeeze(dicomread(handles.name2));

info2 = dicominfo(handles.name2);

save info2 info2;

for i=1:1:info2.NumberOfFrames

handles.imvol2(:,:,i)=medfilt2(handles.imvol2(:,:,i));

end

axes(handles.axes4)

imvolx=sum(handles.imvol2,3);

L1=get(handles.slider3,'value');

L2=get(handles.slider4,'value');

imshow(im2uint8(mat2gray(imvolx))) , hold all,colormap(jet),colorbar;

try

caxis([L1 L2]);

catch

b=msgbox('Wrong scale, right bar must be greater than left one');

set(handles.slider3,'value',0);

set(handles.slider4,'value',255);

end

b=msgbox('slide right and left bars for colors');

waitfor(b);

guidata(hObject, handles);

% hObject handle to pushbutton6 (see GCBO)

% eventdata reserved - to be defined in a future version of MATLAB

% handles structure with handles and user data (see GUIDATA)

% --- Executes on button press in pushbutton7.

function pushbutton7_Callback(hObject, eventdata, handles)

try

close 1

close 2

close 3

close 4

close Dialogue_box_pre_post

catch

end

% read images (post-chemo).

x = inputdlg('Pick Post image:',...

'Define Image', [1]);

handles.name= (x{:}); % image number

handles.imvol = squeeze(dicomread(handles.name));

info = dicominfo(handles.name);

save info info;

for i=1:1:info.NumberOfFrames

handles.imvol(:,:,i)=medfilt2(handles.imvol(:,:,i));%

end

axes(handles.axes1);

imvolx=sum(handles.imvol,3);

L1=get(handles.slider1,'value');

L2=get(handles.slider2,'value');

imshow(im2uint8(mat2gray(imvolx))) , hold all,colormap(jet),colorbar;

try

caxis([L1 L2]);

catch

b=msgbox('Wrong scale, right bar must be greater than left one');

set(handles.slider1,'value',0);

set(handles.slider2,'value',255);

end

b=msgbox('slide right and left bars for colors');

waitfor(b);

guidata(hObject, handles);

% hObject handle to pushbutton7 (see GCBO)

% eventdata reserved - to be defined in a future version of MATLAB

% handles structure with handles and user data (see GUIDATA)

%%%%%%%%%%%%%%%%%%%%%%%%%%%%%%%%%%%%%%%%%%%%%%%%

| **file name** | clinic_heart_disease_system.fig |
| --- | --- |
| **Type** | Figure file |
| **Description** | The design of the main code (GUI design) |
| **comments** | Novel design |

**Code:**

It is design not code.

%%%%%%%%%%%%%%%%%%%%%%%%%%%%%%%%%%%%%%%%%%%%%%%%

| **M-file name** | computeEntropySynchrony |
| --- | --- |
| **Type** | Fun |
| **Description** | Entropy & Synchrony Calculation  Inputs: Vol is the ROI to compute synchrony and entropy for. Outputs are the entropy and synchrony. This function plot and show some results. |
| **comments** | Novel. |

**Code:**

%%%%%%%%%%%%%%%%%%%%%%%%%%%%%%%%%%%%%%%%%%%%%

%% Entropy & Synchrony Calculation %%

%%%%%%%%%%%%%%%%%%%%%%%%%%%%%%%%%%%%%%%%%%%%%

function [entropy,synchrony]=computeEntropySynchrony(vol,par1,par2,par3)

[m n l]=size(vol);

vectora = zeros(m,n);

vectorb = zeros(m,n);

%create a temporary array for image statistics for each loop

temparray = uint16(zeros(1,l));

%create a permanant array of amplitudes and initialise it to 0

amparr = zeros(m,n);

%create a permanant array of phases and initialise it to 0

phasearr = zeros(m,n);

%initialise a counter

c = 0;

volx=sum(vol,3);

for x = 1:1:m

for y = 1:1:n

temparray(:) = 0;

for i = 1:1:l

if (volx(x,y,1) ~= 0)

temparray(i) = vol(x ,y,i );

end%if

end% for

% this calculates the amplitude and the phase in the image

% temparray = medfilt2(temparray,[2 2]);

maxvalue=max(temparray);

if(maxvalue~=0)

maxPoint=find(maxvalue==temparray);

maxlocation=ind2sub(size(temparray),maxPoint);

tt=temparray;

tt(temparray==0)=[];

minvalue=min(tt);

minPoint=find(minvalue==temparray);

% minlocation=ind2sub(size(temparray),minPoint)

amparr(x,y ) = maxvalue - minvalue;

phasearr(x,y) = minPoint(1);

% this calculates the parameters necessary for the synchrony of the heart

if (volx(x ,y ,1) ~= 0)

vectora(x ,y ) = amparr(x ,y ) .* cos(phasearr(x ,y ) ./ 2 .* pi);

vectorb(x ,y ) = amparr(x ,y ) .* sin(phasearr(x ,y ) ./ 2 .* pi);

c = c + 1;

end%if

end

end% for

end% for

save phasearr phasearr vectora vectorb volx

if (par1==-1&par3==10)

%load ind_max ind_max

load prepost tag

figure

subplot(2,2,1),imshow(volx,[]),colormap('gray');colorbar;

if (tag==0)

title('Pre ROI image');

else

title('Post ROI image');

end

A=rad2deg(atan((vectorb+1)./(vectora+1)));

A2=sqrt((vectora.^2)+(vectorb.^2));

%A3=im2bw(sum(A,3),0.01);

subplot(2,2,2),imshow(abs(A),[0 10]),colormap('jet');colorbar;

if (tag==0)

title('Pre Phase image');

else

title('Post Phase image');

end

subplot(2,2,3),

[counts, grayLevels] = MyHistogram(abs(A),A2);

%hist(A);

if (tag==0)

title('Pre Phase histogram');

else

title('Post Phase histogram');

end

%ss=hist(A);

% axis([0 360 0 max(counts)])

xlabel('degree', 'FontSize', 10);

ylabel('frequency', 'FontSize', 10);

subplot(2,2,4),

histogram(abs(A),360);

axis([0 360 0 max(counts)]);

%imshow(A2,[])

xlabel('degree', 'FontSize', 10);

ylabel('frequency', 'FontSize', 10);

%A=phasearr;

end

% A1=rad2deg(angle(fft(A)));

% hist(A)

% for i=1:size(A,1)

% if (A(i)<0)

% A2(i)=(180-A(i));

% end

% if (A(i)>0)

% A2(i)=(A(i));

% end

% end

%this calculates the parameters necessary for the entropy of the heart

phasearr1=phasearr(phasearr~=0);

u=unique(phasearr1);

histV=zeros(size(u));

for i=1:size(phasearr1,1)

for k=1:size(u,1)

if(u(k)==phasearr1(i))

histV(k)=histV(k)+1;

end

end

end

% histV = imhist(uint8(unique(phasearr1)),info.NumberOfFrames);

phist = histV ./ sum(histV);

p=phist(phist~=0);

%this calculates entropy

entropy = -sum(p.*log2(p))/log2(l);

%this calculates synchrony

denominator = sum(sum(sqrt(vectora.^2 + vectorb.^2)));

toti = sum(sum(abs(vectora)));

totj = sum(sum(abs(vectorb)));

numerator = sqrt(toti.^2 + totj.^2);

synchrony = (numerator ./ denominator);

%%%%%%%%%%%%%%%%%%%%%%%%%%%%%%%%%%%%%%%%%%%%%%%%

| **M-file name** | computeEntropySynchronyNo |
| --- | --- |
| **Type** | Fun |
| **Description** | As same as 6, but he only difference is that in this function there are no plots nor figures. |
| **comments** | Novel: Last modified: 29-5-2021 |

**Code**: As same as 6, but without plot statements

%%%%%%%%%%%%%%%%%%%%%%%%%%%%%%%%%%%%%%%%%%%%%%%%

| **M-file name** | Dialogue_box_pre_post.m |
| --- | --- |
| **Type** | M file of GUI |
| **Description** | Dialogue box to show results of the main GUI inside it |
| **comments** | Novel: Last modified 18-3-2021 |

**Code:**

function varargout = Dialogue_box_pre_post(varargin)

% DIALOGUE_BOX_PRE_POST MATLAB code for Dialogue_box_pre_post.fig

% DIALOGUE_BOX_PRE_POST, by itself, creates a new DIALOGUE_BOX_PRE_POST or raises the existing

% singleton*.

%

% H = DIALOGUE_BOX_PRE_POST returns the handle to a new DIALOGUE_BOX_PRE_POST or the handle to

% the existing singleton*.

%

% DIALOGUE_BOX_PRE_POST('CALLBACK',hObject,eventData,handles,...) calls the local

% function named CALLBACK in DIALOGUE_BOX_PRE_POST.M with the given input arguments.

%

% DIALOGUE_BOX_PRE_POST('Property','Value',...) creates a new DIALOGUE_BOX_PRE_POST or raises the

% existing singleton*. Starting from the left, property value pairs are

% applied to the GUI before Dialogue_box_pre_post_OpeningFcn gets called. An

% unrecognized property name or invalid value makes property application

% stop. All inputs are passed to Dialogue_box_pre_post_OpeningFcn via varargin.

%

% *See GUI Options on GUIDE's Tools menu. Choose "GUI allows only one

% instance to run (singleton)".

%

% See also: GUIDE, GUIDATA, GUIHANDLES

% Edit the above text to modify the response to help Dialogue_box_pre_post

% Last Modified by GUIDE v2.5 02-Feb-2021 22:28:23

% Begin initialization code - DO NOT EDIT

gui_Singleton = 1;

gui_State = struct('gui_Name', mfilename, ...

'gui_Singleton', gui_Singleton, ...

'gui_OpeningFcn', @Dialogue_box_pre_post_OpeningFcn, ...

'gui_OutputFcn', @Dialogue_box_pre_post_OutputFcn, ...

'gui_LayoutFcn', [] , ...

'gui_Callback', []);

if nargin && ischar(varargin{1})

gui_State.gui_Callback = str2func(varargin{1});

end

if nargout

[varargout{1:nargout}] = gui_mainfcn(gui_State, varargin{:});

else

gui_mainfcn(gui_State, varargin{:});

end

% End initialization code - DO NOT EDIT

% --- Executes just before Dialogue_box_pre_post is made visible.

function Dialogue_box_pre_post_OpeningFcn(hObject, eventdata, handles, varargin)

% This function has no output args, see OutputFcn.

% hObject handle to figure

% eventdata reserved - to be defined in a future version of MATLAB

% handles structure with handles and user data (see GUIDATA)

% varargin command line arguments to Dialogue_box_pre_post (see VARARGIN)

% Choose default command line output for Dialogue_box_pre_post

handles.output = hObject;

load deletedInfo max_img1 imvolmx1 min_img1 imvolmn1 max_img2 imvolmx2 min_img2 imvolmn2 smoothedPoly11 smoothedPoly12 smoothedPoly21 smoothedPoly22;

%% show results

% pre

axes(handles.axes1)

imshow(max_img2,[]);title('Pre Diastole Image'),colormap(jet);

hold on

h=impoly(gca,smoothedPoly21);

setColor(h,'black');

hold off

axes(handles.axes2)

imshow(imvolmx2,[]);title('Pre Diastole ROI Image'),colormap(jet);

axes(handles.axes3)

imshow(min_img2,[]);title('Pre Systole Image'),colormap(jet);

hold on

h=impoly(gca,smoothedPoly22);

setColor(h,'black');

hold off

axes(handles.axes4)

imshow(imvolmn2,[]);title('Pre Systole ROI Image'),colormap(jet);

% post

axes(handles.axes5)

imshow(max_img1,[]);title('Post Diastole Image'),colormap(jet);

hold on

h=impoly(gca,smoothedPoly11);

setColor(h,'black');

hold off

axes(handles.axes6)

imshow(imvolmx1,[]);title('Post Diastole ROI Image'),colormap(jet);

axes(handles.axes7)

imshow(min_img1,[]);title('Post Systole Image'),colormap(jet);

hold on

h=impoly(gca,smoothedPoly12);

setColor(h,'black');

hold off

axes(handles.axes8)

imshow(imvolmn1,[]);title('Post Systole ROI Image'),colormap(jet);

% Update handles structure

% plot curves

load curvePre x y yy;

axes(handles.axes9);

%plot(x,y,'-*b');

yy = smooth(y);

%hold on;

plot(x,yy,'-*r');

title('Pre LV Time activity curve');

mx=max(y);

mn=min(y);

axis([x(1) x(end) 0 mx]);

xlabel('Time (msec)');

ylabel('Count (count)');

legend('Smoothed Curve',...

'Location','SW');

axes(handles.axes9)

load curvePost x y yy;

axes(handles.axes10);

%plot(x,y,'-*b');

yy = smooth(y);

%hold on;

plot(x,yy,'-*r');

title('Post LV Time activity curve');

mx=max(y);

mn=min(y);

axis([x(1) x(end) 0 mx]);

xlabel('Time (msec)');

ylabel('Count (count)');

legend('Smoothed Curve',...

'Location','SW');

load post AcquisitionDate area1Post area2Post AverageDivPost circularityMaxPost circularityMinPost ElongationMaxPost ...

ElongationMinPost Entro_post ApEnPost FrameTime heart2lungpost HeartRate HighRRValue IntervalsAcquired IntervalsRejected LowRRValue...

LVEFpost Manufacturer minLongDIVmaxLPost minShortDIVmaxLPost PatientAge PatientID PatientWeight PixelNumMaxPost PixelNumMinPost...

post2pre ratePost Sync_post TIDmax TIDmin TotalBGmaxPost TotalBGmaxPost1 TotalBGminPost TotalBGminPost1 phasePost PixelsBkgMaxPost PixelsBkgMinPost meanBGMx2 meanBGMn2 BoundApEnPost epsilonPost LspsilonPost

post=[ {PatientID} {PatientAge} {PatientWeight} {AcquisitionDate} {area1Post} {area2Post} {AverageDivPost} {circularityMaxPost} {circularityMinPost} {ElongationMaxPost} ...

{ElongationMinPost} {Entro_post} {ApEnPost} {FrameTime} {heart2lungpost} {HeartRate} {HighRRValue} {IntervalsAcquired} {IntervalsRejected} {LowRRValue}...

{LVEFpost} {Manufacturer} {minLongDIVmaxLPost} {minShortDIVmaxLPost} {PixelNumMaxPost} {PixelNumMinPost}...

{post2pre} {ratePost} {Sync_post} {TIDmax} {TIDmin} {TotalBGmaxPost} {TotalBGminPost} {TotalBGmaxPost1} {TotalBGminPost1} {PixelsBkgMaxPost} {PixelsBkgMinPost} {phasePost} {meanBGMx2} {meanBGMn2} {BoundApEnPost} {epsilonPost} {LspsilonPost}];

load pre AcquisitionDate2 area1Pre area2Pre AverageDivPre circularityMaxPre circularityMinPre ElongationMaxPre ...

ElongationMinPre Entro_pre ApEnPre FrameTime2 heart2lungpre HeartRate2 HighRRValue2 IntervalsAcquired2 IntervalsRejected2 LowRRValue2...

LVEFpre Manufacturer minLongDIVmaxLPre minShortDIVmaxLPre PatientAge2 PatientID PatientWeight2 PixelNumMaxPre PixelNumMinPre...

post2pre ratePre Sync_pre TIDmax TIDmin TotalBGmaxPre TotalBGmaxPre1 TotalBGminPre TotalBGminPre1 phasePre PixelsBkgMaxPre PixelsBkgMinPre meanBGMx meanBGMn BoundApEnPre epsilonPre LspsilonPre

pre=[{PatientID} {PatientAge} {PatientWeight2} {AcquisitionDate2} {area1Pre} {area2Pre} {AverageDivPre} {circularityMaxPre} {circularityMinPre} {ElongationMaxPre} ...

{ElongationMinPre} {Entro_pre} {ApEnPre} {FrameTime2} {heart2lungpre} {HeartRate2} {HighRRValue2} {IntervalsAcquired2} {IntervalsRejected2} {LowRRValue2}...

{LVEFpre} {Manufacturer} {minLongDIVmaxLPre} {minShortDIVmaxLPre} {PixelNumMaxPre} {PixelNumMinPre}...

{post2pre} {ratePre} {Sync_pre} {TIDmax} {TIDmin} {TotalBGmaxPre} {TotalBGminPre} {TotalBGmaxPre1} {TotalBGminPre1} {PixelsBkgMaxPre} {PixelsBkgMinPre} {phasePre} {meanBGMx} {meanBGMn} {BoundApEnPre} {epsilonPre} {LspsilonPre}];

post=post';

pre=pre';

set(handles.uitable3,'data',post);

set(handles.uitable1,'data',pre);

guidata(hObject, handles);

% UIWAIT makes Dialogue_box_pre_post wait for user response (see UIRESUME)

% uiwait(handles.figure1);

% --- Outputs from this function are returned to the command line.

function varargout = Dialogue_box_pre_post_OutputFcn(hObject, eventdata, handles)

% varargout cell array for returning output args (see VARARGOUT);

% hObject handle to figure

% eventdata reserved - to be defined in a future version of MATLAB

% handles structure with handles and user data (see GUIDATA)

% Get default command line output from handles structure

varargout{1} = handles.output;

% --- Executes on button press in pushbutton1.

function pushbutton1_Callback(hObject, eventdata, handles)

% hObject handle to pushbutton1 (see GCBO)

% eventdata reserved - to be defined in a future version of MATLAB

% handles structure with handles and user data (see GUIDATA)

exportInfo2Excel;

msgbox('data have been written to platform.xlsx');

%system('taskkill /F /IM EXCEL.EXE');

%%%%%%%%%%%%%%%%%%%%%%%%%%%%%%%%%%%%%%%%%%%%%%%%

| **M-file name** | Dialogue_box_pre_post.fig |
| --- | --- |
| **Type** | Figure file (design) |
| **Description** | Design file of the same GUI .m file |
| **comments** | Novel  Last modified: 18-3-2021 |

**Code:** There is no code, just a design**.**

%%%%%%%%%%%%%%%%%%%%%%%%%%%%%%%%%%%%%%%%%%%%%%%%

| **M-file name** | energy_Pcount _Gcount_iteration_To_excel |
| --- | --- |
| **Type** | Script |
| **Description** | Energy experiment. This file writes his output in the excel file named: ***Energy.xlsx*** |
| **comments** | Novel: Last modified: 16-7-2021 |

**Code:**

load preInf energy numofIter Gray_count pixel_count

energyPre=energy;

numofIterPre=numofIter;

Gray_countPre=Gray_count;

pixel_countPre=pixel_count;

load postInf energy numofIter Gray_count pixel_count

energyPost=energy;

numofIterPost=numofIter;

Gray_countPost=Gray_count;

pixel_countPost=pixel_count;

filename = 'energy.xlsx';

T1 = table(energyPre(1), energyPre(2), energyPre(3), energyPre(4), energyPre(5), energyPre(6), energyPre(7), energyPre(8),...

energyPre(9), energyPre(10), energyPre(11), energyPre(12), energyPre(13), energyPre(14), energyPre(15), energyPre(16));

T2= table(numofIterPre(1), numofIterPre(2), numofIterPre(3), numofIterPre(4), numofIterPre(5), numofIterPre(6), numofIterPre(7), numofIterPre(8),...

numofIterPre(9), numofIterPre(10), numofIterPre(11), numofIterPre(12), numofIterPre(13), numofIterPre(14), numofIterPre(15), numofIterPre(16));

T3=table(Gray_countPre(1), Gray_countPre(2), Gray_countPre(3), Gray_countPre(4), Gray_countPre(5), Gray_countPre(6), Gray_countPre(7), Gray_countPre(8),...

Gray_countPre(9), Gray_countPre(10), Gray_countPre(11), Gray_countPre(12), Gray_countPre(13), Gray_countPre(14), Gray_countPre(15), Gray_countPre(16));

T4=table(pixel_countPre(1), pixel_countPre(2), pixel_countPre(3), pixel_countPre(4), pixel_countPre(5), pixel_countPre(6), pixel_countPre(7), pixel_countPre(8),...

pixel_countPre(9), pixel_countPre(10), pixel_countPre(11), pixel_countPre(12), pixel_countPre(13), pixel_countPre(14), pixel_countPre(15), pixel_countPre(16));

writetable(T1,filename,'Sheet','pre','WriteVariableNames',false,'Range',strcat('B3:Q4'));

writetable(T2,filename,'Sheet','pre','WriteVariableNames',false,'Range',strcat('B4:Q4'));

writetable(T3,filename,'Sheet','pre','WriteVariableNames',false,'Range',strcat('B5:Q5'));

writetable(T4,filename,'Sheet','pre','WriteVariableNames',false,'Range',strcat('B6:Q6'));

%

T1 = table(energyPost(1), energyPost(2), energyPost(3), energyPost(4), energyPost(5), energyPost(6), energyPost(7), energyPost(8),...

energyPost(9), energyPost(10), energyPost(11), energyPost(12), energyPost(13), energyPost(14), energyPost(15), energyPost(16));

T2= table(numofIterPost(1), numofIterPost(2), numofIterPost(3), numofIterPost(4), numofIterPost(5), numofIterPost(6), numofIterPost(7), numofIterPost(8),...

numofIterPost(9), numofIterPost(10), numofIterPost(11), numofIterPost(12), numofIterPost(13), numofIterPost(14), numofIterPost(15), numofIterPost(16));

T3=table(Gray_countPost(1), Gray_countPost(2), Gray_countPost(3), Gray_countPost(4), Gray_countPost(5), Gray_countPost(6), Gray_countPost(7), Gray_countPost(8),...

Gray_countPost(9), Gray_countPost(10), Gray_countPost(11), Gray_countPost(12), Gray_countPost(13), Gray_countPost(14), Gray_countPost(15), Gray_countPost(16));

T4=table(pixel_countPost(1), pixel_countPost(2), pixel_countPost(3), pixel_countPost(4), pixel_countPost(5), pixel_countPost(6), pixel_countPost(7), pixel_countPost(8),...

pixel_countPost(9), pixel_countPost(10), pixel_countPost(11), pixel_countPost(12), pixel_countPost(13), pixel_countPost(14), pixel_countPost(15), pixel_countPost(16));

writetable(T1,filename,'Sheet','Post','WriteVariableNames',false,'Range',strcat('B3:Q4'));

writetable(T2,filename,'Sheet','Post','WriteVariableNames',false,'Range',strcat('B4:Q4'));

writetable(T3,filename,'Sheet','Post','WriteVariableNames',false,'Range',strcat('B5:Q5'));

writetable(T4,filename,'Sheet','Post','WriteVariableNames',false,'Range',strcat('B6:Q6'));

%

disp('Data has been written to excel sheet, check energy.xlsx in the current folder!');

%%%%%%%%%%%%%%%%%%%%%%%%%%%%%%%%%%%%%%%%%%%%%%%%

| **M-file name** | EntropySig |
| --- | --- |
| **Type** | Fun |
| **Description** | This file was written someday to compute entropy for an image using entropy equation but now it is discarded (we can remove it) |
| **comments** | Novel: Last modified: 22-4-2021 |

**Code:**

%%%%%%%%%%%%%%%%%%%%%%%%%%%%%%%%%%%%%%%%%%%%%

%% Entropy Calculation %%

%%%%%%%%%%%%%%%%%%%%%%%%%%%%%%%%%%%%%%%%%%%%%

function [en]=EntropySig(signal)

hist1 = hist(signal);

phist = hist1 ./ sum(hist1);

p=phist(phist~=0);

en = -sum(p.*log2(p))/log2(length(signal));

%%%%%%%%%%%%%%%%%%%%%%%%%%%%%%%%%%%%%%%%%%%%%%%%

| **M-file name** | exportInfo2Excel |
| --- | --- |
| **Type** | Script |
| **Description** | This script is written to export Workspace data to a predesigned excel sheet in order to save patient's information and the calculated data. This file writes in ***Platform.xlsx*** |
| **comments** | Novel: Last modified: 15-7-2021 |

**Code:**

%% This script is written to export Workspace data to a predesigned excel sheet in order to save patient's information and the caculated data

%% note this script shoudn't be executed before running

% TID_new_Region_Growing_allParametters.m script !!!

%% Get row number and name of excel sheet.

clear all

load pre AcquisitionDate2 area1Pre area2Pre AverageDivPre circularityMaxPre circularityMinPre ElongationMaxPre ...

ElongationMinPre Entro_pre ApEnPre FrameTime2 heart2lungpre HeartRate2 HighRRValue2 IntervalsAcquired2 IntervalsRejected2 LowRRValue2...

LVEFpre Manufacturer minLongDIVmaxLPre minShortDIVmaxLPre PatientAge2 PatientID PatientWeight2 PixelNumMaxPre PixelNumMinPre...

post2pre ratePre Sync_pre TIDmax TIDmin TotalBGmaxPre TotalBGminPre phasePre BoundApEnPre epsilonPre LspsilonPre ApEnPreMXMN...

Synchrony_PreMXMN Entro_PreMXMN BoundApEnPreMXMN LVEFprePhase

load num num;

filename = 'platform.xlsx';

load post AcquisitionDate area1Post area2Post AverageDivPost circularityMaxPost circularityMinPost ElongationMaxPost ...

ElongationMinPost Entro_post ApEnPost FrameTime heart2lungpost HeartRate HighRRValue IntervalsAcquired IntervalsRejected LowRRValue...

LVEFpost Manufacturer minLongDIVmaxLPost minShortDIVmaxLPost PatientAge PatientID PatientWeight PixelNumMaxPost PixelNumMinPost...

post2pre ratePost Sync_post TIDmax TIDmin TotalBGmaxPost TotalBGminPost phasePost BoundApEnPost epsilonPost LspsilonPost ...

ApEnPostMXMN Entro_PostMXMN Synchrony_PostMXMN BoundApEnPostMXMN LVEFpostPhase

%% PRE

% patient ID

T1 = table({PatientID},{' '},{' '},{PatientAge2},{datetime(AcquisitionDate2,'InputFormat','yyyyMMdd')},{LVEFpre},{LVEFprePhase}...

,Sync_pre,Synchrony_PreMXMN,Entro_pre,Entro_PreMXMN,ApEnPre,ApEnPreMXMN,heart2lungpre,AverageDivPre,minLongDIVmaxLPre,minShortDIVmaxLPre,circularityMaxPre...

,circularityMinPre,ElongationMaxPre,ElongationMinPre,HighRRValue2,IntervalsRejected2,IntervalsAcquired2,HeartRate2...

,FrameTime2,{Manufacturer},{phasePre},BoundApEnPre, BoundApEnPreMXMN,epsilonPre, LspsilonPre,TotalBGmaxPre,PixelNumMaxPre, TotalBGminPre, PixelNumMinPre,...

{' '},...

{datetime(AcquisitionDate,'InputFormat','yyyyMMdd')},{LVEFpost},{LVEFpostPhase},Sync_post, Synchrony_PostMXMN,Entro_post,...

Entro_PostMXMN,ApEnPost,ApEnPostMXMN,heart2lungpost,AverageDivPost,...

minLongDIVmaxLPost,minShortDIVmaxLPost,circularityMaxPost,circularityMinPost,ElongationMaxPost,ElongationMinPost,HighRRValue,...

IntervalsRejected,IntervalsAcquired,HeartRate,FrameTime,{Manufacturer},phasePost,{' '},post2pre,TIDmax,TIDmin,BoundApEnPost,BoundApEnPostMXMN,...

epsilonPost, LspsilonPost, TotalBGmaxPost,PixelNumMaxPost, TotalBGminPost, PixelNumMinPost);

%system('taskkill /F /IM EXCEL.EXE');

writetable(T1,filename,'Sheet','Sheet1','WriteVariableNames',false,'Range',strcat('A',num2str(num)));

% % age and date (post)

% writetable(T2,filename,'Sheet','Sheet1','WriteVariableNames',false,'Range',strcat('D',num2str(num),':','F',num2str(num)));

% %system('taskkill /F /IM EXCEL.EXE');

%

% % Rest of data (post)

% writetable(T3,filename,'Sheet','Sheet1','WriteVariableNames',false,'Range',strcat('G',num2str(num),':','X',num2str(num)));

%system('taskkill /F /IM EXCEL.EXE');

%% POST

% date (post)

%T2 = table({datetime(AcquisitionDate,'InputFormat','yyyyMMdd')},{LVEFpost},Sync_post,Entro_post,ApEnPost,heart2lungpost,AverageDivPost,minLongDIVmaxLPost,minShortDIVmaxLPost,circularityMaxPost,circularityMinPost,ElongationMaxPost,ElongationMinPost,HighRRValue,IntervalsRejected,IntervalsAcquired,HeartRate,FrameTime,{Manufacturer},phasePost,{' '},post2pre,TIDmax,TIDmin);

%T3 = table(Sync_post,Entro_post,ApEnPost,heart2lungpost,AverageDivPost,minLongDIVmaxLPost,minShortDIVmaxLPost,circularityMaxPost,circularityMinPost,ElongationMaxPost,ElongationMinPost,HighRRValue,IntervalsRejected,IntervalsAcquired,HeartRate,FrameTime,{Manufacturer},phasePost);

% writetable(T3,filename,'Sheet','Sheet1','WriteVariableNames',false,'Range',strcat('AB',num2str(num),':','AS',num2str(num)));

% %system('taskkill /F /IM EXCEL.EXE');

%

% %% A2/A1 and TID

%T4 = table(post2pre,TIDmax,TIDmin);

%writetable(T2,filename,'Sheet','Sheet1','WriteVariableNames',false,'Range',strcat('Z',num2str(num),':','AW',num2str(num)));

%system('taskkill /F /IM EXCEL.EXE');

%% increase num for the next patient

num = num+1;

save num num;

%% close excel process

%system('taskkill /F /IM EXCEL.EXE');

%% clear variables

clear all

disp('Data has been written to excel sheet, check platform.xlsx in the current folder!');

%% end :)

%%%%%%%%%%%%%%%%%%%%%%%%%%%%%%%%%%%%%%%%%%%%%%%%

| **M-file name** | fit_ellipse |
| --- | --- |
| **Type** | Fun |
| **Description** | Finds the best fit to an ellipse for the given set of points. |
| **comments** | Open source(245) |

**Code:**

function ellipse_t = fit_ellipse( x,y,axis_handle )

%

% fit_ellipse - finds the best fit to an ellipse for the given set of points.

%

% Format: ellipse_t = fit_ellipse( x,y,axis_handle )

%

% Input: x,y - a set of points in 2 column vectors. AT LEAST 5 points are needed !

% axis_handle - optional. a handle to an axis, at which the estimated ellipse

% will be drawn along with it's axes

%

% Output: ellipse_t - structure that defines the best fit to an ellipse

% a - sub axis (radius) of the X axis of the non-tilt ellipse

% b - sub axis (radius) of the Y axis of the non-tilt ellipse

% phi - orientation in radians of the ellipse (tilt)

% X0 - center at the X axis of the non-tilt ellipse

% Y0 - center at the Y axis of the non-tilt ellipse

% X0_in - center at the X axis of the tilted ellipse

% Y0_in - center at the Y axis of the tilted ellipse

% long_axis - size of the long axis of the ellipse

% short_axis - size of the short axis of the ellipse

% status - status of detection of an ellipse

%

% Note: if an ellipse was not detected (but a parabola or hyperbola), then

% an empty structure is returned

% =====================================================================================

% Ellipse Fit using Least Squares criterion

% =====================================================================================

% We will try to fit the best ellipse to the given measurements. the mathematical

% representation of use will be the CONIC Equation of the Ellipse which is:

%

% Ellipse = a*x^2 + b*x*y + c*y^2 + d*x + e*y + f = 0

%

% The fit-estimation method of use is the Least Squares method (without any weights)

% The estimator is extracted from the following equations:

%

% g(x,y;A) := a*x^2 + b*x*y + c*y^2 + d*x + e*y = f

%

% where:

% A - is the vector of parameters to be estimated (a,b,c,d,e)

% x,y - is a single measurement

%

% We will define the cost function to be:

%

% Cost(A) := (g_c(x_c,y_c;A)-f_c)'*(g_c(x_c,y_c;A)-f_c)

% = (X*A+f_c)'*(X*A+f_c)

% = A'*X'*X*A + 2*f_c'*X*A + N*f^2

%

% where:

% g_c(x_c,y_c;A) - vector function of ALL the measurements

% each element of g_c() is g(x,y;A)

% X - a matrix of the form: [x_c.^2, x_c.*y_c, y_c.^2, x_c, y_c ]

% f_c - is actually defined as ones(length(f),1)*f

%

% Derivation of the Cost function with respect to the vector of parameters "A" yields:

%

% A'*X'*X = -f_c'*X = -f*ones(1,length(f_c))*X = -f*sum(X)

%

% Which yields the estimator:

%

% ~~~~~~~~~~~~~~~~~~~~~~~~~~~~~~~~~~~~~~~~~~~~~~~~~~~~~~~~~~~~~~~~~~~~~~~~~~~~

% | A_least_squares = -f*sum(X)/(X'*X) ->(normalize by -f) = sum(X)/(X'*X) |

% ~~~~~~~~~~~~~~~~~~~~~~~~~~~~~~~~~~~~~~~~~~~~~~~~~~~~~~~~~~~~~~~~~~~~~~~~~~~~

%

% (We will normalize the variables by (-f) since "f" is unknown and can be accounted for later on)

%

% NOW, all that is left to do is to extract the parameters from the Conic Equation.

% We will deal the vector A into the variables: (A,B,C,D,E) and assume F = -1;

%

% Recall the conic representation of an ellipse:

%

% A*x^2 + B*x*y + C*y^2 + D*x + E*y + F = 0

%

% We will check if the ellipse has a tilt (=orientation). The orientation is present

% if the coefficient of the term "x*y" is not zero. If so, we first need to remove the

% tilt of the ellipse.

%

% If the parameter "B" is not equal to zero, then we have an orientation (tilt) to the ellipse.

% we will remove the tilt of the ellipse so as to remain with a conic representation of an

% ellipse without a tilt, for which the math is more simple:

%

% Non tilt conic rep.: A`*x^2 + C`*y^2 + D`*x + E`*y + F` = 0

%

% We will remove the orientation using the following substitution:

%

% Replace x with cx+sy and y with -sx+cy such that the conic representation is:

%

% A(cx+sy)^2 + B(cx+sy)(-sx+cy) + C(-sx+cy)^2 + D(cx+sy) + E(-sx+cy) + F = 0

%

% where: c = cos(phi) , s = sin(phi)

%

% and simplify...

%

% x^2(A*c^2 - Bcs + Cs^2) + xy(2A*cs +(c^2-s^2)B -2Ccs) + ...

% y^2(As^2 + Bcs + Cc^2) + x(Dc-Es) + y(Ds+Ec) + F = 0

%

% The orientation is easily found by the condition of (B_new=0) which results in:

%

% 2A*cs +(c^2-s^2)B -2Ccs = 0 ==> phi = 1/2 * atan( b/(c-a) )

%

% Now the constants c=cos(phi) and s=sin(phi) can be found, and from them

% all the other constants A`,C`,D`,E` can be found.

%

% A` = A*c^2 - B*c*s + C*s^2 D` = D*c-E*s

% B` = 2*A*c*s +(c^2-s^2)*B -2*C*c*s = 0 E` = D*s+E*c

% C` = A*s^2 + B*c*s + C*c^2

%

% Next, we want the representation of the non-tilted ellipse to be as:

%

% Ellipse = ( (X-X0)/a )^2 + ( (Y-Y0)/b )^2 = 1

%

% where: (X0,Y0) is the center of the ellipse

% a,b are the ellipse "radiuses" (or sub-axis)

%

% Using a square completion method we will define:

%

% F`` = -F` + (D`^2)/(4*A`) + (E`^2)/(4*C`)

%

% Such that: a`*(X-X0)^2 = A`(X^2 + X*D`/A` + (D`/(2*A`))^2 )

% c`*(Y-Y0)^2 = C`(Y^2 + Y*E`/C` + (E`/(2*C`))^2 )

%

% which yields the transformations:

%

% X0 = -D`/(2*A`)

% Y0 = -E`/(2*C`)

% a = sqrt( abs( F``/A` ) )

% b = sqrt( abs( F``/C` ) )

%

% And finally we can define the remaining parameters:

%

% long_axis = 2 * max( a,b )

% short_axis = 2 * min( a,b )

% Orientation = phi

%

%

% initialize

orientation_tolerance = 1e-3;

% empty warning stack

warning( '' );

% prepare vectors, must be column vectors

x = x(:);

y = y(:);

% remove bias of the ellipse - to make matrix inversion more accurate. (will be added later on).

mean_x = mean(x);

mean_y = mean(y);

x = x-mean_x;

y = y-mean_y;

% the estimation for the conic equation of the ellipse

X = [x.^2, x.*y, y.^2, x, y ];

a = sum(X)/(X'*X);

% check for warnings

if ~isempty( lastwarn )

disp( 'stopped because of a warning regarding matrix inversion' );

ellipse_t = [];

return

end

% extract parameters from the conic equation

[a,b,c,d,e] = deal( a(1),a(2),a(3),a(4),a(5) );

% remove the orientation from the ellipse

if ( min(abs(b/a),abs(b/c)) > orientation_tolerance )

orientation_rad = 1/2 * atan( b/(c-a) );

cos_phi = cos( orientation_rad );

sin_phi = sin( orientation_rad );

[a,b,c,d,e] = deal(...

a*cos_phi^2 - b*cos_phi*sin_phi + c*sin_phi^2,...

0,...

a*sin_phi^2 + b*cos_phi*sin_phi + c*cos_phi^2,...

d*cos_phi - e*sin_phi,...

d*sin_phi + e*cos_phi );

[mean_x,mean_y] = deal( ...

cos_phi*mean_x - sin_phi*mean_y,...

sin_phi*mean_x + cos_phi*mean_y );

else

orientation_rad = 0;

cos_phi = cos( orientation_rad );

sin_phi = sin( orientation_rad );

end

% check if conic equation represents an ellipse

test = a*c;

switch (1)

case (test>0), status = '';

case (test==0), status = 'Parabola found'; warning( 'fit_ellipse: Did not locate an ellipse' );

case (test<0), status = 'Hyperbola found'; warning( 'fit_ellipse: Did not locate an ellipse' );

end

% if we found an ellipse return it's data

if (test>0)

% make sure coefficients are positive as required

if (a<0), [a,c,d,e] = deal( -a,-c,-d,-e ); end

% final ellipse parameters

X0 = mean_x - d/2/a;

Y0 = mean_y - e/2/c;

F = 1 + (d^2)/(4*a) + (e^2)/(4*c);

[a,b] = deal( sqrt( F/a ),sqrt( F/c ) );

long_axis = 2*max(a,b);

short_axis = 2*min(a,b);

% rotate the axes backwards to find the center point of the original TILTED ellipse

R = [ cos_phi sin_phi; -sin_phi cos_phi ];

P_in = R * [X0;Y0];

X0_in = P_in(1);

Y0_in = P_in(2);

% pack ellipse into a structure

ellipse_t = struct( ...

'a',a,...

'b',b,...

'phi',orientation_rad,...

'X0',X0,...

'Y0',Y0,...

'X0_in',X0_in,...

'Y0_in',Y0_in,...

'long_axis',long_axis,...

'short_axis',short_axis,...

'status','' );

else

% report an empty structure

ellipse_t = struct( ...

'a',[],...

'b',[],...

'phi',[],...

'X0',[],...

'Y0',[],...

'X0_in',[],...

'Y0_in',[],...

'long_axis',[],...

'short_axis',[],...

'status',status );

end

% check if we need to plot an ellipse with it's axes.

if (nargin>2) & ~isempty( axis_handle ) & (test>0)

% rotation matrix to rotate the axes with respect to an angle phi

R = [ cos_phi sin_phi; -sin_phi cos_phi ];

% the axes

ver_line = [ [X0 X0]; Y0+b*[-1 1] ];

horz_line = [ X0+a*[-1 1]; [Y0 Y0] ];

new_ver_line = R*ver_line;

new_horz_line = R*horz_line;

% the ellipse

theta_r = linspace(0,2*pi);

ellipse_x_r = X0 + a*cos( theta_r );

ellipse_y_r = Y0 + b*sin( theta_r );

rotated_ellipse = R * [ellipse_x_r;ellipse_y_r];

% draw

hold_state = get( axis_handle,'NextPlot' );

set( axis_handle,'NextPlot','add' );

plot( new_ver_line(1,:),new_ver_line(2,:),'r' );

plot( new_horz_line(1,:),new_horz_line(2,:),'r' );

plot( rotated_ellipse(1,:),rotated_ellipse(2,:),'r' );

set( axis_handle,'NextPlot',hold_state );

end

%%%%%%%%%%%%%%%%%%%%%%%%%%%%%%%%%%%%%%%%%%%%%%%%

| **M-file name** | Image_ Entropy_ Experiment.m |
| --- | --- |
| **Type** | Script |
| **Description** | Experiment to compute the entropy, ApEn, Bounded ApEn and Synchrony using salt and pepper noise under different noise range. This file writes in ImageNoiseEntropyExperiment.xlsx |
| **comments** | Novel: Written 25-5-2021  Last Modified: 8-9-2021 |

**Code:**

%%

Experiment to compute the entropy, ApEn, Bounded ApEn and Synchrony using salt and pepper noise under different noise range.

%%

m_val=2; r_val=1;

x = inputdlg('Pick Post image:',...

'Define Image', [1]);

name= (x{:}); % image number

imvol = squeeze(dicomread(name));

info = dicominfo(name);

save info info;

for i=1:1:info.NumberOfFrames

imvol(:,:,i)=medfilt2(imvol(:,:,i),[3 3]);%

end

figure,imshow(sum(imvol,3),[]); title('Select seed point');

colormap jet;colorbar

sp = ginput(1);

figure

[a,B, C, D,min_img2,F,imvolmn2,bw1, AA, AB, AC, AD, AE, AF,AG, AH, AI, aj, sp]=max_min_MUGA3(imvol,0,sp,[],[]);

bw1=(bw1);

for i=1:1:info.NumberOfFrames

imvol1(:,:,i)=uint16(bw1).*imvol(:,:,i);%

end

close

imshow(sum(imvol1,3),[]);colormap jet;colorbar

I1=imvol1;

f1 = @(x) imnoise(x,'salt & pepper',0.01);

f2 = @(x) imnoise(x,'salt & pepper',0.02);

f3 = @(x) imnoise(x,'salt & pepper',0.03);

f4 = @(x) imnoise(x,'salt & pepper',0.04);

f5 = @(x) imnoise(x,'salt & pepper',0.05);

f6 = @(x) imnoise(x,'salt & pepper',0.06);

for i=1:16

I2(:,:,i)=roifilt2(I1(:,:,i),bw1,f1);

I3(:,:,i)=roifilt2(I1(:,:,i),bw1,f2);

I4(:,:,i)=roifilt2(I1(:,:,i),bw1,f3);

I5(:,:,i)=roifilt2(I1(:,:,i),bw1,f4);

I6(:,:,i)=roifilt2(I1(:,:,i),bw1,f5);

I7(:,:,i)=roifilt2(I1(:,:,i),bw1,f6);

end

figure

subplot(2,3,1),imshow(sum(I2,3),[0 800]);title('noise=0.01');colormap jet;colorbar

subplot(2,3,2),imshow(sum(I3,3),[0 800]);title('noise=0.02');colormap jet;colorbar

subplot(2,3,3),imshow(sum(I4,3),[0 800]);title('noise=0.03');colormap jet;colorbar

subplot(2,3,4),imshow(sum(I5,3),[0 800]);title('noise=0.04');colormap jet;colorbar

subplot(2,3,5),imshow(sum(I6,3),[0 800]);title('noise=0.05');colormap jet;colorbar

subplot(2,3,6),imshow(sum(I7,3),[0 800]);title('noise=0.06');colormap jet;colorbar

I11=sum(I1,3);

I12=sum(I2,3);

I13=sum(I3,3);

I14=sum(I4,3);

I15=sum(I5,3);

I16=sum(I6,3);

I17=sum(I7,3);

s1=(I11(bw1));

s2=(I12(bw1));

s3=(I13(bw1));

s4=(I14(bw1));

s5=(I15(bw1));

s6=(I16(bw1));

s7=(I17(bw1));

%[,Sync_post]=computeEntropySynchrony(s1,-1,-1,0)

[Ent1 syn1]=computeEntropySynchronyNo(I1);

[Ent2 syn2]=computeEntropySynchronyNo(I2);

[Ent3 syn3]=computeEntropySynchronyNo(I3);

[Ent4 syn4]=computeEntropySynchronyNo(I4);

[Ent5 syn5]=computeEntropySynchronyNo(I5);

[Ent6 syn6]=computeEntropySynchronyNo(I6);

[Ent7 syn7]=computeEntropySynchronyNo(I7);

ApEnPost1= ApEn_slow(s1, m_val,r_val*std(s1));

ApEnPost2= ApEn_slow(s2, m_val,r_val*std(s2));

ApEnPost3= ApEn_slow(s3, m_val,r_val*std(s3));

ApEnPost4= ApEn_slow(s4, m_val,r_val*std(s4));

ApEnPost5= ApEn_slow(s5, m_val,r_val*std(s5));

ApEnPost6= ApEn_slow(s6, m_val,r_val*std(s6));

ApEnPost7= ApEn_slow(s7, m_val,r_val*std(s7));

[BoundApEnPost1,epsilonPost1,LspsilonPost1]=BoundedProcess(s1);

[BoundApEnPost2,epsilonPost2,LspsilonPost2]=BoundedProcess(s2);

[BoundApEnPost3,epsilonPost3,LspsilonPost3]=BoundedProcess(s3);

[BoundApEnPost4,epsilonPost4,LspsilonPost4]=BoundedProcess(s4);

[BoundApEnPost5,epsilonPost5,LspsilonPost5]=BoundedProcess(s5);

[BoundApEnPost6,epsilonPost6,LspsilonPost6]=BoundedProcess(s6);

[BoundApEnPost7,epsilonPost7,LspsilonPost7]=BoundedProcess(s7);

[Ent1 Ent2 Ent3 Ent4 Ent5 Ent6 Ent7]

[ApEnPost1 ApEnPost2 ApEnPost3 ApEnPost4 ApEnPost5 ApEnPost6 ApEnPost7]

[BoundApEnPost1 BoundApEnPost2 BoundApEnPost3 BoundApEnPost4 BoundApEnPost5 BoundApEnPost6 BoundApEnPost7]

[syn1 syn2 syn3 syn4 syn5 syn6 syn7]

filename = 'ImageNoiseEntropyExperiment.xlsx';

T1 = table({Ent1},{Ent2},{Ent3},{Ent4},{Ent5},{Ent6},{Ent7});

%system('taskkill /F /IM EXCEL.EXE');

writetable(T1,filename,'Sheet','Sheet1','WriteVariableNames',false,'Range',strcat('B2:H2'));

T1 = table({ApEnPost1},{ApEnPost2},{ApEnPost3},{ApEnPost4},{ApEnPost5},{ApEnPost6},{ApEnPost7});

%system('taskkill /F /IM EXCEL.EXE');

writetable(T1,filename,'Sheet','Sheet1','WriteVariableNames',false,'Range',strcat('B3:H3'));

T1 = table({BoundApEnPost1},{BoundApEnPost2},{BoundApEnPost3},{BoundApEnPost4},{BoundApEnPost5},{BoundApEnPost6},{BoundApEnPost7});

%system('taskkill /F /IM EXCEL.EXE');

writetable(T1,filename,'Sheet','Sheet1','WriteVariableNames',false,'Range',strcat('B4:H4'));

T1 = table({syn1},{syn2},{syn3},{syn4},{syn5},{syn6},{syn7});

%system('taskkill /F /IM EXCEL.EXE');

writetable(T1,filename,'Sheet','Sheet1','WriteVariableNames',false,'Range',strcat('B5:H4'));

disp('Data has been written to excel sheet, check platform.xlsx in the current folder!');

%%

I1=imvol1;

f1 = @(x) imnoise(x,'salt & pepper',0.03);

f2 = @(x) imnoise(x,'salt & pepper',0.06);

f3 = @(x) imnoise(x,'salt & pepper',0.09);

f4 = @(x) imnoise(x,'salt & pepper',0.12);

f5 = @(x) imnoise(x,'salt & pepper',0.15);

f6 = @(x) imnoise(x,'salt & pepper',0.18);

for i=1:16

I2(:,:,i)=roifilt2(I1(:,:,i),bw1,f1);

I3(:,:,i)=roifilt2(I1(:,:,i),bw1,f2);

I4(:,:,i)=roifilt2(I1(:,:,i),bw1,f3);

I5(:,:,i)=roifilt2(I1(:,:,i),bw1,f4);

I6(:,:,i)=roifilt2(I1(:,:,i),bw1,f5);

I7(:,:,i)=roifilt2(I1(:,:,i),bw1,f6);

end

figure

subplot(2,3,1),imshow(sum(I2,3),[0 800]);title('noise=0.03');colormap jet;colorbar

subplot(2,3,2),imshow(sum(I3,3),[0 800]);title('noise=0.06');colormap jet;colorbar

subplot(2,3,3),imshow(sum(I4,3),[0 800]);title('noise=0.09');colormap jet;colorbar

subplot(2,3,4),imshow(sum(I5,3),[0 800]);title('noise=0.12');colormap jet;colorbar

subplot(2,3,5),imshow(sum(I6,3),[0 800]);title('noise=0.15');colormap jet;colorbar

subplot(2,3,6),imshow(sum(I7,3),[0 800]);title('noise=0.18');colormap jet;colorbar

I11=sum(I1,3);

I12=sum(I2,3);

I13=sum(I3,3);

I14=sum(I4,3);

I15=sum(I5,3);

I16=sum(I6,3);

I17=sum(I7,3);

s1=(I11(bw1));

s2=(I12(bw1));

s3=(I13(bw1));

s4=(I14(bw1));

s5=(I15(bw1));

s6=(I16(bw1));

s7=(I17(bw1));

%[,Sync_post]=computeEntropySynchrony(s1,-1,-1,0)

[Ent1 syn1]=computeEntropySynchronyNo(I1);

[Ent2 syn2]=computeEntropySynchronyNo(I2);

[Ent3 syn3]=computeEntropySynchronyNo(I3);

[Ent4 syn4]=computeEntropySynchronyNo(I4);

[Ent5 syn5]=computeEntropySynchronyNo(I5);

[Ent6 syn6]=computeEntropySynchronyNo(I6);

[Ent7 syn7]=computeEntropySynchronyNo(I7);

ApEnPost1= ApEn_slow(s1, m_val,r_val*std(s1));

ApEnPost2= ApEn_slow(s2, m_val,r_val*std(s2));

ApEnPost3= ApEn_slow(s3, m_val,r_val*std(s3));

ApEnPost4= ApEn_slow(s4, m_val,r_val*std(s4));

ApEnPost5= ApEn_slow(s5, m_val,r_val*std(s5));

ApEnPost6= ApEn_slow(s6, m_val,r_val*std(s6));

ApEnPost7= ApEn_slow(s7, m_val,r_val*std(s7));

[BoundApEnPost1,epsilonPost1,LspsilonPost1]=BoundedProcess(s1);

[BoundApEnPost2,epsilonPost2,LspsilonPost2]=BoundedProcess(s2);

[BoundApEnPost3,epsilonPost3,LspsilonPost3]=BoundedProcess(s3);

[BoundApEnPost4,epsilonPost4,LspsilonPost4]=BoundedProcess(s4);

[BoundApEnPost5,epsilonPost5,LspsilonPost5]=BoundedProcess(s5);

[BoundApEnPost6,epsilonPost6,LspsilonPost6]=BoundedProcess(s6);

[BoundApEnPost7,epsilonPost7,LspsilonPost7]=BoundedProcess(s7);

[Ent1 Ent2 Ent3 Ent4 Ent5 Ent6 Ent7]

[ApEnPost1 ApEnPost2 ApEnPost3 ApEnPost4 ApEnPost5 ApEnPost6 ApEnPost7]

[BoundApEnPost1 BoundApEnPost2 BoundApEnPost3 BoundApEnPost4 BoundApEnPost5 BoundApEnPost6 BoundApEnPost7]

[syn1 syn2 syn3 syn4 syn5 syn6 syn7]

filename = 'ImageNoiseEntropyExperiment.xlsx';

T1 = table({Ent1},{Ent2},{Ent3},{Ent4},{Ent5},{Ent6},{Ent7});

%system('taskkill /F /IM EXCEL.EXE');

writetable(T1,filename,'Sheet','Sheet1','WriteVariableNames',false,'Range',strcat('B9:H9'));

T1 = table({ApEnPost1},{ApEnPost2},{ApEnPost3},{ApEnPost4},{ApEnPost5},{ApEnPost6},{ApEnPost7});

%system('taskkill /F /IM EXCEL.EXE');

writetable(T1,filename,'Sheet','Sheet1','WriteVariableNames',false,'Range',strcat('B10:H10'));

T1 = table({BoundApEnPost1},{BoundApEnPost2},{BoundApEnPost3},{BoundApEnPost4},{BoundApEnPost5},{BoundApEnPost6},{BoundApEnPost7});

%system('taskkill /F /IM EXCEL.EXE');

writetable(T1,filename,'Sheet','Sheet1','WriteVariableNames',false,'Range',strcat('B11:H11'));

T1 = table({syn1},{syn2},{syn3},{syn4},{syn5},{syn6},{syn7});

%system('taskkill /F /IM EXCEL.EXE');

writetable(T1,filename,'Sheet','Sheet1','WriteVariableNames',false,'Range',strcat('B12:H12'));

disp('Data has been written to excel sheet, check platform.xlsx in the current folder!');

%%%%%%%%%%%%%%%%%%%%%%%%%%%%%%%%%%%%%%%%%%%%%%%%

| **M-file name** | Image_ Entropy_ ExperimentPoisson.m |
| --- | --- |
| **Type** | Script |
| **Description** | Experiment to compute the entropy, ApEn, Bounded ApEn and Synchrony using **poisson** noise under different noise range.  This file writes in ImageNoise EntropyExperimentPoisson.xlsx |
| **comments** | Novel: Written: 27-5-2021  Last modified: 8-9-2021 |

**Code:**

%%

%Experiment to compute the entropy, ApEn, Bounded ApEn and Synchrony %using poisson %noise under different noise range.

%%

m_val=2; r_val=1;

x = inputdlg('Pick Post image:',...

'Define Image', [1]);

name= (x{:}); % image number

imvol = squeeze(dicomread(name));

info = dicominfo(name);

save info info;

for i=1:1:info.NumberOfFrames

imvol(:,:,i)=medfilt2(imvol(:,:,i),[5 5]);%

end

figure,imshow(sum(imvol,3),[]); title('Select seed point');

colormap jet;colorbar

sp = ginput(1);

figure

[a,B, C, D,min_img2,F,imvolmn2,bw1, AA, AB, AC, AD, AE, AF,AG, AH, AI, aj, sp]=max_min_MUGA3(imvol,0,sp,[],[]);

bw1=(bw1);

for i=1:1:info.NumberOfFrames

imvol1(:,:,i)=uint16(bw1).*imvol(:,:,i);%

end

close

imshow(sum(imvol1,3),[]);colormap jet;colorbar

I1=imvol1;

I2=uint16(imvol1*1.11);

I3=uint16(imvol1*1.15);

I4=uint16(imvol1*1.2);

I5=uint16(imvol1*1.25);

I6=uint16(imvol1*1.3);

I7=uint16(imvol1*1.35);

f1 = @(x) imnoise(x,'poisson');

for i=1:16

I2(:,:,i)=roifilt2(I2(:,:,i),bw1,f1);

I3(:,:,i)=roifilt2(I3(:,:,i),bw1,f1);

I4(:,:,i)=roifilt2(I4(:,:,i),bw1,f1);

I5(:,:,i)=roifilt2(I5(:,:,i),bw1,f1);

I6(:,:,i)=roifilt2(I6(:,:,i),bw1,f1);

I7(:,:,i)=roifilt2(I7(:,:,i),bw1,f1);

end

figure

subplot(2,3,1),imshow(sum(I2,3),[0 800]);title('noise level 1');colormap jet;colorbar

subplot(2,3,2),imshow(sum(I3,3),[0 800]);title('noise level 2');colormap jet;colorbar

subplot(2,3,3),imshow(sum(I4,3),[0 800]);title('noise level 3');colormap jet;colorbar

subplot(2,3,4),imshow(sum(I5,3),[0 800]);title('noise level 4');colormap jet;colorbar

subplot(2,3,5),imshow(sum(I6,3),[0 800]);title('noise level 5');colormap jet;colorbar

subplot(2,3,6),imshow(sum(I7,3),[0 800]);title('noise level 6');colormap jet;colorbar

I11=sum(I1,3);

I12=sum(I2,3);

I13=sum(I3,3);

I14=sum(I4,3);

I15=sum(I5,3);

I16=sum(I6,3);

I17=sum(I7,3);

s1=(I11(bw1));

s2=(I12(bw1));

s3=(I13(bw1));

s4=(I14(bw1));

s5=(I15(bw1));

s6=(I16(bw1));

s7=(I17(bw1));

%[,Sync_post]=computeEntropySynchrony(s1,-1,-1,0)

[Ent1 syn1]=computeEntropySynchronyNo(I1);

[Ent2 syn2]=computeEntropySynchronyNo(I2);

[Ent3 syn3]=computeEntropySynchronyNo(I3);

[Ent4 syn4]=computeEntropySynchronyNo(I4);

[Ent5 syn5]=computeEntropySynchronyNo(I5);

[Ent6 syn6]=computeEntropySynchronyNo(I6);

[Ent7 syn7]=computeEntropySynchronyNo(I7);

ApEnPost1= ApEn_slow(s1, m_val,r_val*std(s1));

ApEnPost2= ApEn_slow(s2, m_val,r_val*std(s2));

ApEnPost3= ApEn_slow(s3, m_val,r_val*std(s3));

ApEnPost4= ApEn_slow(s4, m_val,r_val*std(s4));

ApEnPost5= ApEn_slow(s5, m_val,r_val*std(s5));

ApEnPost6= ApEn_slow(s6, m_val,r_val*std(s6));

ApEnPost7= ApEn_slow(s7, m_val,r_val*std(s7));

[BoundApEnPost1,epsilonPost1,LspsilonPost1]=BoundedProcess(s1);

[BoundApEnPost2,epsilonPost2,LspsilonPost2]=BoundedProcess(s2);

[BoundApEnPost3,epsilonPost3,LspsilonPost3]=BoundedProcess(s3);

[BoundApEnPost4,epsilonPost4,LspsilonPost4]=BoundedProcess(s4);

[BoundApEnPost5,epsilonPost5,LspsilonPost5]=BoundedProcess(s5);

[BoundApEnPost6,epsilonPost6,LspsilonPost6]=BoundedProcess(s6);

[BoundApEnPost7,epsilonPost7,LspsilonPost7]=BoundedProcess(s7);

[Ent1 Ent2 Ent3 Ent4 Ent5 Ent6 Ent7]

[ApEnPost1 ApEnPost2 ApEnPost3 ApEnPost4 ApEnPost5 ApEnPost6 ApEnPost7]

[BoundApEnPost1 BoundApEnPost2 BoundApEnPost3 BoundApEnPost4 BoundApEnPost5 BoundApEnPost6 BoundApEnPost7]

[syn1 syn2 syn3 syn4 syn5 syn6 syn7]

filename = 'ImageNoiseEntropyExperimentPoisson.xlsx';

T1 = table({Ent1},{Ent2},{Ent3},{Ent4},{Ent5},{Ent6},{Ent7});

%system('taskkill /F /IM EXCEL.EXE');

writetable(T1,filename,'Sheet','Sheet1','WriteVariableNames',false,'Range',strcat('B2:H2'));

T1 = table({ApEnPost1},{ApEnPost2},{ApEnPost3},{ApEnPost4},{ApEnPost5},{ApEnPost6},{ApEnPost7});

%system('taskkill /F /IM EXCEL.EXE');

writetable(T1,filename,'Sheet','Sheet1','WriteVariableNames',false,'Range',strcat('B3:H3'));

T1 = table({BoundApEnPost1},{BoundApEnPost2},{BoundApEnPost3},{BoundApEnPost4},{BoundApEnPost5},{BoundApEnPost6},{BoundApEnPost7});

%system('taskkill /F /IM EXCEL.EXE');

writetable(T1,filename,'Sheet','Sheet1','WriteVariableNames',false,'Range',strcat('B4:H4'));

T1 = table({syn1},{syn2},{syn3},{syn4},{syn5},{syn6},{syn7});

%system('taskkill /F /IM EXCEL.EXE');

writetable(T1,filename,'Sheet','Sheet1','WriteVariableNames',false,'Range',strcat('B5:H4'));

disp('Data has been written to excel sheet, check platform.xlsx in the current folder!');

%%%%%%%%%%%%%%%%%%%%%%%%%%%%%%%%%%%%%%%%%%%%%%%%

| **M-file name** | LVtimeActivityCurve.m |
| --- | --- |
| **Type** | Fun |
| **Description** | Function to compute and plot the LVEF curve. |
| **comments** | Novel: Written then modified: 2-6-2021  Last modified: 8-9-2021 |

**Code:**

function [mx1,mn1,indMax,indMin,weight]= LVtimeActivityCurve(imvolx,z,z2,z3,test)

% this function is to draw the LV time activity curve %

load info info

FrameTime=info.GatedInformationSequence.Item_1.DataInformationSequence.Item_1.FrameTime;

x=FrameTime:FrameTime:16*FrameTime;

y=(zeros(1,info.NumberOfFrames));

%for i=1:info.NumberOfFrames

y(1:info.NumberOfFrames)=sum(sum(imvolx(:,:,1:info.NumberOfFrames)));

y(12)=mean(y(11:14));

y(13)=mean(y(12:14));

mx1=max((y));

mx1=mx1(1);

mn1=min((y));

mn1=mn1(1);

% y1=y;

% y1(y==mn1)=[];

% if ~isempty(z3)

% mn2=min((y1));

% mn2=mn2(1);

% mn1p=mn1;

% mn1=mn1+mn2;

% else

% mn1p=mn1;

% end

indMax=find(mx1==y);

indMin=find(mn1==y);

%sum(sum(imvolx(:,:,indMax)))

%sum(sum(imvolx(:,:,indMin)))

if (z2==0 | z2==2)

else

imvolx=z2;

y(1:info.NumberOfFrames)=sum(sum(imvolx(:,:,1:info.NumberOfFrames)));

y(12)=mean(y(11:14));

y(13)=mean(y(12:14));

mx1=y(indMax);

mx1=mx1(1);

mn1=y(indMin);

mn1=mn1(1);

y1=y;

y1(y==mn1)=[];

if ~isempty(z3)

mn2=min((y1));

mn2=mn2(1);

mn1=mn1+mn2;

end

%sum(sum(imvolx(:,:,indMax)))

%sum(sum(imvolx(:,:,indMin)))

end

%

yy = smooth(y);

if (z2==0 & (test~=1))

%plot(x,y,'-*b');

%hold on;

plot(x,yy,'-*r');

if (z==1)

title('Post LV Time activity curve');

else

title('Pre LV Time activity curve');

end

mx=max(y);

mn=min(y);

axis([x(1) x(end) 0 mx]);

xlabel('Time (msec)');

ylabel('Count (count)');

%legend('Original Curve','Smoothed Curve',...

% 'Location','SW')

legend('Smoothed Curve',...

'Location','SW')

clear info

end

if (z==1)

save curvePost x y yy

elseif (z==2)

save curvePre x y yy

end

weight=zeros(1,16);

if (test==1)

val=(y-yy)/y;

for i=1:16

if val(i)>=3 weight(i)=3;

elseif val(i)>=2 weight(i)=2;

elseif val(i)>=0 weight(i)=1;

elseif val(i)<0 && val(i)>=-1 weight(i)=-1;

elseif val(i)<-1 && val(i)>=-2 weight(i)=-2;

elseif val(i)<-2 && val(i)>=-3 weight(i)=-2;

end

end

end

%%%%%%%%%%%%%%%%%%%%%%%%%%%%%%%%%%%%%%%%%%%%%%%%

| **M-file name** | mask_all.m |
| --- | --- |
| **Type** | Fun |
| **Description** | Function to compute the number of iteration of Active Contouring AC algorithm (based on the gray count of the initial ROIs of image(i.e. imvol)) then applying the AC algorithm on the imvol image to get the corresponding 16 ROIs. |
| **comments** | Novel: Written then modified in: 15-7-2021  Last modified: 8-9-2021 |

**Code:**

function All_masks=mask_all(imvol,sp1,imvol2,bw)

load maskallparam XD YD numofIter numofIterRG

maxIter=max(numofIter);

minIter=min(numofIter);

maxIterRG=max(numofIterRG);

minIterRG=min(numofIterRG);

All_masks=uint16(zeros(size(imvol)));

y=(zeros(1,16));y1=y;

y(1:16)=sum(sum(imvol2(:,:,1:16)));

y1(1)=y(1); y1(end)=y(end);

for i=2:15

y1(i)=round(((y(i-1)+y(i))+y(i+1))/3);

end

y=y1;

mx1=max((y));

mx1=mx1(1);

mn1=min((y));

mn1=mn1(1);

%% if you want to enable lookup table please uncomment these

numofIter=round(maxIter*((y-mn1)/(mx1-mn1)));

numofIter(numofIter<=11 & numofIter>7)=11;

numofIter(numofIter<=7)=minIter;

%%

sp=zeros(16,2);

sp(1,:)=sp1(1,:);% First point (max seed point) the same

for i=2:8

sp(i,2)=sp(i-1,2)-YD;% X displacement

sp(i,1)=sp(i-1,1)-XD;% Y displacement

end

for i=9:16

sp(i,2)=sp(i-1,2)+YD;% X displacement

sp(i,1)=sp(i-1,1)+XD;% Y displacement

end

for i=1:16

[a,All_masks(:,:,i),b,c,energy(i)] = regionGrowing2(im2uint8(mat2gray((imvol(:,:,i)))),3,sp(i,:),numofIter(:,i),numofIterRG(:,i));

%[a,All_masks(:,:,i),b,c] = regionGrowing2(im2uint8(mat2gray((imvol(:,:,i)))),3,sp,8);

end

load prepost1 tag11

if (tag11==0)

save energyPre energy numofIter

else

save energyPost energy numofIter

end

energy

%%%%%%%%%%%%%%%%%%%%%%%%%%%%%%%%%%%%%%%%%%%%%%%%

| **S** | **M-file name** | **Type** | **Description** | **Novel or open-source** |
| --- | --- | --- | --- | --- |
|  | maskall3.m | Fun | The same as maskall but this function was used without plots or figures (only calculations) | Novel same as maskall |

**Code:** the same as maskall without plots and calling regiongrwoing3.m instead of regiongrowing2.m

%%%%%%%%%%%%%%%%%%%%%%%%%%%%%%%%%%%%%%%%%%%%%%%%

| **M-file name** | max_min_MUGA.m |
| --- | --- |
| **Type** | Fun |
| **Description** | This function is used to compute many parameters of the MUGA image including systole and diastole images. |
| **comments** | Novel.: Written and modified in: 27-7-2021  Last modified at: 8-9-2021 |

**Code:**

function [All_masks,smoothedPoly, smoothedPoly1, max_img,min_img,imvolmx,imvolmn, bw, bw1, area1, area2, rate,minLongDIVmaxL, minShortDIVmaxL,AverageDiv, circularityMax, circularityMin,ElongationMax,ElongationMin,sp]=max_min_MUGA(Imag)

load maskallparam numofIter numofIterRG

itrMx=max(numofIter);

itrMxRG=max(numofIterRG);

%x = inputdlg('Pick first image:',...

% 'Define Image', [1]);

%name= (x{:}); % image number

imvol = Imag;

%info = dicominfo(name);

%save info info;

[Y Z O] = size(imvol);

% for i=1:O

% mean_val(i)=mean(mean(imvol(:,:,i)));

% end

% mx_val=max(max(mean_val));

% mn_val=min(min(mean_val));

% ind_max=find(mx_val==mean_val)

% ind_min=find(mn_val==mean_val)

% max_img=imvol(:,:,ind_max);

% min_img=imvol(:,:,ind_min);

%%

%mean_val(ind_max)=-1;

%mx_val=max(max(mean_val));

%mean_val(ind_max)=1000;

%mean_val(ind_min)=1000;

%mn_val=min(min(mean_val));

% if(ind_min==16)

% ind_min=15;

% end

% if(ind_max==16)

% ind_max=15;

%

% end

%% compute max min based on ROI

%imvolx=sum(imvol,3);

imvolx=sum(imvol,3);

%figure(20),

mb=msgbox("Pick a centered seed in this Image","Seed Point");

waitfor(mb);

%imshow(im2uint8(mat2gray(imvolx))) , hold all,colormap(jet),colorbar;

[poly, mask, smoothedPoly,sp] = regionGrowing2(im2uint8(mat2gray(imfilter(imvolx,[0 -1 0;-1 5 -1;0 -1 0]))),4,[],itrMx,itrMxRG);

waitfor(mask);

mask=uint16(mask);

for i=1:O

imvol2(:,:,i)=mask.*imvol(:,:,i);

end

y=(zeros(1,O));

y(1:O)=sum(sum(imvol2(:,:,1:O)));

mx1=max((y));

mx1=mx1(1);

mn1=min((y));

mn1=mn1(1);

ind_max=find(y==mx1);

ind_min=find(y==mn1);

max_img=imvol(:,:,ind_max(1));

min_img=imvol(:,:,ind_min(1));

ind_max=ind_max(1);

%save ind_max ind_max

%%

% if (ind_min~=16 || ind_max~=16)

% ind_max2=ind_max+1;

% ind_min2=ind_min+1;

% end

%max_img2=imvol(:,:,ind_max2);

%min_img2=imvol(:,:,ind_min2);

%% sum

%max_img=max_img+1.2*max_img2;

%min_img=min_img+0.75*(min_img2);

%% ROI max_img

[All_masks,bw, poly, smoothedPoly,bw1,poly1,smoothedPoly1]=ROI_max_min(Imag,max_img,min_img,sp,imvol2);

waitfor(bw);

%bw =uint16(bw);

%% ROI min_img

%[bw1, poly1, smoothedPoly1]=ROI_max_min(bw,min_img,2,meanBG);

waitfor(bw1);

%bw1 =uint16(bw1);

%% show max and min images with their ROI's

% uncomment 78-82 if you want to show max image during execution

%figure(3)

%subplot(1,2,1),imshow(max_img,[]),colormap(jet),colorbar ;title('MAX Image');

%hold on

%impoly(gca,smoothedPoly);

%hold off

imvolmx=max_img;

imvolmx(bw==0)=0;

% uncomment 86-91 if you want to show max roi during execution

%subplot(1,2,2),imshow(imvolmx,[]),colormap(jet),colorbar ;title('MAX ROI Image');

%figure(4)

%subplot(1,2,1),imshow(min_img,[]),colormap(jet),colorbar ;title('MIN Image');

%hold on

%impoly(gca,smoothedPoly1);

%hold off

imvolmn=min_img;

imvolmn(bw1==0)=0;

%subplot(1,2,2),imshow(imvolmn,[]),colormap(jet),colorbar ;title('MIN ROI Image');

%% compute area difference

[m1 n1]=find(bw==1);

[m2 n2]=find(bw1==1);

e1=fit_ellipse(m1,n1);

%area1=0.2645833333*(e1.a)*0.2645833333*(e1.b)*pi;%max

area1=(e1.a)*(e1.b)*pi;%max

e2=fit_ellipse(m2,n2);

%area2=0.2645833333*(e2.a)*0.2645833333*(e2.b)*pi;%min

area2=(e2.a)*(e2.b)*pi;

rate=area2/area1;

%% Diameters Ration

minLongDIVmaxL=(e1.long_axis-e2.long_axis)/e1.long_axis; % LA FS in excel sheet

minShortDIVmaxL=(e1.short_axis-e2.short_axis)/e1.short_axis;% SA FS in excel sheet

AverageDiv=(minLongDIVmaxL+minShortDIVmaxL)/2; % Average FS in excel sheet

%% max image

PerimeterMax=2*pi*sqrt(((e1.short_axis)^2+(e1.long_axis)^2)/2);

circularityMax=(4*pi*area1)/(PerimeterMax.^2); %Circulatory diastole in excel sheet

ElongationMax=e1.short_axis/e1.long_axis; %Elongation diastole in excel sheet

%% min image

PerimeterMin=2*pi*sqrt((e2.short_axis^2+e2.long_axis^2)/2);

circularityMin=(4*pi*area2)/(PerimeterMin.^2); %Circulatory systole in excel sheet

ElongationMin=e2.short_axis/e2.long_axis; %Elongation systole in excel sheet

%%%%%%%%%%%%%%%%%%%%%%%%%%%%%%%%%%%%%%%%%%%%%%%%

| **S** | **M-file name** | **Type** | **Description** | **Novel or open-source** |
| --- | --- | --- | --- | --- |
|  | max_min_MUGA3 | Fun | The same as max_min_MUGA but without plotting capbabilities. | The same as max_min_MUGA |

**Code:** The same as max_min_MUGA but without plotting capbabilities, also the function call Roi_max_min3 not Roi_max_min.

%%%%%%%%%%%%%%%%%%%%%%%%%%%%%%%%%%%%%%%%%%%%%%%%

| **M-file name** | MyHistogram |
| --- | --- |
| **Type** |  |
| **Description** | Compute and plot histogram of phase image.  Inputs:  X1: phase image  X2: number of bins (16)  Outputs: Counts: counts of each gray level in gray Levels matrix. |
| **comments** | Modified version of an open source (246) |

**Code:**

%%

function [counts, grayLevels] = MyHistogram(x1,x2)

[rows, columns, numberOfColorChannels] = size(x1);

counts = zeros(1, 361);

mxVal=max(x2(:));

for col = 1 : columns

for row = 1 : rows

% Get the gray level.

grayLevel = round(x1(row, col));

% Add 1 because graylevel zero goes into index 1 and so on.

if (x2(row,col)>0.001*mxVal)

counts(grayLevel+ 1) = counts(grayLevel+1) + 1;

end

end

end

% Plot the histogram.

grayLevels = 0 : 360;

bar(grayLevels, counts, 'BarWidth', 0.5, 'FaceColor', 'b');

xlabel('degree', 'FontSize', 10);

ylabel('frequency', 'FontSize', 10);

title('Histogram', 'FontSize', 10);

%axis([-180 180 0 max(counts)]);

grid on;

end

%%%%%%%%%%%%%%%%%%%%%%%%%%%%%%%%%%%%%%%%%%%%%%%%

| **M-file name** | PhaseBkg16Images |
| --- | --- |
| **Type** | Script |
| **Description** | Experiment to compute the phase image and phase histogram of the 16-ROIs of the MUGA image. |
| **comments** | Novel: Last modified 14-7-2021 |

**Code:**

x = inputdlg('Pick Post image:',...

'Define Image', [1]);

name= (x{:}); % image number

imvol = squeeze(dicomread(name));

info = dicominfo(name);

save info info;

for i=1:1:info.NumberOfFrames

imvol(:,:,i)=medfilt2(imvol(:,:,i),[3 3]);%

end

figure,imshow(sum(imvol,3),[]); title('Select seed point');

colormap jet;colorbar

sp = ginput(1);

figure

[a,B, C, D,min_img2,F,imvolmn2,bw1, AA, AB, AC, AD, AE, AF,AG, AH, AI, aj, sp]=max_min_MUGA3(imvol,0,sp,[],[]);

NewImg5=imdilate(bw1,strel('disk',3))-imdilate(bw1,strel('disk',2));

S5=regionprops(bwlabel(NewImg5),'Centroid');

cc5=round([S5.Centroid]);

D5=sum(sum(imvolmn2(cc5(2):end,cc5(1):end)));

NewImg5(1:cc5(2),1:cc5(1))=0;

NewImg5(1:cc5(2),cc5(1):end)=0;

NewImg5(cc5(2):end,1:cc5(1))=0;

imvolmn11=imvol;

imvolmn11(NewImg5==0)=0;

meanBGMn=mean(mean(max(imvol(NewImg5==1))));

%meanBGMn=mean([meanBG1,meanBG2,meanBG3,meanBG4,meanBG5])

imvol=imvol-meanBGMn;

phase=(zeros(128,128,16));amp=phase;

for i=1:128

for j=1:128

%AA(1:16)=imvol1(i,j,1:16);

%f=(fftshift(fft(double(AA))));

%Im=sum(exp(1i*angle(f))); R=sum(abs(f));

%phase=sum(rad2deg(atan(Im./R)));

%amp=sum(sqrt((Im.^2)+(R.^2)));

%A1(i,j)=ifft(Im); A2(i,j)=R;

F =(fft(double(imvol(i,j,1:16))));

F_Mag = (abs((F)));

F_Phase =(rad2deg(exp(1i*angle(F))));

% reconstructin

I_Mag = abs(ifft(F_Mag));

I_Phase =abs(ifft(F_Phase));

% Calculate limits for plotting

phase(i,j,1:16)=(I_Phase);

amp(i,j,1:16)=(I_Mag);

end

end

% ss=abs(sum(phase),3);

% max(ss(:))

% min(ss(:))

% max(ss(:))

subplot(131), imshow(((sum(phase,3))),[]), title('Phase plot');colormap jet;colorbar

subplot(132), imshow((sum(amp,3)),[]), title('Amplitude plot');colormap jet;colorbar

subplot(133)

[counts, grayLevels] = MyHistogram(abs(sum(phase,3)),(sum(amp,3)));

axis([-10 360 0 40])

figure

for i=1:16

subplot(4,4,i), imshow((phase(:,:,i)),[]);colormap jet;

end

figure

for i=1:16

subplot(4,4,i), imshow((amp(:,:,i)),[]);colormap jet;

end

%%%%%%%%%%%%%%%%%%%%%%%%%%%%%%%%%%%%%%%%%%%%%%%%

| **M-file name** | PhaseBkgFn |
| --- | --- |
| **Type** | Fun |
| **Description** | Function of computing the phase image of MUGA image. |
| **comments** | Novel: Last modified: 31-5-2021 |

**Code:**

function [phase] = PhaseBkgFn(imvol,bw,sp,postpre)

for i=1:1:16

imvol(:,:,i)=imvol(:,:,i).*bw;%

end

figure

[a,B, C, D,min_img2,F,imvolmn2,bw1, AA, AB, AC, AD, AE, AF,AG, AH, AI, aj, sp]=max_min_MUGA3(imvol,0,sp,[],[]);

NewImg5=imdilate(bw1,strel('disk',3))-imdilate(bw1,strel('disk',2));

S5=regionprops(bwlabel(NewImg5),'Centroid');

cc5=round([S5.Centroid]);

D5=sum(sum(imvolmn2(cc5(2):end,cc5(1):end)));

NewImg5(1:cc5(2),1:cc5(1))=0;

NewImg5(1:cc5(2),cc5(1):end)=0;

NewImg5(cc5(2):end,1:cc5(1))=0;

imvolmn11=imvol;

imvolmn11(NewImg5==0)=0;

meanBGMn=mean(mean(max(imvol(NewImg5==1))));

%meanBGMn=mean([meanBG1,meanBG2,meanBG3,meanBG4,meanBG5])

imvol=imvol-meanBGMn;

phase=(zeros(128,128,3));amp=phase;

for i=1:128

for j=1:128

%AA(1:16)=imvol1(i,j,1:16);

%f=(fftshift(fft(double(AA))));

%Im=sum(exp(1i*angle(f))); R=sum(abs(f));

%phase=sum(rad2deg(atan(Im./R)));

%amp=sum(sqrt((Im.^2)+(R.^2)));

%A1(i,j)=ifft(Im); A2(i,j)=R;

F =(fft(double(imvol(i,j,1:16))));

F_Mag = (abs((F)));

F_Phase =(rad2deg(exp(1i*angle(F))));

% reconstructin

I_Mag = abs(ifft(F_Mag));

I_Phase =abs(ifft(F_Phase));

% Calculate limits for plotting

phase(i,j,1:16)=(I_Phase);

amp(i,j,1:16)=(I_Mag);

end

end

% ss=abs(sum(phase),3);

% max(ss(:))

% min(ss(:))

% max(ss(:))

subplot(131), imshow(((sum(phase,3))),[]), title('Phase plot');colormap jet;colorbar

subplot(132), imshow((sum(amp,3)),[]), title('Amplitude plot');colormap jet;colorbar

subplot(133)

[counts, grayLevels] = MyHistogram(abs(sum(phase,3)),(sum(amp,3)));

axis([-10 360 0 40])

if (postpre==1)sgtitle('post');

else sgtitle('pre');

end

%%%%%%%%%%%%%%%%%%%%%%%%%%%%%%%%%%%%%%%%%%%%%%%%

| **M-file name** | phaseImageAll_ROIs |
| --- | --- |
| **Type** | Script |
| **Description** | Experiment to compute the 16-ROIs phase images of a given MUGA image using FFT2 transform. |
| **comments** | Novel: Last modified 5-5-2021 |

**Code:**

x = inputdlg('Pick Post image:',...

'Define Image', [1]);

handles.name= (x{:}); % image number

handles.imvol = squeeze(dicomread(handles.name));

info = dicominfo(handles.name);

save info info;

load meanBGMn2 meanBGMn2;

load meanBGMn meanBGMn;

for i=1:1:info.NumberOfFrames

handles.imvol(:,:,i)=medfilt2(handles.imvol(:,:,i),[5 5]);%

end

volx=(handles.imvol-meanBGMn2);

image =( volx);

F = fft2(double(image));

F_Mag = abs(F);

F_Phase = rad2deg ((exp(1i*angle(F))));

% reconstructin

I_Mag = ifft2(log(F_Mag+1));

I_Phase =uint16(360*(mat2gray( abs(ifft2(F_Phase)))));

% Calculate limits for plotting

I_Mag_min = min(min(abs(I_Mag)));

I_Mag_max = max(max(abs(I_Mag)));

I_Phase_min = min(min(abs(I_Phase)));

I_Phase_max = max(max(abs(I_Phase)));

% Display reconstructed images

bw=uint16(zeros(size(I_Phase)));

for i=1:16

bw(:,:,i)=uint16(I_Phase(:,:,i)>round(mean(I_Phase(:))));

bw(:,:,i)=(bwareaopen(bw(:,:,i),200));

bw(:,:,i)=bwlabel(imfill(bw(:,:,i),'holes'));

end

subplot(121),imshow(label2rgb(sum(bw,3),'jet','b', 'shuffle'),[]), colorbar

title('reconstructed image only by Phase');

subplot(122)

[counts, grayLevels] = MyHistogram((I_Phase),(I_Mag));

title('reconstructed image only by Magnitude');

axis([0 360 0 30]);

%%%%%%%%%%%%%%%%%%%%%%%%%%%%%%%%%%%%%%%%%%%%%%%%

| **M-file name** | PlotEpsilonVsApEn |
| --- | --- |
| **Type** | Script |
| **Description** | Plot epsilon against ApEn |
| **comments** | Novel: Last modified 15-3-2021 |

**Code:**

BoundApEn=[];epsilon=[];Lespsilon=[];

k=0;

while (1)

k=k+1;

range=strcat('Y',num2str(3+k),':AA',num2str(3+k));

M = xlsread('platform.xlsx','Sheet1',range);

if(isempty(M))break;end

BoundApEn(k) =M(1);

epsilon(k)=M(2);

Lespsilon(k)=M(3);

end

figure

subplot(1,2,1),plot(Lespsilon,BoundApEn,'+r');

xlabel('Log(\epsilon)');

ylabel('ApEn');

axis([-5 0 0 1.5])

title('pre');

BoundApEn=[];epsilon=[];Lespsilon=[];k=0;

while (1)

k=k+1;

range=strcat('BA',num2str(3+k),':BC',num2str(3+k));

M = xlsread('platform.xlsx','Sheet1',range);

if(isempty(M))break;end

BoundApEn(k) =M(1);

epsilon(k)=M(2);

Lespsilon(k)=M(3);

end

subplot(1,2,2),plot(Lespsilon,BoundApEn,'+r');

xlabel('Log(\epsilon)');

ylabel('ApEn');

axis([-5 0 0 1.5])

title('post');

%%%%%%%%%%%%%%%%%%%%%%%%%%%%%%%%%%%%%%%%%%%%%%%%

| **M-file name** | regionGrowing2 |
| --- | --- |
| **Type** |  |
| **Description** | Apply the Region Growing (RGalgorithm) on the MUGA image. ***Note***: RG will call the built-in function (activecountoring) to apply the AC algorithm inside the Mask of the RG algorithm of the phase image. |
| **comments** | Novel: Written and modified 15-7-2021  Last modified: 8-9-2021 |

**Code:**

function [P, J, smoothedPoly,seedPointx,ext_energy] = regionGrowing2(ImgO,n,seedPoint,numofIter,numofIterRG)

amp=(zeros(128,128));phase=amp;

for i=1:128

for j=1:128

F =(fft(double(ImgO(i,j))));

F_Mag = (abs((F)));

% reconstructin

I_Mag = abs(ifft(F_Mag));

F_Phase =(rad2deg(exp(1i*angle(F))));

% Calculate limits for plotting

amp(i,j)=(I_Mag);

I_Phase =abs(ifft(F_Phase));

% Calculate limits for plotting

phase(i,j)=(I_Phase);

end

end

Img=phase;

%figure,imshow(Img,[]);colormap jet

if (n==1 || n==2 || n==3)

p1=seedPoint;

if (n==1)

sp=p1;

save sp sp;

end

else

p1 = ginput(1);

end

Px(1) =p1(1,1); Py(1) =p1(1,2);

Px(2) =p1(1,1)-1; Py(2) =p1(1,2)-1;

Px(3) =p1(1,1)+1; Py(3) =p1(1,2)-1;

Px(4) =p1(1,1)-1; Py(4) =p1(1,2)+1;

Px(5) =p1(1,1)-1; Py(5) =p1(1,2);

Px(6) =p1(1,1); Py(6) =p1(1,2)+1;

Px(7) =p1(1,1); Py(7) =p1(1,2)-1;

Px(8) =p1(1,1)+1; Py(8) =p1(1,2);

p(:,1)=Px';

p(:,2)=Py';

p=uint8(p);

mask = zeros(size(Img));

for i=1:size(p,1)

mask(p(i,2),p(i,1)) = 1;

end

load AC SmoothFactor ContractionBias method

if (n==1 || n==4) % max roi and unified roi

J = activecontour((Img),mask,numofIter,method,'SmoothFactor',SmoothFactor,'ContractionBias',ContractionBias);

elseif (n==2) % min roi

J = activecontour((Img),mask,numofIter,method,'SmoothFactor',SmoothFactor,'ContractionBias',ContractionBias);

elseif (n==3)% all 16 ROIs

J = activecontour((Img),mask,numofIter,method,'SmoothFactor',SmoothFactor,'ContractionBias',ContractionBias);

load AC SmoothFactor ContractionBias method

[seg force] = chenvese(ImgO,J,numofIter,SmoothFactor,'chan');

ext_energy=mean(force(:));

end

%figure,imshow(phase,[])

%

J=imfilter(J,ones(11)/121);

J=imdilate(J,strel('rectangle',[8 2]));

%figure,imshow(J)

% Extract polygon of J

s=regionprops(bwlabel(J),'ConvexHull','Area');

Area=[s.Area];

[xx yy]=find(Area==max(Area));

P=[s(yy).ConvexHull];

%% apply region growing not active contouring AC inside the ROI of AC algorithm

[J1] =regiongrowingX(J.*double(amp),round(p1(2)),round(p1(1)),numofIterRG);

%round(p1) uint8(J).*

%%

numofIter2=numofIter;

load maskallparam numofIter

if numofIter2==min(numofIter)

J=imfilter(J1,ones(9)/81);

J=imdilate(J,strel('rectangle',[4 2]));

else

J=imfilter(J1,ones(11)/121);

J=imdilate(J,strel('rectangle',[6 2]));

end

J=imfill(J,'holes');

J=imopen(J,strel('disk',2));

J=imclose(J,strel('disk',2));

%figure,imshow(J);

s=regionprops(bwlabel(J),'ConvexHull','Area');

Area=[s.Area];

[xx yy]=find(Area==max(Area));

P=[s(yy).ConvexHull];

smoothedPoly=P;

seedPointx=p1;

%%%%%%%%%%%%%%%%%%%%%%%%%%%%%%%%%%%%%%%%%%%%%%%%

| **S** | **M-file name** | **Type** | **Description** | **Novel or open-source** |
| --- | --- | --- | --- | --- |
|  | regionGrowing3 | Fun | The same as regionGrowing2 but without some plotting functions | The same as regionGrowing2 |

**Code:** the same as regionGrowing2 with some little modifications (no energy computations ad no plotting).

%%%%%%%%%%%%%%%%%%%%%%%%%%%%%%%%%%%%%%%%%%%%%%%%

| **M-file name** |  |
| --- | --- |
| **Type** |  |
| **Description** | This function is used to apply region growing RG (not active contouring AC) inside the ROI of AC algorithm. This function is modified so that the (numofiter) will control the algorithm. |
| **comments** | Modified from open Source, D. Kroon, University of Twente (247) |

**Code:**

function J=regiongrowingX(I,x,y,numofIter)

% This function performs "region growing" in an image from a specified

% seedpoint (x,y)

% J = regiongrowing(I,x,y,t)

% Example:

% I = im2double(imread('medtest.png'));

% x=198; y=359;

% J = regiongrowing(I,x,y,0.2);

% figure, imshow(I+J);

% Author: D. Kroon, University of Twente

if(exist('reg_maxdist','var')==0), reg_maxdist=0.2; end

if(exist('y','var')==0), figure, imshow(I,[]); [y,x]=getpts; y=round(y(1)); x=round(x(1)); end

J = zeros(size(I)); % Output

Isizes = size(I); % Dimensions of input image

reg_mean = I(x,y); % The mean of the segmented region

reg_size = 1; % Number of pixels in region

% Free memory to store neighbours of the (segmented) region

neg_free = 10000; neg_pos=0;

neg_list = zeros(neg_free,3);

pixdist=0; % Distance of the region newest pixel to the regio mean

% Neighbor locations (footprint)

neigb=[-1 0; 1 0; 0 -1;0 1];

% Start regiogrowing until distance between regio and posible new pixels become

% higher than a certain treshold

cnt=1;

while(cnt<numofIter)

cnt=cnt+1;

% Add new neighbors pixels

for j=1:4,

% Calculate the neighbour coordinate

xn = x +neigb(j,1); yn = y +neigb(j,2);

% Check if neighbour is inside or outside the image

ins=(xn>=1)&&(yn>=1)&&(xn<=Isizes(1))&&(yn<=Isizes(2));

% Add neighbor if inside and not already part of the segmented area

if(ins&&(J(xn,yn)==0))

neg_pos = neg_pos+1;

neg_list(neg_pos,:) = [xn yn I(xn,yn)]; J(xn,yn)=1;

end

end

% Add a new block of free memory

if(neg_pos+10>neg_free), neg_free=neg_free+10000; neg_list((neg_pos+1):neg_free,:)=0; end

% Add pixel with intensity nearest to the mean of the region, to the region

dist = abs(neg_list(1:neg_pos,3)-reg_mean);

[pixdist, index] = min(dist);

J(x,y)=2; reg_size=reg_size+1;

% Calculate the new mean of the region

reg_mean= (reg_mean*reg_size + neg_list(index,3))/(reg_size+1);

% Save the x and y coordinates of the pixel (for the neighbour add proccess)

x = neg_list(index,1); y = neg_list(index,2);

% Remove the pixel from the neighbour (check) list

neg_list(index,:)=neg_list(neg_pos,:); neg_pos=neg_pos-1;

end

% Return the segmented area as logical matrix

J=J>1;

%%%%%%%%%%%%%%%%%%%%%%%%%%%%%%%%%%%%%%%%%%%%%%%%

| **M-file name** | ROI_max_min |
| --- | --- |
| **Type** | Fun |
| **Description** | Function used to compute the maximum and minimum ROI of image. |
| **comments** | Novel: Written and modified: 15-7-2021  Last modified: 8-9-2021 |

**Code:**

function [All_masks,bw, poly, smoothedPoly,bw1,poly1,smoothedPoly1]=ROI_max_min(imvol,max_img,min_img,sp,imvol2)

load maskallparam numofIter numofIterRG XD YD

I2=max_img;

itrMx=max(numofIter);

itrMxRG=max(numofIterRG);

%I2=medfilt2(I2,[5 5]);

%s=strel('disk',1);

%I3=imopen(I2,fliplr(s));

I3=imfilter(I2,[0 -1 0;-1 5 -1;0 -1 0]);

%figure(31), imshow(im2uint8(mat2gray(I2)),[]) , hold all,colormap(jet),colorbar, title("Pick a centered seed in this Image");

[poly, mask, smoothedPoly,sp] = regionGrowing2(im2uint8(mat2gray(I3)),1,sp,itrMx,itrMxRG);

waitfor(poly);

bw=mask;

%if (n==1)

I2=min_img;

%I2=medfilt2(I,[5 5]);

%s=strel('disk',1);

%I3=imopen(I2,fliplr(s));

I3=imfilter(I2,[0 -1 0;-1 5 -1;0 -1 0]);

sp1=sp;

sp1(1,2)=sp(1,2)-7*YD;

sp1(1,1)=sp(1,1)-7*XD;

itrMin=min(numofIter);

itrMinRG=min(numofIterRG);

%elseif (n==2)

%figure(41), imshow(im2uint8(mat2gray(I)),[]) , hold all,colormap(jet),colorbar, title("Pick a centered seed in this Min Image");

[poly1, mask2, smoothedPoly1] = regionGrowing2(im2uint8(mat2gray(I3)),2,sp1,itrMin,itrMinRG);

waitfor(poly);

bw1=mask2;

%bw1(bw==0)=0;

%end

%[roiwindow]=example4(I,poly);

%figure,imshow(mask,[]);

All_masks=mask_all(imvol,sp,imvol2,bw);

end

%%%%%%%%%%%%%%%%%%%%%%%%%%%%%%%%%%%%%%%%%%%%%%%%

| **S** | **M-file name** | **Type** | **Description** | **Novel or open-source** |
| --- | --- | --- | --- | --- |
|  | ROI_max_min3 | Fun | The same as ROI_max_min but without plotting | Novel as ROI_max_min |

**Code:** The same as ROI_max_min but without plotting

%%%%%%%%%%%%%%%%%%%%%%%%%%%%%%%%%%%%%%%%%%%%%%%%

| **M-file name** | Filtering_ Experiment_MSI_PSNR |
| --- | --- |
| **Type** | Script |
| **Description** | This script apply an experiment that uses the MUGA image (16-frame), applies different noise types on it, then apply different filters (Median, Average or mean, Wiener and MMWF). The PSNR and MSE are computed and stored in the file called Filtering.xlsx file. |
| **comments** | Novel: Last modified 8-8-2021 |

**Code:**

% read images (post-chemo).

x = inputdlg('Pick Post image:',...

'Define Image', [1]);

name= (x{:}); % image number

imvol = squeeze(dicomread(name));

info = dicominfo(name);

imvol=uint16(sum(imvol,3));

% adding gaussian noise to imvol

NG1=imnoise(imvol,'Gaussian',0,0.00001);

NG2=imnoise(imvol,'Gaussian',0,0.00002);

NG3=imnoise(imvol,'Gaussian',0,0.00003);

NG4=imnoise(imvol,'Gaussian',0,0.00004);

NG5=imnoise(imvol,'Gaussian',0,0.00005);

NG6=imnoise(imvol,'Gaussian',0,0.00006);

% adding poisson noise to imvol

% if you work on a specific ROI you need to compute bw using max_min_muga

I1=imvol;

I11=uint16(imvol.*1.1);

I12=uint16(imvol.*1.2);

I13=uint16(imvol.*1.3);

I14=uint16(imvol.*1.4);

I15=uint16(imvol.*1.5);

I16=uint16(imvol.*1.6);

bw1=ones(size(I11));

f1 = @(x) imnoise(x,'poisson');

NP1=roifilt2(I11,bw1,f1);

NP2=roifilt2(I12,bw1,f1);

NP3=roifilt2(I13,bw1,f1);

NP4=roifilt2(I14,bw1,f1);

NP5=roifilt2(I15,bw1,f1);

NP6=roifilt2(I16,bw1,f1);

% adding speckle noise to imvol

NS1=imnoise(imvol,'Speckle',0.1);

NS2=imnoise(imvol,'Speckle',0.2);

NS3=imnoise(imvol,'Speckle',0.3);

NS4=imnoise(imvol,'Speckle',0.4);

NS5=imnoise(imvol,'Speckle',0.5);

NS6=imnoise(imvol,'Speckle',0.6);

%% Filtering

%% 1st: filtering gaussian noise

% Median Filtering

FG_MED1=medfilt2(NG1,[5 5]); % FG means Filter gaussian noise, med means using median filter

FG_MED2=medfilt2(NG2,[5 5]);

FG_MED3=medfilt2(NG3,[5 5]);

FG_MED4=medfilt2(NG4,[5 5]);

FG_MED5=medfilt2(NG5,[5 5]);

FG_MED6=medfilt2(NG6,[5 5]);

% Mean Filtering

h1=fspecial('average',5);

FG_AVG1=filter2(h1,NG1); % FG means Filter gaussian noise, AVG means using mean (average) filter

FG_AVG2=filter2(h1,NG2);

FG_AVG3=filter2(h1,NG3);

FG_AVG4=filter2(h1,NG4);

FG_AVG5=filter2(h1,NG5);

FG_AVG6=filter2(h1,NG6);

% Wiener Filtering

FG_WNR1=wiener2(NG1,[5 5]); % FG means Filter gaussian noise, med means using weiner filter

FG_WNR2=wiener2(NG2,[5 5]);

FG_WNR3=wiener2(NG3,[5 5]);

FG_WNR4=wiener2(NG4,[5 5]);

FG_WNR5=wiener2(NG5,[5 5]);

FG_WNR6=wiener2(NG6,[5 5]);

% MMWF Filtering

FG_MMW1=MMWF_2D(NG1,5); % FG means Filter gaussian noise, med means using modified median weiner filter

FG_MMW2=MMWF_2D(NG2,5);

FG_MMW3=MMWF_2D(NG3,5);

FG_MMW4=MMWF_2D(NG4,5);

FG_MMW5=MMWF_2D(NG5,5);

FG_MMW6=MMWF_2D(NG6,5);

%% 2nd: filtering poisson noise

% Median Filtering

FP_MED1=medfilt2(NP1,[5 5]); % FP means Filter possion noise, med means using median filter

FP_MED2=medfilt2(NP2,[5 5]);

FP_MED3=medfilt2(NP3,[5 5]);

FP_MED4=medfilt2(NP4,[5 5]);

FP_MED5=medfilt2(NP5,[5 5]);

FP_MED6=medfilt2(NP6,[5 5]);

% Mean Filtering

h1=fspecial('average',5);

FP_AVG1=filter2(h1,NP1); % FP means Filter possion noise, AVG means using mean (average) filter

FP_AVG2=filter2(h1,NP2);

FP_AVG3=filter2(h1,NP3);

FP_AVG4=filter2(h1,NP4);

FP_AVG5=filter2(h1,NP5);

FP_AVG6=filter2(h1,NP6);

% Wiener Filtering

FP_WNR1=wiener2(NP1,[5 5]); % FP means Filter possion noise, med means using weiner filter

FP_WNR2=wiener2(NP2,[5 5]);

FP_WNR3=wiener2(NP3,[5 5]);

FP_WNR4=wiener2(NP4,[5 5]);

FP_WNR5=wiener2(NP5,[5 5]);

FP_WNR6=wiener2(NP6,[5 5]);

% MMWF Filtering

FP_MMW1=MMWF_2D(NP1,5); % FP means Filter possion noise, med means using modified median weiner filter

FP_MMW2=MMWF_2D(NP2,5);

FP_MMW3=MMWF_2D(NP3,5);

FP_MMW4=MMWF_2D(NP4,5);

FP_MMW5=MMWF_2D(NP5,5);

FP_MMW6=MMWF_2D(NP6,5);

%% 3rd: filtering speckle noise

% Median Filtering

FS_MED1=medfilt2(NS1,[5 5]); % FS means Filter speckle noise, med means using median filter

FS_MED2=medfilt2(NS2,[5 5]);

FS_MED3=medfilt2(NS3,[5 5]);

FS_MED4=medfilt2(NS4,[5 5]);

FS_MED5=medfilt2(NS5,[5 5]);

FS_MED6=medfilt2(NS6,[5 5]);

% Mean Filtering

h1=fspecial('average',5);

FS_AVG1=filter2(h1,NS1); % FS means Filter speckle noise, AVG means using mean (average) filter

FS_AVG2=filter2(h1,NS2);

FS_AVG3=filter2(h1,NS3);

FS_AVG4=filter2(h1,NS4);

FS_AVG5=filter2(h1,NS5);

FS_AVG6=filter2(h1,NS6);

% Wiener Filtering

FS_WNR1=wiener2(NS1,[5 5]); % FS means Filter speckle noise, med means using weiner filter

FS_WNR2=wiener2(NS2,[5 5]);

FS_WNR3=wiener2(NS3,[5 5]);

FS_WNR4=wiener2(NS4,[5 5]);

FS_WNR5=wiener2(NS5,[5 5]);

FS_WNR6=wiener2(NS6,[5 5]);

% MMWF Filtering

FS_MMW1=MMWF_2D(NS1,5); % FS means Filter speckle noise, med means using modified median weiner filter

FS_MMW2=MMWF_2D(NS2,5);

FS_MMW3=MMWF_2D(NS3,5);

FS_MMW4=MMWF_2D(NS4,5);

FS_MMW5=MMWF_2D(NS5,5);

FS_MMW6=MMWF_2D(NS6,5);

%% compute PSNR

%10%

PSNR_G10_MED= round(psnr(imvol, uint16(FG_MED1)));

PSNR_G10_AVG= round(psnr(imvol, uint16(FG_AVG1)));

PSNR_G10_WNR= round(psnr(imvol, uint16(FG_WNR1)));

PSNR_G10_MMW= round(psnr(imvol, uint16(FG_MMW1)));

PSNR_P10_MED= round(psnr(imvol, uint16(FP_MED1)));

PSNR_P10_AVG= round(psnr(imvol, uint16(FP_AVG1)));

PSNR_P10_WNR= round(psnr(imvol, uint16(FP_WNR1)));

PSNR_P10_MMW= round(psnr(imvol, uint16(FP_MMW1)));

PSNR_S10_MED= round(psnr(imvol, uint16(FS_MED1)));

PSNR_S10_AVG= round(psnr(imvol, uint16(FS_AVG1)));

PSNR_S10_WNR= round(psnr(imvol, uint16(FS_WNR1)));

PSNR_S10_MMW= round(psnr(imvol, uint16(FS_MMW1)));

%20%

PSNR_G20_MED= round(psnr(imvol, uint16(FG_MED2)));

PSNR_G20_AVG= round(psnr(imvol, uint16(FG_AVG2)));

PSNR_G20_WNR= round(psnr(imvol, uint16(FG_WNR2)));

PSNR_G20_MMW= round(psnr(imvol, uint16(FG_MMW2)));

PSNR_P20_MED= round(psnr(imvol, uint16(FP_MED2)));

PSNR_P20_AVG= round(psnr(imvol, uint16(FP_AVG2)));

PSNR_P20_WNR= round(psnr(imvol, uint16(FP_WNR2)));

PSNR_P20_MMW= round(psnr(imvol, uint16(FP_MMW2)));

PSNR_S20_MED= round(psnr(imvol, uint16(FS_MED2)));

PSNR_S20_AVG= round(psnr(imvol, uint16(FS_AVG2)));

PSNR_S20_WNR= round(psnr(imvol, uint16(FS_WNR2)));

PSNR_S20_MMW= round(psnr(imvol, uint16(FS_MMW2)));

%30%

PSNR_G30_MED= round(psnr(imvol, uint16(FG_MED3)));

PSNR_G30_AVG= round(psnr(imvol, uint16(FG_AVG3)));

PSNR_G30_WNR= round(psnr(imvol, uint16(FG_WNR3)));

PSNR_G30_MMW= round(psnr(imvol, uint16(FG_MMW3)));

PSNR_P30_MED= round(psnr(imvol, uint16(FP_MED3)));

PSNR_P30_AVG= round(psnr(imvol, uint16(FP_AVG3)));

PSNR_P30_WNR= round(psnr(imvol, uint16(FP_WNR3)));

PSNR_P30_MMW= round(psnr(imvol, uint16(FP_MMW3)));

PSNR_S30_MED= round(psnr(imvol, uint16(FS_MED3)));

PSNR_S30_AVG= round(psnr(imvol, uint16(FS_AVG3)));

PSNR_S30_WNR= round(psnr(imvol, uint16(FS_WNR3)));

PSNR_S30_MMW= round(psnr(imvol, uint16(FS_MMW3)));

%40%

PSNR_G40_MED= round(psnr(imvol, uint16(FG_MED4)));

PSNR_G40_AVG= round(psnr(imvol, uint16(FG_AVG4)));

PSNR_G40_WNR= round(psnr(imvol, uint16(FG_WNR4)));

PSNR_G40_MMW= round(psnr(imvol, uint16(FG_MMW4)));

PSNR_P40_MED= round(psnr(imvol, uint16(FP_MED4)));

PSNR_P40_AVG= round(psnr(imvol, uint16(FP_AVG4)));

PSNR_P40_WNR= round(psnr(imvol, uint16(FP_WNR4)));

PSNR_P40_MMW= round(psnr(imvol, uint16(FP_MMW4)));

PSNR_S40_MED= round(psnr(imvol, uint16(FS_MED4)));

PSNR_S40_AVG= round(psnr(imvol, uint16(FS_AVG4)));

PSNR_S40_WNR= round(psnr(imvol, uint16(FS_WNR4)));

PSNR_S40_MMW= round(psnr(imvol, uint16(FS_MMW4)));

%50%

PSNR_G50_MED= round(psnr(imvol, uint16(FG_MED5)));

PSNR_G50_AVG= round(psnr(imvol, uint16(FG_AVG5)));

PSNR_G50_WNR= round(psnr(imvol, uint16(FG_WNR5)));

PSNR_G50_MMW= round(psnr(imvol, uint16(FG_MMW5)));

PSNR_P50_MED= round(psnr(imvol, uint16(FP_MED5)));

PSNR_P50_AVG= round(psnr(imvol, uint16(FP_AVG5)));

PSNR_P50_WNR= round(psnr(imvol, uint16(FP_WNR5)));

PSNR_P50_MMW= round(psnr(imvol, uint16(FP_MMW5)));

PSNR_S50_MED= round(psnr(imvol, uint16(FS_MED5)));

PSNR_S50_AVG= round(psnr(imvol, uint16(FS_AVG5)));

PSNR_S50_WNR= round(psnr(imvol, uint16(FS_WNR5)));

PSNR_S50_MMW= round(psnr(imvol, uint16(FS_MMW5)));

%60%

PSNR_G60_MED= round(psnr(imvol, uint16(FG_MED6)));

PSNR_G60_AVG= round(psnr(imvol, uint16(FG_AVG6)));

PSNR_G60_WNR= round(psnr(imvol, uint16(FG_WNR6)));

PSNR_G60_MMW= round(psnr(imvol, uint16(FG_MMW6)));

PSNR_P60_MED= round(psnr(imvol, uint16(FP_MED6)));

PSNR_P60_AVG= round(psnr(imvol, uint16(FP_AVG6)));

PSNR_P60_WNR= round(psnr(imvol, uint16(FP_WNR6)));

PSNR_P60_MMW= round(psnr(imvol, uint16(FP_MMW6)));

PSNR_S60_MED= round(psnr(imvol, uint16(FS_MED6)));

PSNR_S60_AVG= round(psnr(imvol, uint16(FS_AVG6)));

PSNR_S60_WNR= round(psnr(imvol, uint16(FS_WNR6)));

PSNR_S60_MMW= round(psnr(imvol, uint16(FS_MMW6)));

%% Compute MSE

%10%

MSE_G10_MED= round(immse(imvol, uint16(FG_MED1)));

MSE_G10_AVG= round(immse(imvol, uint16(FG_AVG1)));

MSE_G10_WNR= round(immse(imvol, uint16(FG_WNR1)));

MSE_G10_MMW= round(immse(imvol, uint16(FG_MMW1)));

MSE_P10_MED= round(immse(imvol, uint16(FP_MED1)));

MSE_P10_AVG= round(immse(imvol, uint16(FP_AVG1)));

MSE_P10_WNR= round(immse(imvol, uint16(FP_WNR1)));

MSE_P10_MMW= round(immse(imvol, uint16(FP_MMW1)));

MSE_S10_MED= round(immse(imvol, uint16(FS_MED1)));

MSE_S10_AVG= round(immse(imvol, uint16(FS_AVG1)));

MSE_S10_WNR= round(immse(imvol, uint16(FS_WNR1)));

MSE_S10_MMW= round(immse(imvol, uint16(FS_MMW1)));

%20%

MSE_G20_MED= round(immse(imvol, uint16(FG_MED2)));

MSE_G20_AVG= round(immse(imvol, uint16(FG_AVG2)));

MSE_G20_WNR= round(immse(imvol, uint16(FG_WNR2)));

MSE_G20_MMW= round(immse(imvol, uint16(FG_MMW2)));

MSE_P20_MED= round(immse(imvol, uint16(FP_MED2)));

MSE_P20_AVG= round(immse(imvol, uint16(FP_AVG2)));

MSE_P20_WNR= round(immse(imvol, uint16(FP_WNR2)));

MSE_P20_MMW= round(immse(imvol, uint16(FP_MMW2)));

MSE_S20_MED= round(immse(imvol, uint16(FS_MED2)));

MSE_S20_AVG= round(immse(imvol, uint16(FS_AVG2)));

MSE_S20_WNR= round(immse(imvol, uint16(FS_WNR2)));

MSE_S20_MMW= round(immse(imvol, uint16(FS_MMW2)));

%30%

MSE_G30_MED= round(immse(imvol, uint16(FG_MED3)));

MSE_G30_AVG= round(immse(imvol, uint16(FG_AVG3)));

MSE_G30_WNR= round(immse(imvol, uint16(FG_WNR3)));

MSE_G30_MMW= round(immse(imvol, uint16(FG_MMW3)));

MSE_P30_MED= round(immse(imvol, uint16(FP_MED3)));

MSE_P30_AVG= round(immse(imvol, uint16(FP_AVG3)));

MSE_P30_WNR= round(immse(imvol, uint16(FP_WNR3)));

MSE_P30_MMW= round(immse(imvol, uint16(FP_MMW3)));

MSE_S30_MED= round(immse(imvol, uint16(FS_MED3)));

MSE_S30_AVG= round(immse(imvol, uint16(FS_AVG3)));

MSE_S30_WNR= round(immse(imvol, uint16(FS_WNR3)));

MSE_S30_MMW= round(immse(imvol, uint16(FS_MMW3)));

%40%

MSE_G40_MED= round(immse(imvol, uint16(FG_MED4)));

MSE_G40_AVG= round(immse(imvol, uint16(FG_AVG4)));

MSE_G40_WNR= round(immse(imvol, uint16(FG_WNR4)));

MSE_G40_MMW= round(immse(imvol, uint16(FG_MMW4)));

MSE_P40_MED= round(immse(imvol, uint16(FP_MED4)));

MSE_P40_AVG= round(immse(imvol, uint16(FP_AVG4)));

MSE_P40_WNR= round(immse(imvol, uint16(FP_WNR4)));

MSE_P40_MMW= round(immse(imvol, uint16(FP_MMW4)));

MSE_S40_MED= round(immse(imvol, uint16(FS_MED4)));

MSE_S40_AVG= round(immse(imvol, uint16(FS_AVG4)));

MSE_S40_WNR= round(immse(imvol, uint16(FS_WNR4)));

MSE_S40_MMW= round(immse(imvol, uint16(FS_MMW4)));

%50%

MSE_G50_MED= round(immse(imvol, uint16(FG_MED5)));

MSE_G50_AVG= round(immse(imvol, uint16(FG_AVG5)));

MSE_G50_WNR= round(immse(imvol, uint16(FG_WNR5)));

MSE_G50_MMW= round(immse(imvol, uint16(FG_MMW5)));

MSE_P50_MED= round(immse(imvol, uint16(FP_MED5)));

MSE_P50_AVG= round(immse(imvol, uint16(FP_AVG5)));

MSE_P50_WNR= round(immse(imvol, uint16(FP_WNR5)));

MSE_P50_MMW= round(immse(imvol, uint16(FP_MMW5)));

MSE_S50_MED= round(immse(imvol, uint16(FS_MED5)));

MSE_S50_AVG= round(immse(imvol, uint16(FS_AVG5)));

MSE_S50_WNR= round(immse(imvol, uint16(FS_WNR5)));

MSE_S50_MMW= round(immse(imvol, uint16(FS_MMW5)));

%60%

MSE_G60_MED= round(immse(imvol, uint16(FG_MED6)));

MSE_G60_AVG= round(immse(imvol, uint16(FG_AVG6)));

MSE_G60_WNR= round(immse(imvol, uint16(FG_WNR6)));

MSE_G60_MMW= round(immse(imvol, uint16(FG_MMW6)));

MSE_P60_MED= round(immse(imvol, uint16(FP_MED6)));

MSE_P60_AVG= round(immse(imvol, uint16(FP_AVG6)));

MSE_P60_WNR= round(immse(imvol, uint16(FP_WNR6)));

MSE_P60_MMW= round(immse(imvol, uint16(FP_MMW6)));

MSE_S60_MED= round(immse(imvol, uint16(FS_MED6)));

MSE_S60_AVG= round(immse(imvol, uint16(FS_AVG6)));

MSE_S60_WNR= round(immse(imvol, uint16(FS_WNR6)));

MSE_S60_MMW= round(immse(imvol, uint16(FS_MMW6)));

%% show 10% results

% show 10% noise and filtering results

hf=figure

subplot(3,6,1);imshow(imvol,[]);title('Original','Fontsize',12);colormap jet;

subplot(3,6,2);imshow(NG1,[]);title('Gaussian noise 10%','Fontsize',12);colormap jet;

ha=subplot(3,6,3);imshow(FG_MED1,[]);title('Median filter','Fontsize',12);colormap jet;

pos = get(ha, 'position');pos(2)=pos(2)-0.05;

txt=strcat('PSNR= ',num2str(PSNR_G10_MED),', MSE= ',num2str(MSE_G10_MED));

annotation(hf, 'textbox', pos, 'String', num2str(txt), 'vert', 'bottom', 'EdgeColor','none','FontSize',12,'FitBoxToText','on');

ha=subplot(3,6,4);imshow(FG_AVG1,[]);title('Mean filter','Fontsize',12);colormap jet;

pos = get(ha, 'position');pos(2)=pos(2)-0.05;

txt=strcat('PSNR= ',num2str(PSNR_G10_AVG),', MSE= ',num2str(MSE_G10_AVG));

annotation(hf, 'textbox', pos, 'String', num2str(txt), 'vert', 'bottom', 'EdgeColor','none','FontSize',12,'FitBoxToText','on');

ha=subplot(3,6,5);imshow(FG_WNR1,[]);title('Weiner filter','Fontsize',12);colormap jet;

pos = get(ha, 'position');pos(2)=pos(2)-0.05;

txt=strcat('PSNR= ',num2str(PSNR_G10_WNR),', MSE= ',num2str(MSE_G10_WNR));

annotation(hf, 'textbox', pos, 'String', num2str(txt), 'vert', 'bottom', 'EdgeColor','none','FontSize',12,'FitBoxToText','on');

ha=subplot(3,6,6);imshow(FG_MMW1,[]);title('MMWF filer','Fontsize',12);colormap jet;

pos = get(ha, 'position');pos(2)=pos(2)-0.05;

txt=strcat('PSNR= ',num2str(PSNR_G10_MMW),', MSE= ',num2str(MSE_G10_MMW));

annotation(hf, 'textbox', pos, 'String', num2str(txt), 'vert', 'bottom', 'EdgeColor','none','FontSize',12,'FitBoxToText','on');

subplot(3,6,7);imshow(imvol,[]);title('Original','Fontsize',12);colormap jet;

subplot(3,6,8);imshow(NP6,[]);title(["Possion noise=","imnoise(Pixel Value*1.1)"],'Fontsize',12);colormap jet;

ha=subplot(3,6,9),imshow(FP_MED1,[]);title('Median filter','Fontsize',12);colormap jet;

pos = get(ha, 'position');pos(2)=pos(2)-0.05;

txt=strcat('PSNR= ',num2str(PSNR_P10_MED),', MSE= ',num2str(MSE_P10_MED));

annotation(hf, 'textbox', pos, 'String', num2str(txt), 'vert', 'bottom', 'EdgeColor','none','FontSize',12,'FitBoxToText','on');

ha=subplot(3,6,10);imshow(FP_AVG1,[]);title('Mean filter','Fontsize',12);colormap jet;

pos = get(ha, 'position');pos(2)=pos(2)-0.05;

txt=strcat('PSNR= ',num2str(PSNR_P10_AVG),', MSE= ',num2str(MSE_P10_AVG));

annotation(hf, 'textbox', pos, 'String', num2str(txt), 'vert', 'bottom', 'EdgeColor','none','FontSize',12,'FitBoxToText','on');

ha=subplot(3,6,11);imshow(FP_WNR1,[]);title('Weiner filter','Fontsize',12);colormap jet;

pos = get(ha, 'position');pos(2)=pos(2)-0.05;

txt=strcat('PSNR= ',num2str(PSNR_P10_WNR),', MSE= ',num2str(MSE_P10_WNR));

annotation(hf, 'textbox', pos, 'String', num2str(txt), 'vert', 'bottom', 'EdgeColor','none','FontSize',12,'FitBoxToText','on');

ha=subplot(3,6,12);imshow(FP_MMW1,[]);title('MMWF filer','Fontsize',12);colormap jet;

pos = get(ha, 'position');pos(2)=pos(2)-0.05;

txt=strcat('PSNR= ',num2str(PSNR_P10_MMW),', MSE= ',num2str(MSE_P10_MMW));

annotation(hf, 'textbox', pos, 'String', num2str(txt), 'vert', 'bottom', 'EdgeColor','none','FontSize',12,'FitBoxToText','on');

subplot(3,6,13);imshow(imvol,[]);title('Original','Fontsize',12);colormap jet;

subplot(3,6,14);imshow(NS1,[]);title('Speckle noise 10%','Fontsize',12);colormap jet;

ha=subplot(3,6,15);imshow(FS_MED1,[]);title('Median filter','Fontsize',12);colormap jet;

pos = get(ha, 'position');pos(2)=pos(2)-0.05;

txt=strcat('PSNR= ',num2str(PSNR_S10_MED),', MSE= ',num2str(MSE_S10_MED));

annotation(hf, 'textbox', pos, 'String', num2str(txt), 'vert', 'bottom', 'EdgeColor','none','FontSize',12,'FitBoxToText','on');

ha=subplot(3,6,16);imshow(FS_AVG1,[]);title('Mean filter','Fontsize',12);colormap jet;

pos = get(ha, 'position');pos(2)=pos(2)-0.05;

txt=strcat('PSNR= ',num2str(PSNR_S10_AVG),', MSE= ',num2str(MSE_S10_AVG));

annotation(hf, 'textbox', pos, 'String', num2str(txt), 'vert', 'bottom', 'EdgeColor','none','FontSize',12,'FitBoxToText','on');

ha=subplot(3,6,17);imshow(FS_WNR1,[]);title('Weiner filter','Fontsize',12);colormap jet;

pos = get(ha, 'position');pos(2)=pos(2)-0.05;

txt=strcat('PSNR= ',num2str(PSNR_S10_WNR),', MSE= ',num2str(MSE_S10_WNR));

annotation(hf, 'textbox', pos, 'String', num2str(txt), 'vert', 'bottom', 'EdgeColor','none','FontSize',12,'FitBoxToText','on');

ha=subplot(3,6,18);imshow(FS_MMW1,[]);title('MMWF filer','Fontsize',12);colormap jet;

pos = get(ha, 'position');pos(2)=pos(2)-0.05;

txt=strcat('PSNR= ',num2str(PSNR_S10_MMW),', MSE= ',num2str(MSE_S10_MMW));

annotation(hf, 'textbox', pos, 'String', num2str(txt), 'vert', 'bottom', 'EdgeColor','none','FontSize',12,'FitBoxToText','on');

% show 30% noise and filtering results

hf=figure

subplot(3,6,1),imshow(imvol,[]);title('Original','Fontsize',12);colormap jet;

subplot(3,6,2),imshow(NG3,[]);title('Gaussian noise 30%','Fontsize',12);colormap jet;

ha=subplot(3,6,3),imshow(FG_MED3,[]);title('Median filter','Fontsize',12);colormap jet;

pos = get(ha, 'position');pos(2)=pos(2)-0.05;

txt=strcat('PSNR= ',num2str(PSNR_G30_MED),', MSE= ',num2str(MSE_G30_MED));

annotation(hf, 'textbox', pos, 'String', num2str(txt), 'vert', 'bottom', 'EdgeColor','none','FontSize',12,'FitBoxToText','on');

ha=subplot(3,6,4),imshow(FG_AVG3,[]);title('Mean filter','Fontsize',12);colormap jet;

pos = get(ha, 'position');pos(2)=pos(2)-0.05;

txt=strcat('PSNR= ',num2str(PSNR_G30_AVG),', MSE= ',num2str(MSE_G30_AVG));

annotation(hf, 'textbox', pos, 'String', num2str(txt), 'vert', 'bottom', 'EdgeColor','none','FontSize',12,'FitBoxToText','on');

ha=subplot(3,6,5);imshow(FG_WNR3,[]);title('Weiner filter','Fontsize',12);colormap jet;

pos = get(ha, 'position');pos(2)=pos(2)-0.05;

txt=strcat('PSNR= ',num2str(PSNR_G30_WNR),', MSE= ',num2str(MSE_G30_WNR));

annotation(hf, 'textbox', pos, 'String', num2str(txt), 'vert', 'bottom', 'EdgeColor','none','FontSize',12,'FitBoxToText','on');

ha=subplot(3,6,6);imshow(FG_MMW3,[]);title('MMWF filer','Fontsize',12);colormap jet;

pos = get(ha, 'position');pos(2)=pos(2)-0.05;

txt=strcat('PSNR= ',num2str(PSNR_G30_MMW),', MSE= ',num2str(MSE_G30_MMW));

annotation(hf, 'textbox', pos, 'String', num2str(txt), 'vert', 'bottom', 'EdgeColor','none','FontSize',12,'FitBoxToText','on');

subplot(3,6,7);imshow(imvol,[]);title('Original','Fontsize',12);colormap jet;

subplot(3,6,8);imshow(NP6,[]);title(["Possion noise=","imnoise(Pixel Value*1.3)"],'Fontsize',12);colormap jet;

ha=subplot(3,6,9);imshow(FP_MED3,[]);title('Median filter','Fontsize',12);colormap jet;

pos = get(ha, 'position');pos(2)=pos(2)-0.05;

txt=strcat('PSNR= ',num2str(PSNR_P30_MED),', MSE= ',num2str(MSE_P30_MED));

annotation(hf, 'textbox', pos, 'String', num2str(txt), 'vert', 'bottom', 'EdgeColor','none','FontSize',12,'FitBoxToText','on');

ha=subplot(3,6,10);imshow(FP_AVG3,[]);title('Mean filter','Fontsize',12);colormap jet;

pos = get(ha, 'position');pos(2)=pos(2)-0.05;

txt=strcat('PSNR= ',num2str(PSNR_P30_AVG),', MSE= ',num2str(MSE_P30_AVG));

annotation(hf, 'textbox', pos, 'String', num2str(txt), 'vert', 'bottom', 'EdgeColor','none','FontSize',12,'FitBoxToText','on');

ha=subplot(3,6,11);imshow(FP_WNR3,[]);title('Weiner filter','Fontsize',12);colormap jet;

pos = get(ha, 'position');pos(2)=pos(2)-0.05;

txt=strcat('PSNR= ',num2str(PSNR_P30_WNR),', MSE= ',num2str(MSE_P30_WNR));

annotation(hf, 'textbox', pos, 'String', num2str(txt), 'vert', 'bottom', 'EdgeColor','none','FontSize',12,'FitBoxToText','on');

ha=subplot(3,6,12);imshow(FP_MMW3,[]);title('MMWF filer','Fontsize',12);colormap jet;

pos = get(ha, 'position');pos(2)=pos(2)-0.05;

txt=strcat('PSNR= ',num2str(PSNR_P30_MMW),', MSE= ',num2str(MSE_P30_MMW));

annotation(hf, 'textbox', pos, 'String', num2str(txt), 'vert', 'bottom', 'EdgeColor','none','FontSize',12,'FitBoxToText','on');

subplot(3,6,13);imshow(imvol,[]);title('Original','Fontsize',12);colormap jet;

subplot(3,6,14);imshow(NS3,[]);title('Speckle noise 30%','Fontsize',12);colormap jet;

ha=subplot(3,6,15);imshow(FS_MED3,[]);title('Median filter','Fontsize',12);colormap jet;

pos = get(ha, 'position');pos(2)=pos(2)-0.05;

txt=strcat('PSNR= ',num2str(PSNR_S30_MED),', MSE= ',num2str(MSE_S30_MED));

annotation(hf, 'textbox', pos, 'String', num2str(txt), 'vert', 'bottom', 'EdgeColor','none','FontSize',12,'FitBoxToText','on');

ha=subplot(3,6,16);imshow(FS_AVG3,[]);title('Mean filter','Fontsize',12);colormap jet;

pos = get(ha, 'position');pos(2)=pos(2)-0.05;

txt=strcat('PSNR= ',num2str(PSNR_S30_AVG),', MSE= ',num2str(MSE_S30_AVG));

annotation(hf, 'textbox', pos, 'String', num2str(txt), 'vert', 'bottom', 'EdgeColor','none','FontSize',12,'FitBoxToText','on');

ha=subplot(3,6,17);imshow(FS_WNR3,[]);title('Weiner filter','Fontsize',12);colormap jet;

pos = get(ha, 'position');pos(2)=pos(2)-0.05;

txt=strcat('PSNR= ',num2str(PSNR_S30_WNR),', MSE= ',num2str(MSE_S30_WNR));

annotation(hf, 'textbox', pos, 'String', num2str(txt), 'vert', 'bottom', 'EdgeColor','none','FontSize',12,'FitBoxToText','on');

ha=subplot(3,6,18);imshow(FS_MMW3,[]);title('MMWF filer','Fontsize',12);colormap jet;

pos = get(ha, 'position');pos(2)=pos(2)-0.05;

txt=strcat('PSNR= ',num2str(PSNR_S30_MMW),', MSE= ',num2str(MSE_S30_MMW));

annotation(hf, 'textbox', pos, 'String', num2str(txt), 'vert', 'bottom', 'EdgeColor','none','FontSize',12,'FitBoxToText','on');

% show 60% noise and filtering results

hf=figure

subplot(3,6,1);imshow(imvol,[]);title('Original','Fontsize',12);colormap jet;

subplot(3,6,2);imshow(NG6,[]);title('Gaussian noise 60%','Fontsize',12);colormap jet;

ha=subplot(3,6,3);imshow(FG_MED6,[]);title('Median filter','Fontsize',12);colormap jet;

pos = get(ha, 'position');pos(2)=pos(2)-0.05;

txt=strcat('PSNR= ',num2str(PSNR_G60_MED),', MSE= ',num2str(MSE_G60_MED));

annotation(hf, 'textbox', pos, 'String', num2str(txt), 'vert', 'bottom', 'EdgeColor','none','FontSize',12,'FitBoxToText','on');

ha=subplot(3,6,4);imshow(FG_AVG6,[]);title('Mean filter','Fontsize',12);colormap jet;

pos = get(ha, 'position');pos(2)=pos(2)-0.05;

txt=strcat('PSNR= ',num2str(PSNR_G60_AVG),', MSE= ',num2str(MSE_G60_AVG));

annotation(hf, 'textbox', pos, 'String', num2str(txt), 'vert', 'bottom', 'EdgeColor','none','FontSize',12,'FitBoxToText','on');

ha=subplot(3,6,5);imshow(FG_WNR6,[]);title('Weiner filter','Fontsize',12);colormap jet;

pos = get(ha, 'position');pos(2)=pos(2)-0.05;

txt=strcat('PSNR= ',num2str(PSNR_G60_WNR),', MSE= ',num2str(MSE_G60_WNR));

annotation(hf, 'textbox', pos, 'String', num2str(txt), 'vert', 'bottom', 'EdgeColor','none','FontSize',12,'FitBoxToText','on');

ha=subplot(3,6,6);imshow(FG_MMW6,[]);title('MMWF filer','Fontsize',12);colormap jet;

pos = get(ha, 'position');pos(2)=pos(2)-0.05;

txt=strcat('PSNR= ',num2str(PSNR_G60_MMW),', MSE= ',num2str(MSE_G60_MMW));

annotation(hf, 'textbox', pos, 'String', num2str(txt), 'vert', 'bottom', 'EdgeColor','none','FontSize',12,'FitBoxToText','on');

subplot(3,6,7);imshow(imvol,[]);title('Original','Fontsize',12);colormap jet;

subplot(3,6,8);imshow(NP6,[]);title(["Possion noise=","imnoise(Pixel Value*1.6)"],'Fontsize',12);colormap jet;

ha=subplot(3,6,9);imshow(FP_MED6,[]);title('Median filter','Fontsize',12);colormap jet;

pos = get(ha, 'position');pos(2)=pos(2)-0.05;

txt=strcat('PSNR= ',num2str(PSNR_P60_MED),', MSE= ',num2str(MSE_P60_MED));

annotation(hf, 'textbox', pos, 'String', num2str(txt), 'vert', 'bottom', 'EdgeColor','none','FontSize',12,'FitBoxToText','on');

ha=subplot(3,6,10);imshow(FP_AVG6,[]);title('Mean filter','Fontsize',12);colormap jet;

pos = get(ha, 'position');pos(2)=pos(2)-0.05;

txt=strcat('PSNR= ',num2str(PSNR_P60_AVG),', MSE= ',num2str(MSE_P60_AVG));

annotation(hf, 'textbox', pos, 'String', num2str(txt), 'vert', 'bottom', 'EdgeColor','none','FontSize',12,'FitBoxToText','on');

ha=subplot(3,6,11);imshow(FP_WNR6,[]);title('Weiner filter','Fontsize',12);colormap jet;

pos = get(ha, 'position');pos(2)=pos(2)-0.05;

txt=strcat('PSNR= ',num2str(PSNR_P60_WNR),', MSE= ',num2str(MSE_P60_WNR));

annotation(hf, 'textbox', pos, 'String', num2str(txt), 'vert', 'bottom', 'EdgeColor','none','FontSize',12,'FitBoxToText','on');

ha=subplot(3,6,12);imshow(FP_MMW6,[]);title('MMWF filer','Fontsize',12);colormap jet;

pos = get(ha, 'position');pos(2)=pos(2)-0.05;

txt=strcat('PSNR= ',num2str(PSNR_P60_MMW),', MSE= ',num2str(MSE_P60_MMW));

annotation(hf, 'textbox', pos, 'String', num2str(txt), 'vert', 'bottom', 'EdgeColor','none','FontSize',12,'FitBoxToText','on');

subplot(3,6,13);imshow(imvol,[]);title('Original','Fontsize',12);colormap jet;

subplot(3,6,14);imshow(NS6,[]);title('Speckle noise 60%','Fontsize',12);colormap jet;

ha=subplot(3,6,15);imshow(FS_MED6,[]);title('Median filter','Fontsize',12);colormap jet;

pos = get(ha, 'position');pos(2)=pos(2)-0.05;

txt=strcat('PSNR= ',num2str(PSNR_S60_MED),', MSE= ',num2str(MSE_S60_MED));

annotation(hf, 'textbox', pos, 'String', num2str(txt), 'vert', 'bottom', 'EdgeColor','none','FontSize',12,'FitBoxToText','on');

ha=subplot(3,6,16);imshow(FS_AVG6,[]);title('Mean filter','Fontsize',12);colormap jet;

pos = get(ha, 'position');pos(2)=pos(2)-0.05;

txt=strcat('PSNR= ',num2str(PSNR_S60_AVG),', MSE= ',num2str(MSE_S60_AVG));

annotation(hf, 'textbox', pos, 'String', num2str(txt), 'vert', 'bottom', 'EdgeColor','none','FontSize',12,'FitBoxToText','on');

ha=subplot(3,6,17);imshow(FS_WNR6,[]);title('Weiner filter','Fontsize',12);colormap jet;

pos = get(ha, 'position');pos(2)=pos(2)-0.05;

txt=strcat('PSNR= ',num2str(PSNR_S60_WNR),', MSE= ',num2str(MSE_S60_WNR));

annotation(hf, 'textbox', pos, 'String', num2str(txt), 'vert', 'bottom', 'EdgeColor','none','FontSize',12,'FitBoxToText','on');

ha=subplot(3,6,18);imshow(FS_MMW6,[]);title('MMWF filer','Fontsize',12);colormap jet;

pos = get(ha, 'position');pos(2)=pos(2)-0.05;

txt=strcat('PSNR= ',num2str(PSNR_S60_MMW),', MSE= ',num2str(MSE_S60_MMW));

annotation(hf, 'textbox', pos, 'String', num2str(txt), 'vert', 'bottom', 'EdgeColor','none','FontSize',12,'FitBoxToText','on');

%% Export to excel sheet

% PSNR

filename = 'Filtering.xlsx';

T1 = table({PSNR_G10_MED},{PSNR_G20_MED},{PSNR_G30_MED},{PSNR_G40_MED},{PSNR_G50_MED},{PSNR_G60_MED});

writetable(T1,filename,'Sheet','Sheet1','WriteVariableNames',false,'Range',strcat('B3:G3'));

T1 = table({PSNR_G10_AVG},{PSNR_G20_AVG},{PSNR_G30_AVG},{PSNR_G40_AVG},{PSNR_G50_AVG},{PSNR_G60_AVG});

writetable(T1,filename,'Sheet','Sheet1','WriteVariableNames',false,'Range',strcat('B4:G4'));

T1 = table({PSNR_G10_WNR},{PSNR_G20_WNR},{PSNR_G30_WNR},{PSNR_G40_WNR},{PSNR_G50_WNR},{PSNR_G60_WNR});

writetable(T1,filename,'Sheet','Sheet1','WriteVariableNames',false,'Range',strcat('B5:G5'));

T1 = table({PSNR_G10_MMW},{PSNR_G20_MMW},{PSNR_G30_MMW},{PSNR_G40_MMW},{PSNR_G50_MMW},{PSNR_G60_MMW});

writetable(T1,filename,'Sheet','Sheet1','WriteVariableNames',false,'Range',strcat('B6:G6'));

%

T1 = table({PSNR_P10_MED},{PSNR_P20_MED},{PSNR_P30_MED},{PSNR_P40_MED},{PSNR_P50_MED},{PSNR_P60_MED});

writetable(T1,filename,'Sheet','Sheet1','WriteVariableNames',false,'Range',strcat('B10:G10'));

T1 = table({PSNR_P10_AVG},{PSNR_P20_AVG},{PSNR_P30_AVG},{PSNR_P40_AVG},{PSNR_P50_AVG},{PSNR_P60_AVG});

writetable(T1,filename,'Sheet','Sheet1','WriteVariableNames',false,'Range',strcat('B11:G11'));

T1 = table({PSNR_P10_WNR},{PSNR_P20_WNR},{PSNR_P30_WNR},{PSNR_P40_WNR},{PSNR_P50_WNR},{PSNR_P60_WNR});

writetable(T1,filename,'Sheet','Sheet1','WriteVariableNames',false,'Range',strcat('B12:G12'));

T1 = table({PSNR_P10_MMW},{PSNR_P20_MMW},{PSNR_P30_MMW},{PSNR_P40_MMW},{PSNR_P50_MMW},{PSNR_P60_MMW});

writetable(T1,filename,'Sheet','Sheet1','WriteVariableNames',false,'Range',strcat('B13:G13'));

%

T1 = table({PSNR_S10_MED},{PSNR_S20_MED},{PSNR_S30_MED},{PSNR_S40_MED},{PSNR_S50_MED},{PSNR_S60_MED});

writetable(T1,filename,'Sheet','Sheet1','WriteVariableNames',false,'Range',strcat('B17:G17'));

T1 = table({PSNR_S10_AVG},{PSNR_S20_AVG},{PSNR_S30_AVG},{PSNR_S40_AVG},{PSNR_S50_AVG},{PSNR_S60_AVG});

writetable(T1,filename,'Sheet','Sheet1','WriteVariableNames',false,'Range',strcat('B18:G18'));

T1 = table({PSNR_S10_WNR},{PSNR_S20_WNR},{PSNR_S30_WNR},{PSNR_S40_WNR},{PSNR_S50_WNR},{PSNR_S60_WNR});

writetable(T1,filename,'Sheet','Sheet1','WriteVariableNames',false,'Range',strcat('B19:G19'));

T1 = table({PSNR_S10_MMW},{PSNR_S20_MMW},{PSNR_S30_MMW},{PSNR_S40_MMW},{PSNR_S50_MMW},{PSNR_S60_MMW});

writetable(T1,filename,'Sheet','Sheet1','WriteVariableNames',false,'Range',strcat('B20:G20'));

% MSE

filename = 'Filtering.xlsx';

T1 = table({MSE_G10_MED},{MSE_G20_MED},{MSE_G30_MED},{MSE_G40_MED},{MSE_G50_MED},{MSE_G60_MED});

writetable(T1,filename,'Sheet','Sheet1','WriteVariableNames',false,'Range',strcat('K3:P3'));

T1 = table({MSE_G10_AVG},{MSE_G20_AVG},{MSE_G30_AVG},{MSE_G40_AVG},{MSE_G50_AVG},{MSE_G60_AVG});

writetable(T1,filename,'Sheet','Sheet1','WriteVariableNames',false,'Range',strcat('K4:P4'));

T1 = table({MSE_G10_WNR},{MSE_G20_WNR},{MSE_G30_WNR},{MSE_G40_WNR},{MSE_G50_WNR},{MSE_G60_WNR});

writetable(T1,filename,'Sheet','Sheet1','WriteVariableNames',false,'Range',strcat('K5:P5'));

T1 = table({MSE_G10_MMW},{MSE_G20_MMW},{MSE_G30_MMW},{MSE_G40_MMW},{MSE_G50_MMW},{MSE_G60_MMW});

writetable(T1,filename,'Sheet','Sheet1','WriteVariableNames',false,'Range',strcat('K6:P6'));

%

T1 = table({MSE_P10_MED},{MSE_P20_MED},{MSE_P30_MED},{MSE_P40_MED},{MSE_P50_MED},{MSE_P60_MED});

writetable(T1,filename,'Sheet','Sheet1','WriteVariableNames',false,'Range',strcat('K10:P10'));

T1 = table({MSE_P10_AVG},{MSE_P20_AVG},{MSE_P30_AVG},{MSE_P40_AVG},{MSE_P50_AVG},{MSE_P60_AVG});

writetable(T1,filename,'Sheet','Sheet1','WriteVariableNames',false,'Range',strcat('K11:P11'));

T1 = table({MSE_P10_WNR},{MSE_P20_WNR},{MSE_P30_WNR},{MSE_P40_WNR},{MSE_P50_WNR},{MSE_P60_WNR});

writetable(T1,filename,'Sheet','Sheet1','WriteVariableNames',false,'Range',strcat('K12:P12'));

T1 = table({MSE_P10_MMW},{MSE_P20_MMW},{MSE_P30_MMW},{MSE_P40_MMW},{MSE_P50_MMW},{MSE_P60_MMW});

writetable(T1,filename,'Sheet','Sheet1','WriteVariableNames',false,'Range',strcat('K13:P13'));

%

T1 = table({MSE_S10_MED},{MSE_S20_MED},{MSE_S30_MED},{MSE_S40_MED},{MSE_S50_MED},{MSE_S60_MED});

writetable(T1,filename,'Sheet','Sheet1','WriteVariableNames',false,'Range',strcat('K17:P17'));

T1 = table({MSE_S10_AVG},{MSE_S20_AVG},{MSE_S30_AVG},{MSE_S40_AVG},{MSE_S50_AVG},{MSE_S60_AVG});

writetable(T1,filename,'Sheet','Sheet1','WriteVariableNames',false,'Range',strcat('K18:P18'));

T1 = table({MSE_S10_WNR},{MSE_S20_WNR},{MSE_S30_WNR},{MSE_S40_WNR},{MSE_S50_WNR},{MSE_S60_WNR});

writetable(T1,filename,'Sheet','Sheet1','WriteVariableNames',false,'Range',strcat('K19:P19'));

T1 = table({MSE_S10_MMW},{MSE_S20_MMW},{MSE_S30_MMW},{MSE_S40_MMW},{MSE_S50_MMW},{MSE_S60_MMW});

writetable(T1,filename,'Sheet','Sheet1','WriteVariableNames',false,'Range',strcat('K20:P20'));

disp('Data has been written to excel sheet, check Filtering.xlsx in the current folder!')

| **M-file name** | MMWF_2D.m |
| --- | --- |
| **Type** | Fun |
| **Description** | Function to apply the modified median wiener filter. |
| **comments** | Open source: designed by Carlo Vittorio Cannistraci 2014 |

| **M-file name** | elongation.m |
| --- | --- |
| **Type** | script |
| **Description** | code to compute elongation of specific ROI of RNA image. |
| **comments** | The code is written by us using a modified methodology described in the following paper (248) |

**Code:**

%% code to compute elongation

x = inputdlg('Pick Post image:',...

'Define Image', [1]);

name= (x{:}); % image number

imvol = squeeze(dicomread(name));

info = dicominfo(name);

save info info;

for i=1:1:info.NumberOfFrames

imvol(:,:,i)=medfilt2(imvol(:,:,i),[3 3]);%

end

figure,imshow(sum(imvol,3),[]); title('Select seed point');

colormap jet;colorbar

sp = ginput(1);

[All_masks,B, C, D,min_img2,F,imvolmn2,bw1, AA, AB, AC, AD, AE, AF,AG, AH, AI, aj, sp]=max_min_MUGA3(imvol,0,sp,[],[]);

bw1=uint16(bw1);

imvol1=All_masks(:,:,1);

imvol2=All_masks(:,:,3);

imvol3=All_masks(:,:,5);

imvol4=All_masks(:,:,7);

h1=2; h2=2; h3=2; h4=2; h5=2; % we took image i=1, then image i=3, then i=5, then i=7;

S1=regionprops(bwlabel(imvol1),'MajorAxisLength');

d1=[S1.MajorAxisLength];

S2=regionprops(bwlabel(imvol2),'MajorAxisLength');

d2=[S2.MajorAxisLength];

S3=regionprops(bwlabel(imvol3),'MajorAxisLength');

d3=[S3.MajorAxisLength];

S4=regionprops(bwlabel(imvol4),'MajorAxisLength');

d4=[S4.MajorAxisLength];

r1=round(d1/2); r2=round(d2/2); r3=round(d3/2); r4=round(d4/2); r5=0;

l12=sqrt(h1^2+(r1-r2)^2);

l23=sqrt(h2^2+(r2-r3)^2);

l34=sqrt(h3^2+(r3-r4)^2);

l45=sqrt(h4^2+(r4-0)^2);

s1=pi*(r1+r2)*l12;

s2=pi*(r2+r3)*l23;

s3=pi*(r3+r4)*l34;

s4=pi*(r4+r5)*l45;

SLV_exp=s1+s2+s3+s4;

v1=(pi/3)*(h1)*(r1^2+r1*r2+r2^2);

v2=(pi/3)*(h2)*(r2^2+r2*r3+r3^2);

v3=(pi/3)*(h3)*(r3^2+r3*r4+r4^2);

v4=(pi/3)*(h4)*(r4^2+r4*r5+r5^2);

VLV=v1+v2+v3+v4;

SLV_theor=4*pi*((3*VLV/4*pi)^(1/3))^2;

% Elongation

ELO=-(SLV_exp-SLV_theor)/SLV_theor

%%%%%%%%%%%%%%%%%%%%%%%%%%%%%%%%%%%%%%%%%%%%%%%%%%%%%%%%%%%%%%%%

| **M-file name** | Image_Entropy_ Experiment_ New_Version_ Possion_ Gaussian |
| --- | --- |
| **Type** | Script |
| **Description** | Experiment to compute the entropy, ApEn, Bounded ApEn and Synchrony using **poisson, Gaussian and speckle** noise under different noise range.  This file writes in ImageNoise EntropyExperimentPoisson.xlsx |
| **comments** | Novel: Written: 31-12-2021 |

**Code:**

%% Possion Entropy calculations

imvolx = squeeze(dicomread(name));

info = dicominfo(name);

for i=1:1:info.NumberOfFrames

imvolx(:,:,i)=medfilt2(imvolx(:,:,i),[5 5]);%

end

Tem=imvolx;

f1 = @(x) imnoise(x,'poisson');

I11=uint16(Tem.*sfac1);

I12=uint16(Tem.*sfac2);

I13=uint16(Tem.*sfac3);

I14=uint16(Tem.*sfac4);

I15=uint16(Tem.*sfac5);

I16=uint16(Tem.*sfac6);

for i=1:16

PP1(:,:,i)=double((1/sfac1)*roifilt2(I11(:,:,i),bw1,f1));

PP2(:,:,i)=double((1/sfac2)*roifilt2(I12(:,:,i),bw1,f1));

PP3(:,:,i)=double((1/sfac3)*roifilt2(I13(:,:,i),bw1,f1));

PP4(:,:,i)=double((1/sfac4)*roifilt2(I14(:,:,i),bw1,f1));

PP5(:,:,i)=double((1/sfac5)*roifilt2(I15(:,:,i),bw1,f1));

PP6(:,:,i)=double((1/sfac6)*roifilt2(I16(:,:,i),bw1,f1));

end

[xxx syn1]=computeEntropySynchronyNo(Tem);

[xxx syn2]=computeEntropySynchronyNo(PP1);

[xxx syn3]=computeEntropySynchronyNo(PP2);

[xxx syn4]=computeEntropySynchronyNo(PP3);

[xxx syn5]=computeEntropySynchronyNo(PP4);

[xxx syn6]=computeEntropySynchronyNo(PP5);

[xxx syn7]=computeEntropySynchronyNo(PP6);

m_val=2; r_val=1;

I11=imvol;

I12 = NP1;

I13 = NP2;

I14 = NP3;

I15 = NP4;

I16 = NP5;

I17 = NP6;

% bw1=bw2

s1=double(I11(bw1));

s2=(I12(bw1));

s3=(I13(bw1));

s4=(I14(bw1));

s5=(I15(bw1));

s6=(I16(bw1));

s7=(I17(bw1));

%[,Sync_post]=computeEntropySynchrony(s1,-1,-1,0)

[Ent1 ]=EntropySig(s1);

[Ent2 ]=EntropySig(s2);

[Ent3 ]=EntropySig(s3);

[Ent4 ]=EntropySig(s4);

[Ent5 ]=EntropySig(s5);

[Ent6 ]=EntropySig(s6);

[Ent7 ]=EntropySig(s7);

ApEnPost1= ApEn_slow(s1, m_val,r_val*std2(s1));

ApEnPost2= ApEn_slow(s2, m_val,r_val*std2(s2));

ApEnPost3= ApEn_slow(s3, m_val,r_val*std2(s3));

ApEnPost4= ApEn_slow(s4, m_val,r_val*std2(s4));

ApEnPost5= ApEn_slow(s5, m_val,r_val*std2(s5));

ApEnPost6= ApEn_slow(s6, m_val,r_val*std2(s6));

ApEnPost7= ApEn_slow(s7, m_val,r_val*std2(s7));

[BoundApEnPost1,epsilonPost1,LspsilonPost1]=BoundedProcess(s1);

[BoundApEnPost2,epsilonPost2,LspsilonPost2]=BoundedProcess(s2);

[BoundApEnPost3,epsilonPost3,LspsilonPost3]=BoundedProcess(s3);

[BoundApEnPost4,epsilonPost4,LspsilonPost4]=BoundedProcess(s4);

[BoundApEnPost5,epsilonPost5,LspsilonPost5]=BoundedProcess(s5);

[BoundApEnPost6,epsilonPost6,LspsilonPost6]=BoundedProcess(s6);

[BoundApEnPost7,epsilonPost7,LspsilonPost7]=BoundedProcess(s7);

[Ent1 Ent2 Ent3 Ent4 Ent5 Ent6 Ent7]

[ApEnPost1 ApEnPost2 ApEnPost3 ApEnPost4 ApEnPost5 ApEnPost6 ApEnPost7]

[BoundApEnPost1 BoundApEnPost2 BoundApEnPost3 BoundApEnPost4 BoundApEnPost5 BoundApEnPost6 BoundApEnPost7]

filename = 'ImageNoiseEntropyExperiment.xlsx';

T1 = table({Ent1},{Ent2},{Ent3},{Ent4},{Ent5},{Ent6},{Ent7});

%system('taskkill /F /IM EXCEL.EXE');

writetable(T1,filename,'Sheet','Sheet1','WriteVariableNames',false,'Range',strcat('B2:H2'));

T1 = table({ApEnPost1},{ApEnPost2},{ApEnPost3},{ApEnPost4},{ApEnPost5},{ApEnPost6},{ApEnPost7});

%system('taskkill /F /IM EXCEL.EXE');

writetable(T1,filename,'Sheet','Sheet1','WriteVariableNames',false,'Range',strcat('B3:H3'));

T1 = table({BoundApEnPost1},{BoundApEnPost2},{BoundApEnPost3},{BoundApEnPost4},{BoundApEnPost5},{BoundApEnPost6},{BoundApEnPost7});

%system('taskkill /F /IM EXCEL.EXE');

writetable(T1,filename,'Sheet','Sheet1','WriteVariableNames',false,'Range',strcat('B4:H4'));

T1 = table({syn1},{syn2},{syn3},{syn4},{syn5},{syn6},{syn7});

%system('taskkill /F /IM EXCEL.EXE');

writetable(T1,filename,'Sheet','Sheet1','WriteVariableNames',false,'Range',strcat('B5:H5'));

disp('Data has been written to excel sheet, check ImageNoiseEntropyExperiment.xlsx in the current folder!');

%%

%% Gaussian Entropy calculations

Tem=imvolx;

for i=1:16

GG1(:,:,i)=imnoise(Tem(:,:,i).*uint16(bw1),'Gaussian',sfacG1);

GG2(:,:,i)=imnoise(Tem(:,:,i).*uint16(bw1),'Gaussian',sfacG2);

GG3(:,:,i)=imnoise(Tem(:,:,i).*uint16(bw1),'Gaussian',sfacG3);

GG4(:,:,i)=imnoise(Tem(:,:,i).*uint16(bw1),'Gaussian',sfacG4);

GG5(:,:,i)=imnoise(Tem(:,:,i).*uint16(bw1),'Gaussian',sfacG5);

GG6(:,:,i)=imnoise(Tem(:,:,i).*uint16(bw1),'Gaussian',sfacG6);

end

[xxx syn1]=computeEntropySynchronyNo(Tem);

[xxx syn2]=computeEntropySynchronyNo(GG1);

[xxx syn3]=computeEntropySynchronyNo(GG2);

[xxx syn4]=computeEntropySynchronyNo(GG3);

[xxx syn5]=computeEntropySynchronyNo(GG4);

[xxx syn6]=computeEntropySynchronyNo(GG5);

[xxx syn7]=computeEntropySynchronyNo(GG6);

m_val=2; r_val=1;

I111=imvol;

I112 = NG1;

I113 = NG2;

I114 = NG3;

I115 = NG4;

I116 = NG5;

I117 = NG6;

% bw1=bw2

s11=double(I111(bw1));

s12=double((I112(bw1)));

s13=double((I113(bw1)));

s14=double((I114(bw1)));

s15=double((I115(bw1)));

s16=double((I116(bw1)));

s17=double((I117(bw1)));

%[,Sync_post]=computeEntropySynchrony(s1,-1,-1,0)

[Ent1 ]=EntropySig(s11);

[Ent2 ]=EntropySig(s12);

[Ent3 ]=EntropySig(s13);

[Ent4 ]=EntropySig(s14);

[Ent5 ]=EntropySig(s15);

[Ent6 ]=EntropySig(s16);

[Ent7 ]=EntropySig(s17);

ApEnPost1= ApEn_slow(s1, m_val,r_val*std2(s11));

ApEnPost2= ApEn_slow(s2, m_val,r_val*std2(s12));

ApEnPost3= ApEn_slow(s3, m_val,r_val*std2(s13));

ApEnPost4= ApEn_slow(s4, m_val,r_val*std2(s14));

ApEnPost5= ApEn_slow(s5, m_val,r_val*std2(s15));

ApEnPost6= ApEn_slow(s6, m_val,r_val*std2(s16));

ApEnPost7= ApEn_slow(s7, m_val,r_val*std2(s17));

[BoundApEnPost1,epsilonPost1,LspsilonPost1]=BoundedProcess(s11);

[BoundApEnPost2,epsilonPost2,LspsilonPost2]=BoundedProcess(s12);

[BoundApEnPost3,epsilonPost3,LspsilonPost3]=BoundedProcess(s13);

[BoundApEnPost4,epsilonPost4,LspsilonPost4]=BoundedProcess(s14);

[BoundApEnPost5,epsilonPost5,LspsilonPost5]=BoundedProcess(s15);

[BoundApEnPost6,epsilonPost6,LspsilonPost6]=BoundedProcess(s16);

[BoundApEnPost7,epsilonPost7,LspsilonPost7]=BoundedProcess(s17);

[Ent1 Ent2 Ent3 Ent4 Ent5 Ent6 Ent7]

[ApEnPost1 ApEnPost2 ApEnPost3 ApEnPost4 ApEnPost5 ApEnPost6 ApEnPost7]

[BoundApEnPost1 BoundApEnPost2 BoundApEnPost3 BoundApEnPost4 BoundApEnPost5 BoundApEnPost6 BoundApEnPost7]

filename = 'ImageNoiseEntropyExperiment.xlsx';

T1 = table({Ent1},{Ent2},{Ent3},{Ent4},{Ent5},{Ent6},{Ent7});

%system('taskkill /F /IM EXCEL.EXE');

writetable(T1,filename,'Sheet','Sheet1','WriteVariableNames',false,'Range',strcat('B9:H9'));

T1 = table({ApEnPost1},{ApEnPost2},{ApEnPost3},{ApEnPost4},{ApEnPost5},{ApEnPost6},{ApEnPost7});

%system('taskkill /F /IM EXCEL.EXE');

writetable(T1,filename,'Sheet','Sheet1','WriteVariableNames',false,'Range',strcat('B10:H10'));

T1 = table({BoundApEnPost1},{BoundApEnPost2},{BoundApEnPost3},{BoundApEnPost4},{BoundApEnPost5},{BoundApEnPost6},{BoundApEnPost7});

%system('taskkill /F /IM EXCEL.EXE');

writetable(T1,filename,'Sheet','Sheet1','WriteVariableNames',false,'Range',strcat('B11:H11'));

T1 = table({syn1},{syn2},{syn3},{syn4},{syn5},{syn6},{syn7});

%system('taskkill /F /IM EXCEL.EXE');

writetable(T1,filename,'Sheet','Sheet1','WriteVariableNames',false,'Range',strcat('B12:H12'));

disp('Data has been written to excel sheet, check ImageNoiseEntropyExperiment.xlsx in the current folder!');

%%%%%%%%%%%%%%%%%%%%%%%%%%%%%%%%%%%%%%%%%%%%%%%%%%%%%%%%%%%%%%%%%%%

| **M-file name** | filtering_ experiment_ new_version_ fixed_noise_level |
| --- | --- |
| **Type** | Script |
| **Description** | Experiment to compute the entropy, ApEn, Bounded ApEn and Synchrony using **poisson, Gaussian and speckle** noise under different noise range.  This file writes in ImageNoise EntropyExperimentPoisson.xlsx. it uses fixed noise level (the code iterates many times until getting the same PSNR of all noisy images of the three noise types then apply the experiment). |
| **comments** | Novel: Written: 31-12-2021 |

**Code:**

clear all

close all

clc

% read images (post-chemo).

x = inputdlg('Pick Post image:',...

'Define Image', [1]);

name= (x{:}); % image number

imvol = squeeze(dicomread(name));

info = dicominfo(name);

for i=1:1:info.NumberOfFrames

imvol(:,:,i)=medfilt2(imvol(:,:,i),[5 5]);%

end

figure,imshow(sum(imvol,3),[]); title('Select seed point');

colormap jet;colorbar

sp = ginput(1);

[a,B, C, D,min_img2,F,imvolmn2,bw1, AA, AB, AC, AD, AE, AF,AG, AH, AI, aj, sp]=max_min_MUGA3(imvol,0,sp,[],[]);

close

imvol=uint16(sum(imvol,3));

mn=mean(imvol(bw1));

stdd=std2(imvol(bw1));

val1=stdd/mn;

% adding poisson noise to imvol

% if you work on a specific ROI you need to compute bw using max_min_muga

smax=max(imvol(bw1));

sfac1=double(400*val1)/double(smax);

sfac2=double(350*val1)/double(smax);

sfac3=double(200*val1)/double(smax);

sfac4=double(100*val1)/double(smax);

sfac5=double(75*val1)/double(smax);

sfac6=double(50*val1)/double(smax);

I1=imvol;

I11=uint16(imvol.*sfac1);

I12=uint16(imvol.*sfac2);

I13=uint16(imvol.*sfac3);

I14=uint16(imvol.*sfac4);

I15=uint16(imvol.*sfac5);

I16=uint16(imvol.*sfac6);

bw2=ones(size(I11));

f1 = @(x) imnoise(x,'poisson');

NP1=double((1/sfac1)*roifilt2(I11,bw2,f1));

NP2=double((1/sfac2)*roifilt2(I12,bw2,f1));

NP3=double((1/sfac3)*roifilt2(I13,bw2,f1));

NP4=double((1/sfac4)*roifilt2(I14,bw2,f1));

NP5=double((1/sfac5)*roifilt2(I15,bw2,f1));

NP6=double((1/sfac6)*roifilt2(I16,bw2,f1));

% adding gaussian noise to imvol

%f1 = @(x) imnoise(x,'Gaussian',0,10*val1);

%f2 = @(x) imnoise(x,'Gaussian',0,20*val1);

%f3 = @(x) imnoise(x,'Gaussian',0,30*val1);

%f4 = @(x) imnoise(x,'Gaussian',0,40*val1);

%f5 = @(x) imnoise(x,'Gaussian',0,50*val1);

%f6 = @(x) imnoise(x,'Gaussian',0,60*val1);

% NG1=roifilt2(I11,bw1,f1);

% imvol(bw1)=NG1(bw1);

% NG2=roifilt2(I11,bw1,f2);

% NG3=roifilt2(I11,bw1,f3);

% NG4=roifilt2(I11,bw1,f4);

% NG5=roifilt2(I11,bw1,f5);

% NG6=roifilt2(I11,bw1,f6);

psnrP1=psnr(uint16(NP1),imvol);

psnrP2=psnr(uint16(NP2),imvol);

psnrP3=psnr(uint16(NP3),imvol);

psnrP4=psnr(uint16(NP4),imvol);

psnrP5=psnr(uint16(NP5),imvol);

psnrP6=psnr(uint16(NP6),imvol);

psnrG1=0;psnrG2=0;psnrG3=0;psnrG4=0;psnrG5=0;psnrG6=0;

smax=max(imvol(bw1));

Exp_param1=0.00;

Exp_param2=0.00;

Exp_param3=0.00;

Exp_param4=0.00;

Exp_param5=0.00;

Exp_param6=0.00;

while (round(psnrP1)~=round(psnrG1))

sfacG1=double(Exp_param1*val1)/double(smax);

NG1=imnoise(imvol,'Gaussian',0,sfacG1);

psnrG1=psnr(NG1,imvol)

Exp_param1=Exp_param1+0.0001;

end

while (round(psnrP2)~=round(psnrG2))

sfacG2=double(Exp_param2*val1)/double(smax);

NG2=imnoise(imvol,'Gaussian',0,sfacG2);

psnrG2=psnr(NG2,imvol)

Exp_param2=Exp_param2+0.0001;

end

while (round(psnrP3)~=round(psnrG3))

sfacG3=double(Exp_param3*val1)/double(smax);

NG3=imnoise(imvol,'Gaussian',0,sfacG3);

psnrG3=psnr(NG3,imvol)

Exp_param3=Exp_param3+0.0001;

end

while (round(psnrP4)~=round(psnrG4))

sfacG4=double(Exp_param4*val1)/double(smax);

NG4=imnoise(imvol,'Gaussian',0,sfacG4);

psnrG4=psnr(NG4,imvol)

Exp_param4=Exp_param4+0.0001;

end

while (round(psnrP5)~=round(psnrG5))

sfacG5=double(Exp_param5*val1)/double(smax);

NG5=imnoise(imvol,'Gaussian',0,sfacG5);

psnrG5=psnr(NG5,imvol)

Exp_param5=Exp_param5+0.0001;

end

while (round(psnrP6)~=round(psnrG6))

sfacG6=double(Exp_param6*val1)/double(smax);

NG6=imnoise(imvol,'Gaussian',0,sfacG6);

psnrG6=psnr(NG6,imvol)

Exp_param6=Exp_param6+0.0001;

end

% adding speckle noise to imvol

Exp_param1=0.00;

Exp_param2=0.00;

Exp_param3=0.00;

Exp_param4=0.00;

Exp_param5=0.00;

Exp_param6=0.00;

psnrS1=0;psnrS2=0;psnrS3=0;psnrS4=0;psnrS5=0;psnrS6=0;

while (round(psnrP1)~=round(psnrS1))

sfacS1=double(Exp_param1*val1)/double(smax);

NS1=imnoise(imvol,'Speckle',sfacS1);

psnrS1=psnr(NS1,imvol)

Exp_param1=Exp_param1+0.1;

end

while (round(psnrP2)~=round(psnrS2))

sfacS2=double(Exp_param2*val1)/double(smax);

NS2=imnoise(imvol,'Speckle',sfacS2);

psnrS2=psnr(NS2,imvol)

Exp_param2=Exp_param2+0.1;

end

while (round(psnrP3)~=round(psnrS3))

sfacS3=double(Exp_param3*val1)/double(smax);

NS3=imnoise(imvol,'Speckle',sfacS3);

psnrS3=psnr(NS3,imvol)

Exp_param3=Exp_param3+0.1;

end

while (round(psnrP4)~=round(psnrS4))

sfacS4=double(Exp_param4*val1)/double(smax);

NS4=imnoise(imvol,'Speckle',sfacS4);

psnrS4=psnr(NS4,imvol)

Exp_param4=Exp_param4+0.1;

end

while (round(psnrP5)~=round(psnrS5))

sfacS5=double(Exp_param5*val1)/double(smax);

NS5=imnoise(imvol,'Speckle',sfacS5);

psnrS5=psnr(NS5,imvol)

Exp_param5=Exp_param5+0.1;

end

while (round(psnrP6)~=round(psnrS6))

sfacS6=double(Exp_param6*val1)/double(smax);

NS6=imnoise(imvol,'Speckle',sfacS6);

psnrS6=psnr(NS6,imvol)

Exp_param6=Exp_param6+0.1;

end

%% Filtering

%% 1st: filtering gaussian noise

% Median Filtering

FG_MED1=medfilt2(NG1,[5 5]); % FG means Filter gaussian noise, med means using median filter

FG_MED2=medfilt2(NG2,[5 5]);

FG_MED3=medfilt2(NG3,[5 5]);

FG_MED4=medfilt2(NG4,[5 5]);

FG_MED5=medfilt2(NG5,[5 5]);

FG_MED6=medfilt2(NG6,[5 5]);

% Mean Filtering

h1=fspecial('average',5);

FG_AVG1=filter2(h1,NG1); % FG means Filter gaussian noise, AVG means using mean (average) filter

FG_AVG2=filter2(h1,NG2);

FG_AVG3=filter2(h1,NG3);

FG_AVG4=filter2(h1,NG4);

FG_AVG5=filter2(h1,NG5);

FG_AVG6=filter2(h1,NG6);

% Wiener Filtering

FG_WNR1=wiener2(NG1,[5 5]); % FG means Filter gaussian noise, med means using weiner filter

FG_WNR2=wiener2(NG2,[5 5]);

FG_WNR3=wiener2(NG3,[5 5]);

FG_WNR4=wiener2(NG4,[5 5]);

FG_WNR5=wiener2(NG5,[5 5]);

FG_WNR6=wiener2(NG6,[5 5]);

% MMWF Filtering

FG_MMW1=MMWF_2D(NG1,5); % FG means Filter gaussian noise, med means using modified median weiner filter

FG_MMW2=MMWF_2D(NG2,5);

FG_MMW3=MMWF_2D(NG3,5);

FG_MMW4=MMWF_2D(NG4,5);

FG_MMW5=MMWF_2D(NG5,5);

FG_MMW6=MMWF_2D(NG6,5);

%% 2nd: filtering poisson noise

% Median Filtering

FP_MED1=medfilt2(NP1,[5 5]); % FP means Filter possion noise, med means using median filter

FP_MED2=medfilt2(NP2,[5 5]);

FP_MED3=medfilt2(NP3,[5 5]);

FP_MED4=medfilt2(NP4,[5 5]);

FP_MED5=medfilt2(NP5,[5 5]);

FP_MED6=medfilt2(NP6,[5 5]);

% Mean Filtering

h1=fspecial('average',5);

FP_AVG1=filter2(h1,NP1); % FP means Filter possion noise, AVG means using mean (average) filter

FP_AVG2=filter2(h1,NP2);

FP_AVG3=filter2(h1,NP3);

FP_AVG4=filter2(h1,NP4);

FP_AVG5=filter2(h1,NP5);

FP_AVG6=filter2(h1,NP6);

% Wiener Filtering

FP_WNR1=wiener2(NP1,[5 5]); % FP means Filter possion noise, med means using weiner filter

FP_WNR2=wiener2(NP2,[5 5]);

FP_WNR3=wiener2(NP3,[5 5]);

FP_WNR4=wiener2(NP4,[5 5]);

FP_WNR5=wiener2(NP5,[5 5]);

FP_WNR6=wiener2(NP6,[5 5]);

% MMWF Filtering

FP_MMW1=MMWF_2D(NP1,5); % FP means Filter possion noise, med means using modified median weiner filter

FP_MMW2=MMWF_2D(NP2,5);

FP_MMW3=MMWF_2D(NP3,5);

FP_MMW4=MMWF_2D(NP4,5);

FP_MMW5=MMWF_2D(NP5,5);

FP_MMW6=MMWF_2D(NP6,5);

%% 3rd: filtering speckle noise

% Median Filtering

FS_MED1=medfilt2(NS1,[5 5]); % FS means Filter speckle noise, med means using median filter

FS_MED2=medfilt2(NS2,[5 5]);

FS_MED3=medfilt2(NS3,[5 5]);

FS_MED4=medfilt2(NS4,[5 5]);

FS_MED5=medfilt2(NS5,[5 5]);

FS_MED6=medfilt2(NS6,[5 5]);

% Mean Filtering

h1=fspecial('average',5);

FS_AVG1=filter2(h1,NS1); % FS means Filter speckle noise, AVG means using mean (average) filter

FS_AVG2=filter2(h1,NS2);

FS_AVG3=filter2(h1,NS3);

FS_AVG4=filter2(h1,NS4);

FS_AVG5=filter2(h1,NS5);

FS_AVG6=filter2(h1,NS6);

% Wiener Filtering

FS_WNR1=wiener2(NS1,[5 5]); % FS means Filter speckle noise, med means using weiner filter

FS_WNR2=wiener2(NS2,[5 5]);

FS_WNR3=wiener2(NS3,[5 5]);

FS_WNR4=wiener2(NS4,[5 5]);

FS_WNR5=wiener2(NS5,[5 5]);

FS_WNR6=wiener2(NS6,[5 5]);

% MMWF Filtering

FS_MMW1=MMWF_2D(NS1,5); % FS means Filter speckle noise, med means using modified median weiner filter

FS_MMW2=MMWF_2D(NS2,5);

FS_MMW3=MMWF_2D(NS3,5);

FS_MMW4=MMWF_2D(NS4,5);

FS_MMW5=MMWF_2D(NS5,5);

FS_MMW6=MMWF_2D(NS6,5);

%% compute PSNR

%10%

PSNR_G10_MED= round(psnr(imvol, uint16(FG_MED1)));

PSNR_G10_AVG= round(psnr(imvol, uint16(FG_AVG1)));

PSNR_G10_WNR= round(psnr(imvol, uint16(FG_WNR1)));

PSNR_G10_MMW= round(psnr(imvol, uint16(FG_MMW1)));

PSNR_P10_MED= round(psnr(imvol, uint16(FP_MED1)));

PSNR_P10_AVG= round(psnr(imvol, uint16(FP_AVG1)));

PSNR_P10_WNR= round(psnr(imvol, uint16(FP_WNR1)));

PSNR_P10_MMW= round(psnr(imvol, uint16(FP_MMW1)));

PSNR_S10_MED= round(psnr(imvol, uint16(FS_MED1)));

PSNR_S10_AVG= round(psnr(imvol, uint16(FS_AVG1)));

PSNR_S10_WNR= round(psnr(imvol, uint16(FS_WNR1)));

PSNR_S10_MMW= round(psnr(imvol, uint16(FS_MMW1)));

%20%

PSNR_G20_MED= round(psnr(imvol, uint16(FG_MED2)));

PSNR_G20_AVG= round(psnr(imvol, uint16(FG_AVG2)));

PSNR_G20_WNR= round(psnr(imvol, uint16(FG_WNR2)));

PSNR_G20_MMW= round(psnr(imvol, uint16(FG_MMW2)));

PSNR_P20_MED= round(psnr(imvol, uint16(FP_MED2)));

PSNR_P20_AVG= round(psnr(imvol, uint16(FP_AVG2)));

PSNR_P20_WNR= round(psnr(imvol, uint16(FP_WNR2)));

PSNR_P20_MMW= round(psnr(imvol, uint16(FP_MMW2)));

PSNR_S20_MED= round(psnr(imvol, uint16(FS_MED2)));

PSNR_S20_AVG= round(psnr(imvol, uint16(FS_AVG2)));

PSNR_S20_WNR= round(psnr(imvol, uint16(FS_WNR2)));

PSNR_S20_MMW= round(psnr(imvol, uint16(FS_MMW2)));

%30%

PSNR_G30_MED= round(psnr(imvol, uint16(FG_MED3)));

PSNR_G30_AVG= round(psnr(imvol, uint16(FG_AVG3)));

PSNR_G30_WNR= round(psnr(imvol, uint16(FG_WNR3)));

PSNR_G30_MMW= round(psnr(imvol, uint16(FG_MMW3)));

PSNR_P30_MED= round(psnr(imvol, uint16(FP_MED3)));

PSNR_P30_AVG= round(psnr(imvol, uint16(FP_AVG3)));

PSNR_P30_WNR= round(psnr(imvol, uint16(FP_WNR3)));

PSNR_P30_MMW= round(psnr(imvol, uint16(FP_MMW3)));

PSNR_S30_MED= round(psnr(imvol, uint16(FS_MED3)));

PSNR_S30_AVG= round(psnr(imvol, uint16(FS_AVG3)));

PSNR_S30_WNR= round(psnr(imvol, uint16(FS_WNR3)));

PSNR_S30_MMW= round(psnr(imvol, uint16(FS_MMW3)));

%40%

PSNR_G40_MED= round(psnr(imvol, uint16(FG_MED4)));

PSNR_G40_AVG= round(psnr(imvol, uint16(FG_AVG4)));

PSNR_G40_WNR= round(psnr(imvol, uint16(FG_WNR4)));

PSNR_G40_MMW= round(psnr(imvol, uint16(FG_MMW4)));

PSNR_P40_MED= round(psnr(imvol, uint16(FP_MED4)));

PSNR_P40_AVG= round(psnr(imvol, uint16(FP_AVG4)));

PSNR_P40_WNR= round(psnr(imvol, uint16(FP_WNR4)));

PSNR_P40_MMW= round(psnr(imvol, uint16(FP_MMW4)));

PSNR_S40_MED= round(psnr(imvol, uint16(FS_MED4)));

PSNR_S40_AVG= round(psnr(imvol, uint16(FS_AVG4)));

PSNR_S40_WNR= round(psnr(imvol, uint16(FS_WNR4)));

PSNR_S40_MMW= round(psnr(imvol, uint16(FS_MMW4)));

%50%

PSNR_G50_MED= round(psnr(imvol, uint16(FG_MED5)));

PSNR_G50_AVG= round(psnr(imvol, uint16(FG_AVG5)));

PSNR_G50_WNR= round(psnr(imvol, uint16(FG_WNR5)));

PSNR_G50_MMW= round(psnr(imvol, uint16(FG_MMW5)));

PSNR_P50_MED= round(psnr(imvol, uint16(FP_MED5)));

PSNR_P50_AVG= round(psnr(imvol, uint16(FP_AVG5)));

PSNR_P50_WNR= round(psnr(imvol, uint16(FP_WNR5)));

PSNR_P50_MMW= round(psnr(imvol, uint16(FP_MMW5)));

PSNR_S50_MED= round(psnr(imvol, uint16(FS_MED5)));

PSNR_S50_AVG= round(psnr(imvol, uint16(FS_AVG5)));

PSNR_S50_WNR= round(psnr(imvol, uint16(FS_WNR5)));

PSNR_S50_MMW= round(psnr(imvol, uint16(FS_MMW5)));

%60%

PSNR_G60_MED= round(psnr(imvol, uint16(FG_MED6)));

PSNR_G60_AVG= round(psnr(imvol, uint16(FG_AVG6)));

PSNR_G60_WNR= round(psnr(imvol, uint16(FG_WNR6)));

PSNR_G60_MMW= round(psnr(imvol, uint16(FG_MMW6)));

PSNR_P60_MED= round(psnr(imvol, uint16(FP_MED6)));

PSNR_P60_AVG= round(psnr(imvol, uint16(FP_AVG6)));

PSNR_P60_WNR= round(psnr(imvol, uint16(FP_WNR6)));

PSNR_P60_MMW= round(psnr(imvol, uint16(FP_MMW6)));

PSNR_S60_MED= round(psnr(imvol, uint16(FS_MED6)));

PSNR_S60_AVG= round(psnr(imvol, uint16(FS_AVG6)));

PSNR_S60_WNR= round(psnr(imvol, uint16(FS_WNR6)));

PSNR_S60_MMW= round(psnr(imvol, uint16(FS_MMW6)));

%% Compute MSE

%10%

MSE_G10_MED= round(immse(imvol, uint16(FG_MED1)));

MSE_G10_AVG= round(immse(imvol, uint16(FG_AVG1)));

MSE_G10_WNR= round(immse(imvol, uint16(FG_WNR1)));

MSE_G10_MMW= round(immse(imvol, uint16(FG_MMW1)));

MSE_P10_MED= round(immse(imvol, uint16(FP_MED1)));

MSE_P10_AVG= round(immse(imvol, uint16(FP_AVG1)));

MSE_P10_WNR= round(immse(imvol, uint16(FP_WNR1)));

MSE_P10_MMW= round(immse(imvol, uint16(FP_MMW1)));

MSE_S10_MED= round(immse(imvol, uint16(FS_MED1)));

MSE_S10_AVG= round(immse(imvol, uint16(FS_AVG1)));

MSE_S10_WNR= round(immse(imvol, uint16(FS_WNR1)));

MSE_S10_MMW= round(immse(imvol, uint16(FS_MMW1)));

%20%

MSE_G20_MED= round(immse(imvol, uint16(FG_MED2)));

MSE_G20_AVG= round(immse(imvol, uint16(FG_AVG2)));

MSE_G20_WNR= round(immse(imvol, uint16(FG_WNR2)));

MSE_G20_MMW= round(immse(imvol, uint16(FG_MMW2)));

MSE_P20_MED= round(immse(imvol, uint16(FP_MED2)));

MSE_P20_AVG= round(immse(imvol, uint16(FP_AVG2)));

MSE_P20_WNR= round(immse(imvol, uint16(FP_WNR2)));

MSE_P20_MMW= round(immse(imvol, uint16(FP_MMW2)));

MSE_S20_MED= round(immse(imvol, uint16(FS_MED2)));

MSE_S20_AVG= round(immse(imvol, uint16(FS_AVG2)));

MSE_S20_WNR= round(immse(imvol, uint16(FS_WNR2)));

MSE_S20_MMW= round(immse(imvol, uint16(FS_MMW2)));

%30%

MSE_G30_MED= round(immse(imvol, uint16(FG_MED3)));

MSE_G30_AVG= round(immse(imvol, uint16(FG_AVG3)));

MSE_G30_WNR= round(immse(imvol, uint16(FG_WNR3)));

MSE_G30_MMW= round(immse(imvol, uint16(FG_MMW3)));

MSE_P30_MED= round(immse(imvol, uint16(FP_MED3)));

MSE_P30_AVG= round(immse(imvol, uint16(FP_AVG3)));

MSE_P30_WNR= round(immse(imvol, uint16(FP_WNR3)));

MSE_P30_MMW= round(immse(imvol, uint16(FP_MMW3)));

MSE_S30_MED= round(immse(imvol, uint16(FS_MED3)));

MSE_S30_AVG= round(immse(imvol, uint16(FS_AVG3)));

MSE_S30_WNR= round(immse(imvol, uint16(FS_WNR3)));

MSE_S30_MMW= round(immse(imvol, uint16(FS_MMW3)));

%40%

MSE_G40_MED= round(immse(imvol, uint16(FG_MED4)));

MSE_G40_AVG= round(immse(imvol, uint16(FG_AVG4)));

MSE_G40_WNR= round(immse(imvol, uint16(FG_WNR4)));

MSE_G40_MMW= round(immse(imvol, uint16(FG_MMW4)));

MSE_P40_MED= round(immse(imvol, uint16(FP_MED4)));

MSE_P40_AVG= round(immse(imvol, uint16(FP_AVG4)));

MSE_P40_WNR= round(immse(imvol, uint16(FP_WNR4)));

MSE_P40_MMW= round(immse(imvol, uint16(FP_MMW4)));

MSE_S40_MED= round(immse(imvol, uint16(FS_MED4)));

MSE_S40_AVG= round(immse(imvol, uint16(FS_AVG4)));

MSE_S40_WNR= round(immse(imvol, uint16(FS_WNR4)));

MSE_S40_MMW= round(immse(imvol, uint16(FS_MMW4)));

%50%

MSE_G50_MED= round(immse(imvol, uint16(FG_MED5)));

MSE_G50_AVG= round(immse(imvol, uint16(FG_AVG5)));

MSE_G50_WNR= round(immse(imvol, uint16(FG_WNR5)));

MSE_G50_MMW= round(immse(imvol, uint16(FG_MMW5)));

MSE_P50_MED= round(immse(imvol, uint16(FP_MED5)));

MSE_P50_AVG= round(immse(imvol, uint16(FP_AVG5)));

MSE_P50_WNR= round(immse(imvol, uint16(FP_WNR5)));

MSE_P50_MMW= round(immse(imvol, uint16(FP_MMW5)));

MSE_S50_MED= round(immse(imvol, uint16(FS_MED5)));

MSE_S50_AVG= round(immse(imvol, uint16(FS_AVG5)));

MSE_S50_WNR= round(immse(imvol, uint16(FS_WNR5)));

MSE_S50_MMW= round(immse(imvol, uint16(FS_MMW5)));

%60%

MSE_G60_MED= round(immse(imvol, uint16(FG_MED6)));

MSE_G60_AVG= round(immse(imvol, uint16(FG_AVG6)));

MSE_G60_WNR= round(immse(imvol, uint16(FG_WNR6)));

MSE_G60_MMW= round(immse(imvol, uint16(FG_MMW6)));

MSE_P60_MED= round(immse(imvol, uint16(FP_MED6)));

MSE_P60_AVG= round(immse(imvol, uint16(FP_AVG6)));

MSE_P60_WNR= round(immse(imvol, uint16(FP_WNR6)));

MSE_P60_MMW= round(immse(imvol, uint16(FP_MMW6)));

MSE_S60_MED= round(immse(imvol, uint16(FS_MED6)));

MSE_S60_AVG= round(immse(imvol, uint16(FS_AVG6)));

MSE_S60_WNR= round(immse(imvol, uint16(FS_WNR6)));

MSE_S60_MMW= round(immse(imvol, uint16(FS_MMW6)));

%% CNR

%10%

[CNR_G10_MED, CR_G10_MED]= (cnr(imvol,bw1, (FG_MED1),NG1));

[CNR_G10_AVG, CR_G10_AVG]= (cnr(imvol,bw1, (FG_AVG1),NG1));

[CNR_G10_WNR, CR_G10_WNR]= (cnr(imvol,bw1, (FG_WNR1),NG1));

[CNR_G10_MMW, CR_G10_MMW]= (cnr(imvol,bw1, (FG_MMW1),NG1));

[CNR_P10_MED, CR_P10_MED]= (cnr(imvol,bw1, (FP_MED1),NP1));

[CNR_P10_AVG, CR_P10_AVG]= (cnr(imvol,bw1, (FP_AVG1),NP1));

[CNR_P10_WNR, CR_P10_WNR]= (cnr(imvol,bw1, (FP_WNR1),NP1));

[CNR_P10_MMW, CR_P10_MMW]= (cnr(imvol,bw1, (FP_MMW1),NP1));

[CNR_S10_MED, CR_S10_MED]= (cnr(imvol,bw1, (FS_MED1),NS1));

[CNR_S10_AVG, CR_S10_AVG]= (cnr(imvol,bw1, (FS_AVG1),NS1));

[CNR_S10_WNR, CR_S10_WNR]= (cnr(imvol,bw1, (FS_WNR1),NS1));

[CNR_S10_MMW, CR_S10_MMW]= (cnr(imvol,bw1, (FS_MMW1),NS1));

%20%

[CNR_G20_MED, CR_G20_MED]= (cnr(imvol,bw1, (FG_MED2),NG2));

[CNR_G20_AVG, CR_G20_AVG]= (cnr(imvol,bw1, (FG_AVG2),NG2));

[CNR_G20_WNR, CR_G20_WNR]= (cnr(imvol,bw1, (FG_WNR2),NG2));

[CNR_G20_MMW, CR_G20_MMW]= (cnr(imvol,bw1, (FG_MMW2),NG2));

[CNR_P20_MED, CR_P20_MED]= (cnr(imvol,bw1, (FP_MED2),NP2));

[CNR_P20_AVG, CR_P20_AVG]= (cnr(imvol,bw1, (FP_AVG2),NP2));

[CNR_P20_WNR, CR_P20_WNR]= (cnr(imvol,bw1, (FP_WNR2),NP2));

[CNR_P20_MMW, CR_P20_MMW]= (cnr(imvol,bw1, (FP_MMW2),NP2));

[CNR_S20_MED, CR_S20_MED]= (cnr(imvol,bw1, (FS_MED2),NS2));

[CNR_S20_AVG, CR_S20_AVG]= (cnr(imvol,bw1, (FS_AVG2),NS2));

[CNR_S20_WNR, CR_S20_WNR]= (cnr(imvol,bw1, (FS_WNR2),NS2));

[CNR_S20_MMW, CR_S20_MMW]= (cnr(imvol,bw1, (FS_MMW2),NS2));

%30%

[CNR_G30_MED, CR_G30_MED]= (cnr(imvol,bw1, (FG_MED3),NG3));

[CNR_G30_AVG, CR_G30_AVG]= (cnr(imvol,bw1, (FG_AVG3),NG3));

[CNR_G30_WNR, CR_G30_WNR]= (cnr(imvol,bw1, (FG_WNR3),NG3));

[CNR_G30_MMW, CR_G30_MMW]= (cnr(imvol,bw1, (FG_MMW3),NG3));

[CNR_P30_MED, CR_P30_MED]= (cnr(imvol,bw1, (FP_MED3),NP3));

[CNR_P30_AVG, CR_P30_AVG]= (cnr(imvol,bw1, (FP_AVG3),NP3));

[CNR_P30_WNR, CR_P30_WNR]= (cnr(imvol,bw1, (FP_WNR3),NP3));

[CNR_P30_MMW, CR_P30_MMW]= (cnr(imvol,bw1, (FP_MMW3),NP3));

[CNR_S30_MED, CR_S30_MED]= (cnr(imvol,bw1, (FS_MED3),NS3));

[CNR_S30_AVG, CR_S30_AVG]= (cnr(imvol,bw1, (FS_AVG3),NS3));

[CNR_S30_WNR, CR_S30_WNR]= (cnr(imvol,bw1, (FS_WNR3),NS3));

[CNR_S30_MMW, CR_S30_MMW]= (cnr(imvol,bw1, (FS_MMW3),NS3));

%40%

[CNR_G40_MED, CR_G40_MED]= (cnr(imvol,bw1, (FG_MED4),NG4));

[CNR_G40_AVG, CR_G40_AVG]= (cnr(imvol,bw1, (FG_AVG4),NG4));

[CNR_G40_WNR, CR_G40_WNR]= (cnr(imvol,bw1, (FG_WNR4),NG4));

[CNR_G40_MMW, CR_G40_MMW]= (cnr(imvol,bw1, (FG_MMW4),NG4));

[CNR_P40_MED, CR_P40_MED]= (cnr(imvol,bw1, (FP_MED4),NP4));

[CNR_P40_AVG, CR_P40_AVG]= (cnr(imvol,bw1, (FP_AVG4),NP4));

[CNR_P40_WNR, CR_P40_WNR]= (cnr(imvol,bw1, (FP_WNR4),NP4));

[CNR_P40_MMW, CR_P40_MMW]= (cnr(imvol,bw1, (FP_MMW4),NP4));

[CNR_S40_MED, CR_S40_MED]= (cnr(imvol,bw1, (FS_MED4),NS4));

[CNR_S40_AVG, CR_S40_AVG]= (cnr(imvol,bw1, (FS_AVG4),NS4));

[CNR_S40_WNR, CR_S40_WNR]= (cnr(imvol,bw1, (FS_WNR4),NS4));

[CNR_S40_MMW, CR_S40_MMW]= (cnr(imvol,bw1, (FS_MMW4),NS4));

%50%

[CNR_G50_MED, CR_G50_MED]= (cnr(imvol,bw1, (FG_MED5),NG5));

[CNR_G50_AVG, CR_G50_AVG]= (cnr(imvol,bw1, (FG_AVG5),NG5));

[CNR_G50_WNR, CR_G50_WNR]= (cnr(imvol,bw1, (FG_WNR5),NG5));

[CNR_G50_MMW, CR_G50_MMW]= (cnr(imvol,bw1, (FG_MMW5),NG5));

[CNR_P50_MED, CR_P50_MED]= (cnr(imvol,bw1, (FP_MED5),NP5));

[CNR_P50_AVG, CR_P50_AVG]= (cnr(imvol,bw1, (FP_AVG5),NP5));

[CNR_P50_WNR, CR_P50_WNR]= (cnr(imvol,bw1, (FP_WNR5),NP5));

[CNR_P50_MMW, CR_P50_MMW]= (cnr(imvol,bw1, (FP_MMW5),NP5));

[CNR_S50_MED, CR_S50_MED]= (cnr(imvol,bw1, (FS_MED5),NS5));

[CNR_S50_AVG, CR_S50_AVG]= (cnr(imvol,bw1, (FS_AVG5),NS5));

[CNR_S50_WNR, CR_S50_WNR]= (cnr(imvol,bw1, (FS_WNR5),NS5));

[CNR_S50_MMW, CR_S50_MMW]= (cnr(imvol,bw1, (FS_MMW5),NS5));

%60%

[CNR_G60_MED, CR_G60_MED]= (cnr(imvol,bw1, (FG_MED6),NG6));

[CNR_G60_AVG, CR_G60_AVG]= (cnr(imvol,bw1, (FG_AVG6),NG6));

[CNR_G60_WNR, CR_G60_WNR]= (cnr(imvol,bw1, (FG_WNR6),NG6));

[CNR_G60_MMW, CR_G60_MMW]= (cnr(imvol,bw1, (FG_MMW6),NG6));

[CNR_P60_MED, CR_P60_MED]= (cnr(imvol,bw1, (FP_MED6),NP6));

[CNR_P60_AVG, CR_P60_AVG]= (cnr(imvol,bw1, (FP_AVG6),NP6));

[CNR_P60_WNR, CR_P60_WNR]= (cnr(imvol,bw1, (FP_WNR6),NP6));

[CNR_P60_MMW, CR_P60_MMW]= (cnr(imvol,bw1, (FP_MMW6),NP6));

[CNR_S60_MED, CR_S60_MED]= (cnr(imvol,bw1, (FS_MED6),NS6));

[CNR_S60_AVG, CR_S60_AVG]= (cnr(imvol,bw1, (FS_AVG6),NS6));

[CNR_S60_WNR, CR_S60_WNR]= (cnr(imvol,bw1, (FS_WNR6),NS6));

[CNR_S60_MMW, CR_S60_MMW]= (cnr(imvol,bw1, (FS_MMW6),NS6));

%% Degree of smoothness DOS

%10%

DOS_G10_MED= round(dos(FG_MED1));

DOS_G10_AVG= round(dos(FG_AVG1));

DOS_G10_WNR= round(dos(FG_WNR1));

DOS_G10_MMW= round(dos(FG_MMW1));

DOS_P10_MED= round(dos(FP_MED1));

DOS_P10_AVG= round(dos(FP_AVG1));

DOS_P10_WNR= round(dos(FP_WNR1));

DOS_P10_MMW= round(dos(FP_MMW1));

DOS_S10_MED= round(dos(FS_MED1));

DOS_S10_AVG= round(dos(FS_AVG1));

DOS_S10_WNR= round(dos(FS_WNR1));

DOS_S10_MMW= round(dos(FS_MMW1));

%20%

DOS_G20_MED= round(dos(FG_MED2));

DOS_G20_AVG= round(dos(FG_AVG2));

DOS_G20_WNR= round(dos(FG_WNR2));

DOS_G20_MMW= round(dos(FG_MMW2));

DOS_P20_MED= round(dos(FP_MED2));

DOS_P20_AVG= round(dos(FP_AVG2));

DOS_P20_WNR= round(dos(FP_WNR2));

DOS_P20_MMW= round(dos(FP_MMW2));

DOS_S20_MED= round(dos(FS_MED2));

DOS_S20_AVG= round(dos(FS_AVG2));

DOS_S20_WNR= round(dos(FS_WNR2));

DOS_S20_MMW= round(dos(FS_MMW2));

%10%

DOS_G30_MED= round(dos(FG_MED3));

DOS_G30_AVG= round(dos(FG_AVG3));

DOS_G30_WNR= round(dos(FG_WNR3));

DOS_G30_MMW= round(dos(FG_MMW3));

DOS_P30_MED= round(dos(FP_MED3));

DOS_P30_AVG= round(dos(FP_AVG3));

DOS_P30_WNR= round(dos(FP_WNR3));

DOS_P30_MMW= round(dos(FP_MMW3));

DOS_S30_MED= round(dos(FS_MED3));

DOS_S30_AVG= round(dos(FS_AVG3));

DOS_S30_WNR= round(dos(FS_WNR3));

DOS_S30_MMW= round(dos(FS_MMW3));

%10%

DOS_G40_MED= round(dos(FG_MED4));

DOS_G40_AVG= round(dos(FG_AVG4));

DOS_G40_WNR= round(dos(FG_WNR4));

DOS_G40_MMW= round(dos(FG_MMW4));

DOS_P40_MED= round(dos(FP_MED4));

DOS_P40_AVG= round(dos(FP_AVG4));

DOS_P40_WNR= round(dos(FP_WNR4));

DOS_P40_MMW= round(dos(FP_MMW4));

DOS_S40_MED= round(dos(FS_MED4));

DOS_S40_AVG= round(dos(FS_AVG4));

DOS_S40_WNR= round(dos(FS_WNR4));

DOS_S40_MMW= round(dos(FS_MMW4));

%10

DOS_G50_MED= round(dos(FG_MED5));

DOS_G50_AVG= round(dos(FG_AVG5));

DOS_G50_WNR= round(dos(FG_WNR5));

DOS_G50_MMW= round(dos(FG_MMW5));

DOS_P50_MED= round(dos(FP_MED5));

DOS_P50_AVG= round(dos(FP_AVG5));

DOS_P50_WNR= round(dos(FP_WNR5));

DOS_P50_MMW= round(dos(FP_MMW5));

DOS_S50_MED= round(dos(FS_MED5));

DOS_S50_AVG= round(dos(FS_AVG5));

DOS_S50_WNR= round(dos(FS_WNR5));

DOS_S50_MMW= round(dos(FS_MMW5));

%10

DOS_G60_MED= round(dos(FG_MED6));

DOS_G60_AVG= round(dos(FG_AVG6));

DOS_G60_WNR= round(dos(FG_WNR6));

DOS_G60_MMW= round(dos(FG_MMW6));

DOS_P60_MED= round(dos(FP_MED6));

DOS_P60_AVG= round(dos(FP_AVG6));

DOS_P60_WNR= round(dos(FP_WNR6));

DOS_P60_MMW= round(dos(FP_MMW6));

DOS_S60_MED= round(dos(FS_MED6));

DOS_S60_AVG= round(dos(FS_AVG6));

DOS_S60_WNR= round(dos(FS_WNR6));

DOS_S60_MMW= round(dos(FS_MMW6));

%%%

%% show 10% results

% show 10% noise and filtering results

hf=figure

subplot(3,6,1);imshow(imvol,[]);title('Original','Fontsize',12);colormap jet;

msg1=strcat('Gaussian noise PSNR=',num2str(round(psnr(imvol,NG1))));

subplot(3,6,2);imshow(NG1,[]);title(msg1,'Fontsize',12);colormap jet;

ha=subplot(3,6,3);imshow(FG_MED1,[]);title('Median filter','Fontsize',12);colormap jet;

pos = get(ha, 'position');pos(2)=pos(2)-0.08;pos(1)=pos(1)+0.01;

txt=strcat('PSNR= ',num2str(PSNR_G10_MED),', CNR= ',num2str(CNR_G10_MED));

annotation(hf, 'textbox', pos, 'String', num2str(txt), 'vert', 'bottom', 'EdgeColor','none','FontSize',12,'FitBoxToText','off');

ha=subplot(3,6,4);imshow(FG_AVG1,[]);title('Mean filter','Fontsize',12);colormap jet;

pos = get(ha, 'position');pos(2)=pos(2)-0.08;pos(1)=pos(1)+0.01;

txt=strcat('PSNR= ',num2str(PSNR_G10_AVG),', CNR= ',num2str(CNR_G10_AVG));

annotation(hf, 'textbox', pos, 'String', num2str(txt), 'vert', 'bottom', 'EdgeColor','none','FontSize',12,'FitBoxToText','off');

ha=subplot(3,6,5);imshow(FG_WNR1,[]);title('Weiner filter','Fontsize',12);colormap jet;

pos = get(ha, 'position');pos(2)=pos(2)-0.08;pos(1)=pos(1)+0.01;

txt=strcat('PSNR= ',num2str(PSNR_G10_WNR),', CNR= ',num2str(CNR_G10_WNR));

annotation(hf, 'textbox', pos, 'String', num2str(txt), 'vert', 'bottom', 'EdgeColor','none','FontSize',12,'FitBoxToText','off');

ha=subplot(3,6,6);imshow(FG_MMW1,[]);title('MMWF filer','Fontsize',12);colormap jet;

pos = get(ha, 'position');pos(2)=pos(2)-0.08;pos(1)=pos(1)+0.01;

txt=strcat('PSNR= ',num2str(PSNR_G10_MMW),', CNR= ',num2str(CNR_G10_MMW));

annotation(hf, 'textbox', pos, 'String', num2str(txt), 'vert', 'bottom', 'EdgeColor','none','FontSize',12,'FitBoxToText','off');

subplot(3,6,7);imshow(imvol,[]);title('Original','Fontsize',12);colormap jet;

msg1=strcat('Possion noise PSNR=',num2str(round(psnr(imvol,uint16(NP1)))));

subplot(3,6,8);imshow(NP1,[]);title(msg1,'Fontsize',12);colormap jet;

ha=subplot(3,6,9),imshow(FP_MED1,[]);colormap jet;

pos = get(ha, 'position');pos(2)=pos(2)-0.08;pos(1)=pos(1)+0.01;

txt=strcat('PSNR= ',num2str(PSNR_P10_MED),', CNR= ',num2str(CNR_P10_MED));

annotation(hf, 'textbox', pos, 'String', num2str(txt), 'vert', 'bottom', 'EdgeColor','none','FontSize',12,'FitBoxToText','off');

ha=subplot(3,6,10);imshow(FP_AVG1,[]);colormap jet;

pos = get(ha, 'position');pos(2)=pos(2)-0.08;pos(1)=pos(1)+0.01;

txt=strcat('PSNR= ',num2str(PSNR_P10_AVG),', CNR= ',num2str(CNR_P10_AVG));

annotation(hf, 'textbox', pos, 'String', num2str(txt), 'vert', 'bottom', 'EdgeColor','none','FontSize',12,'FitBoxToText','off');

ha=subplot(3,6,11);imshow(FP_WNR1,[]);colormap jet;

pos = get(ha, 'position');pos(2)=pos(2)-0.08;pos(1)=pos(1)+0.01;

txt=strcat('PSNR= ',num2str(PSNR_P10_WNR),', CNR= ',num2str(CNR_P10_WNR));

annotation(hf, 'textbox', pos, 'String', num2str(txt), 'vert', 'bottom', 'EdgeColor','none','FontSize',12,'FitBoxToText','off');

ha=subplot(3,6,12);imshow(FP_MMW1,[]);colormap jet;

pos = get(ha, 'position');pos(2)=pos(2)-0.08;pos(1)=pos(1)+0.01;

txt=strcat('PSNR= ',num2str(PSNR_P10_MMW),', CNR= ',num2str(CNR_P10_MMW));

annotation(hf, 'textbox', pos, 'String', num2str(txt), 'vert', 'bottom', 'EdgeColor','none','FontSize',12,'FitBoxToText','off');

subplot(3,6,13);imshow(imvol,[]);title('Original','Fontsize',12);colormap jet;

msg1=strcat('Speckle noise PSNR=',num2str(round(psnr(imvol,NS1))));

subplot(3,6,14);imshow(NS1,[]);title(msg1,'Fontsize',12);colormap jet;

ha=subplot(3,6,15);imshow(FS_MED1,[]);colormap jet;

pos = get(ha, 'position');pos(2)=pos(2)-0.08;pos(1)=pos(1)+0.01;

txt=strcat('PSNR= ',num2str(PSNR_S10_MED),', CNR= ',num2str(CNR_S10_MED));

annotation(hf, 'textbox', pos, 'String', num2str(txt), 'vert', 'bottom', 'EdgeColor','none','FontSize',12,'FitBoxToText','off');

ha=subplot(3,6,16);imshow(FS_AVG1,[]);colormap jet;

pos = get(ha, 'position');pos(2)=pos(2)-0.08;pos(1)=pos(1)+0.01;

txt=strcat('PSNR= ',num2str(PSNR_S10_AVG),', CNR= ',num2str(CNR_S10_AVG));

annotation(hf, 'textbox', pos, 'String', num2str(txt), 'vert', 'bottom', 'EdgeColor','none','FontSize',12,'FitBoxToText','off');

ha=subplot(3,6,17);imshow(FS_WNR1,[]);colormap jet;

pos = get(ha, 'position');pos(2)=pos(2)-0.08;pos(1)=pos(1)+0.01;

txt=strcat('PSNR= ',num2str(PSNR_S10_WNR),', CNR= ',num2str(CNR_S10_WNR));

annotation(hf, 'textbox', pos, 'String', num2str(txt), 'vert', 'bottom', 'EdgeColor','none','FontSize',12,'FitBoxToText','off');

ha=subplot(3,6,18);imshow(FS_MMW1,[]);colormap jet;

pos = get(ha, 'position');pos(2)=pos(2)-0.08;pos(1)=pos(1)+0.01;

txt=strcat('PSNR= ',num2str(PSNR_S10_MMW),', CNR= ',num2str(CNR_S10_MMW));

annotation(hf, 'textbox', pos, 'String', num2str(txt), 'vert', 'bottom', 'EdgeColor','none','FontSize',12,'FitBoxToText','off');

% show 30% noise and filtering results

hf=figure

subplot(3,6,1),imshow(imvol,[]);title('Original','Fontsize',12);colormap jet;

msg1=strcat('Gaussian noise PSNR=',num2str(round(psnr(imvol,NG3))));

subplot(3,6,2),imshow(NG3,[]);title(msg1,'Fontsize',12);colormap jet;

ha=subplot(3,6,3),imshow(FG_MED3,[]);title('Median filter','Fontsize',12);colormap jet;

pos = get(ha, 'position');pos(2)=pos(2)-0.08;pos(1)=pos(1)+0.01;

txt=strcat('PSNR= ',num2str(PSNR_G30_MED),', CNR= ',num2str(CNR_G30_MED));

annotation(hf, 'textbox', pos, 'String', num2str(txt), 'vert', 'bottom', 'EdgeColor','none','FontSize',12,'FitBoxToText','off');

ha=subplot(3,6,4),imshow(FG_AVG3,[]);title('Mean filter','Fontsize',12);colormap jet;

pos = get(ha, 'position');pos(2)=pos(2)-0.08;pos(1)=pos(1)+0.01;

txt=strcat('PSNR= ',num2str(PSNR_G30_AVG),', CNR= ',num2str(CNR_G30_AVG));

annotation(hf, 'textbox', pos, 'String', num2str(txt), 'vert', 'bottom', 'EdgeColor','none','FontSize',12,'FitBoxToText','off');

ha=subplot(3,6,5);imshow(FG_WNR3,[]);title('Weiner filter','Fontsize',12);colormap jet;

pos = get(ha, 'position');pos(2)=pos(2)-0.08;pos(1)=pos(1)+0.01;

txt=strcat('PSNR= ',num2str(PSNR_G30_WNR),', CNR= ',num2str(CNR_G30_WNR));

annotation(hf, 'textbox', pos, 'String', num2str(txt), 'vert', 'bottom', 'EdgeColor','none','FontSize',12,'FitBoxToText','off');

ha=subplot(3,6,6);imshow(FG_MMW3,[]);title('MMWF filer','Fontsize',12);colormap jet;

pos = get(ha, 'position');pos(2)=pos(2)-0.08;pos(1)=pos(1)+0.01;

txt=strcat('PSNR= ',num2str(PSNR_G30_MMW),', CNR= ',num2str(CNR_G30_MMW));

annotation(hf, 'textbox', pos, 'String', num2str(txt), 'vert', 'bottom', 'EdgeColor','none','FontSize',12,'FitBoxToText','off');

subplot(3,6,7);imshow(imvol,[]);title('Original','Fontsize',12);colormap jet;

msg1=strcat('Possion noise PSNR=',num2str(round(psnr(imvol,uint16(NP3)))));

subplot(3,6,8);imshow(NP3,[]);title(msg1,'Fontsize',12);colormap jet;

ha=subplot(3,6,9);imshow(FP_MED3,[]);colormap jet;

pos = get(ha, 'position');pos(2)=pos(2)-0.08;pos(1)=pos(1)+0.01;

txt=strcat('PSNR= ',num2str(PSNR_P30_MED),', CNR= ',num2str(CNR_P30_MED));

annotation(hf, 'textbox', pos, 'String', num2str(txt), 'vert', 'bottom', 'EdgeColor','none','FontSize',12,'FitBoxToText','off');

ha=subplot(3,6,10);imshow(FP_AVG3,[]);colormap jet;

pos = get(ha, 'position');pos(2)=pos(2)-0.08;pos(1)=pos(1)+0.01;

txt=strcat('PSNR= ',num2str(PSNR_P30_AVG),', CNR= ',num2str(CNR_P30_AVG));

annotation(hf, 'textbox', pos, 'String', num2str(txt), 'vert', 'bottom', 'EdgeColor','none','FontSize',12,'FitBoxToText','off');

ha=subplot(3,6,11);imshow(FP_WNR3,[]);colormap jet;

pos = get(ha, 'position');pos(2)=pos(2)-0.08;pos(1)=pos(1)+0.01;

txt=strcat('PSNR= ',num2str(PSNR_P30_WNR),', CNR= ',num2str(CNR_P30_WNR));

annotation(hf, 'textbox', pos, 'String', num2str(txt), 'vert', 'bottom', 'EdgeColor','none','FontSize',12,'FitBoxToText','off');

ha=subplot(3,6,12);imshow(FP_MMW3,[]);colormap jet;

pos = get(ha, 'position');pos(2)=pos(2)-0.08;pos(1)=pos(1)+0.01;

txt=strcat('PSNR= ',num2str(PSNR_P30_MMW),', CNR= ',num2str(CNR_P30_MMW));

annotation(hf, 'textbox', pos, 'String', num2str(txt), 'vert', 'bottom', 'EdgeColor','none','FontSize',12,'FitBoxToText','off');

subplot(3,6,13);imshow(imvol,[]);title('Original','Fontsize',12);colormap jet;

msg1=strcat('Speckle noise PSNR=',num2str(round(psnr(imvol,(NS3)))));

subplot(3,6,14);imshow(NS3,[]);title(msg1,'Fontsize',12);colormap jet;

ha=subplot(3,6,15);imshow(FS_MED3,[]);colormap jet;

pos = get(ha, 'position');pos(2)=pos(2)-0.08;pos(1)=pos(1)+0.01;

txt=strcat('PSNR= ',num2str(PSNR_S30_MED),', CNR= ',num2str(CNR_S30_MED));

annotation(hf, 'textbox', pos, 'String', num2str(txt), 'vert', 'bottom', 'EdgeColor','none','FontSize',12,'FitBoxToText','off');

ha=subplot(3,6,16);imshow(FS_AVG3,[]);colormap jet;

pos = get(ha, 'position');pos(2)=pos(2)-0.08;pos(1)=pos(1)+0.01;

txt=strcat('PSNR= ',num2str(PSNR_S30_AVG),', CNR= ',num2str(CNR_S30_AVG));

annotation(hf, 'textbox', pos, 'String', num2str(txt), 'vert', 'bottom', 'EdgeColor','none','FontSize',12,'FitBoxToText','off');

ha=subplot(3,6,17);imshow(FS_WNR3,[]);colormap jet;

pos = get(ha, 'position');pos(2)=pos(2)-0.08;pos(1)=pos(1)+0.01;

txt=strcat('PSNR= ',num2str(PSNR_S30_WNR),', CNR= ',num2str(CNR_S30_WNR));

annotation(hf, 'textbox', pos, 'String', num2str(txt), 'vert', 'bottom', 'EdgeColor','none','FontSize',12,'FitBoxToText','off');

ha=subplot(3,6,18);imshow(FS_MMW3,[]);colormap jet;

pos = get(ha, 'position');pos(2)=pos(2)-0.08;pos(1)=pos(1)+0.01;

txt=strcat('PSNR= ',num2str(PSNR_S30_MMW),', CNR= ',num2str(CNR_S30_MMW));

annotation(hf, 'textbox', pos, 'String', num2str(txt), 'vert', 'bottom', 'EdgeColor','none','FontSize',12,'FitBoxToText','off');

% show 60% noise and filtering results

hf=figure

subplot(3,6,1);imshow(imvol,[]);title('Original','Fontsize',12);colormap jet;

msg1=strcat('Gaussian noise PSNR=',num2str(round(psnr(imvol,NG6))));

subplot(3,6,2);imshow(NG6,[]);title(msg1,'Fontsize',12);colormap jet;

ha=subplot(3,6,3);imshow(FG_MED6,[]);title('Median filter','Fontsize',12);colormap jet;

pos = get(ha, 'position');pos(2)=pos(2)-0.08;pos(1)=pos(1)+0.01;

txt=strcat('PSNR= ',num2str(PSNR_G60_MED),', CNR= ',num2str(CNR_G60_MED));

annotation(hf, 'textbox', pos, 'String', num2str(txt), 'vert', 'bottom', 'EdgeColor','none','FontSize',12,'FitBoxToText','off');

ha=subplot(3,6,4);imshow(FG_AVG6,[]);title('Mean filter','Fontsize',12);colormap jet;

pos = get(ha, 'position');pos(2)=pos(2)-0.08;pos(1)=pos(1)+0.01;

txt=strcat('PSNR= ',num2str(PSNR_G60_AVG),', CNR= ',num2str(CNR_G60_AVG));

annotation(hf, 'textbox', pos, 'String', num2str(txt), 'vert', 'bottom', 'EdgeColor','none','FontSize',12,'FitBoxToText','off');

ha=subplot(3,6,5);imshow(FG_WNR6,[]);title('Weiner filter','Fontsize',12);colormap jet;

pos = get(ha, 'position');pos(2)=pos(2)-0.08;pos(1)=pos(1)+0.01;

txt=strcat('PSNR= ',num2str(PSNR_G60_WNR),', CNR= ',num2str(CNR_G60_WNR));

annotation(hf, 'textbox', pos, 'String', num2str(txt), 'vert', 'bottom', 'EdgeColor','none','FontSize',12,'FitBoxToText','off');

ha=subplot(3,6,6);imshow(FG_MMW6,[]);title('MMWF filer','Fontsize',12);colormap jet;

pos = get(ha, 'position');pos(2)=pos(2)-0.08;pos(1)=pos(1)+0.01;

txt=strcat('PSNR= ',num2str(PSNR_G60_MMW),', CNR= ',num2str(CNR_G60_MMW));

annotation(hf, 'textbox', pos, 'String', num2str(txt), 'vert', 'bottom', 'EdgeColor','none','FontSize',12,'FitBoxToText','off');

subplot(3,6,7);imshow(imvol,[]);title('Original','Fontsize',12);colormap jet;

msg1=strcat('Possion noise PSNR=',num2str(round(psnr(imvol,uint16(NP6)))));

subplot(3,6,8);imshow(NP6,[]);title(msg1,'Fontsize',12);colormap jet;

ha=subplot(3,6,9);imshow(FP_MED6,[]);colormap jet;

pos = get(ha, 'position');pos(2)=pos(2)-0.08;pos(1)=pos(1)+0.01;

txt=strcat('PSNR= ',num2str(PSNR_P60_MED),', CNR= ',num2str(CNR_P60_MED));

annotation(hf, 'textbox', pos, 'String', num2str(txt), 'vert', 'bottom', 'EdgeColor','none','FontSize',12,'FitBoxToText','off');

ha=subplot(3,6,10);imshow(FP_AVG6,[]);colormap jet;

pos = get(ha, 'position');pos(2)=pos(2)-0.08;pos(1)=pos(1)+0.01;

txt=strcat('PSNR= ',num2str(PSNR_P60_AVG),', CNR= ',num2str(CNR_P60_AVG));

annotation(hf, 'textbox', pos, 'String', num2str(txt), 'vert', 'bottom', 'EdgeColor','none','FontSize',12,'FitBoxToText','off');

ha=subplot(3,6,11);imshow(FP_WNR6,[]);colormap jet;

pos = get(ha, 'position');pos(2)=pos(2)-0.08;pos(1)=pos(1)+0.01;

txt=strcat('PSNR= ',num2str(PSNR_P60_WNR),', CNR= ',num2str(CNR_P60_WNR));

annotation(hf, 'textbox', pos, 'String', num2str(txt), 'vert', 'bottom', 'EdgeColor','none','FontSize',12,'FitBoxToText','off');

ha=subplot(3,6,12);imshow(FP_MMW6,[]);colormap jet;

pos = get(ha, 'position');pos(2)=pos(2)-0.08;pos(1)=pos(1)+0.01;

txt=strcat('PSNR= ',num2str(PSNR_P60_MMW),', CNR= ',num2str(CNR_P60_MMW));

annotation(hf, 'textbox', pos, 'String', num2str(txt), 'vert', 'bottom', 'EdgeColor','none','FontSize',12,'FitBoxToText','off');

subplot(3,6,13);imshow(imvol,[]);title('Original','Fontsize',12);colormap jet;

msg1=strcat('Speckle noise PSNR=',num2str(round(psnr(imvol,(NS6)))));

subplot(3,6,14);imshow(NS6,[]);title(msg1,'Fontsize',12);colormap jet;

ha=subplot(3,6,15);imshow(FS_MED6,[]);colormap jet;

pos = get(ha, 'position');pos(2)=pos(2)-0.08;pos(1)=pos(1)+0.01;

txt=strcat('PSNR= ',num2str(PSNR_S60_MED),', CNR= ',num2str(CNR_S60_MED));

annotation(hf, 'textbox', pos, 'String', num2str(txt), 'vert', 'bottom', 'EdgeColor','none','FontSize',12,'FitBoxToText','off');

ha=subplot(3,6,16);imshow(FS_AVG6,[]);colormap jet;

pos = get(ha, 'position');pos(2)=pos(2)-0.08;pos(1)=pos(1)+0.01;

txt=strcat('PSNR= ',num2str(PSNR_S60_AVG),', CNR= ',num2str(CNR_S60_AVG));

annotation(hf, 'textbox', pos, 'String', num2str(txt), 'vert', 'bottom', 'EdgeColor','none','FontSize',12,'FitBoxToText','off');

ha=subplot(3,6,17);imshow(FS_WNR6,[]);colormap jet;

pos = get(ha, 'position');pos(2)=pos(2)-0.08;pos(1)=pos(1)+0.01;

txt=strcat('PSNR= ',num2str(PSNR_S60_WNR),', CNR= ',num2str(CNR_S60_WNR));

annotation(hf, 'textbox', pos, 'String', num2str(txt), 'vert', 'bottom', 'EdgeColor','none','FontSize',12,'FitBoxToText','off');

ha=subplot(3,6,18);imshow(FS_MMW6,[]);colormap jet;

pos = get(ha, 'position');pos(2)=pos(2)-0.08;pos(1)=pos(1)+0.01;

txt=strcat('PSNR= ',num2str(PSNR_S60_MMW),', CNR= ',num2str(CNR_S60_MMW));

annotation(hf, 'textbox', pos, 'String', num2str(txt), 'vert', 'bottom', 'EdgeColor','none','FontSize',12,'FitBoxToText','off');

%% Export to excel sheet

% PSNR

filename = 'Filtering.xlsx';

T1 = table({PSNR_G10_MED},{PSNR_G20_MED},{PSNR_G30_MED},{PSNR_G40_MED},{PSNR_G50_MED},{PSNR_G60_MED});

writetable(T1,filename,'Sheet','Sheet1','WriteVariableNames',false,'Range',strcat('B3:G3'));

T1 = table({PSNR_G10_AVG},{PSNR_G20_AVG},{PSNR_G30_AVG},{PSNR_G40_AVG},{PSNR_G50_AVG},{PSNR_G60_AVG});

writetable(T1,filename,'Sheet','Sheet1','WriteVariableNames',false,'Range',strcat('B4:G4'));

T1 = table({PSNR_G10_WNR},{PSNR_G20_WNR},{PSNR_G30_WNR},{PSNR_G40_WNR},{PSNR_G50_WNR},{PSNR_G60_WNR});

writetable(T1,filename,'Sheet','Sheet1','WriteVariableNames',false,'Range',strcat('B5:G5'));

T1 = table({PSNR_G10_MMW},{PSNR_G20_MMW},{PSNR_G30_MMW},{PSNR_G40_MMW},{PSNR_G50_MMW},{PSNR_G60_MMW});

writetable(T1,filename,'Sheet','Sheet1','WriteVariableNames',false,'Range',strcat('B6:G6'));

%

T1 = table({PSNR_P10_MED},{PSNR_P20_MED},{PSNR_P30_MED},{PSNR_P40_MED},{PSNR_P50_MED},{PSNR_P60_MED});

writetable(T1,filename,'Sheet','Sheet1','WriteVariableNames',false,'Range',strcat('B10:G10'));

T1 = table({PSNR_P10_AVG},{PSNR_P20_AVG},{PSNR_P30_AVG},{PSNR_P40_AVG},{PSNR_P50_AVG},{PSNR_P60_AVG});

writetable(T1,filename,'Sheet','Sheet1','WriteVariableNames',false,'Range',strcat('B11:G11'));

T1 = table({PSNR_P10_WNR},{PSNR_P20_WNR},{PSNR_P30_WNR},{PSNR_P40_WNR},{PSNR_P50_WNR},{PSNR_P60_WNR});

writetable(T1,filename,'Sheet','Sheet1','WriteVariableNames',false,'Range',strcat('B12:G12'));

T1 = table({PSNR_P10_MMW},{PSNR_P20_MMW},{PSNR_P30_MMW},{PSNR_P40_MMW},{PSNR_P50_MMW},{PSNR_P60_MMW});

writetable(T1,filename,'Sheet','Sheet1','WriteVariableNames',false,'Range',strcat('B13:G13'));

%

T1 = table({PSNR_S10_MED},{PSNR_S20_MED},{PSNR_S30_MED},{PSNR_S40_MED},{PSNR_S50_MED},{PSNR_S60_MED});

writetable(T1,filename,'Sheet','Sheet1','WriteVariableNames',false,'Range',strcat('B17:G17'));

T1 = table({PSNR_S10_AVG},{PSNR_S20_AVG},{PSNR_S30_AVG},{PSNR_S40_AVG},{PSNR_S50_AVG},{PSNR_S60_AVG});

writetable(T1,filename,'Sheet','Sheet1','WriteVariableNames',false,'Range',strcat('B18:G18'));

T1 = table({PSNR_S10_WNR},{PSNR_S20_WNR},{PSNR_S30_WNR},{PSNR_S40_WNR},{PSNR_S50_WNR},{PSNR_S60_WNR});

writetable(T1,filename,'Sheet','Sheet1','WriteVariableNames',false,'Range',strcat('B19:G19'));

T1 = table({PSNR_S10_MMW},{PSNR_S20_MMW},{PSNR_S30_MMW},{PSNR_S40_MMW},{PSNR_S50_MMW},{PSNR_S60_MMW});

writetable(T1,filename,'Sheet','Sheet1','WriteVariableNames',false,'Range',strcat('B20:G20'));

% MSE

filename = 'Filtering.xlsx';

T1 = table({MSE_G10_MED},{MSE_G20_MED},{MSE_G30_MED},{MSE_G40_MED},{MSE_G50_MED},{MSE_G60_MED});

writetable(T1,filename,'Sheet','Sheet1','WriteVariableNames',false,'Range',strcat('K3:P3'));

T1 = table({MSE_G10_AVG},{MSE_G20_AVG},{MSE_G30_AVG},{MSE_G40_AVG},{MSE_G50_AVG},{MSE_G60_AVG});

writetable(T1,filename,'Sheet','Sheet1','WriteVariableNames',false,'Range',strcat('K4:P4'));

T1 = table({MSE_G10_WNR},{MSE_G20_WNR},{MSE_G30_WNR},{MSE_G40_WNR},{MSE_G50_WNR},{MSE_G60_WNR});

writetable(T1,filename,'Sheet','Sheet1','WriteVariableNames',false,'Range',strcat('K5:P5'));

T1 = table({MSE_G10_MMW},{MSE_G20_MMW},{MSE_G30_MMW},{MSE_G40_MMW},{MSE_G50_MMW},{MSE_G60_MMW});

writetable(T1,filename,'Sheet','Sheet1','WriteVariableNames',false,'Range',strcat('K6:P6'));

%

T1 = table({MSE_P10_MED},{MSE_P20_MED},{MSE_P30_MED},{MSE_P40_MED},{MSE_P50_MED},{MSE_P60_MED});

writetable(T1,filename,'Sheet','Sheet1','WriteVariableNames',false,'Range',strcat('K10:P10'));

T1 = table({MSE_P10_AVG},{MSE_P20_AVG},{MSE_P30_AVG},{MSE_P40_AVG},{MSE_P50_AVG},{MSE_P60_AVG});

writetable(T1,filename,'Sheet','Sheet1','WriteVariableNames',false,'Range',strcat('K11:P11'));

T1 = table({MSE_P10_WNR},{MSE_P20_WNR},{MSE_P30_WNR},{MSE_P40_WNR},{MSE_P50_WNR},{MSE_P60_WNR});

writetable(T1,filename,'Sheet','Sheet1','WriteVariableNames',false,'Range',strcat('K12:P12'));

T1 = table({MSE_P10_MMW},{MSE_P20_MMW},{MSE_P30_MMW},{MSE_P40_MMW},{MSE_P50_MMW},{MSE_P60_MMW});

writetable(T1,filename,'Sheet','Sheet1','WriteVariableNames',false,'Range',strcat('K13:P13'));

%

T1 = table({MSE_S10_MED},{MSE_S20_MED},{MSE_S30_MED},{MSE_S40_MED},{MSE_S50_MED},{MSE_S60_MED});

writetable(T1,filename,'Sheet','Sheet1','WriteVariableNames',false,'Range',strcat('K17:P17'));

T1 = table({MSE_S10_AVG},{MSE_S20_AVG},{MSE_S30_AVG},{MSE_S40_AVG},{MSE_S50_AVG},{MSE_S60_AVG});

writetable(T1,filename,'Sheet','Sheet1','WriteVariableNames',false,'Range',strcat('K18:P18'));

T1 = table({MSE_S10_WNR},{MSE_S20_WNR},{MSE_S30_WNR},{MSE_S40_WNR},{MSE_S50_WNR},{MSE_S60_WNR});

writetable(T1,filename,'Sheet','Sheet1','WriteVariableNames',false,'Range',strcat('K19:P19'));

T1 = table({MSE_S10_MMW},{MSE_S20_MMW},{MSE_S30_MMW},{MSE_S40_MMW},{MSE_S50_MMW},{MSE_S60_MMW});

writetable(T1,filename,'Sheet','Sheet1','WriteVariableNames',false,'Range',strcat('K20:P20'));

% CNR

filename = 'Filtering.xlsx';

T1 = table({CNR_G10_MED},{CNR_G20_MED},{CNR_G30_MED},{CNR_G40_MED},{CNR_G50_MED},{CNR_G60_MED});

writetable(T1,filename,'Sheet','Sheet1','WriteVariableNames',false,'Range',strcat('T3:Y3'));

T1 = table({CNR_G10_AVG},{CNR_G20_AVG},{CNR_G30_AVG},{CNR_G40_AVG},{CNR_G50_AVG},{CNR_G60_AVG});

writetable(T1,filename,'Sheet','Sheet1','WriteVariableNames',false,'Range',strcat('T4:Y4'));

T1 = table({CNR_G10_WNR},{CNR_G20_WNR},{CNR_G30_WNR},{CNR_G40_WNR},{CNR_G50_WNR},{CNR_G60_WNR});

writetable(T1,filename,'Sheet','Sheet1','WriteVariableNames',false,'Range',strcat('T5:Y5'));

T1 = table({CNR_G10_MMW},{CNR_G20_MMW},{CNR_G30_MMW},{CNR_G40_MMW},{CNR_G50_MMW},{CNR_G60_MMW});

writetable(T1,filename,'Sheet','Sheet1','WriteVariableNames',false,'Range',strcat('T6:Y6'));

%

T1 = table({CNR_P10_MED},{CNR_P20_MED},{CNR_P30_MED},{CNR_P40_MED},{CNR_P50_MED},{CNR_P60_MED});

writetable(T1,filename,'Sheet','Sheet1','WriteVariableNames',false,'Range',strcat('T10:Y10'));

T1 = table({CNR_P10_AVG},{CNR_P20_AVG},{CNR_P30_AVG},{CNR_P40_AVG},{CNR_P50_AVG},{CNR_P60_AVG});

writetable(T1,filename,'Sheet','Sheet1','WriteVariableNames',false,'Range',strcat('T11:Y11'));

T1 = table({CNR_P10_WNR},{CNR_P20_WNR},{CNR_P30_WNR},{CNR_P40_WNR},{CNR_P50_WNR},{CNR_P60_WNR});

writetable(T1,filename,'Sheet','Sheet1','WriteVariableNames',false,'Range',strcat('T12:Y12'));

T1 = table({CNR_P10_MMW},{CNR_P20_MMW},{CNR_P30_MMW},{CNR_P40_MMW},{CNR_P50_MMW},{CNR_P60_MMW});

writetable(T1,filename,'Sheet','Sheet1','WriteVariableNames',false,'Range',strcat('T13:Y13'));

%

T1 = table({CNR_S10_MED},{CNR_S20_MED},{CNR_S30_MED},{CNR_S40_MED},{CNR_S50_MED},{CNR_S60_MED});

writetable(T1,filename,'Sheet','Sheet1','WriteVariableNames',false,'Range',strcat('T17:Y17'));

T1 = table({CNR_S10_AVG},{CNR_S20_AVG},{CNR_S30_AVG},{CNR_S40_AVG},{CNR_S50_AVG},{CNR_S60_AVG});

writetable(T1,filename,'Sheet','Sheet1','WriteVariableNames',false,'Range',strcat('T18:Y18'));

T1 = table({CNR_S10_WNR},{CNR_S20_WNR},{CNR_S30_WNR},{CNR_S40_WNR},{CNR_S50_WNR},{CNR_S60_WNR});

writetable(T1,filename,'Sheet','Sheet1','WriteVariableNames',false,'Range',strcat('T19:Y19'));

T1 = table({CNR_S10_MMW},{CNR_S20_MMW},{CNR_S30_MMW},{CNR_S40_MMW},{CNR_S50_MMW},{CNR_S60_MMW});

writetable(T1,filename,'Sheet','Sheet1','WriteVariableNames',false,'Range',strcat('T20:Y20'));

%

%% Contrast Resolution CR

% CR

filename = 'Filtering.xlsx';

T1 = table({CR_G10_MED},{CR_G20_MED},{CR_G30_MED},{CR_G40_MED},{CR_G50_MED},{CR_G60_MED});

writetable(T1,filename,'Sheet','Sheet1','WriteVariableNames',false,'Range',strcat('AC3:AH3'));

T1 = table({CR_G10_AVG},{CR_G20_AVG},{CR_G30_AVG},{CR_G40_AVG},{CR_G50_AVG},{CR_G60_AVG});

writetable(T1,filename,'Sheet','Sheet1','WriteVariableNames',false,'Range',strcat('AC4:AH4'));

T1 = table({CR_G10_WNR},{CR_G20_WNR},{CR_G30_WNR},{CR_G40_WNR},{CR_G50_WNR},{CR_G60_WNR});

writetable(T1,filename,'Sheet','Sheet1','WriteVariableNames',false,'Range',strcat('AC5:AH5'));

T1 = table({CR_G10_MMW},{CR_G20_MMW},{CR_G30_MMW},{CR_G40_MMW},{CR_G50_MMW},{CR_G60_MMW});

writetable(T1,filename,'Sheet','Sheet1','WriteVariableNames',false,'Range',strcat('AC6:AH6'));

%

T1 = table({CR_P10_MED},{CR_P20_MED},{CR_P30_MED},{CR_P40_MED},{CR_P50_MED},{CR_P60_MED});

writetable(T1,filename,'Sheet','Sheet1','WriteVariableNames',false,'Range',strcat('AC10:AH10'));

T1 = table({CR_P10_AVG},{CR_P20_AVG},{CR_P30_AVG},{CR_P40_AVG},{CR_P50_AVG},{CR_P60_AVG});

writetable(T1,filename,'Sheet','Sheet1','WriteVariableNames',false,'Range',strcat('AC11:AH11'));

T1 = table({CR_P10_WNR},{CR_P20_WNR},{CR_P30_WNR},{CR_P40_WNR},{CR_P50_WNR},{CR_P60_WNR});

writetable(T1,filename,'Sheet','Sheet1','WriteVariableNames',false,'Range',strcat('AC12:AH12'));

T1 = table({CR_P10_MMW},{CR_P20_MMW},{CR_P30_MMW},{CR_P40_MMW},{CR_P50_MMW},{CR_P60_MMW});

writetable(T1,filename,'Sheet','Sheet1','WriteVariableNames',false,'Range',strcat('AC13:AH13'));

%

T1 = table({CR_S10_MED},{CR_S20_MED},{CR_S30_MED},{CR_S40_MED},{CR_S50_MED},{CR_S60_MED});

writetable(T1,filename,'Sheet','Sheet1','WriteVariableNames',false,'Range',strcat('AC17:AH17'));

T1 = table({CR_S10_AVG},{CR_S20_AVG},{CR_S30_AVG},{CR_S40_AVG},{CR_S50_AVG},{CR_S60_AVG});

writetable(T1,filename,'Sheet','Sheet1','WriteVariableNames',false,'Range',strcat('AC18:AH18'));

T1 = table({CR_S10_WNR},{CR_S20_WNR},{CR_S30_WNR},{CR_S40_WNR},{CR_S50_WNR},{CR_S60_WNR});

writetable(T1,filename,'Sheet','Sheet1','WriteVariableNames',false,'Range',strcat('AC19:AH19'));

T1 = table({CR_S10_MMW},{CR_S20_MMW},{CR_S30_MMW},{CR_S40_MMW},{CR_S50_MMW},{CR_S60_MMW});

writetable(T1,filename,'Sheet','Sheet1','WriteVariableNames',false,'Range',strcat('AC20:AH20'));

%

%% Degree of smoothness DOS

% DOS

filename = 'Filtering.xlsx';

T1 = table({DOS_G10_MED},{DOS_G20_MED},{DOS_G30_MED},{DOS_G40_MED},{DOS_G50_MED},{DOS_G60_MED});

writetable(T1,filename,'Sheet','Sheet1','WriteVariableNames',false,'Range',strcat('AL3:AQ3'));

T1 = table({DOS_G10_AVG},{DOS_G20_AVG},{DOS_G30_AVG},{DOS_G40_AVG},{DOS_G50_AVG},{DOS_G60_AVG});

writetable(T1,filename,'Sheet','Sheet1','WriteVariableNames',false,'Range',strcat('AL4:AQ4'));

T1 = table({DOS_G10_WNR},{DOS_G20_WNR},{DOS_G30_WNR},{DOS_G40_WNR},{DOS_G50_WNR},{DOS_G60_WNR});

writetable(T1,filename,'Sheet','Sheet1','WriteVariableNames',false,'Range',strcat('AL5:AQ5'));

T1 = table({DOS_G10_MMW},{DOS_G20_MMW},{DOS_G30_MMW},{DOS_G40_MMW},{DOS_G50_MMW},{DOS_G60_MMW});

writetable(T1,filename,'Sheet','Sheet1','WriteVariableNames',false,'Range',strcat('AL6:AQ6'));

T1 = table({DOS_P10_MED},{DOS_P20_MED},{DOS_P30_MED},{DOS_P40_MED},{DOS_P50_MED},{DOS_P60_MED});

writetable(T1,filename,'Sheet','Sheet1','WriteVariableNames',false,'Range',strcat('AL10:AQ10'));

T1 = table({DOS_P10_AVG},{DOS_P20_AVG},{DOS_P30_AVG},{DOS_P40_AVG},{DOS_P50_AVG},{DOS_P60_AVG});

writetable(T1,filename,'Sheet','Sheet1','WriteVariableNames',false,'Range',strcat('AL11:AQ11'));

T1 = table({DOS_P10_WNR},{DOS_P20_WNR},{DOS_P30_WNR},{DOS_P40_WNR},{DOS_P50_WNR},{DOS_P60_WNR});

writetable(T1,filename,'Sheet','Sheet1','WriteVariableNames',false,'Range',strcat('AL12:AQ12'));

T1 = table({DOS_P10_MMW},{DOS_P20_MMW},{DOS_P30_MMW},{DOS_P40_MMW},{DOS_P50_MMW},{DOS_P60_MMW});

writetable(T1,filename,'Sheet','Sheet1','WriteVariableNames',false,'Range',strcat('AL13:AQ13'));

T1 = table({DOS_S10_MED},{DOS_S20_MED},{DOS_S30_MED},{DOS_S40_MED},{DOS_S50_MED},{DOS_S60_MED});

writetable(T1,filename,'Sheet','Sheet1','WriteVariableNames',false,'Range',strcat('AL17:AQ17'));

T1 = table({DOS_S10_AVG},{DOS_S20_AVG},{DOS_S30_AVG},{DOS_S40_AVG},{DOS_S50_AVG},{DOS_S60_AVG});

writetable(T1,filename,'Sheet','Sheet1','WriteVariableNames',false,'Range',strcat('AL18:AQ18'));

T1 = table({DOS_S10_WNR},{DOS_S20_WNR},{DOS_S30_WNR},{DOS_S40_WNR},{DOS_S50_WNR},{DOS_S60_WNR});

writetable(T1,filename,'Sheet','Sheet1','WriteVariableNames',false,'Range',strcat('AL19:AQ19'));

T1 = table({DOS_S10_MMW},{DOS_S20_MMW},{DOS_S30_MMW},{DOS_S40_MMW},{DOS_S50_MMW},{DOS_S60_MMW});

writetable(T1,filename,'Sheet','Sheet1','WriteVariableNames',false,'Range',strcat('AL20:AQ20'));

% noise levels

T1 = table(round(psnr(imvol,NG1)),round(psnr(imvol,NG2)),round(psnr(imvol,NG3)),round(psnr(imvol,NG4)),round(psnr(imvol,NG5)),round(psnr(imvol,NG6)));

writetable(T1,filename,'Sheet','Sheet1','WriteVariableNames',false,'Range',strcat('B26:G26'));

T1 = table(round(psnr(imvol,uint16(NP1))),round(psnr(imvol,uint16(NP2))),round(psnr(imvol,uint16(NP3))),round(psnr(imvol,uint16(NP4))),round(psnr(imvol,uint16(NP5))),round(psnr(imvol,uint16(NP6))));

writetable(T1,filename,'Sheet','Sheet1','WriteVariableNames',false,'Range',strcat('B27:G27'));

T1 = table(round(psnr(imvol,uint16(NS1))),round(psnr(imvol,uint16(NS2))),round(psnr(imvol,uint16(NS3))),round(psnr(imvol,uint16(NS4))),round(psnr(imvol,uint16(NS5))),round(psnr(imvol,uint16(NS6))));

writetable(T1,filename,'Sheet','Sheet1','WriteVariableNames',false,'Range',strcat('B28:G28'));

disp('Data has been written to excel sheet, check Filtering.xlsx in the current folder!');

%%%%%%%%%%%%%%%%%%%%%%%%%%%%%%%%%%%%%%%%%%%%%%%%%%%%%%%

| **M-file name** | Image_ Entropy_ Experiment_ modified_ Whole_Image |
| --- | --- |
| **Type** | Script |
| **Description** | As same as entropy experiment, but using the whole image instead of ROI |
| **comments** | Novel: Written: 31-12-2021 |

**Code:** The same code as previous files but the input will be the original whole muga image.

| **M-file name** | Image_ Entropy_ Experiment_ Possion_Gaussian_ ColumnScanning |
| --- | --- |
| **Type** | Script |
| **Description** | As same as entropy experiment, but using two new image scanning methods; the first is the column scanning and the second is the circular scanning of row scanning. |
| **comments** | Novel: Written: 31-12-2021 |

**Code:** The same as previous code but using different scanning methods:

%column scanning

[rows,cols] = size(imvol);

k=1;

s1Row=[];s2Row=[];s3Row=[];s4Row=[];s5Row=[];s6Row=[];s7Row=[];

for j = 1:cols

for i = 1:rows

if(bw1(j,i)==1)

s1Row(k)=double(I11(j,i));

s2Row(k)=(I12(j,i));

s3Row(k)=(I13(j,i));

s4Row(k)=(I14(j,i));

s5Row(k)=(I15(j,i));

s6Row(k)=(I16(j,i));

s7Row(k)=(I17(j,i));

k=k+1;

end

end

end

s1Row=s1Row';

s2Row=s2Row';

s3Row=s3Row';

s4Row=s4Row';

s5Row=s5Row';

s6Row=s6Row';

s7Row=s7Row';

%% Circular scanning

%%

rp=regionprops(bwlabel(bw1),'Centroid','MajorAxisLength');

MJA=round([rp.MajorAxisLength]);

Centroid=round([rp.Centroid])

[xgrid, ygrid] = meshgrid(1:size(bw1,2), 1:size(bw1,1));

MatX=[];MatY=[];

rad=round(MJA/2);

I11=imvol;

I12 = NP1;

I13 = NP2;

I14 = NP3;

I15 = NP4;

I16 = NP5;

I17 = NP6;

iter=1;

PC1=[];PC2=[];PC3=[];PC4=[];PC5=[];PC6=[];PC7=[];

while (1)

mask = edge(((xgrid-Centroid(1)).^2 + (ygrid-Centroid(2)).^2) <= (rad).^2,'canny');

PC1=[PC1;double(I11(mask))];

PC2=[PC2;I12(mask)];

PC3=[PC3;I13(mask)];

PC4=[PC4;I14(mask)];

PC5=[PC5;I15(mask)];

PC6=[PC6;I16(mask)];

PC7=[PC7;I17(mask)];

iter=iter+1;

rad=rad-1;

if(rad<0)

break;

end

end

%%%%%%%%%%%%%%%%%%%%%%%%%%%%%%%%%%%%%%%%%%%%%%%%%%%%%%%%%%%%%%%%%%%

| **S** | **M-file name** | **Type** | **Description** | **Novel or open-source** |
| --- | --- | --- | --- | --- |
|  |  |  |  |  |

| **M-file name** | Filtering_ Experiment _CNR _SR_MSI_PSNR |
| --- | --- |
| **Type** | Script |
| **Description** | This script apply an experiment that uses the MUGA image (16-frame), applies different noise types on it, then apply different filters (Median, Average or mean, Wiener and MMWF), then the CNR,SR,PSNR and MSE are computed and stored in the file called: Filtering.xlsx file. |
| **comments** | Novel: Last modified 22-12-2021 |

Code:

% read images (post-chemo).

x = inputdlg('Pick Post image:',...

'Define Image', [1]);

name= (x{:}); % image number

imvol = squeeze(dicomread(name));

info = dicominfo(name);

for i=1:1:info.NumberOfFrames

imvol(:,:,i)=medfilt2(imvol(:,:,i),[3 3]);%

end

figure,imshow(sum(imvol,3),[]); title('Select seed point');

colormap jet;colorbar

sp = ginput(1);

[a,B, C, D,min_img2,F,imvolmn2,bw1, AA, AB, AC, AD, AE, AF,AG, AH, AI, aj, sp]=max_min_MUGA3(imvol,0,sp,[],[]);

close

imvol=uint16(sum(imvol,3));

% adding gaussian noise to imvol

NG1=imnoise(imvol,'Gaussian',0,0.00001);

NG2=imnoise(imvol,'Gaussian',0,0.00002);

NG3=imnoise(imvol,'Gaussian',0,0.00003);

NG4=imnoise(imvol,'Gaussian',0,0.00004);

NG5=imnoise(imvol,'Gaussian',0,0.00005);

NG6=imnoise(imvol,'Gaussian',0,0.00006);

% adding poisson noise to imvol

% if you work on a specific ROI you need to compute bw using max_min_muga

I1=imvol;

I11=uint16(imvol./10);

I12=uint16(imvol./20);

I13=uint16(imvol./30);

I14=uint16(imvol./40);

I15=uint16(imvol./50);

I16=uint16(imvol./60);

bw1=ones(size(I11));

f1 = @(x) imnoise(x,'poisson');

NP1=10*roifilt2(I11,bw1,f1);

NP2=20*roifilt2(I12,bw1,f1);

NP3=30*roifilt2(I13,bw1,f1);

NP4=40*roifilt2(I14,bw1,f1);

NP5=50*roifilt2(I15,bw1,f1);

NP6=60*roifilt2(I16,bw1,f1);

% adding speckle noise to imvol

NS1=imnoise(imvol,'Speckle',0.1);

NS2=imnoise(imvol,'Speckle',0.2);

NS3=imnoise(imvol,'Speckle',0.3);

NS4=imnoise(imvol,'Speckle',0.4);

NS5=imnoise(imvol,'Speckle',0.5);

NS6=imnoise(imvol,'Speckle',0.6);

%% Filtering

%% 1st: filtering gaussian noise

% Median Filtering

FG_MED1=medfilt2(NG1,[5 5]); % FG means Filter gaussian noise, med means using median filter

FG_MED2=medfilt2(NG2,[5 5]);

FG_MED3=medfilt2(NG3,[5 5]);

FG_MED4=medfilt2(NG4,[5 5]);

FG_MED5=medfilt2(NG5,[5 5]);

FG_MED6=medfilt2(NG6,[5 5]);

% Mean Filtering

h1=fspecial('average',5);

FG_AVG1=filter2(h1,NG1); % FG means Filter gaussian noise, AVG means using mean (average) filter

FG_AVG2=filter2(h1,NG2);

FG_AVG3=filter2(h1,NG3);

FG_AVG4=filter2(h1,NG4);

FG_AVG5=filter2(h1,NG5);

FG_AVG6=filter2(h1,NG6);

% Wiener Filtering

FG_WNR1=wiener2(NG1,[5 5]); % FG means Filter gaussian noise, med means using weiner filter

FG_WNR2=wiener2(NG2,[5 5]);

FG_WNR3=wiener2(NG3,[5 5]);

FG_WNR4=wiener2(NG4,[5 5]);

FG_WNR5=wiener2(NG5,[5 5]);

FG_WNR6=wiener2(NG6,[5 5]);

% MMWF Filtering

FG_MMW1=MMWF_2D(NG1,5); % FG means Filter gaussian noise, med means using modified median weiner filter

FG_MMW2=MMWF_2D(NG2,5);

FG_MMW3=MMWF_2D(NG3,5);

FG_MMW4=MMWF_2D(NG4,5);

FG_MMW5=MMWF_2D(NG5,5);

FG_MMW6=MMWF_2D(NG6,5);

%% 2nd: filtering poisson noise

% Median Filtering

FP_MED1=medfilt2(NP1,[5 5]); % FP means Filter possion noise, med means using median filter

FP_MED2=medfilt2(NP2,[5 5]);

FP_MED3=medfilt2(NP3,[5 5]);

FP_MED4=medfilt2(NP4,[5 5]);

FP_MED5=medfilt2(NP5,[5 5]);

FP_MED6=medfilt2(NP6,[5 5]);

% Mean Filtering

h1=fspecial('average',5);

FP_AVG1=filter2(h1,NP1); % FP means Filter possion noise, AVG means using mean (average) filter

FP_AVG2=filter2(h1,NP2);

FP_AVG3=filter2(h1,NP3);

FP_AVG4=filter2(h1,NP4);

FP_AVG5=filter2(h1,NP5);

FP_AVG6=filter2(h1,NP6);

% Wiener Filtering

FP_WNR1=wiener2(NP1,[5 5]); % FP means Filter possion noise, med means using weiner filter

FP_WNR2=wiener2(NP2,[5 5]);

FP_WNR3=wiener2(NP3,[5 5]);

FP_WNR4=wiener2(NP4,[5 5]);

FP_WNR5=wiener2(NP5,[5 5]);

FP_WNR6=wiener2(NP6,[5 5]);

% MMWF Filtering

FP_MMW1=MMWF_2D(NP1,5); % FP means Filter possion noise, med means using modified median weiner filter

FP_MMW2=MMWF_2D(NP2,5);

FP_MMW3=MMWF_2D(NP3,5);

FP_MMW4=MMWF_2D(NP4,5);

FP_MMW5=MMWF_2D(NP5,5);

FP_MMW6=MMWF_2D(NP6,5);

%% 3rd: filtering speckle noise

% Median Filtering

FS_MED1=medfilt2(NS1,[5 5]); % FS means Filter speckle noise, med means using median filter

FS_MED2=medfilt2(NS2,[5 5]);

FS_MED3=medfilt2(NS3,[5 5]);

FS_MED4=medfilt2(NS4,[5 5]);

FS_MED5=medfilt2(NS5,[5 5]);

FS_MED6=medfilt2(NS6,[5 5]);

% Mean Filtering

h1=fspecial('average',5);

FS_AVG1=filter2(h1,NS1); % FS means Filter speckle noise, AVG means using mean (average) filter

FS_AVG2=filter2(h1,NS2);

FS_AVG3=filter2(h1,NS3);

FS_AVG4=filter2(h1,NS4);

FS_AVG5=filter2(h1,NS5);

FS_AVG6=filter2(h1,NS6);

% Wiener Filtering

FS_WNR1=wiener2(NS1,[5 5]); % FS means Filter speckle noise, med means using weiner filter

FS_WNR2=wiener2(NS2,[5 5]);

FS_WNR3=wiener2(NS3,[5 5]);

FS_WNR4=wiener2(NS4,[5 5]);

FS_WNR5=wiener2(NS5,[5 5]);

FS_WNR6=wiener2(NS6,[5 5]);

% MMWF Filtering

FS_MMW1=MMWF_2D(NS1,5); % FS means Filter speckle noise, med means using modified median weiner filter

FS_MMW2=MMWF_2D(NS2,5);

FS_MMW3=MMWF_2D(NS3,5);

FS_MMW4=MMWF_2D(NS4,5);

FS_MMW5=MMWF_2D(NS5,5);

FS_MMW6=MMWF_2D(NS6,5);

%% compute PSNR

%10%

PSNR_G10_MED= round(psnr(imvol, uint16(FG_MED1)));

PSNR_G10_AVG= round(psnr(imvol, uint16(FG_AVG1)));

PSNR_G10_WNR= round(psnr(imvol, uint16(FG_WNR1)));

PSNR_G10_MMW= round(psnr(imvol, uint16(FG_MMW1)));

PSNR_P10_MED= round(psnr(imvol, uint16(FP_MED1)));

PSNR_P10_AVG= round(psnr(imvol, uint16(FP_AVG1)));

PSNR_P10_WNR= round(psnr(imvol, uint16(FP_WNR1)));

PSNR_P10_MMW= round(psnr(imvol, uint16(FP_MMW1)));

PSNR_S10_MED= round(psnr(imvol, uint16(FS_MED1)));

PSNR_S10_AVG= round(psnr(imvol, uint16(FS_AVG1)));

PSNR_S10_WNR= round(psnr(imvol, uint16(FS_WNR1)));

PSNR_S10_MMW= round(psnr(imvol, uint16(FS_MMW1)));

%20%

PSNR_G20_MED= round(psnr(imvol, uint16(FG_MED2)));

PSNR_G20_AVG= round(psnr(imvol, uint16(FG_AVG2)));

PSNR_G20_WNR= round(psnr(imvol, uint16(FG_WNR2)));

PSNR_G20_MMW= round(psnr(imvol, uint16(FG_MMW2)));

PSNR_P20_MED= round(psnr(imvol, uint16(FP_MED2)));

PSNR_P20_AVG= round(psnr(imvol, uint16(FP_AVG2)));

PSNR_P20_WNR= round(psnr(imvol, uint16(FP_WNR2)));

PSNR_P20_MMW= round(psnr(imvol, uint16(FP_MMW2)));

PSNR_S20_MED= round(psnr(imvol, uint16(FS_MED2)));

PSNR_S20_AVG= round(psnr(imvol, uint16(FS_AVG2)));

PSNR_S20_WNR= round(psnr(imvol, uint16(FS_WNR2)));

PSNR_S20_MMW= round(psnr(imvol, uint16(FS_MMW2)));

%30%

PSNR_G30_MED= round(psnr(imvol, uint16(FG_MED3)));

PSNR_G30_AVG= round(psnr(imvol, uint16(FG_AVG3)));

PSNR_G30_WNR= round(psnr(imvol, uint16(FG_WNR3)));

PSNR_G30_MMW= round(psnr(imvol, uint16(FG_MMW3)));

PSNR_P30_MED= round(psnr(imvol, uint16(FP_MED3)));

PSNR_P30_AVG= round(psnr(imvol, uint16(FP_AVG3)));

PSNR_P30_WNR= round(psnr(imvol, uint16(FP_WNR3)));

PSNR_P30_MMW= round(psnr(imvol, uint16(FP_MMW3)));

PSNR_S30_MED= round(psnr(imvol, uint16(FS_MED3)));

PSNR_S30_AVG= round(psnr(imvol, uint16(FS_AVG3)));

PSNR_S30_WNR= round(psnr(imvol, uint16(FS_WNR3)));

PSNR_S30_MMW= round(psnr(imvol, uint16(FS_MMW3)));

%40%

PSNR_G40_MED= round(psnr(imvol, uint16(FG_MED4)));

PSNR_G40_AVG= round(psnr(imvol, uint16(FG_AVG4)));

PSNR_G40_WNR= round(psnr(imvol, uint16(FG_WNR4)));

PSNR_G40_MMW= round(psnr(imvol, uint16(FG_MMW4)));

PSNR_P40_MED= round(psnr(imvol, uint16(FP_MED4)));

PSNR_P40_AVG= round(psnr(imvol, uint16(FP_AVG4)));

PSNR_P40_WNR= round(psnr(imvol, uint16(FP_WNR4)));

PSNR_P40_MMW= round(psnr(imvol, uint16(FP_MMW4)));

PSNR_S40_MED= round(psnr(imvol, uint16(FS_MED4)));

PSNR_S40_AVG= round(psnr(imvol, uint16(FS_AVG4)));

PSNR_S40_WNR= round(psnr(imvol, uint16(FS_WNR4)));

PSNR_S40_MMW= round(psnr(imvol, uint16(FS_MMW4)));

%50%

PSNR_G50_MED= round(psnr(imvol, uint16(FG_MED5)));

PSNR_G50_AVG= round(psnr(imvol, uint16(FG_AVG5)));

PSNR_G50_WNR= round(psnr(imvol, uint16(FG_WNR5)));

PSNR_G50_MMW= round(psnr(imvol, uint16(FG_MMW5)));

PSNR_P50_MED= round(psnr(imvol, uint16(FP_MED5)));

PSNR_P50_AVG= round(psnr(imvol, uint16(FP_AVG5)));

PSNR_P50_WNR= round(psnr(imvol, uint16(FP_WNR5)));

PSNR_P50_MMW= round(psnr(imvol, uint16(FP_MMW5)));

PSNR_S50_MED= round(psnr(imvol, uint16(FS_MED5)));

PSNR_S50_AVG= round(psnr(imvol, uint16(FS_AVG5)));

PSNR_S50_WNR= round(psnr(imvol, uint16(FS_WNR5)));

PSNR_S50_MMW= round(psnr(imvol, uint16(FS_MMW5)));

%60%

PSNR_G60_MED= round(psnr(imvol, uint16(FG_MED6)));

PSNR_G60_AVG= round(psnr(imvol, uint16(FG_AVG6)));

PSNR_G60_WNR= round(psnr(imvol, uint16(FG_WNR6)));

PSNR_G60_MMW= round(psnr(imvol, uint16(FG_MMW6)));

PSNR_P60_MED= round(psnr(imvol, uint16(FP_MED6)));

PSNR_P60_AVG= round(psnr(imvol, uint16(FP_AVG6)));

PSNR_P60_WNR= round(psnr(imvol, uint16(FP_WNR6)));

PSNR_P60_MMW= round(psnr(imvol, uint16(FP_MMW6)));

PSNR_S60_MED= round(psnr(imvol, uint16(FS_MED6)));

PSNR_S60_AVG= round(psnr(imvol, uint16(FS_AVG6)));

PSNR_S60_WNR= round(psnr(imvol, uint16(FS_WNR6)));

PSNR_S60_MMW= round(psnr(imvol, uint16(FS_MMW6)));

%% Compute MSE

%10%

MSE_G10_MED= round(immse(imvol, uint16(FG_MED1)));

MSE_G10_AVG= round(immse(imvol, uint16(FG_AVG1)));

MSE_G10_WNR= round(immse(imvol, uint16(FG_WNR1)));

MSE_G10_MMW= round(immse(imvol, uint16(FG_MMW1)));

MSE_P10_MED= round(immse(imvol, uint16(FP_MED1)));

MSE_P10_AVG= round(immse(imvol, uint16(FP_AVG1)));

MSE_P10_WNR= round(immse(imvol, uint16(FP_WNR1)));

MSE_P10_MMW= round(immse(imvol, uint16(FP_MMW1)));

MSE_S10_MED= round(immse(imvol, uint16(FS_MED1)));

MSE_S10_AVG= round(immse(imvol, uint16(FS_AVG1)));

MSE_S10_WNR= round(immse(imvol, uint16(FS_WNR1)));

MSE_S10_MMW= round(immse(imvol, uint16(FS_MMW1)));

%20%

MSE_G20_MED= round(immse(imvol, uint16(FG_MED2)));

MSE_G20_AVG= round(immse(imvol, uint16(FG_AVG2)));

MSE_G20_WNR= round(immse(imvol, uint16(FG_WNR2)));

MSE_G20_MMW= round(immse(imvol, uint16(FG_MMW2)));

MSE_P20_MED= round(immse(imvol, uint16(FP_MED2)));

MSE_P20_AVG= round(immse(imvol, uint16(FP_AVG2)));

MSE_P20_WNR= round(immse(imvol, uint16(FP_WNR2)));

MSE_P20_MMW= round(immse(imvol, uint16(FP_MMW2)));

MSE_S20_MED= round(immse(imvol, uint16(FS_MED2)));

MSE_S20_AVG= round(immse(imvol, uint16(FS_AVG2)));

MSE_S20_WNR= round(immse(imvol, uint16(FS_WNR2)));

MSE_S20_MMW= round(immse(imvol, uint16(FS_MMW2)));

%30%

MSE_G30_MED= round(immse(imvol, uint16(FG_MED3)));

MSE_G30_AVG= round(immse(imvol, uint16(FG_AVG3)));

MSE_G30_WNR= round(immse(imvol, uint16(FG_WNR3)));

MSE_G30_MMW= round(immse(imvol, uint16(FG_MMW3)));

MSE_P30_MED= round(immse(imvol, uint16(FP_MED3)));

MSE_P30_AVG= round(immse(imvol, uint16(FP_AVG3)));

MSE_P30_WNR= round(immse(imvol, uint16(FP_WNR3)));

MSE_P30_MMW= round(immse(imvol, uint16(FP_MMW3)));

MSE_S30_MED= round(immse(imvol, uint16(FS_MED3)));

MSE_S30_AVG= round(immse(imvol, uint16(FS_AVG3)));

MSE_S30_WNR= round(immse(imvol, uint16(FS_WNR3)));

MSE_S30_MMW= round(immse(imvol, uint16(FS_MMW3)));

%40%

MSE_G40_MED= round(immse(imvol, uint16(FG_MED4)));

MSE_G40_AVG= round(immse(imvol, uint16(FG_AVG4)));

MSE_G40_WNR= round(immse(imvol, uint16(FG_WNR4)));

MSE_G40_MMW= round(immse(imvol, uint16(FG_MMW4)));

MSE_P40_MED= round(immse(imvol, uint16(FP_MED4)));

MSE_P40_AVG= round(immse(imvol, uint16(FP_AVG4)));

MSE_P40_WNR= round(immse(imvol, uint16(FP_WNR4)));

MSE_P40_MMW= round(immse(imvol, uint16(FP_MMW4)));

MSE_S40_MED= round(immse(imvol, uint16(FS_MED4)));

MSE_S40_AVG= round(immse(imvol, uint16(FS_AVG4)));

MSE_S40_WNR= round(immse(imvol, uint16(FS_WNR4)));

MSE_S40_MMW= round(immse(imvol, uint16(FS_MMW4)));

%50%

MSE_G50_MED= round(immse(imvol, uint16(FG_MED5)));

MSE_G50_AVG= round(immse(imvol, uint16(FG_AVG5)));

MSE_G50_WNR= round(immse(imvol, uint16(FG_WNR5)));

MSE_G50_MMW= round(immse(imvol, uint16(FG_MMW5)));

MSE_P50_MED= round(immse(imvol, uint16(FP_MED5)));

MSE_P50_AVG= round(immse(imvol, uint16(FP_AVG5)));

MSE_P50_WNR= round(immse(imvol, uint16(FP_WNR5)));

MSE_P50_MMW= round(immse(imvol, uint16(FP_MMW5)));

MSE_S50_MED= round(immse(imvol, uint16(FS_MED5)));

MSE_S50_AVG= round(immse(imvol, uint16(FS_AVG5)));

MSE_S50_WNR= round(immse(imvol, uint16(FS_WNR5)));

MSE_S50_MMW= round(immse(imvol, uint16(FS_MMW5)));

%60%

MSE_G60_MED= round(immse(imvol, uint16(FG_MED6)));

MSE_G60_AVG= round(immse(imvol, uint16(FG_AVG6)));

MSE_G60_WNR= round(immse(imvol, uint16(FG_WNR6)));

MSE_G60_MMW= round(immse(imvol, uint16(FG_MMW6)));

MSE_P60_MED= round(immse(imvol, uint16(FP_MED6)));

MSE_P60_AVG= round(immse(imvol, uint16(FP_AVG6)));

MSE_P60_WNR= round(immse(imvol, uint16(FP_WNR6)));

MSE_P60_MMW= round(immse(imvol, uint16(FP_MMW6)));

MSE_S60_MED= round(immse(imvol, uint16(FS_MED6)));

MSE_S60_AVG= round(immse(imvol, uint16(FS_AVG6)));

MSE_S60_WNR= round(immse(imvol, uint16(FS_WNR6)));

MSE_S60_MMW= round(immse(imvol, uint16(FS_MMW6)));

%% CNR

%10%

[CNR_G10_MED, CR_G10_MED]= (cnr(imvol,bw1, (FG_MED1),NG1));

[CNR_G10_AVG, CR_G10_AVG]= (cnr(imvol,bw1, (FG_AVG1),NG1));

[CNR_G10_WNR, CR_G10_WNR]= (cnr(imvol,bw1, (FG_WNR1),NG1));

[CNR_G10_MMW, CR_G10_MMW]= (cnr(imvol,bw1, (FG_MMW1),NG1));

[CNR_P10_MED, CR_P10_MED]= (cnr(imvol,bw1, (FP_MED1),NP1));

[CNR_P10_AVG, CR_P10_AVG]= (cnr(imvol,bw1, (FP_AVG1),NP1));

[CNR_P10_WNR, CR_P10_WNR]= (cnr(imvol,bw1, (FP_WNR1),NP1));

[CNR_P10_MMW, CR_P10_MMW]= (cnr(imvol,bw1, (FP_MMW1),NP1));

[CNR_S10_MED, CR_S10_MED]= (cnr(imvol,bw1, (FS_MED1),NS1));

[CNR_S10_AVG, CR_S10_AVG]= (cnr(imvol,bw1, (FS_AVG1),NS1));

[CNR_S10_WNR, CR_S10_WNR]= (cnr(imvol,bw1, (FS_WNR1),NS1));

[CNR_S10_MMW, CR_S10_MMW]= (cnr(imvol,bw1, (FS_MMW1),NS1));

%20%

[CNR_G20_MED, CR_G20_MED]= (cnr(imvol,bw1, (FG_MED2),NG2));

[CNR_G20_AVG, CR_G20_AVG]= (cnr(imvol,bw1, (FG_AVG2),NG2));

[CNR_G20_WNR, CR_G20_WNR]= (cnr(imvol,bw1, (FG_WNR2),NG2));

[CNR_G20_MMW, CR_G20_MMW]= (cnr(imvol,bw1, (FG_MMW2),NG2));

[CNR_P20_MED, CR_P20_MED]= (cnr(imvol,bw1, (FP_MED2),NP2));

[CNR_P20_AVG, CR_P20_AVG]= (cnr(imvol,bw1, (FP_AVG2),NP2));

[CNR_P20_WNR, CR_P20_WNR]= (cnr(imvol,bw1, (FP_WNR2),NP2));

[CNR_P20_MMW, CR_P20_MMW]= (cnr(imvol,bw1, (FP_MMW2),NP2));

[CNR_S20_MED, CR_S20_MED]= (cnr(imvol,bw1, (FS_MED2),NS2));

[CNR_S20_AVG, CR_S20_AVG]= (cnr(imvol,bw1, (FS_AVG2),NS2));

[CNR_S20_WNR, CR_S20_WNR]= (cnr(imvol,bw1, (FS_WNR2),NS2));

[CNR_S20_MMW, CR_S20_MMW]= (cnr(imvol,bw1, (FS_MMW2),NS2));

%30%

[CNR_G30_MED, CR_G30_MED]= (cnr(imvol,bw1, (FG_MED3),NG3));

[CNR_G30_AVG, CR_G30_AVG]= (cnr(imvol,bw1, (FG_AVG3),NG3));

[CNR_G30_WNR, CR_G30_WNR]= (cnr(imvol,bw1, (FG_WNR3),NG3));

[CNR_G30_MMW, CR_G30_MMW]= (cnr(imvol,bw1, (FG_MMW3),NG3));

[CNR_P30_MED, CR_P30_MED]= (cnr(imvol,bw1, (FP_MED3),NP3));

[CNR_P30_AVG, CR_P30_AVG]= (cnr(imvol,bw1, (FP_AVG3),NP3));

[CNR_P30_WNR, CR_P30_WNR]= (cnr(imvol,bw1, (FP_WNR3),NP3));

[CNR_P30_MMW, CR_P30_MMW]= (cnr(imvol,bw1, (FP_MMW3),NP3));

[CNR_S30_MED, CR_S30_MED]= (cnr(imvol,bw1, (FS_MED3),NS3));

[CNR_S30_AVG, CR_S30_AVG]= (cnr(imvol,bw1, (FS_AVG3),NS3));

[CNR_S30_WNR, CR_S30_WNR]= (cnr(imvol,bw1, (FS_WNR3),NS3));

[CNR_S30_MMW, CR_S30_MMW]= (cnr(imvol,bw1, (FS_MMW3),NS3));

%40%

[CNR_G40_MED, CR_G40_MED]= (cnr(imvol,bw1, (FG_MED4),NG4));

[CNR_G40_AVG, CR_G40_AVG]= (cnr(imvol,bw1, (FG_AVG4),NG4));

[CNR_G40_WNR, CR_G40_WNR]= (cnr(imvol,bw1, (FG_WNR4),NG4));

[CNR_G40_MMW, CR_G40_MMW]= (cnr(imvol,bw1, (FG_MMW4),NG4));

[CNR_P40_MED, CR_P40_MED]= (cnr(imvol,bw1, (FP_MED4),NP4));

[CNR_P40_AVG, CR_P40_AVG]= (cnr(imvol,bw1, (FP_AVG4),NP4));

[CNR_P40_WNR, CR_P40_WNR]= (cnr(imvol,bw1, (FP_WNR4),NP4));

[CNR_P40_MMW, CR_P40_MMW]= (cnr(imvol,bw1, (FP_MMW4),NP4));

[CNR_S40_MED, CR_S40_MED]= (cnr(imvol,bw1, (FS_MED4),NS4));

[CNR_S40_AVG, CR_S40_AVG]= (cnr(imvol,bw1, (FS_AVG4),NS4));

[CNR_S40_WNR, CR_S40_WNR]= (cnr(imvol,bw1, (FS_WNR4),NS4));

[CNR_S40_MMW, CR_S40_MMW]= (cnr(imvol,bw1, (FS_MMW4),NS4));

%50%

[CNR_G50_MED, CR_G50_MED]= (cnr(imvol,bw1, (FG_MED5),NG5));

[CNR_G50_AVG, CR_G50_AVG]= (cnr(imvol,bw1, (FG_AVG5),NG5));

[CNR_G50_WNR, CR_G50_WNR]= (cnr(imvol,bw1, (FG_WNR5),NG5));

[CNR_G50_MMW, CR_G50_MMW]= (cnr(imvol,bw1, (FG_MMW5),NG5));

[CNR_P50_MED, CR_P50_MED]= (cnr(imvol,bw1, (FP_MED5),NP5));

[CNR_P50_AVG, CR_P50_AVG]= (cnr(imvol,bw1, (FP_AVG5),NP5));

[CNR_P50_WNR, CR_P50_WNR]= (cnr(imvol,bw1, (FP_WNR5),NP5));

[CNR_P50_MMW, CR_P50_MMW]= (cnr(imvol,bw1, (FP_MMW5),NP5));

[CNR_S50_MED, CR_S50_MED]= (cnr(imvol,bw1, (FS_MED5),NS5));

[CNR_S50_AVG, CR_S50_AVG]= (cnr(imvol,bw1, (FS_AVG5),NS5));

[CNR_S50_WNR, CR_S50_WNR]= (cnr(imvol,bw1, (FS_WNR5),NS5));

[CNR_S50_MMW, CR_S50_MMW]= (cnr(imvol,bw1, (FS_MMW5),NS5));

%60%

[CNR_G60_MED, CR_G60_MED]= (cnr(imvol,bw1, (FG_MED6),NG6));

[CNR_G60_AVG, CR_G60_AVG]= (cnr(imvol,bw1, (FG_AVG6),NG6));

[CNR_G60_WNR, CR_G60_WNR]= (cnr(imvol,bw1, (FG_WNR6),NG6));

[CNR_G60_MMW, CR_G60_MMW]= (cnr(imvol,bw1, (FG_MMW6),NG6));

[CNR_P60_MED, CR_P60_MED]= (cnr(imvol,bw1, (FP_MED6),NP6));

[CNR_P60_AVG, CR_P60_AVG]= (cnr(imvol,bw1, (FP_AVG6),NP6));

[CNR_P60_WNR, CR_P60_WNR]= (cnr(imvol,bw1, (FP_WNR6),NP6));

[CNR_P60_MMW, CR_P60_MMW]= (cnr(imvol,bw1, (FP_MMW6),NP6));

[CNR_S60_MED, CR_S60_MED]= (cnr(imvol,bw1, (FS_MED6),NS6));

[CNR_S60_AVG, CR_S60_AVG]= (cnr(imvol,bw1, (FS_AVG6),NS6));

[CNR_S60_WNR, CR_S60_WNR]= (cnr(imvol,bw1, (FS_WNR6),NS6));

[CNR_S60_MMW, CR_S60_MMW]= (cnr(imvol,bw1, (FS_MMW6),NS6));

%% Degree of smoothness DOS

%10%

DOS_G10_MED= round(dos(FG_MED1));

DOS_G10_AVG= round(dos(FG_AVG1));

DOS_G10_WNR= round(dos(FG_WNR1));

DOS_G10_MMW= round(dos(FG_MMW1));

DOS_P10_MED= round(dos(FP_MED1));

DOS_P10_AVG= round(dos(FP_AVG1));

DOS_P10_WNR= round(dos(FP_WNR1));

DOS_P10_MMW= round(dos(FP_MMW1));

DOS_S10_MED= round(dos(FS_MED1));

DOS_S10_AVG= round(dos(FS_AVG1));

DOS_S10_WNR= round(dos(FS_WNR1));

DOS_S10_MMW= round(dos(FS_MMW1));

%20%

DOS_G20_MED= round(dos(FG_MED2));

DOS_G20_AVG= round(dos(FG_AVG2));

DOS_G20_WNR= round(dos(FG_WNR2));

DOS_G20_MMW= round(dos(FG_MMW2));

DOS_P20_MED= round(dos(FP_MED2));

DOS_P20_AVG= round(dos(FP_AVG2));

DOS_P20_WNR= round(dos(FP_WNR2));

DOS_P20_MMW= round(dos(FP_MMW2));

DOS_S20_MED= round(dos(FS_MED2));

DOS_S20_AVG= round(dos(FS_AVG2));

DOS_S20_WNR= round(dos(FS_WNR2));

DOS_S20_MMW= round(dos(FS_MMW2));

%10%

DOS_G30_MED= round(dos(FG_MED3));

DOS_G30_AVG= round(dos(FG_AVG3));

DOS_G30_WNR= round(dos(FG_WNR3));

DOS_G30_MMW= round(dos(FG_MMW3));

DOS_P30_MED= round(dos(FP_MED3));

DOS_P30_AVG= round(dos(FP_AVG3));

DOS_P30_WNR= round(dos(FP_WNR3));

DOS_P30_MMW= round(dos(FP_MMW3));

DOS_S30_MED= round(dos(FS_MED3));

DOS_S30_AVG= round(dos(FS_AVG3));

DOS_S30_WNR= round(dos(FS_WNR3));

DOS_S30_MMW= round(dos(FS_MMW3));

%10%

DOS_G40_MED= round(dos(FG_MED4));

DOS_G40_AVG= round(dos(FG_AVG4));

DOS_G40_WNR= round(dos(FG_WNR4));

DOS_G40_MMW= round(dos(FG_MMW4));

DOS_P40_MED= round(dos(FP_MED4));

DOS_P40_AVG= round(dos(FP_AVG4));

DOS_P40_WNR= round(dos(FP_WNR4));

DOS_P40_MMW= round(dos(FP_MMW4));

DOS_S40_MED= round(dos(FS_MED4));

DOS_S40_AVG= round(dos(FS_AVG4));

DOS_S40_WNR= round(dos(FS_WNR4));

DOS_S40_MMW= round(dos(FS_MMW4));

%10

DOS_G50_MED= round(dos(FG_MED5));

DOS_G50_AVG= round(dos(FG_AVG5));

DOS_G50_WNR= round(dos(FG_WNR5));

DOS_G50_MMW= round(dos(FG_MMW5));

DOS_P50_MED= round(dos(FP_MED5));

DOS_P50_AVG= round(dos(FP_AVG5));

DOS_P50_WNR= round(dos(FP_WNR5));

DOS_P50_MMW= round(dos(FP_MMW5));

DOS_S50_MED= round(dos(FS_MED5));

DOS_S50_AVG= round(dos(FS_AVG5));

DOS_S50_WNR= round(dos(FS_WNR5));

DOS_S50_MMW= round(dos(FS_MMW5));

%10

DOS_G60_MED= round(dos(FG_MED6));

DOS_G60_AVG= round(dos(FG_AVG6));

DOS_G60_WNR= round(dos(FG_WNR6));

DOS_G60_MMW= round(dos(FG_MMW6));

DOS_P60_MED= round(dos(FP_MED6));

DOS_P60_AVG= round(dos(FP_AVG6));

DOS_P60_WNR= round(dos(FP_WNR6));

DOS_P60_MMW= round(dos(FP_MMW6));

DOS_S60_MED= round(dos(FS_MED6));

DOS_S60_AVG= round(dos(FS_AVG6));

DOS_S60_WNR= round(dos(FS_WNR6));

DOS_S60_MMW= round(dos(FS_MMW6));

%%%

%% show 10% results

% show 10% noise and filtering results

hf=figure

subplot(3,6,1);imshow(imvol,[]);title('Original','Fontsize',12);colormap jet;

subplot(3,6,2);imshow(NG1,[]);title('Gaussian noise 10%','Fontsize',12);colormap jet;

ha=subplot(3,6,3);imshow(FG_MED1,[]);title('Median filter','Fontsize',12);colormap jet;

pos = get(ha, 'position');pos(2)=pos(2)-0.08;pos(1)=pos(1)+0.01;

txt=strcat('PSNR= ',num2str(PSNR_G10_MED),', CNR= ',num2str(CNR_G10_MED));

annotation(hf, 'textbox', pos, 'String', num2str(txt), 'vert', 'bottom', 'EdgeColor','none','FontSize',12,'FitBoxToText','off');

ha=subplot(3,6,4);imshow(FG_AVG1,[]);title('Mean filter','Fontsize',12);colormap jet;

pos = get(ha, 'position');pos(2)=pos(2)-0.08;pos(1)=pos(1)+0.01;

txt=strcat('PSNR= ',num2str(PSNR_G10_AVG),', CNR= ',num2str(CNR_G10_AVG));

annotation(hf, 'textbox', pos, 'String', num2str(txt), 'vert', 'bottom', 'EdgeColor','none','FontSize',12,'FitBoxToText','off');

ha=subplot(3,6,5);imshow(FG_WNR1,[]);title('Weiner filter','Fontsize',12);colormap jet;

pos = get(ha, 'position');pos(2)=pos(2)-0.08;pos(1)=pos(1)+0.01;

txt=strcat('PSNR= ',num2str(PSNR_G10_WNR),', CNR= ',num2str(CNR_G10_WNR));

annotation(hf, 'textbox', pos, 'String', num2str(txt), 'vert', 'bottom', 'EdgeColor','none','FontSize',12,'FitBoxToText','off');

ha=subplot(3,6,6);imshow(FG_MMW1,[]);title('MMWF filer','Fontsize',12);colormap jet;

pos = get(ha, 'position');pos(2)=pos(2)-0.08;pos(1)=pos(1)+0.01;

txt=strcat('PSNR= ',num2str(PSNR_G10_MMW),', CNR= ',num2str(CNR_G10_MMW));

annotation(hf, 'textbox', pos, 'String', num2str(txt), 'vert', 'bottom', 'EdgeColor','none','FontSize',12,'FitBoxToText','off');

subplot(3,6,7);imshow(imvol,[]);title('Original','Fontsize',12);colormap jet;

subplot(3,6,8);imshow(NP1,[]);title(["Possion noise=","imnoise(Pixel Value/10)*10"],'Fontsize',12);colormap jet;

ha=subplot(3,6,9),imshow(FP_MED1,[]);title('Median filter','Fontsize',12);colormap jet;

pos = get(ha, 'position');pos(2)=pos(2)-0.08;pos(1)=pos(1)+0.01;

txt=strcat('PSNR= ',num2str(PSNR_P10_MED),', CNR= ',num2str(CNR_P10_MED));

annotation(hf, 'textbox', pos, 'String', num2str(txt), 'vert', 'bottom', 'EdgeColor','none','FontSize',12,'FitBoxToText','off');

ha=subplot(3,6,10);imshow(FP_AVG1,[]);title('Mean filter','Fontsize',12);colormap jet;

pos = get(ha, 'position');pos(2)=pos(2)-0.08;pos(1)=pos(1)+0.01;

txt=strcat('PSNR= ',num2str(PSNR_P10_AVG),', CNR= ',num2str(CNR_P10_AVG));

annotation(hf, 'textbox', pos, 'String', num2str(txt), 'vert', 'bottom', 'EdgeColor','none','FontSize',12,'FitBoxToText','off');

ha=subplot(3,6,11);imshow(FP_WNR1,[]);title('Weiner filter','Fontsize',12);colormap jet;

pos = get(ha, 'position');pos(2)=pos(2)-0.08;pos(1)=pos(1)+0.01;

txt=strcat('PSNR= ',num2str(PSNR_P10_WNR),', CNR= ',num2str(CNR_P10_WNR));

annotation(hf, 'textbox', pos, 'String', num2str(txt), 'vert', 'bottom', 'EdgeColor','none','FontSize',12,'FitBoxToText','off');

ha=subplot(3,6,12);imshow(FP_MMW1,[]);title('MMWF filer','Fontsize',12);colormap jet;

pos = get(ha, 'position');pos(2)=pos(2)-0.08;pos(1)=pos(1)+0.01;

txt=strcat('PSNR= ',num2str(PSNR_P10_MMW),', CNR= ',num2str(CNR_P10_MMW));

annotation(hf, 'textbox', pos, 'String', num2str(txt), 'vert', 'bottom', 'EdgeColor','none','FontSize',12,'FitBoxToText','off');

subplot(3,6,13);imshow(imvol,[]);title('Original','Fontsize',12);colormap jet;

subplot(3,6,14);imshow(NS1,[]);title('Speckle noise 10%','Fontsize',12);colormap jet;

ha=subplot(3,6,15);imshow(FS_MED1,[]);title('Median filter','Fontsize',12);colormap jet;

pos = get(ha, 'position');pos(2)=pos(2)-0.08;pos(1)=pos(1)+0.01;

txt=strcat('PSNR= ',num2str(PSNR_S10_MED),', CNR= ',num2str(CNR_S10_MED));

annotation(hf, 'textbox', pos, 'String', num2str(txt), 'vert', 'bottom', 'EdgeColor','none','FontSize',12,'FitBoxToText','off');

ha=subplot(3,6,16);imshow(FS_AVG1,[]);title('Mean filter','Fontsize',12);colormap jet;

pos = get(ha, 'position');pos(2)=pos(2)-0.08;pos(1)=pos(1)+0.01;

txt=strcat('PSNR= ',num2str(PSNR_S10_AVG),', CNR= ',num2str(CNR_S10_AVG));

annotation(hf, 'textbox', pos, 'String', num2str(txt), 'vert', 'bottom', 'EdgeColor','none','FontSize',12,'FitBoxToText','off');

ha=subplot(3,6,17);imshow(FS_WNR1,[]);title('Weiner filter','Fontsize',12);colormap jet;

pos = get(ha, 'position');pos(2)=pos(2)-0.08;pos(1)=pos(1)+0.01;

txt=strcat('PSNR= ',num2str(PSNR_S10_WNR),', CNR= ',num2str(CNR_S10_WNR));

annotation(hf, 'textbox', pos, 'String', num2str(txt), 'vert', 'bottom', 'EdgeColor','none','FontSize',12,'FitBoxToText','off');

ha=subplot(3,6,18);imshow(FS_MMW1,[]);title('MMWF filer','Fontsize',12);colormap jet;

pos = get(ha, 'position');pos(2)=pos(2)-0.08;pos(1)=pos(1)+0.01;

txt=strcat('PSNR= ',num2str(PSNR_S10_MMW),', CNR= ',num2str(CNR_S10_MMW));

annotation(hf, 'textbox', pos, 'String', num2str(txt), 'vert', 'bottom', 'EdgeColor','none','FontSize',12,'FitBoxToText','off');

% show 30% noise and filtering results

hf=figure

subplot(3,6,1),imshow(imvol,[]);title('Original','Fontsize',12);colormap jet;

subplot(3,6,2),imshow(NG3,[]);title('Gaussian noise 30%','Fontsize',12);colormap jet;

ha=subplot(3,6,3),imshow(FG_MED3,[]);title('Median filter','Fontsize',12);colormap jet;

pos = get(ha, 'position');pos(2)=pos(2)-0.08;pos(1)=pos(1)+0.01;

txt=strcat('PSNR= ',num2str(PSNR_G30_MED),', CNR= ',num2str(CNR_G30_MED));

annotation(hf, 'textbox', pos, 'String', num2str(txt), 'vert', 'bottom', 'EdgeColor','none','FontSize',12,'FitBoxToText','off');

ha=subplot(3,6,4),imshow(FG_AVG3,[]);title('Mean filter','Fontsize',12);colormap jet;

pos = get(ha, 'position');pos(2)=pos(2)-0.08;pos(1)=pos(1)+0.01;

txt=strcat('PSNR= ',num2str(PSNR_G30_AVG),', CNR= ',num2str(CNR_G30_AVG));

annotation(hf, 'textbox', pos, 'String', num2str(txt), 'vert', 'bottom', 'EdgeColor','none','FontSize',12,'FitBoxToText','off');

ha=subplot(3,6,5);imshow(FG_WNR3,[]);title('Weiner filter','Fontsize',12);colormap jet;

pos = get(ha, 'position');pos(2)=pos(2)-0.08;pos(1)=pos(1)+0.01;

txt=strcat('PSNR= ',num2str(PSNR_G30_WNR),', CNR= ',num2str(CNR_G30_WNR));

annotation(hf, 'textbox', pos, 'String', num2str(txt), 'vert', 'bottom', 'EdgeColor','none','FontSize',12,'FitBoxToText','off');

ha=subplot(3,6,6);imshow(FG_MMW3,[]);title('MMWF filer','Fontsize',12);colormap jet;

pos = get(ha, 'position');pos(2)=pos(2)-0.08;pos(1)=pos(1)+0.01;

txt=strcat('PSNR= ',num2str(PSNR_G30_MMW),', CNR= ',num2str(CNR_G30_MMW));

annotation(hf, 'textbox', pos, 'String', num2str(txt), 'vert', 'bottom', 'EdgeColor','none','FontSize',12,'FitBoxToText','off');

subplot(3,6,7);imshow(imvol,[]);title('Original','Fontsize',12);colormap jet;

subplot(3,6,8);imshow(NP3,[]);title(["Possion noise=","imnoise(Pixel Value/30)*30"],'Fontsize',12);colormap jet;

ha=subplot(3,6,9);imshow(FP_MED3,[]);title('Median filter','Fontsize',12);colormap jet;

pos = get(ha, 'position');pos(2)=pos(2)-0.08;pos(1)=pos(1)+0.01;

txt=strcat('PSNR= ',num2str(PSNR_P30_MED),', CNR= ',num2str(CNR_P30_MED));

annotation(hf, 'textbox', pos, 'String', num2str(txt), 'vert', 'bottom', 'EdgeColor','none','FontSize',12,'FitBoxToText','off');

ha=subplot(3,6,10);imshow(FP_AVG3,[]);title('Mean filter','Fontsize',12);colormap jet;

pos = get(ha, 'position');pos(2)=pos(2)-0.08;pos(1)=pos(1)+0.01;

txt=strcat('PSNR= ',num2str(PSNR_P30_AVG),', CNR= ',num2str(CNR_P30_AVG));

annotation(hf, 'textbox', pos, 'String', num2str(txt), 'vert', 'bottom', 'EdgeColor','none','FontSize',12,'FitBoxToText','off');

ha=subplot(3,6,11);imshow(FP_WNR3,[]);title('Weiner filter','Fontsize',12);colormap jet;

pos = get(ha, 'position');pos(2)=pos(2)-0.08;pos(1)=pos(1)+0.01;

txt=strcat('PSNR= ',num2str(PSNR_P30_WNR),', CNR= ',num2str(CNR_P30_WNR));

annotation(hf, 'textbox', pos, 'String', num2str(txt), 'vert', 'bottom', 'EdgeColor','none','FontSize',12,'FitBoxToText','off');

ha=subplot(3,6,12);imshow(FP_MMW3,[]);title('MMWF filer','Fontsize',12);colormap jet;

pos = get(ha, 'position');pos(2)=pos(2)-0.08;pos(1)=pos(1)+0.01;

txt=strcat('PSNR= ',num2str(PSNR_P30_MMW),', CNR= ',num2str(CNR_P30_MMW));

annotation(hf, 'textbox', pos, 'String', num2str(txt), 'vert', 'bottom', 'EdgeColor','none','FontSize',12,'FitBoxToText','off');

subplot(3,6,13);imshow(imvol,[]);title('Original','Fontsize',12);colormap jet;

subplot(3,6,14);imshow(NS3,[]);title('Speckle noise 30%','Fontsize',12);colormap jet;

ha=subplot(3,6,15);imshow(FS_MED3,[]);title('Median filter','Fontsize',12);colormap jet;

pos = get(ha, 'position');pos(2)=pos(2)-0.08;pos(1)=pos(1)+0.01;

txt=strcat('PSNR= ',num2str(PSNR_S30_MED),', CNR= ',num2str(CNR_S30_MED));

annotation(hf, 'textbox', pos, 'String', num2str(txt), 'vert', 'bottom', 'EdgeColor','none','FontSize',12,'FitBoxToText','off');

ha=subplot(3,6,16);imshow(FS_AVG3,[]);title('Mean filter','Fontsize',12);colormap jet;

pos = get(ha, 'position');pos(2)=pos(2)-0.08;pos(1)=pos(1)+0.01;

txt=strcat('PSNR= ',num2str(PSNR_S30_AVG),', CNR= ',num2str(CNR_S30_AVG));

annotation(hf, 'textbox', pos, 'String', num2str(txt), 'vert', 'bottom', 'EdgeColor','none','FontSize',12,'FitBoxToText','off');

ha=subplot(3,6,17);imshow(FS_WNR3,[]);title('Weiner filter','Fontsize',12);colormap jet;

pos = get(ha, 'position');pos(2)=pos(2)-0.08;pos(1)=pos(1)+0.01;

txt=strcat('PSNR= ',num2str(PSNR_S30_WNR),', CNR= ',num2str(CNR_S30_WNR));

annotation(hf, 'textbox', pos, 'String', num2str(txt), 'vert', 'bottom', 'EdgeColor','none','FontSize',12,'FitBoxToText','off');

ha=subplot(3,6,18);imshow(FS_MMW3,[]);title('MMWF filer','Fontsize',12);colormap jet;

pos = get(ha, 'position');pos(2)=pos(2)-0.08;pos(1)=pos(1)+0.01;

txt=strcat('PSNR= ',num2str(PSNR_S30_MMW),', CNR= ',num2str(CNR_S30_MMW));

annotation(hf, 'textbox', pos, 'String', num2str(txt), 'vert', 'bottom', 'EdgeColor','none','FontSize',12,'FitBoxToText','off');

% show 60% noise and filtering results

hf=figure

subplot(3,6,1);imshow(imvol,[]);title('Original','Fontsize',12);colormap jet;

subplot(3,6,2);imshow(NG6,[]);title('Gaussian noise 60%','Fontsize',12);colormap jet;

ha=subplot(3,6,3);imshow(FG_MED6,[]);title('Median filter','Fontsize',12);colormap jet;

pos = get(ha, 'position');pos(2)=pos(2)-0.08;pos(1)=pos(1)+0.01;

txt=strcat('PSNR= ',num2str(PSNR_G60_MED),', CNR= ',num2str(CNR_G60_MED));

annotation(hf, 'textbox', pos, 'String', num2str(txt), 'vert', 'bottom', 'EdgeColor','none','FontSize',12,'FitBoxToText','off');

ha=subplot(3,6,4);imshow(FG_AVG6,[]);title('Mean filter','Fontsize',12);colormap jet;

pos = get(ha, 'position');pos(2)=pos(2)-0.08;pos(1)=pos(1)+0.01;

txt=strcat('PSNR= ',num2str(PSNR_G60_AVG),', CNR= ',num2str(CNR_G60_AVG));

annotation(hf, 'textbox', pos, 'String', num2str(txt), 'vert', 'bottom', 'EdgeColor','none','FontSize',12,'FitBoxToText','off');

ha=subplot(3,6,5);imshow(FG_WNR6,[]);title('Weiner filter','Fontsize',12);colormap jet;

pos = get(ha, 'position');pos(2)=pos(2)-0.08;pos(1)=pos(1)+0.01;

txt=strcat('PSNR= ',num2str(PSNR_G60_WNR),', CNR= ',num2str(CNR_G60_WNR));

annotation(hf, 'textbox', pos, 'String', num2str(txt), 'vert', 'bottom', 'EdgeColor','none','FontSize',12,'FitBoxToText','off');

ha=subplot(3,6,6);imshow(FG_MMW6,[]);title('MMWF filer','Fontsize',12);colormap jet;

pos = get(ha, 'position');pos(2)=pos(2)-0.08;pos(1)=pos(1)+0.01;

txt=strcat('PSNR= ',num2str(PSNR_G60_MMW),', CNR= ',num2str(CNR_G60_MMW));

annotation(hf, 'textbox', pos, 'String', num2str(txt), 'vert', 'bottom', 'EdgeColor','none','FontSize',12,'FitBoxToText','off');

subplot(3,6,7);imshow(imvol,[]);title('Original','Fontsize',12);colormap jet;

subplot(3,6,8);imshow(NP6,[]);title(["Possion noise=","imnoise(Pixel Value/60)*60"],'Fontsize',12);colormap jet;

ha=subplot(3,6,9);imshow(FP_MED6,[]);title('Median filter','Fontsize',12);colormap jet;

pos = get(ha, 'position');pos(2)=pos(2)-0.08;pos(1)=pos(1)+0.01;

txt=strcat('PSNR= ',num2str(PSNR_P60_MED),', CNR= ',num2str(CNR_P60_MED));

annotation(hf, 'textbox', pos, 'String', num2str(txt), 'vert', 'bottom', 'EdgeColor','none','FontSize',12,'FitBoxToText','off');

ha=subplot(3,6,10);imshow(FP_AVG6,[]);title('Mean filter','Fontsize',12);colormap jet;

pos = get(ha, 'position');pos(2)=pos(2)-0.08;pos(1)=pos(1)+0.01;

txt=strcat('PSNR= ',num2str(PSNR_P60_AVG),', CNR= ',num2str(CNR_P60_AVG));

annotation(hf, 'textbox', pos, 'String', num2str(txt), 'vert', 'bottom', 'EdgeColor','none','FontSize',12,'FitBoxToText','off');

ha=subplot(3,6,11);imshow(FP_WNR6,[]);title('Weiner filter','Fontsize',12);colormap jet;

pos = get(ha, 'position');pos(2)=pos(2)-0.08;pos(1)=pos(1)+0.01;

txt=strcat('PSNR= ',num2str(PSNR_P60_WNR),', CNR= ',num2str(CNR_P60_WNR));

annotation(hf, 'textbox', pos, 'String', num2str(txt), 'vert', 'bottom', 'EdgeColor','none','FontSize',12,'FitBoxToText','off');

ha=subplot(3,6,12);imshow(FP_MMW6,[]);title('MMWF filer','Fontsize',12);colormap jet;

pos = get(ha, 'position');pos(2)=pos(2)-0.08;pos(1)=pos(1)+0.01;

txt=strcat('PSNR= ',num2str(PSNR_P60_MMW),', CNR= ',num2str(CNR_P60_MMW));

annotation(hf, 'textbox', pos, 'String', num2str(txt), 'vert', 'bottom', 'EdgeColor','none','FontSize',12,'FitBoxToText','off');

subplot(3,6,13);imshow(imvol,[]);title('Original','Fontsize',12);colormap jet;

subplot(3,6,14);imshow(NS6,[]);title('Speckle noise 60%','Fontsize',12);colormap jet;

ha=subplot(3,6,15);imshow(FS_MED6,[]);title('Median filter','Fontsize',12);colormap jet;

pos = get(ha, 'position');pos(2)=pos(2)-0.08;pos(1)=pos(1)+0.01;

txt=strcat('PSNR= ',num2str(PSNR_S60_MED),', CNR= ',num2str(CNR_S60_MED));

annotation(hf, 'textbox', pos, 'String', num2str(txt), 'vert', 'bottom', 'EdgeColor','none','FontSize',12,'FitBoxToText','off');

ha=subplot(3,6,16);imshow(FS_AVG6,[]);title('Mean filter','Fontsize',12);colormap jet;

pos = get(ha, 'position');pos(2)=pos(2)-0.08;pos(1)=pos(1)+0.01;

txt=strcat('PSNR= ',num2str(PSNR_S60_AVG),', CNR= ',num2str(CNR_S60_AVG));

annotation(hf, 'textbox', pos, 'String', num2str(txt), 'vert', 'bottom', 'EdgeColor','none','FontSize',12,'FitBoxToText','off');

ha=subplot(3,6,17);imshow(FS_WNR6,[]);title('Weiner filter','Fontsize',12);colormap jet;

pos = get(ha, 'position');pos(2)=pos(2)-0.08;pos(1)=pos(1)+0.01;

txt=strcat('PSNR= ',num2str(PSNR_S60_WNR),', CNR= ',num2str(CNR_S60_WNR));

annotation(hf, 'textbox', pos, 'String', num2str(txt), 'vert', 'bottom', 'EdgeColor','none','FontSize',12,'FitBoxToText','off');

ha=subplot(3,6,18);imshow(FS_MMW6,[]);title('MMWF filer','Fontsize',12);colormap jet;

pos = get(ha, 'position');pos(2)=pos(2)-0.08;pos(1)=pos(1)+0.01;

txt=strcat('PSNR= ',num2str(PSNR_S60_MMW),', CNR= ',num2str(CNR_S60_MMW));

annotation(hf, 'textbox', pos, 'String', num2str(txt), 'vert', 'bottom', 'EdgeColor','none','FontSize',12,'FitBoxToText','off');

%% Export to excel sheet

% PSNR

filename = 'Filtering.xlsx';

T1 = table({PSNR_G10_MED},{PSNR_G20_MED},{PSNR_G30_MED},{PSNR_G40_MED},{PSNR_G50_MED},{PSNR_G60_MED});

writetable(T1,filename,'Sheet','Sheet1','WriteVariableNames',false,'Range',strcat('B3:G3'));

T1 = table({PSNR_G10_AVG},{PSNR_G20_AVG},{PSNR_G30_AVG},{PSNR_G40_AVG},{PSNR_G50_AVG},{PSNR_G60_AVG});

writetable(T1,filename,'Sheet','Sheet1','WriteVariableNames',false,'Range',strcat('B4:G4'));

T1 = table({PSNR_G10_WNR},{PSNR_G20_WNR},{PSNR_G30_WNR},{PSNR_G40_WNR},{PSNR_G50_WNR},{PSNR_G60_WNR});

writetable(T1,filename,'Sheet','Sheet1','WriteVariableNames',false,'Range',strcat('B5:G5'));

T1 = table({PSNR_G10_MMW},{PSNR_G20_MMW},{PSNR_G30_MMW},{PSNR_G40_MMW},{PSNR_G50_MMW},{PSNR_G60_MMW});

writetable(T1,filename,'Sheet','Sheet1','WriteVariableNames',false,'Range',strcat('B6:G6'));

%

T1 = table({PSNR_P10_MED},{PSNR_P20_MED},{PSNR_P30_MED},{PSNR_P40_MED},{PSNR_P50_MED},{PSNR_P60_MED});

writetable(T1,filename,'Sheet','Sheet1','WriteVariableNames',false,'Range',strcat('B10:G10'));

T1 = table({PSNR_P10_AVG},{PSNR_P20_AVG},{PSNR_P30_AVG},{PSNR_P40_AVG},{PSNR_P50_AVG},{PSNR_P60_AVG});

writetable(T1,filename,'Sheet','Sheet1','WriteVariableNames',false,'Range',strcat('B11:G11'));

T1 = table({PSNR_P10_WNR},{PSNR_P20_WNR},{PSNR_P30_WNR},{PSNR_P40_WNR},{PSNR_P50_WNR},{PSNR_P60_WNR});

writetable(T1,filename,'Sheet','Sheet1','WriteVariableNames',false,'Range',strcat('B12:G12'));

T1 = table({PSNR_P10_MMW},{PSNR_P20_MMW},{PSNR_P30_MMW},{PSNR_P40_MMW},{PSNR_P50_MMW},{PSNR_P60_MMW});

writetable(T1,filename,'Sheet','Sheet1','WriteVariableNames',false,'Range',strcat('B13:G13'));

%

T1 = table({PSNR_S10_MED},{PSNR_S20_MED},{PSNR_S30_MED},{PSNR_S40_MED},{PSNR_S50_MED},{PSNR_S60_MED});

writetable(T1,filename,'Sheet','Sheet1','WriteVariableNames',false,'Range',strcat('B17:G17'));

T1 = table({PSNR_S10_AVG},{PSNR_S20_AVG},{PSNR_S30_AVG},{PSNR_S40_AVG},{PSNR_S50_AVG},{PSNR_S60_AVG});

writetable(T1,filename,'Sheet','Sheet1','WriteVariableNames',false,'Range',strcat('B18:G18'));

T1 = table({PSNR_S10_WNR},{PSNR_S20_WNR},{PSNR_S30_WNR},{PSNR_S40_WNR},{PSNR_S50_WNR},{PSNR_S60_WNR});

writetable(T1,filename,'Sheet','Sheet1','WriteVariableNames',false,'Range',strcat('B19:G19'));

T1 = table({PSNR_S10_MMW},{PSNR_S20_MMW},{PSNR_S30_MMW},{PSNR_S40_MMW},{PSNR_S50_MMW},{PSNR_S60_MMW});

writetable(T1,filename,'Sheet','Sheet1','WriteVariableNames',false,'Range',strcat('B20:G20'));

% MSE

filename = 'Filtering.xlsx';

T1 = table({MSE_G10_MED},{MSE_G20_MED},{MSE_G30_MED},{MSE_G40_MED},{MSE_G50_MED},{MSE_G60_MED});

writetable(T1,filename,'Sheet','Sheet1','WriteVariableNames',false,'Range',strcat('K3:P3'));

T1 = table({MSE_G10_AVG},{MSE_G20_AVG},{MSE_G30_AVG},{MSE_G40_AVG},{MSE_G50_AVG},{MSE_G60_AVG});

writetable(T1,filename,'Sheet','Sheet1','WriteVariableNames',false,'Range',strcat('K4:P4'));

T1 = table({MSE_G10_WNR},{MSE_G20_WNR},{MSE_G30_WNR},{MSE_G40_WNR},{MSE_G50_WNR},{MSE_G60_WNR});

writetable(T1,filename,'Sheet','Sheet1','WriteVariableNames',false,'Range',strcat('K5:P5'));

T1 = table({MSE_G10_MMW},{MSE_G20_MMW},{MSE_G30_MMW},{MSE_G40_MMW},{MSE_G50_MMW},{MSE_G60_MMW});

writetable(T1,filename,'Sheet','Sheet1','WriteVariableNames',false,'Range',strcat('K6:P6'));

%

T1 = table({MSE_P10_MED},{MSE_P20_MED},{MSE_P30_MED},{MSE_P40_MED},{MSE_P50_MED},{MSE_P60_MED});

writetable(T1,filename,'Sheet','Sheet1','WriteVariableNames',false,'Range',strcat('K10:P10'));

T1 = table({MSE_P10_AVG},{MSE_P20_AVG},{MSE_P30_AVG},{MSE_P40_AVG},{MSE_P50_AVG},{MSE_P60_AVG});

writetable(T1,filename,'Sheet','Sheet1','WriteVariableNames',false,'Range',strcat('K11:P11'));

T1 = table({MSE_P10_WNR},{MSE_P20_WNR},{MSE_P30_WNR},{MSE_P40_WNR},{MSE_P50_WNR},{MSE_P60_WNR});

writetable(T1,filename,'Sheet','Sheet1','WriteVariableNames',false,'Range',strcat('K12:P12'));

T1 = table({MSE_P10_MMW},{MSE_P20_MMW},{MSE_P30_MMW},{MSE_P40_MMW},{MSE_P50_MMW},{MSE_P60_MMW});

writetable(T1,filename,'Sheet','Sheet1','WriteVariableNames',false,'Range',strcat('K13:P13'));

%

T1 = table({MSE_S10_MED},{MSE_S20_MED},{MSE_S30_MED},{MSE_S40_MED},{MSE_S50_MED},{MSE_S60_MED});

writetable(T1,filename,'Sheet','Sheet1','WriteVariableNames',false,'Range',strcat('K17:P17'));

T1 = table({MSE_S10_AVG},{MSE_S20_AVG},{MSE_S30_AVG},{MSE_S40_AVG},{MSE_S50_AVG},{MSE_S60_AVG});

writetable(T1,filename,'Sheet','Sheet1','WriteVariableNames',false,'Range',strcat('K18:P18'));

T1 = table({MSE_S10_WNR},{MSE_S20_WNR},{MSE_S30_WNR},{MSE_S40_WNR},{MSE_S50_WNR},{MSE_S60_WNR});

writetable(T1,filename,'Sheet','Sheet1','WriteVariableNames',false,'Range',strcat('K19:P19'));

T1 = table({MSE_S10_MMW},{MSE_S20_MMW},{MSE_S30_MMW},{MSE_S40_MMW},{MSE_S50_MMW},{MSE_S60_MMW});

writetable(T1,filename,'Sheet','Sheet1','WriteVariableNames',false,'Range',strcat('K20:P20'));

% CNR

filename = 'Filtering.xlsx';

T1 = table({CNR_G10_MED},{CNR_G20_MED},{CNR_G30_MED},{CNR_G40_MED},{CNR_G50_MED},{CNR_G60_MED});

writetable(T1,filename,'Sheet','Sheet1','WriteVariableNames',false,'Range',strcat('T3:Y3'));

T1 = table({CNR_G10_AVG},{CNR_G20_AVG},{CNR_G30_AVG},{CNR_G40_AVG},{CNR_G50_AVG},{CNR_G60_AVG});

writetable(T1,filename,'Sheet','Sheet1','WriteVariableNames',false,'Range',strcat('T4:Y4'));

T1 = table({CNR_G10_WNR},{CNR_G20_WNR},{CNR_G30_WNR},{CNR_G40_WNR},{CNR_G50_WNR},{CNR_G60_WNR});

writetable(T1,filename,'Sheet','Sheet1','WriteVariableNames',false,'Range',strcat('T5:Y5'));

T1 = table({CNR_G10_MMW},{CNR_G20_MMW},{CNR_G30_MMW},{CNR_G40_MMW},{CNR_G50_MMW},{CNR_G60_MMW});

writetable(T1,filename,'Sheet','Sheet1','WriteVariableNames',false,'Range',strcat('T6:Y6'));

%

T1 = table({CNR_P10_MED},{CNR_P20_MED},{CNR_P30_MED},{CNR_P40_MED},{CNR_P50_MED},{CNR_P60_MED});

writetable(T1,filename,'Sheet','Sheet1','WriteVariableNames',false,'Range',strcat('T10:Y10'));

T1 = table({CNR_P10_AVG},{CNR_P20_AVG},{CNR_P30_AVG},{CNR_P40_AVG},{CNR_P50_AVG},{CNR_P60_AVG});

writetable(T1,filename,'Sheet','Sheet1','WriteVariableNames',false,'Range',strcat('T11:Y11'));

T1 = table({CNR_P10_WNR},{CNR_P20_WNR},{CNR_P30_WNR},{CNR_P40_WNR},{CNR_P50_WNR},{CNR_P60_WNR});

writetable(T1,filename,'Sheet','Sheet1','WriteVariableNames',false,'Range',strcat('T12:Y12'));

T1 = table({CNR_P10_MMW},{CNR_P20_MMW},{CNR_P30_MMW},{CNR_P40_MMW},{CNR_P50_MMW},{CNR_P60_MMW});

writetable(T1,filename,'Sheet','Sheet1','WriteVariableNames',false,'Range',strcat('T13:Y13'));

%

T1 = table({CNR_S10_MED},{CNR_S20_MED},{CNR_S30_MED},{CNR_S40_MED},{CNR_S50_MED},{CNR_S60_MED});

writetable(T1,filename,'Sheet','Sheet1','WriteVariableNames',false,'Range',strcat('T17:Y17'));

T1 = table({CNR_S10_AVG},{CNR_S20_AVG},{CNR_S30_AVG},{CNR_S40_AVG},{CNR_S50_AVG},{CNR_S60_AVG});

writetable(T1,filename,'Sheet','Sheet1','WriteVariableNames',false,'Range',strcat('T18:Y18'));

T1 = table({CNR_S10_WNR},{CNR_S20_WNR},{CNR_S30_WNR},{CNR_S40_WNR},{CNR_S50_WNR},{CNR_S60_WNR});

writetable(T1,filename,'Sheet','Sheet1','WriteVariableNames',false,'Range',strcat('T19:Y19'));

T1 = table({CNR_S10_MMW},{CNR_S20_MMW},{CNR_S30_MMW},{CNR_S40_MMW},{CNR_S50_MMW},{CNR_S60_MMW});

writetable(T1,filename,'Sheet','Sheet1','WriteVariableNames',false,'Range',strcat('T20:Y20'));

%

%% Contrast Resolution CR

% CR

filename = 'Filtering.xlsx';

T1 = table({CR_G10_MED},{CR_G20_MED},{CR_G30_MED},{CR_G40_MED},{CR_G50_MED},{CR_G60_MED});

writetable(T1,filename,'Sheet','Sheet1','WriteVariableNames',false,'Range',strcat('AC3:AH3'));

T1 = table({CR_G10_AVG},{CR_G20_AVG},{CR_G30_AVG},{CR_G40_AVG},{CR_G50_AVG},{CR_G60_AVG});

writetable(T1,filename,'Sheet','Sheet1','WriteVariableNames',false,'Range',strcat('AC4:AH4'));

T1 = table({CR_G10_WNR},{CR_G20_WNR},{CR_G30_WNR},{CR_G40_WNR},{CR_G50_WNR},{CR_G60_WNR});

writetable(T1,filename,'Sheet','Sheet1','WriteVariableNames',false,'Range',strcat('AC5:AH5'));

T1 = table({CR_G10_MMW},{CR_G20_MMW},{CR_G30_MMW},{CR_G40_MMW},{CR_G50_MMW},{CR_G60_MMW});

writetable(T1,filename,'Sheet','Sheet1','WriteVariableNames',false,'Range',strcat('AC6:AH6'));

%

T1 = table({CR_P10_MED},{CR_P20_MED},{CR_P30_MED},{CR_P40_MED},{CR_P50_MED},{CR_P60_MED});

writetable(T1,filename,'Sheet','Sheet1','WriteVariableNames',false,'Range',strcat('AC10:AH10'));

T1 = table({CR_P10_AVG},{CR_P20_AVG},{CR_P30_AVG},{CR_P40_AVG},{CR_P50_AVG},{CR_P60_AVG});

writetable(T1,filename,'Sheet','Sheet1','WriteVariableNames',false,'Range',strcat('AC11:AH11'));

T1 = table({CR_P10_WNR},{CR_P20_WNR},{CR_P30_WNR},{CR_P40_WNR},{CR_P50_WNR},{CR_P60_WNR});

writetable(T1,filename,'Sheet','Sheet1','WriteVariableNames',false,'Range',strcat('AC12:AH12'));

T1 = table({CR_P10_MMW},{CR_P20_MMW},{CR_P30_MMW},{CR_P40_MMW},{CR_P50_MMW},{CR_P60_MMW});

writetable(T1,filename,'Sheet','Sheet1','WriteVariableNames',false,'Range',strcat('AC13:AH13'));

%

T1 = table({CR_S10_MED},{CR_S20_MED},{CR_S30_MED},{CR_S40_MED},{CR_S50_MED},{CR_S60_MED});

writetable(T1,filename,'Sheet','Sheet1','WriteVariableNames',false,'Range',strcat('AC17:AH17'));

T1 = table({CR_S10_AVG},{CR_S20_AVG},{CR_S30_AVG},{CR_S40_AVG},{CR_S50_AVG},{CR_S60_AVG});

writetable(T1,filename,'Sheet','Sheet1','WriteVariableNames',false,'Range',strcat('AC18:AH18'));

T1 = table({CR_S10_WNR},{CR_S20_WNR},{CR_S30_WNR},{CR_S40_WNR},{CR_S50_WNR},{CR_S60_WNR});

writetable(T1,filename,'Sheet','Sheet1','WriteVariableNames',false,'Range',strcat('AC19:AH19'));

T1 = table({CR_S10_MMW},{CR_S20_MMW},{CR_S30_MMW},{CR_S40_MMW},{CR_S50_MMW},{CR_S60_MMW});

writetable(T1,filename,'Sheet','Sheet1','WriteVariableNames',false,'Range',strcat('AC20:AH20'));

%

%% Degree of smoothness DOS

% DOS

filename = 'Filtering.xlsx';

T1 = table({DOS_G10_MED},{DOS_G20_MED},{DOS_G30_MED},{DOS_G40_MED},{DOS_G50_MED},{DOS_G60_MED});

writetable(T1,filename,'Sheet','Sheet1','WriteVariableNames',false,'Range',strcat('AL3:AQ3'));

T1 = table({DOS_G10_AVG},{DOS_G20_AVG},{DOS_G30_AVG},{DOS_G40_AVG},{DOS_G50_AVG},{DOS_G60_AVG});

writetable(T1,filename,'Sheet','Sheet1','WriteVariableNames',false,'Range',strcat('AL4:AQ4'));

T1 = table({DOS_G10_WNR},{DOS_G20_WNR},{DOS_G30_WNR},{DOS_G40_WNR},{DOS_G50_WNR},{DOS_G60_WNR});

writetable(T1,filename,'Sheet','Sheet1','WriteVariableNames',false,'Range',strcat('AL5:AQ5'));

T1 = table({DOS_G10_MMW},{DOS_G20_MMW},{DOS_G30_MMW},{DOS_G40_MMW},{DOS_G50_MMW},{DOS_G60_MMW});

writetable(T1,filename,'Sheet','Sheet1','WriteVariableNames',false,'Range',strcat('AL6:AQ6'));

T1 = table({DOS_P10_MED},{DOS_P20_MED},{DOS_P30_MED},{DOS_P40_MED},{DOS_P50_MED},{DOS_P60_MED});

writetable(T1,filename,'Sheet','Sheet1','WriteVariableNames',false,'Range',strcat('AL10:AQ10'));

T1 = table({DOS_P10_AVG},{DOS_P20_AVG},{DOS_P30_AVG},{DOS_P40_AVG},{DOS_P50_AVG},{DOS_P60_AVG});

writetable(T1,filename,'Sheet','Sheet1','WriteVariableNames',false,'Range',strcat('AL11:AQ11'));

T1 = table({DOS_P10_WNR},{DOS_P20_WNR},{DOS_P30_WNR},{DOS_P40_WNR},{DOS_P50_WNR},{DOS_P60_WNR});

writetable(T1,filename,'Sheet','Sheet1','WriteVariableNames',false,'Range',strcat('AL12:AQ12'));

T1 = table({DOS_P10_MMW},{DOS_P20_MMW},{DOS_P30_MMW},{DOS_P40_MMW},{DOS_P50_MMW},{DOS_P60_MMW});

writetable(T1,filename,'Sheet','Sheet1','WriteVariableNames',false,'Range',strcat('AL13:AQ13'));

T1 = table({DOS_S10_MED},{DOS_S20_MED},{DOS_S30_MED},{DOS_S40_MED},{DOS_S50_MED},{DOS_S60_MED});

writetable(T1,filename,'Sheet','Sheet1','WriteVariableNames',false,'Range',strcat('AL17:AQ17'));

T1 = table({DOS_S10_AVG},{DOS_S20_AVG},{DOS_S30_AVG},{DOS_S40_AVG},{DOS_S50_AVG},{DOS_S60_AVG});

writetable(T1,filename,'Sheet','Sheet1','WriteVariableNames',false,'Range',strcat('AL18:AQ18'));

T1 = table({DOS_S10_WNR},{DOS_S20_WNR},{DOS_S30_WNR},{DOS_S40_WNR},{DOS_S50_WNR},{DOS_S60_WNR});

writetable(T1,filename,'Sheet','Sheet1','WriteVariableNames',false,'Range',strcat('AL19:AQ19'));

T1 = table({DOS_S10_MMW},{DOS_S20_MMW},{DOS_S30_MMW},{DOS_S40_MMW},{DOS_S50_MMW},{DOS_S60_MMW});

writetable(T1,filename,'Sheet','Sheet1','WriteVariableNames',false,'Range',strcat('AL20:AQ20'));

%

disp('Data has been written to excel sheet, check Filtering.xlsx in the current folder!');

%%%%%%%%%%%%%%%%%%%%%%%%%%%%%%%%%%%%%%%%%%%%%%%%%%%%%%%%%%%%%%%%

| **M-file name** | Cnr.m |
| --- | --- |
| **Type** | Fun |
| **Description** | Funtion to compute the contrast to noise ratio and the contrast resolution. |
| **comments** | Novel: Written by us based on paper (249) |

**Code:**

function [cnr_val,CR] = cnr(img1,bw,img2,N)

%% Written at 21-9-2021 based on paper:

% https://www.researchgate.net/publication/228664578_Practical_Evaluation_of_Image_Quality_in_Computed_Radiographic_CR_Imaging_Systems

% https://en.wikipedia.org/wiki/Contrast_resolution

% https://en.wikipedia.org/wiki/Contrast-to-noise_ratio

%% This function computes the contrast to noise ratio and the contrast

%% resolution

% img1 is the original image

% imag2 is the filtered image

% N is the noisy image (img1+noise)

%%

A = double(img1); B = double(img2);

N = double(N);

sigNois=A-N; % compute the noise only

mn1=mean2(A(bw==1)); % mean value of original image

mn2=mean2(B(bw==1)); % mean value of filtered image

sd=std2(sigNois(bw==1)); % std of the noise

dif=abs(mn1-mn2); % mean difference

cnr_val=dif./sd; % cnr value

% contrast resolution

CR=dif/(mn1+mn2);

End

%%%%%%%%%%%%%%%%%%%%%%%%%%%%%%%%%%%%%%%%%%%%%%%%%%%%%%%%%%

| **M-file name** | dos.m |
| --- | --- |
| **Type** | Fun |
| **Description** | Function to computes the degree of smoothness of an image according to the sum of magnitude (sqrt of the square of all gradients). |
| **comments** | Written by us at 21-9-2021 based on information from (250) |

**Code:**

function [dos] = dos(img1)

%% Written at 21-9-2021 based on information of https://stats.stackexchange.com/questions/379666/measure-of-smoothness

%% this function computes the degree of smoothness of an image according to

%% the sum of magnitude (sqrt of the square of all gradients)

% img is the image to compute dos for

% dos is the degree of smoothness

[m n l] = size(img1);

dos = sum(sum(sum(sqrt(gradient(gradient(gradient(double(img1)))).^2))))/(m*n*l);

end

**Final note:**

We applied an experiments to check the best filter can be used for our algorithm (median, mean, wiener and MMWF). The clinic_heart_disease_system.m Matlab file is modified to satisfy this experiment so that 4 different applications of each stage of our algorithm are computed and compared. The results of this experiment are located in file: Platform.xlsx (4 sheets, each of which represents the corresponding filter results).
