# Supplementary material for: Cardiotoxicity detection tool for breast cancer chemotherapy: a retrospective study
Source: PeerJ Comput Sci. 2024 Aug 2;12:e2230. doi: 10.7717/peerj-cs.2230 (PMC11323080; doi:10.7717/peerj-cs.2230)
Supplement: Supplemental Information 16 [file peerj-cs-10-2230-s016.docx]

**
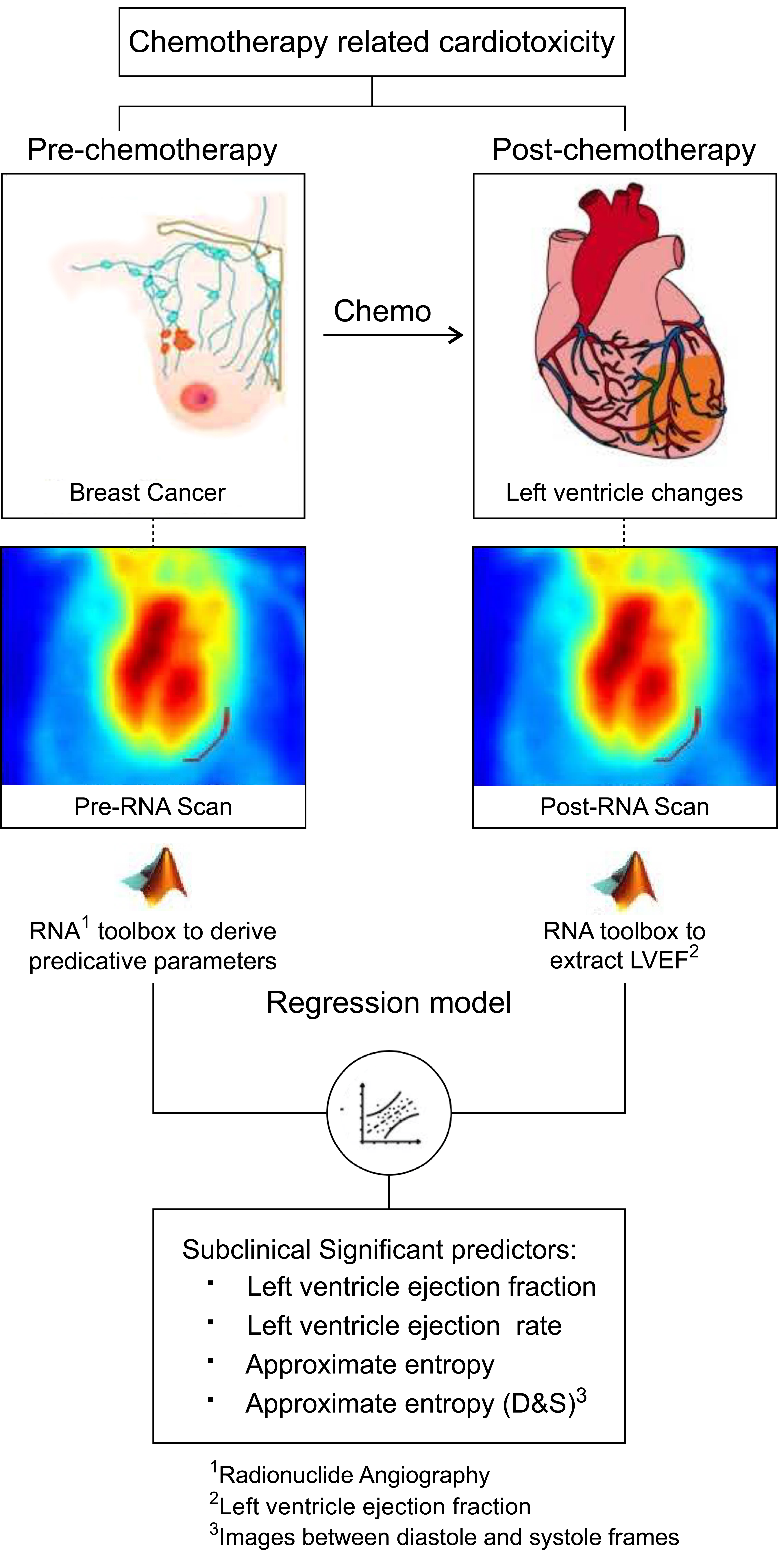
**

**Graphical abstract:** Subclinical left ventricle ejection fraction changes in patients undergoing cardiotoxic chemotherapy were detectable using predictive parameters derived from baseline radionuclide angiography scans using a novel MATLAB® code (i.e., RNA toolbox).
