# Supplementary material for: Cardiotoxicity detection tool for breast cancer chemotherapy: a retrospective study
Source: PeerJ Comput Sci. 2024 Aug 2;12:e2230. doi: 10.7717/peerj-cs.2230 (PMC11323080; doi:10.7717/peerj-cs.2230)

Supplementary Table 1: Definitions of main parameters derived using RNA toolbox from RNA images.

| Parameter | Function and definition |
| --- | --- |
| LVEF | The LVEF depending on activity within background-corrected systole and diastole images. It is defined as the fraction of chamber volume ejected in relation to blood volume in ventricle at end-diastolic phase (ED). |
| Mean ϕ | The Mean phase angle of all 16 RNA images. it defines the point time activity curve (TAC) at which the Fourier function recaches its peak (i.e. onset of contraction). |
| Phase image | It is the images that represent the timing of contraction in RNA image. |
| SD ϕ | The standard deviation of the phase angle. It is the simplest parameter to quantify cardiac dyssynchrony |
| Amplitude image | Shows the amplitude of each phase angle distribution within RNA image. |
| Entropy | The entropy (regularity of RNA data series). It is used to measure randomness in LV. |
| Approximate entropy ApEn | The entropy of the system. It is defined similar to entropy, but considering the system’s variability. |
| Bounded Approximate Entropy | The entropy of the system (considering long-term and short-term variations and system characteristics ‘deterministic vs stochastic’ ) |
| Epsilon (ε) | A parameter of the bounded-ApEn model that is combined with ApEn to determine the uncertainty or variability of stochastic signals. |
| Synchrony | Describes how ventricular myocytes are coordinated in the LV myocardium when contracting. |
| Lung to left ventricle ratio (LHR) | Lung to heart (LV) ratio. It is an indicator of heart failure. |
| Circularity systole | The circularity index of the LV systole image |
| Circularity diastole | The circularity index of the LV diastole image |
| Elongation systole | The elongation index of the LV systole image |
| Elongation diastole | The elongation index of the LV diastole image |

Supplementary Table 2: Chemotherapeutic drugs with corresponding mean doses ± standard deviation (STD).

| Drug (mg) | cyclophosphamide | Epirubicin | Fluorouracil | Docetaxel | Trastuzumab |
| --- | --- | --- | --- | --- | --- |
| Mean dose ± (STD) | 4241 ± (2579) | 676 ± (492) | 4008 ± (3175) | 636±(246) | 6668±(7818) |

Supplementary Table 3: Mean background grey values (Bkg1 – Bkg6 and eSoft Bkg) of different locations with corresponding standard deviations.

|  | Mean | Std. Deviation |
| --- | --- | --- |
| Bkg1 | 25 | 2.9 |
| Bkg2 | 23 | 2.4 |
| Bkg3 | 25 | 3.9 |
| Bkg4 | 25 | 2.5 |
| Bkg5 | 24 | 1.9 |
| Bkg6 | 24 | 2.3 |
| eSoft Bkg | 25 | 1.7 |

Supplementary Table 4: Inter-Bkg Correlation Matrix: Show correlation between backgrounds (Bkg1 – Bkg6 and eSoft Bkg). The highest correlation has been found between eSoft and Bkg1 (highlighted).

|  | Bkg1^a^ | Bkg2 | Bkg3 | Bkg4 | Bkg5 | Bkg16 | eSoft Bkg |
| --- | --- | --- | --- | --- | --- | --- | --- |
| Bkg1 | 1.000 | .170 | .066 | .289 | .058 | .327 | .793 |
| Bkg2 | .170 | 1.000 | .437 | .005 | .436 | -.012 | -.115 |
| Bkg3 | .066 | .437 | 1.000 | -.244 | .091 | .069 | .024 |
| Bkg4 | .289 | .005 | -.244 | 1.000 | .031 | .034 | .231 |
| Bkg5 | .058 | .436 | .091 | .031 | 1.000 | -.047 | -.114 |
| Bkg6 | .327 | -.012 | .069 | .034 | -.047 | 1.000 | .289 |
| eSoft Bkg | .793 | -.115 | .024 | .231 | -.114 | .289 | 1.000 |

Bkg^a^ Background

Supplementary Figure 1

Supplementary Figure 1: An image generated from the code in reference 32, which automatically generates the region of interest (ROI) surrounding the heart and automatically draws 6 backgrounds in different spatial regions (1-6) around the heart’s ROI. The lines (1-6) represent the location of the center of the background ROI.


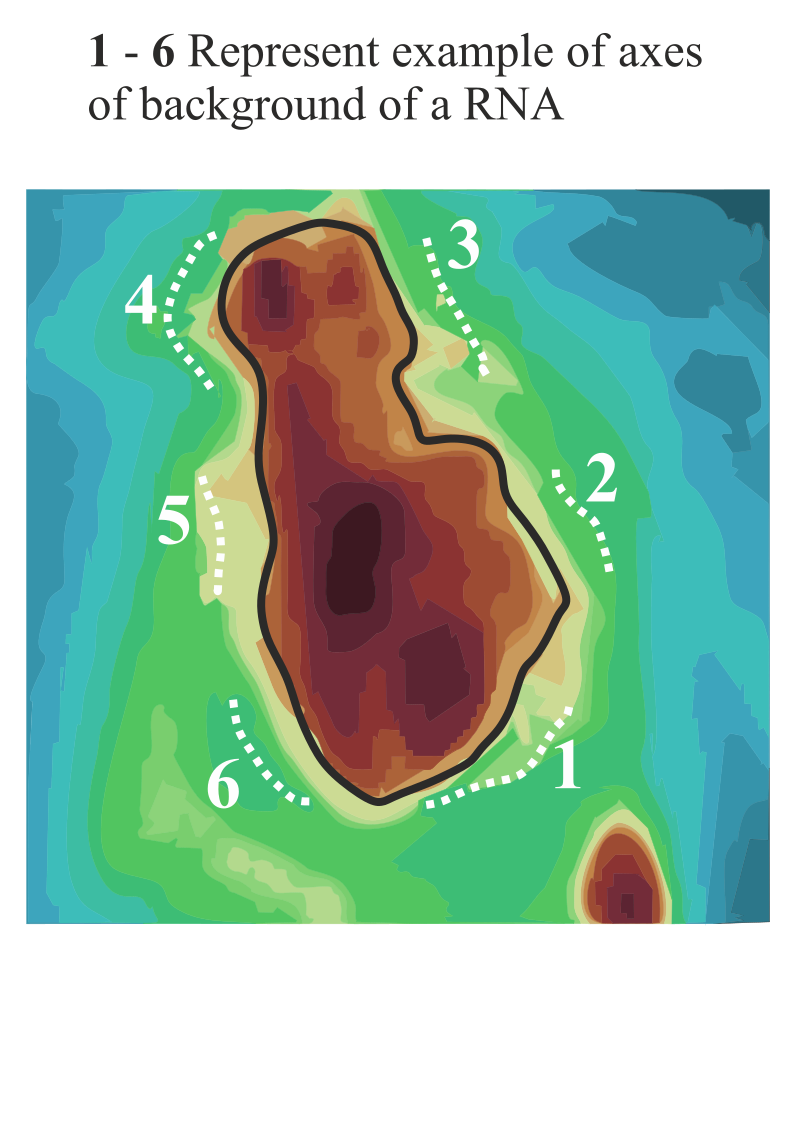

Supplement: Supplemental Information 17 [file peerj-cs-10-2230-s017.docx]
